# Building-Up of a DNA Barcode Library for True Bugs (Insecta: Hemiptera: Heteroptera) of Germany Reveals Taxonomic Uncertainties and Surprises

**DOI:** 10.1371/journal.pone.0106940

**Published:** 2014-09-09

**Authors:** Michael J. Raupach, Lars Hendrich, Stefan M. Küchler, Fabian Deister, Jérome Morinière, Martin M. Gossner

**Affiliations:** 1 Molecular Taxonomy of Marine Organisms, German Center of Marine Biodiversity (DZMB), Senckenberg am Meer, Wilhelmshaven, Germany; 2 Sektion Insecta varia, Bavarian State Collection of Zoology (SNSB – ZSM), München, Germany; 3 Department of Animal Ecology II, University of Bayreuth, Bayreuth, Germany; 4 Taxonomic coordinator – Barcoding Fauna Bavarica, Bavarian State Collection of Zoology (SNSB – ZSM), München, Germany; 5 Terrestrial Ecology Research Group, Department of Ecology and Ecosystem Management, Technische Universität München, Freising-Weihenstephan, Germany; Consiglio Nazionale delle Ricerche (CNR), Italy

## Abstract

During the last few years, DNA barcoding has become an efficient method for the identification of species. In the case of insects, most published DNA barcoding studies focus on species of the Ephemeroptera, Trichoptera, Hymenoptera and especially Lepidoptera. In this study we test the efficiency of DNA barcoding for true bugs (Hemiptera: Heteroptera), an ecological and economical highly important as well as morphologically diverse insect taxon. As part of our study we analyzed DNA barcodes for 1742 specimens of 457 species, comprising 39 families of the Heteroptera. We found low nucleotide distances with a minimum pairwise K2P distance <2.2% within 21 species pairs (39 species). For ten of these species pairs (18 species), minimum pairwise distances were zero. In contrast to this, deep intraspecific sequence divergences with maximum pairwise distances >2.2% were detected for 16 traditionally recognized and valid species. With a successful identification rate of 91.5% (418 species) our study emphasizes the use of DNA barcodes for the identification of true bugs and represents an important step in building-up a comprehensive barcode library for true bugs in Germany and Central Europe as well. Our study also highlights the urgent necessity of taxonomic revisions for various taxa of the Heteroptera, with a special focus on various species of the Miridae. In this context we found evidence for on-going hybridization events within various taxonomically challenging genera (e.g. *Nabis* Latreille, 1802 (Nabidae), *Lygus* Hahn, 1833 (Miridae), *Phytocoris* Fallén, 1814 (Miridae)) as well as the putative existence of cryptic species (e.g. *Aneurus avenius* (Duffour, 1833) (Aradidae) or *Orius niger* (Wolff, 1811) (Anthocoridae)).

## Introduction

True bugs or Heteroptera are a highly diverse taxon of the Hemiptera which count as one of the big five insect orders in terms of species richness [1]. Up to date, more than 42,000 species are described worldwide [2]. While the diversity concentrates in the tropics and subtropics, not less than 9,365 species are known from the Palaearctic region [3], and more than 1,100 from Central Europe [4]. True bugs evolved an astonishing diversity of morphological structures, and their ecological diversity is formidable. They colonized almost all ecosystems worldwide except the deep sea and Polar region. Species of the genus *Halobates* Eschscholtz, 1822 are unique among insects in spending their entire life on the surface of the open ocean [5], whereas species of the family Aphelocheiridae spent most of their life submerged in streams and lakes [6]. Beside species sucking on plant sap including extremely specialized species such as gall inducers (e.g. species of the genus *Copium* Thunberg, 1822 [7]), fungi hyphen (e.g. Aradidae) or other arthropods (e.g. the genus *Nabis* Latreille, 1802), also vertebrate ectoparasites (e.g. the family Cimicidae [8]), kleptoparasites (e.g. among specimens of *Velia caprai* Tamanini, 1947 [9], or in spider webs [10]) are described). Heteroptera are known to be serious pest species [11] as well as effective biocontrol agents [12]. Mutualistic interactions with ants [13] occur in some species and as subsocial behavior parental care by females is a widely known phenomenon which independently developed in several Heteroptera families [14], but also male caring has been described (e.g. the giant water bugs of the family Belastomatidae [15]). In general, mating strategies are very diverse in Heteroptera, incl. traumatic insemination in bedbugs and anthocorids (e.g. [16]). The diversity in ecological requirements and their specificity to particular habitats or host plants makes them suitable ecological [17]–[19] and biodiversity indicators [20], [21] as well as indicators for climate change [22].

Due to their high ecological and economic (e.g. as potential pest species) importance a reliable species identification is highly demanded. Identification of Heteroptera by morphological characteristics is, however, time consuming, and in some groups also very difficult such as e.g. in the Miridae, the by far most species rich family. The identification of nymphal stages or eggs is even more critical or even impossible, although necessary, e.g. for early stage detection of potential pest species. Due to high levels of morphological variation in diagnostic characteristics as result of putative hybridizations it is not surprising that in some genera the taxonomic status of various species is subject of discussion, e.g. various species of the genus *Lygus* Hahn, 1833 of the Miridae [23]. Therefore, molecular methods are seen as promising complementary tool to morphological based methods.

In this context, DNA barcoding has become an effective molecular method for species identification regardless of the development stage of the analyzed specimen [24]–[26], representing an efficient approach for valid species identification for large-scale biodiversity studies [27], [28]. For the Metazoa, the classical barcode fragment consists of a 658 base pair (bp) fragment of the mitochondrial cytochrome *c* oxidase subunit 1 (CO1) gene [24], [25]. The idea of DNA barcoding relies on the concept that each species will most likely have unique DNA barcodes and that intraspecific CO1 variation is typically lower than the interspecific variability. As consequence, a so-called barcoding gap is given which allows an undoubted molecular species identification [24]–[26]. Despite the fact that DNA barcoding has been criticized, feared, not accepted and/or simply not understood [29]–[32], DNA barcodes have become an important and increasingly used tool as part of an integrative taxonomy in modern species descriptions [33]–[38] as well as various other biological disciplines, e.g. forensics [39], [40], pest biology [41], and conservation biology [42], [43]. Not surprisingly, new insights into ecology and species biology have already emerged from various DNA barcode studies [44], and the rise of new-generation sequencing technologies will increase the use of DNA barcoding as part of molecular biomonitoring studies [45]–[48].

While DNA barcoding has been successfully used for the molecular identification of a broad variety of insect taxa, including Ephemeroptera [49], [50], Trichoptera [51], Lepidoptera [52]–[55], Hymenoptera [28], [56] and Coleoptera [57]–[61], studies analyzing Heteroptera are still rare. However, pioneering works revealed the potential of this modern approach for a valid identification of true bugs [23], [62]–[65]. For a few selected taxa, even so-called micro-barcodes have been tested [66].

In this study we present the first comprehensive DNA barcode analysis of 1742 specimens representing 457 species of the Heteroptera of Germany. To evaluate the efficiency of DNA barcoding, our data set includes a variety of morphological highly similar and putatively closely related and/or sibling species within taxonomically difficult genera, such as *Nabis* (Nabidae), *Lygus* or *Phytocoris* Fallén, 1814 (both Miridae).

## Material and Methods

### Sampling of specimens

All analyzed true bugs were collected between 2005 and 2012 using various methods (i.e. hand collecting, sweep-netting, Malaise-, window- and pitfall-traps). Most specimens were collected in nine different federal states of Germany (*n* = 1680; 96.5%) (see Appendix S1), but for comparison, some selected specimens from other countries as Austria (29; 1.6%), France (21; 1.2%), Italy (7; 0.4%) and Switzerland (5; 0.3%) were also included in our analysis. All specimens were stored in ethanol (70 or 96%) or pinned. The number of analyzed specimens per species ranged from one to a maximum of 16 in the case of *Orius minutus* (Linnaeus, 1758) (Anthocoridae) and *Plagiognathus arbustorum* (Fabricius, 1794) (Miridae) (see Appendix S1). Individuals were identified to species level either by two of the authors (MMG, SMK) or by other taxonomic specialists, using appropriate taxonomic literature [67]–[84].

### DNA sequencing and data depository

Laboratory operations were carried out at the Canadian Center for DNA Barcoding (CCDB), University of Guelph, following standardized high-throughput protocols for DNA barcode amplification and sequencing [85], [86]. For specimens with a body length >3 mm one or two legs were removed from each individual and used for DNA extraction, while complete specimens were used for specimen ≤3 mm. All relevant voucher information, taxonomic classifications, images, DNA barcodes, used primer pairs and trace files are publicly accessible in the project GEBUG in the Barcode of Life Datasystems (BOLD; www.boldsystems.org) [87], [88], which represents a fused project of a part of the Fauna Bavaria campaign [89] and EUBUG. In 2006 the Bavarian State Collection of Zoology (ZSM) started a close collaboration with the Biodiversity Institute of Ontario (‘BIO’, Guelph, Canada) to assemble a DNA barcode library for all animals, plants and fungi known to occur in Bavaria in the framework of the International Barcode of Life Initiative (‘iBOL’). Over the past seven years, the ZSM submitted tissue samples from more than 150,000 identified vouchers belonging to more than 40,000 insect species. EUBUG was a private project initiated by MJR and MMG in 2010. Photos of all specimens as well as all sequence records are available on BOLD (public data set http://dx.doi.org/10.5883/DS-HETGER) whereas sequence data are also deposited in GenBank (accession numbers see Appendix S2).

### DNA barcode analysis

The analysis of intra- and interspecific nucleotide variability of the analyzed true bug species with barcodes >400 base pairs, representing a barcode fragment size of more than 60%, were based on the Kimura 2-parameter (K2P; [90]), using the analytical tools on BOLD (align sequences: BOLD aligner; ambiguous base/gap handling: pairwise deletion). All barcodes were subject to the Barcode Index Number (BIN) system of BOLD, which clusters sequences to produce operational taxonomic units that closely correspond to species [87]. BINs are unique in that clusters are indexed in a regimented way so genetically identical taxa encountered in different studies reside under shared identifiers [88]. Based on these suggestions we used a threshold of 2.2% for a rough differentiation of low and high intraspecific as well as interspecific K2P distances [87]. We performed a neighbour-joining cluster analysis [91] with non-parametric bootstrap replicates (*n* = 1000) [92] for a graphical representation of patterns of nucleotide divergences based on K2P distances using MEGA 6.4 [93] for all analyzed specimens. Furthermore, we constructed statistical parsimony networks exemplarily for case studies of haplotype sharing including *Charagochilus gyllenhalii* (Fallén, 1807) and *C. weberi* Wagner, 1953 (Miridae), four selected species of the genus *Nabis* (Nabidae), three species of *Lygus* (Miridae), and *Orius niger* (Wolff, 1811) (Anthocoridae) as example of high intraspecific distances and distinct lineages with TCS 1.21 [94], using default settings. The use of statistical parsimony networks allows an easy identification of haplotype sharing between species as consequence of on-going hybridization or recent speciation. Such networks also allow the detection of distinct lineages and therefore the putative existence of cryptic species [95].

Finally, we used the Automatic Barcode Gap Program ABGD [96] to analyze the hypothesis of independent lineages and presence of putative cryptic species. This tool aims to identify barcode gaps by defining the first significant gap that occurs in a dataset of aligned sequences. The program computes pairwise distances and splits the dataset in primary partitions based on an estimated value of intraspecific variability. Following this, the program recursively applies this procedure on these primary partitions to get finer secondary partitions until no more splitting can be performed under the given priors. Here, partitioning of the data needs i) an estimator for the intraspecific variability and ii) a prior for the relative gap width, which is used to decide, weather a discovered gap is intra- or interspecific, by excluding small gaps from the analysis. As consequence of the use of different prior values, the number and composition of groups can change. To make sure that our results are not only an artifact of our used priors we altered both values and visualized all results over the range of both values. In doing so we used a Perl-script APE (ABGD Parameter Explorer, see Appendix S3) combined with the ABGD offline version and changed the input 100 times. During a single run, ABGD itself increased the maximum intraspecific divergence from a value P_min_ = 0.001 to P_max_ = 0.1 in a given number of steps. In our analysis we repeated the calculation set for the number of steps between both extreme values (*n* = 100) to achieve a finer resolution. Consequently, these steps were computed for each primary and secondary partition. APE was also used to increase the relative gap width from x = 0.1 to x = 10 and to analyze and document the output of each run. As result, the first run showed groups separated by small gaps whereas the last possible run defined groups by very large gaps. In contrast to the original ABGD software, our approach produced results for datasets which caused no results when using the default setting of x = 1.5, considering the relative gap width as important parameter. Besides these modifications we also set the distance method to K2P. We performed this analysis on subsets of our data containing species on family level for which at least more than one BIN has been assigned. The output of our script was visualized in color-coded matrix plots, showing the number of groups which were found for each combination of the two altered parameters over the complete run (not shown). As final result of this analysis we got the intraspecific divergence estimator P, ranging from 0.1 to 10%. In our analysis this value indicated the size of the barcoding gap which has to be used to cluster all morphologically classified sequences of a species as one group. Values of P were correlated with the observed maximum pairwise distances. However, the primary hypothesis is difficult to discuss and compute using ABGD with three or less specimens [96].

## Results

**Figure 1:**
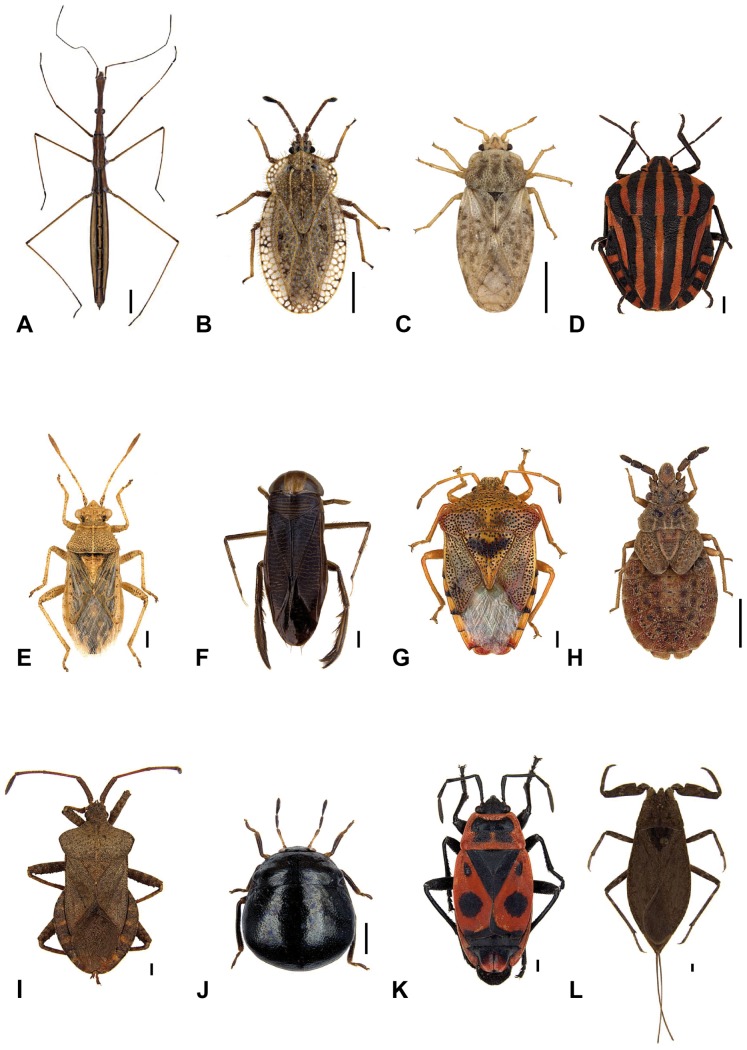
Images of selected and now barcoded species of the Heteroptera of Germany. A: *Hydrometra gracilenta* Horváth, 1899 (Hydrometridae), B: *Tingis ampliata* (Herrich-Schaeffer, 1838) (Tingidae), C: *Piesma maculatum* (Laporte, 1833) (Piesmatidae), D: *Graphosoma lineatum* (Linnaeus, 1758) (Pentatomidae), E: *Rhopalus parumpunctatus* Schilling, 1829 (Rhopalidae), F: *Hesperocorixa sahlbergi* (Fieber, 1848) (Corixidae), G: *Elasmucha grisea* (Linnaeus, 1758) (Acanthosomatidae), H: *Aradus cinnamomeus* Panzer, 1806 (Aradidae), I: *Coreus marginatus* (Linnaeus, 1758) (Coreidae), J: *Coptosoma scutellatum* (Geoffroy, 1785) (Plataspidae), K: *Pyrrhocoris apterus* (Linnaeus, 1758) (Pyrrhocoridae), and L: *Nepa cinerea* Linnaeus, 1758 (Nepidae). Scale bars  = 1 mm.

The presented barcode library comprised 1742 specimens of 457 species, representing 39 families of the Heteroptera (see Fig. 1 for representative species of some selected families). Analyzed fragments lengths ranged from a minimum of 402 bp to the full fragment size of 658 bp (Appendix S4). The average fragment size differed greatly among Heteroptera families. Among families with high number of analyzed specimens Rhyparochromidae and Rhopalidae showed, for example, generally high fragment length while Anthocoridae, Miridae and Pentatomidae showed much greater variation (Appendix S5).

The mean sequence composition in the generated sequences were A = 31.8%, C = 18.6%, G = 16.0% and T = 33.6%, revealing a high AT-content (65.4%) as it is typically known from this gene fragment for arthropods. Our analysis revealed unique BINs for 408 species (89.3%) and two BINs for 13 species (2.9%) (see Appendix S6). Furthermore, we found three BINs for the 12 analyzed specimens of *Stenodema calcarata* (Fallén, 1807) (Miridae) (0.2%). As consequence of short fragment lengths (400–450 bp) or presence of multiple nucleotide ambiguities, 35 species (7.6%) were without BINs. We observed considerable overlaps between intraspecific (0–23.31%) and interspecific divergences (0–25.95%; see Appendix S6 and S7). Our BIN analysis revealed a low nucleotide variability with a minimum pairwise K2P distance <2.2% for 21 species pairs (39 species) and 20 BINs (Table 1) and eight species with maximum pairwise distances (MPDs) >2.2% and one corresponding BIN (Table 2). Furthermore, 15 species showed MPDs >2.2% and at least two corresponding BINs (Table 3). For these species we also calculated the intraspecific divergence estimator P using the modified ABGD analysis (Table 3). A summary of all matrix plots is part of the appendix (Appendix S8).

**Table 1:** A table of 21 species pairs of the analyzed Heteroptera with a minimum pairwise distance (K2P) of 0 to 2.2% and corresponding BINs.

Family	Species 1	Species 2	Minimum pairwise K2P distance (%)	BIN
**Acanthosomatidae**	*Elasmostethus interstinctus* (3)	*Elasmostethus minor* (1)	0	ABZ2225
**Lygaeidae**	*Arocatus longiceps* (12)	*Arocatus roeselii* (2)	0	AAY8974
**Miridae**	*Charagochilus gyllenhalii* (3)	*Charagochilus weberi* (6)	0	AAY9446
**Miridae**	*Lygus gemellatus* (2)	*Lygus pratensis* (7)	0	AAY8966
**Miridae**	*Lygus wagneri* (1)	*Lygus gemellatus* (2)	0	AAY8966
**Miridae**	*Strongylocoris leucocephalus* (5)	*Strongylocoris steganoides* (4)	0	ACD1310
**Miridae**	*Trigonotylus caelestialium* (10)	*Trigonotylus pulchellus* (2)	0	AAF9949
**Nabidae**	*Nabis brevis* (11)	*Nabis rugosus* (6)	0	AAZ3346
**Nabidae**	*Nabis ericetorum* (5)	*Nabis brevis* (11)	0	AAZ3346
**Scutelleridae**	*Eurygaster maura* (5)	*Eurygaster testudinaria* (5)	0	n. a.
**Miridae**	*Agnocoris reclairei* (2)*	*Agnocoris rubicundus* (4)	0.15	AAZ9002
**Nabidae**	*Nabis pseudoferus* (10)	*Nabis brevis* (11)	0.15	AAZ3346
**Rhyparochromidae**	*Trapezonotus arenarius* (2)	*Trapezonotus dispar* (5)*	0.15	ABA2811
**Miridae**	*Adelphocoris quadripunctatus* (7)*	*Adelphocoris reichelii* (2)*	0.31	ABY7543
**Miridae**	*Phytocoris austriacus* (1)	*Phytocoris varipes* (2)	0.5	AAH9369
**Pentatomidae**	*Chlorochroa juniperina* (1)	*Chlorochroa pinicola* (2)*	0.93	ABV5200
**Rhyparochromidae**	*Megalonotus chiragra* (3)	*Megalonotus sabulicola* (1)	0.93	AAF4462
**Cymidae**	*Cymus aurescens* (6)*	*Cymus glandicolor* (4)*	1.26	AAY9365
**Miridae**	*Globiceps flavomaculatus* (3)*	*Globiceps fulvicollis* (9)	1.27	ABU6740
**Miridae**	*Phytocoris pini* (2)*	*Phytocoris tiliae* (5)	1.4	AAF5821
**Lygaeidae**	*Kleidocerys ericae* (1)	*Kleidocerys resedae* (14)*	1.71	AAY8761
	all other species	all other species	>2.2	

At least one specimen of both compared species showed a distance value below this threshold in terms of a pairwise comparison. Numbers in brackets indicate the number of analyzed specimens whereas asterisks mark monophyletic species/lineages.

**Table 2:** A table of eight species of the Heteroptera with a maximum pairwise distance (K2P) of >2.2% and one corresponding BIN.

Family	Species	Number of analyzed specimens (*n*) with (left) and without (right) BIN	Mean pairwise K2P distance (%)	Maximum pairwise K2P distance (%)	BINs
**Miridae**	Stenodema laevigata*	9/-	0.52	2.27	AAY9089
**Miridae**	*Globiceps fluvicollis*	5/4	1.39	2.31	ABU6740
**Anthocoridae**	Orius majusculus*	1/3	1.15	2.31	ABA5781
**Coreidae**	Coriomeris denticulatus*	8/1	1.28	2.34	ABU9164
**Nabidae**	Nabis limbatus*	11/1	0.54	2.58	ABU7333
**Nabidae**	Nabis ferus*	5/-	1.45	3.16	ABU9496
**Miridae**	Psallus ambiguus*	7/1	0.84	3.19	AAY8936
**Miridae**	*Phytocoris tiliae*	5/-	1.74	3.24	AAF5851

At least two specimens of the listed species showed a distance value higher than this threshold in terms of a pairwise comparison. Asterisks indicate monophyletic species/lineages.

**Table 3:** A table of 15 species of the Heteroptera with at least two corresponding BINs, mean and maximum pairwise K2P distances and an ABGD intraspecific divergence estimator (P) with a gap size factor of 0.1.

Family	Species	Number of analyzed specimens (*n*) with (left) and without BIN (right)	Mean pairwise K2P distance (%)	Maximum pairwise K2P distance (%)	BINs	Intraspecific divergence estimator P (%)
**Miridae**	*Adelphocoris lineolatus*	9/2	0.81	2.05	ACE7444, ACF1257	1.56
**Rhyparochromidae**	Raglius alboacuminatus*	3/-	1.47	2.2	ABW8820, ACA7459	<0.1
**Corixidae**	Sigara falleni*	4/-	1.45	2.24	AAH9524, ABY7152	2.06
**Blissidae**	Ischnodemus sabuleti*	11/-	0.75	2.55	ABY6046, AAT9271	2.06
**Pentatomidae**	Troilus luridus*	2/-	2.66	2.66	ABX8078, AAY9349	<0.1
**Miridae**	Polymerus unifasciatus*	7/-	1.27	3.97	AAY9312, AAZ3255	3.76
**Veliidae**	Microvelia reticulata*	4/-	2.15	3.98	AAG4340, AAG4341	3.76
**Rhopalidae**	Stictopleurus abutilon*	6/-	1.89	5.11	AAY9315, AAZ3130	4.75
**Miridae**	Atractotomus magnicornis*	8/-	2.33	5.46	ABV9583, AAE0766	4.98
**Miridae**	Plesiodema pinetella*	3/1	3.09	5.46	AAY8946, ABU8515	1.56
**Miridae**	Pilophorus clavatus*	5/-	4.21	7.15	ABA3473, ABA3474	7.92
**Anthocoridae**	Orius niger*	10/-	4.19	8.56	ABU8870, ABW5859	>10
**Miridae**	Stenodema calcarata*	12/-	3.03	8.63	AAY9091, ACI8060, AAZ3133	8.7
**Miridae**	*Phytocoris dimidiatus*	2/-	10.96	10.96	ABV5430, ABV8607	10
**Aradidae**	Aneurus avenius*	7/-	12.82	23.31	ABU9082, ABW2173	>10

Asterisks indicate monophyletic species/lineages.

Appendix S9 shows the results of the neighbour-joining cluster analysis based on K2P distances including bootstrap values. As part of this analysis we found non-monophyletic lineages for 26 species, including 15 species of the Miridae (57%), four species of the Nabidae (15%), two species of the Lygaeidae, Rhyparochromidae and Scutelleridae (3x 8%), and one species belonging to the Acathosomatidae (4%) (see Appendix S9).

### Species pairs with low nucleotide variability

Our data revealed a low nucleotide variability between 24 species pairs (39 species) with a minimum pairwise K2P distance <2.2% [87], including ten species pairs with a minimum pairwise K2P distance of zero (Table 1). Considering the used BIN parameter and given results, a molecular identification between specimens of the previous reported species pairs was not possible.

**Figure 2:**
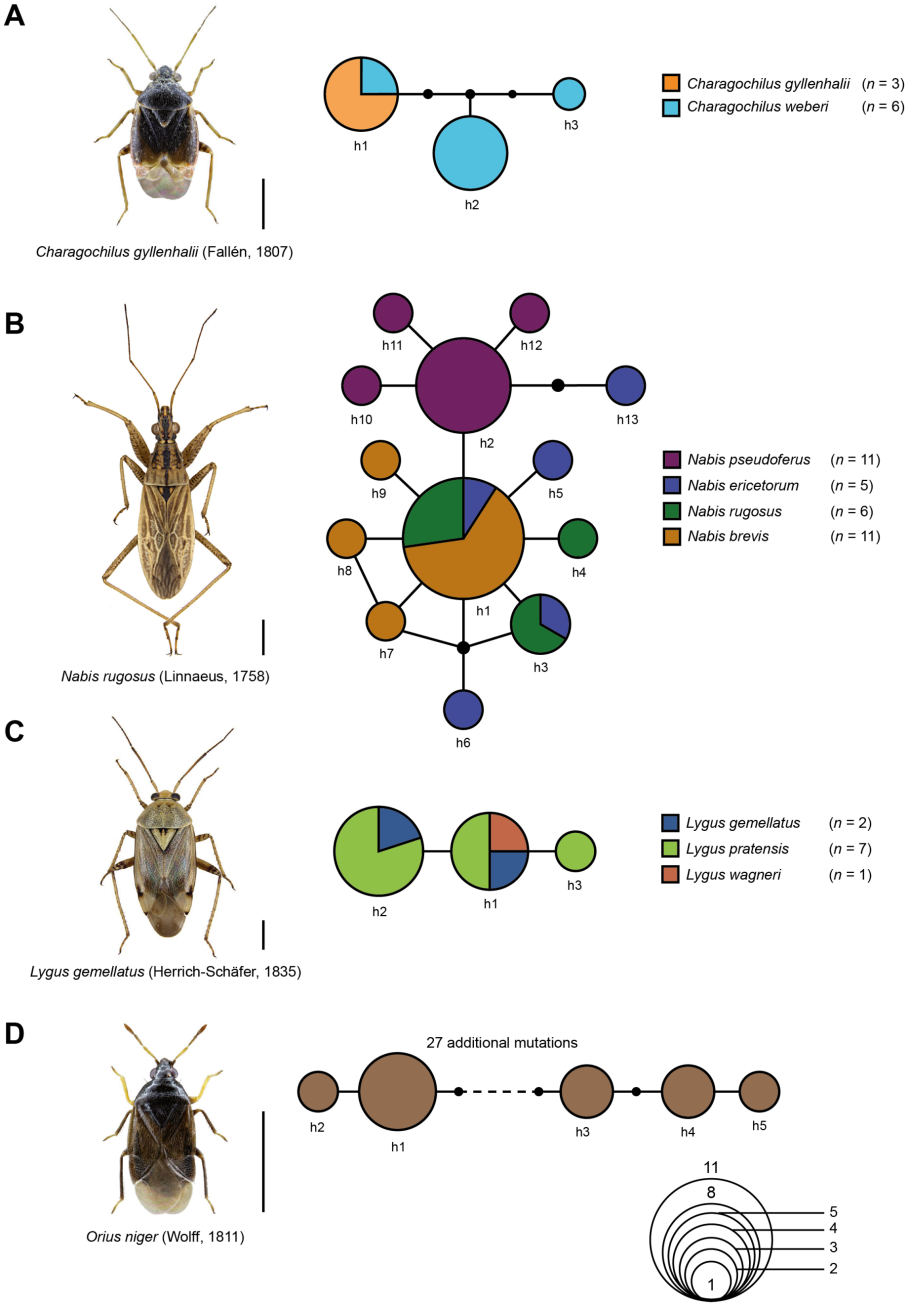
Statistical parsimony networks showing the mutational relationships among the analyzed mitochondrial CO1 haplotypes of A: *Charagochilus gyllenhalii* and *C. weberi*, B: *Nabis brevis, N. ericetorum*, *N. pseudoferus*, and *N. rugosus*, C: *Lygus gemellatus*, *L*. *pratensis*, and *L*. *wagneri*, and D: *Orius niger*. Each line in the network represents a single mutational change; small black dots indicate missing haplotypes. The numbers of analyzed specimens (*n*) are listed, while the diameter of the circles is proportional to the number of haplotypes sampled (see given Open circles with numbers). Scale bars  = 1 mm.

The statistical maximum parsimony analysis using the default setting of a 95% connection limit resolved for *Charagochilus gyllenhalii* and *C. weberi* (Fig. 2a) and the four analyzed species of the genus *Nabis* (Fig. 2b) revealed a multiple sharing of haplotypes. In the case of *Charagochilus* Fieber, 1858, *C. gyllenhalii* (number of analyzed specimens *n* = 3) and *C. weberi* (*n* = 6) all analyzed specimens of *C. weberi* were represented by one haplotype (h1) which was also shared by one specimen of *C. gyllenhalii*. Both other haplotypes found for *Charagochilus gyllenhalii* were separated from haplotype h1 by two (h2) and three (h3) additional mutational steps, respectively. For the four analyzed species of the genus *Nabis*, *N. ericetorum* Scholtz, 1847 (*n* = 5), *N. rugosus* (Linnaeus, 1758) (*n* = 6) and *N. brevis* Scholtz, 1847 (*n* = 11) shared identical DNA barcodes with one dominant haplotype (h1), whereas all analyzed specimens of *Nabis pseudoferus* Remane, 1949 (*n* = 11) were separated by at least one mutational step from this dominant haplotype. However, one haplotype of *Nabis ericetorum* (h13) was more closely related to *N. pseudoferus* than to the other tree *Nabis* species. In the case of the analyzed specimens of *Lygus* (Fig. 2c), one haplotype (h1) was shared by specimens of three species: *Lygus gemellatus* (Herich-Schäfer, 1835) (*n* = 1), *L. pratensis* (Linnaeus, 1758) (*n* = 2) and *L. wagneri* Remane 1955 (*n* = 1). Two other haplotypes (h2: 1x *Lygus gemellatus*, 4x *L. pratensis*; h3: 1x *L. pratensis*) were directly connected to this haplotype.

### High nucleotide variability within species

In contrast to the low nucleotide distances, our analyses revealed 24 species with distances >2.2%, including eight species with one corresponding BIN (Table 2) and 15 species with at least two BINs (Table 3) For one species (*Macrotylus paykullii* (Fallén, 1807); Miridae), no BINs were available due to a short fragment length of 407 bp for all three specimens. Values of the intraspecific divergence estimator P using the modified ABGD pipeline ranged from values <0.1 to >10%, correlating with the observed MPDs in most cases. Low values were found for the rhyparochromid species *Raglius alboacuminatus* (Goeze, 1778) (*n* = 3) (P<0.1%) and the mirid *Troilus luridus* (Fabricius, 1775) (*n* = 2) (<0.1%) as consequence of a limited number of analyzed specimens. The observed low value of *Plesiodema pinetella* (Zetterstedt, 1828) (Miridae) was most probably caused by the low number of analyzed specimens (*n* = 4) and the large distance to its nearest neighbor species (18.11%).

The statistical maximum parsimony analysis of *Orius niger* (*n* = 10) identified five haplotypes but two distinct and unconnected sub-networks (Fig. 2d). Lowering the connection limit to 90% revealed a putative connection between both sub-networks via 29 mutational steps.

## Discussion

Our sequence library represents an important step of analyzing the utility of DNA barcodes to discriminate true bug species, in particular for Central Europe. For many species, these barcode sequences represent the very first available molecular data. With a successful identification rate of 91.5% ( = 418 species), our data clearly demonstrate the ability of DNA barcoding to discriminate most species within this ecological and economical highly important taxon [20], [97] and coincide with high rates of successful species identification of previous barcoding studies of true bugs [63], [64].

Nevertheless, our data also highlight the need of further taxonomic revisions using both morphological and molecular methods in order to work up the classification of various species within different families. This is especially true for the Miridae, a taxon with more than 10,000 described species worldwide [2], [98], [99] and about 400 species known from Germany [100]. With 172 nominal species of 457 studied species (37.6%) and 702 analyzed specimens out of 1742 (40.3%), the Miridae also represent the most dominant taxon within our dataset. As with many hyper-diverse groups, the taxonomic impediment for the Miridae is significant, as evidenced low morphological variability in taxonomical important traits and numerous recent species discoveries [99], [101].

### Species pairs with low nucleotide variability

When species pairs have very recent origins or even still hybridize, the use of DNA barcodes for an efficient species identification finds its limit: after the initial “split”, new sister species will share alleles and mutations in slowly evolving genes [102]. Beside a given morphological and phenotypic plasticity, morphological distinctiveness may evolve much faster than the barcode fragment. Such situation has been already demonstrated for two Central European ground beetles, *Pterostichus nigrita* (Paykull, 1790) and *P. rhaeticus* Herr, 1837 [57].

For the analyzed Heteroptera we found pairwise K2P distances with values below 2.2% and haplotype sharing for 21 species pairs (39 species), indicating recent speciation events with on-going hybridization or recently evolved and distinct species. A case example of haplotype sharing caused by such effects was demonstrated for *Charagochilus gyllenhalii* and *C. weberi* with haplotype h1 found in both species (Fig. 2a). The species status of *Charagochilus weberi* is, however, discussed controversially among taxonomists. Whereas it is treated as valid and accepted species in most books on taxonomy [70] and ecology [103] as well as in databases [104], [105] and catalogues [106], the species status is still subject to discussion and doubted by some taxonomists (H. Simon, pers. com.).

Identical haplotypes and hybridization between even four species was given for the genus *Nabis* (Fig. 2b). There was no evidence for any differentiation among *Nabis ericetorum*, *N. rugosus* and *N. brevis*, whereas all analyzed specimens of *Nabis pseudoferus* were separated at least by one mutational step from this dominant haplotype. This missing molecular differentiation among *Nabis brevis*, *N. rugosus* and *N. pseudoferus* has been confirmed by partial 16S rDNA sequence data (S. Roth, pers. com.). All of the four *Nabis*-species belong to the subgenus *Nabis*, which includes two additional species in Central Europe, *Nabis ferus* and *N. punctatus*. The latter species was not part of our data-set, but *Nabis ferus* showed a clear distance to the four other *Nabis*-species (11.96% distance to *Nabis ericetorum*, see appendix S5 and S8). A clear differentiation of *Nabis ferus* from *N. brevis* was also shown for partial nuclear 18S rDNA sequences [107]. Interestingly, all species of the subgenus *Nabis* can be clearly distinguished by the shape of their genitalia [75], [108] and are assigned to two morphological “groups”: a) the “*Nabis-rugosus* L.-group” with the species *Nabis rugosus*, *N. brevis* and *N. ericetorum*, and b) the “*Nabis-ferus* (L.) sensu Ekblom-group” with the species *N. pseudoferus* [108]. Although their ecological requirements differ slightly, the ecological niche space overlaps, and thus two or more species can sometimes be observed in the same habitat. Hybridization among subspecies within the genus *Nabis* is a well-known phenomenon [108], [109], and interspecific copulations between *N. ferus* and *N. rugosus* as well as between *N. brevis* and *N. rugosus* and *N. ericetorum* have already been observed [110], [111]. In this context, our molecular data give strong evidence for on-going hybridization events between *Nabis pseudoferus*, *N. ericetorum*, *N. rugosus*, and *N. brevis*.

**Figure 3:**
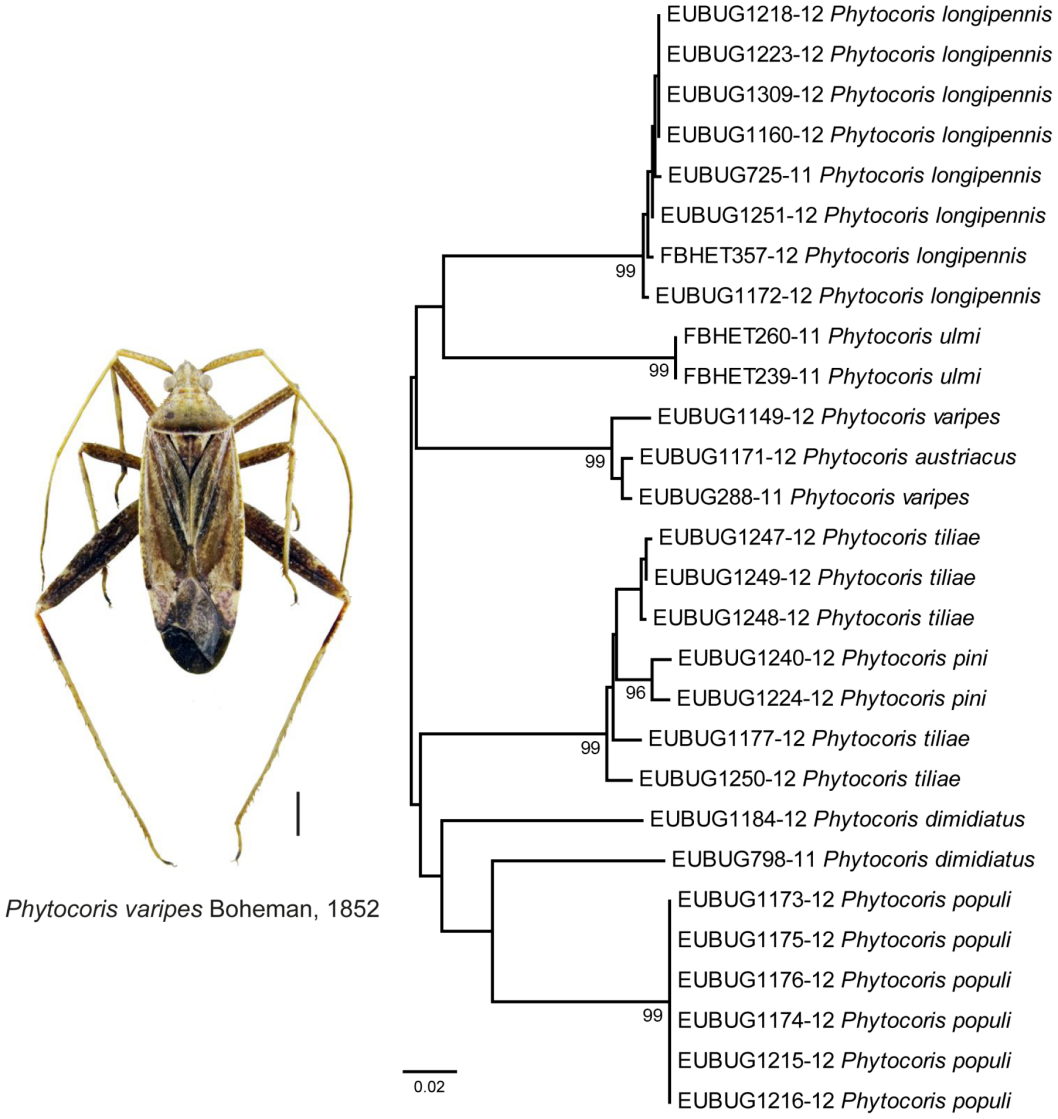
Subtree of the neighbour joining tree based on Kimura 2-parameter distances of all studied specimens of the genus *Phytocoris* (Miridae). Branches with specimen ID-number from BOLD and species name. Numbers next to internal branches are bootstrap values (in %). Scale bar  = 1 mm.

Low nucleotide distances were also found within the genus *Phytocoris* Fallén, 1814. With over 500 described species this genus represents the most speciose genera of the family Miridae [112]. Due to insufficient morphological diagnostic characters, an identification of many species is extremely difficult, in particular in females. Up to date, 15 different species are known from Germany [99]. As part of this study we analyzed eight species of *Phytocoris* (Fig. 3). Low pairwise K2P distances were found for two species pairs: *Phytocoris austriacus* Wagner, 1954 (*n* = 1) – *P. varipes* Boheman, 1852 (*n* = 2) with 0.5% and *Phytocoris pini* Kirschbaum, 1856 (*n* = 2) – *P. tiliae* (Fabricius, 1777) (*n* = 5) with 1.4%. Paraphyletic lineages were revealed for *Phytocoris varipes* and *P. tiliae*. Taxonomic problems within this genus were also revealed by a previous barcoding study [64], indicating the mandatory need of a comprehensive taxonomic revision of this genus.

Evidence for hybridization was also found within the notorious genus *Lygus*, the most important agricultural pests among the Miridae [23], [113], [114]. The existence of various morphologically similar species within this genus makes the identification of specimens in some cases quite difficult [115]. Whereas the analyzed eight specimens of *Lygus rugulipennis* Poppius, 1911 represent a well-defined distinct cluster (see Appendix S8), haplotype sharing was observed for *Lygus gemellatus* (*n* = 2), *L. pratensis* (*n* = 7), and *L. wagneri* (*n* = 1), preventing a successful species identification using DNA barcodes (Fig. 2c). However, the incapacity of DNA barcodes to discriminate various *Lygus* species has been already shown in a previous study [64].

In the case of other species pairs with non-monophyletic lineages as consequence of haplotype sharing we found evidence for on-going hybridization in *Elasmostethus interstinctus* (Linnaeus, 1758) (*n* = 3) and its sibling species *E. minor* Horváth, 1899 (*n* = 1) (Acanthosomatidae), *Arocatus longiceps* Stål, 1872 (*n* = 12) – *A. roeselii* (Schilling, 1829) (*n* = 2) (Lygaeidae), *Strongylocoris leucocephalus* (Linnaeus, 1758) (*n* = 5) – *S. steganoides* (J. Sahlberg, 1875) (*n* = 4) (Miridae), *Trigonotylus caelestialium* (Kirkaldy, 1902) (*n* = 10) – *T. pulchellus* (Hahn, 1834) (*n* = 2) (Miridae), and *Eurygaster maura* (Linnaeus, 1758) (*n* = 5) – *E. testudinaria* (Geoffroy, 1785) (*n* = 5) (Scutelleridae) (Table 1). Low nucleotide distances of monophyletic lineages/species associated with only one BIN were found for five species pairs: *Adelphocoris quadripunctatus* (Fabricius, 1794) (*n* = 7) – *A*. *reichelii* (Fieber, 1836) (*n* = 2) (Miridae) (0.31%), *Chlorochroa juniperina* (Linnaeus, 1758) (*n* = 1) – *C. pinicola* (Mulsant & Rey, 1852) (*n* = 2) (Pentatomidae) (0.93%), *Megalonotus chiragra* (Fabricius, 1794) (*n* = 3) – *M. sabulicola* (Thomson, 1870) (*n* = 1) (Rhyparochromidae) (0.93%), *Cymus aurescens* Distant, 1883 (*n* = 6) – *C. glandicolor* Hahn, 1832 (*n* = 5) (Cymidae) (1.26%), and *Kleidocerys ericae* (Horváth, 1908) (*n* = 1) – *K. resedae* (Panzer, 1797) (*n* = 14) (Lygaeidae) (1.71%) (Table 1). One paraphyletic and one monophyletic species lineage pooled by only one BIN was found for *Agnocoris reclairei* (Wagner, 1949) (*n* = 2) – *A. rubicundus* (Fallén, 1807) (*n* = 4) (Miridae) (0.15%), *Trapezonotus arenarius* (Linnaeus, 1758) (*n* = 2) – *T. dispar* Stål, 1872 (*n* = 5) (Rhyparochromidae) (0.15%), and *Globiceps flavomaculatus* (Fabricius, 1794) (*n* = 3) – *G. fulvicollis* Jakovlev, 1877 (*n* = 9) (Miridae) (1.27%). Low interspecific distances but distinct monophyletic lineages with two BINs were found for *Notonecta lutea* Müller, 1776 (*n* = 9) – *N. reuteri* Hungerford, 1928 (*n* = 4) (Notonectidae) with a minimum pairwise K2P distance of 1.24%, indicating a putative recent speciation event. Interestingly, both species can be identified based on significant differences in the coloration of the corium and scutellum, their genital morphology and their different habitat requirements, without doubt.

In all above mentioned cases, only the analysis of i) more specimens sampled from different localities, ii) other faster evolving markers, e.g. microsatellites or SNPs, iii) ecological parameters, and iv) comprehensive morphological and morphometric studies will give more insights into the taxonomic status of such a species complex.

### High nucleotide variability within species

Distinct lineages of mitochondrial DNA can be caused by various effects. These include population separation by phylogeographic events [116]–[119], incomplete lineage sorting [120]–[123], the presence of maternally inherited endosymbionts (e.g. *Wolbachia* [124], [125]), and simply the existence of cryptic species [26], [126], [127]. For the analyzed Heteroptera we found MPDs >2.2% and one corresponding BIN for eight species (Table 2). In contrast to this, 15 species were assigned to two or three BINs (only *Stenodema calcarata*), with MPDs ranging from 2.05% (*Adelphocoris lineolatus* (Goeze, 1778)) up to 23.31% (*Aneurus avenius* (Dufour, 1833)) (Tab. 3). High intraspecific values were also found for *Macrotylus paykullii* (Fallén, 1807) (Miridae) with a mean pairwise K2P distance of 4.34% and a MPD of 6.05% (Appendix S5). However, as consequence of the short length of the three analyzed sequences (407 bp), no BINs have been assigned to this sequences.

Based on the given data we are unable to clarify the reasons of the observed nucleotide distances and distinct lineages in most cases. Nevertheless, we strongly favor the presence of cryptic species as explanation when MPDs >5% were found. Certainly, additional studies have to be performed for clarification. However, these are normally not given for a barcode library which relies on a correct identification of the analyzed specimens. An exceptionally high nucleotide distance was found within *Aneurus avenius* (Aradidae), separating two monophyletic lineages with 23.31%. This is also supported by an intraspecific divergence estimator >10% (Table 3). Here, our morphological studies clearly exclude the presence of its sibling species in Central Europe, *Aneurus laevis* (Fabricius, 1775), as well as other closely related species (e.g. *Iralunelus gallicus* (Stys, 1974)) within the analyzed specimens. Thus, the probability of the presence of a cryptic species is high. In the case of the two genera *Stictopleurus* Stål, 1872 (Rhopalidae) and *Pilophorus* Hahn, 1826 (Miridae) our analysis comprised all species known from Central Europe. Consequently, the existence of cryptic species in these genera is also likely. Our molecular data also gave evidence for the putative existence of a cryptic species within *Orius niger* (Wolff, 1811) (Fig. 2c). This predatory species is widely common and can be discriminated clearly from other *Orius* species by various morphological characteristics, e.g. the black color except front legs and antennae for most specimens, single long hairs located on both the anterior and posterior angles of the pronotum, and their genital morphology [72]. Nevertheless, our data revealed two distinct lineages with a MPD of 8.56% and a P estimator of >10%. Interestingly, a recent study using the nuclear internal transcribed spacer-1 (ITS-1) for species identification found no evidence of distinct lineages or the existence of a cryptic species [128]. In other morphologically distinct species the probability of cryptic species is assumed to be high, such as in *Stenodema calcarata* (Fallén, 1807) (Miridae). This species belongs to the subgenus *Brachystira* which is morphologically clearly separated not only from the subgenus *Stenodema* [66], [129], but also from its sibling species in Central Europe, *Stenodema trispinosa* Reuter, 1904 by the number and form of the spines on their metafemora [67]. This is also true for *Stenodema pilosa* (Jakovlev, 1889) which is found in Central Asia. A taxonomically difficult taxon is the genus *Atractotomus* Fieber, 1858 [130], with six known species from Central Europe [131]. However, the morphological identification of the analyzed specimens of *Atractotomus magnicornis* (Fallén, 1807) was clear without ambiguity. In the case of *Plesiodema pinetella* (Miridae), no other species of this genus are known for Central Europe [3]. Whereas this species can be easily confused with another species of the Miridae, to be specific *Phoenicocoris obscurellus* (Fallén, 1829), DNA barcoding allowed an identification of both species free of doubts. Nevertheless, the observed high nucleotide variability within *Plesiodema pinetella* is somewhat surprising. Of course, for all these species more individuals from different localities have to be checked to clarify their taxonomic status using both morphological and molecular, in particular nuclear, data.

## Conclusion

Our study represents the first step in building-up a comprehensive DNA barcode library for true bugs in Central Europe. Furthermore, our data clearly demonstrate the usefulness of DNA barcoding for heteropteran species identification for most of the analyzed species. In spite of the fact that taxonomic research of the Heteroptera in Germany has a long history and tradition of more than 200 years, the species status of various taxa is still subject to discussion. This regards not only to the specious and taxonomically difficult Miridae, but surprisingly also much more comprehensively processed species among the Pentatomomorpha. Our study revealed several gaps between morphology and barcoding-based groupings across Heteroptera species cluster. Our DNA barcode library thus opens up a way towards a desired more intensive collaboration between morphological and molecular Heteroptera taxonomists. This will be crucial for resolving the still existing uncertainties in Heteroptera taxonomy.

## Supporting Information

Appendix S1 **Number of analyzed specimens of true bugs sampled in Germany per Bundesland.**
(DOCX)

Appendix S2 **Sample IDs, accession numbers, species names and sample localities of all analyzed specimens.** Non-German specimens are highlighted in light gray.
(PDF)

Appendix S3 **Supplementary information about the used PERL-Script APE and modified ABGD pipeline.** Species of the Heteroptera with at least two corresponding BINs (see Table 3) are marked in bold.
(DOCX)

Appendix S4 **Frequency histogram of barcode sequence length including all 1742 analyzed sequences.** A total of 1207 (69%) sequences showed full sequence length of 658 base pairs and a total of 1231 (71%) a sequence length of at least 650 base pairs.
(DOCX)

Appendix S5 **Box-Whisker-Plot (Median, 25% and 75% percentiles, Min-Max values excluding outliers) of average barcode sequence length per Heteroptera family.** The numbers right of the graph indicate the number of sequences per family. A total of 1742 sequences were analysed.
(DOCX)

Appendix S6 **Barcode distances of the analyzed Heteroptera.** Divergence values were calculated for all sequences >400 base pairs, using the Nearest Neighbour Summary as part of the Barcode Gap Analysis tool provided in the Barcode of Life Data System (BOLD). Used distance model: Kimura 2-paramter, align sequencing option: BOLD aligner (amino acid based HMM), ambiguous base/gap handling: pairwise deletion. ISD  =  intra-specific distance.
(DOCX)

Appendix S7 **The distribution of sequence divergence within species and within genera based on the given classification.** All tables and histograms were calculated using the BOLD V3.0 working bench.
(DOCX)

Appendix S8 **Matrix plots of the ABGD results** The image plots show species names on the y- and the intraspecific divergence prior on the x-axis. For each plot, the relative gap width X is constant (minimum and maximum X values are shown). The image plots were produced automatically by discriminating different cases. Possible cases are i) all sequences of one species are found in one single group (red), or ii) sequences that belong to one species are found in at least two groups (blue).
(PDF)

Appendix S9 **Complete Neighbour joining topology of all analyzed specimens of the Heteroptera based on Kimura 2-parameter distances.** Branches with specimen ID-number from BOLD and species name. Numbers next to internal branches are bootstrap values (1000 replicates, in %).
(PDF)

Appendix S10 **Table of all non-monophyletic species/lineages of the analyzed data set.**
(DOCX)
