# Supplementary material for: Building-Up of a DNA Barcode Library for True Bugs (Insecta: Hemiptera: Heteroptera) of Germany Reveals Taxonomic Uncertainties and Surprises
Source: PLoS One. 2014 Sep 9;9(9):e106940. doi: 10.1371/journal.pone.0106940 (PMC4159288; doi:10.1371/journal.pone.0106940)
Supplement: Appendix S1 — Number of analyzed specimens of true bugs sampled in Germany per Bundesland. (DOCX) [file pone.0106940.s001.docx]

| **Land** | **No. of analyzed specimens** | **%** |
| --- | --- | --- |
| Bavaria | 1035 | 61.61 |
| Baden-Wuerttemberg | 212 | 12.62 |
| Thuringia | 159 | 9.46 |
| Rhineland Palatinate | 118 | 7.02 |
| Brandenburg | 106 | 6.31 |
| North Rhine-Westphalia | 24 | 1.43 |
| Lower Saxony | 12 | 0.71 |
| Mecklenburg-Vorpommern | 11 | 0.66 |
| Hesse | 3 | 0.18 |
