# Supplementary material for: Building-Up of a DNA Barcode Library for True Bugs (Insecta: Hemiptera: Heteroptera) of Germany Reveals Taxonomic Uncertainties and Surprises
Source: PLoS One. 2014 Sep 9;9(9):e106940. doi: 10.1371/journal.pone.0106940 (PMC4159288; doi:10.1371/journal.pone.0106940)
Supplement: Appendix S2 — Sample IDs, accession numbers, species names and sample localities of all analyzed specimens. Non-German specimens are highlighted in light gray. (PDF) [file pone.0106940.s002.pdf]

Sample ID	Accession no.	Family	Species	State/Province	Sector	Lat	Lon
BFB_Heteroptera_Kuechler_0284	**KM022167**	Acanthosomatidae	***Acanthosoma haemorrhoidale***	Bavaria	Bayreuth, University	49,9290	11,5830
EUBUG_52_f_Acanhaem1	**KM023091**	Acanthosomatidae	***Acanthosoma haemorrhoidale***	Bavaria	Neuburg an der Donau	48,7320	11,2840
EUBUG_53_f_Acanhaem2	**KM021565**	Acanthosomatidae	***Acanthosoma haemorrhoidale***	Bavaria	Neuburg an der Donau	48,7350	11,2810
EUBUG_54_f_Acanhaem3	**KM021981**	Acanthosomatidae	***Acanthosoma haemorrhoidale***	Bavaria	Neuburg an der Donau	48,7400	11,2790
EUBUG_56_m_Acanhaem6	**KM022893**	Acanthosomatidae	***Acanthosoma haemorrhoidale***	Bavaria	Neuburg an der Donau	48,7480	11,2830
BFB_Heteroptera_Kuechler_0101	**KM022594**	Acanthosomatidae	***Cyphostethus tristriatus***	Bavaria	Bayreuth, Bindlacher Berg	50,0070	11,6140
BFB_Heteroptera_Kuechler_0213	**KM022839**	Acanthosomatidae	***Elasmostethus interstinctus***	Bavaria	Bayreuth, Kraimoos Weiher	49,8200	11,5930
EUBUG_1225_f_Elasinte5	**KM021522**	Acanthosomatidae	***Elasmostethus interstinctus***	Bavaria	Bavarian Forest, Rainer Wald	48,9150	12,4550
EUBUG_320_m_Elasinte1	**KM022220**	Acanthosomatidae	***Elasmostethus interstinctus***	Bavaria	Neuburg an der Donau	48,7370	11,2930
EUBUG_1331_m_Elasmino3	**KM022153**	Acanthosomatidae	***Elasmostethus minor***	Thuringia	Hainich-Duen	51,3370	10,3650
BFB_Heteroptera_Kuechler_0228	**KM021862**	Acanthosomatidae	***Elasmucha ferrugata***	Bavaria	Bayreuth, Botanic Garden	49,9230	11,5850
BFB_Heteroptera_Kuechler_0229	**KM021857**	Acanthosomatidae	***Elasmucha ferrugata***	Bavaria	Bayreuth, Botanic Garden	49,9230	11,5850
EUBUG_1161_m_Elasferr1	**KM022122**	Acanthosomatidae	***Elasmucha ferrugata***	Bavaria	Bavarian Forest, Riedelhuette	48,9210	13,4120
EUBUG_1232_m_Elasferr2	**KM022186**	Acanthosomatidae	***Elasmucha ferrugata***	Bavaria	Bavarian Forest, Saldenburg	48,7910	13,3510
BFB_Heteroptera_Kuechler_0179	**KM022451**	Acanthosomatidae	***Elasmucha grisea***	Bavaria	Bayreuth, Botanic Garden	49,9230	11,5870
EUBUG_1050_m_Elasgris3	**KM021603**	Acanthosomatidae	***Elasmucha grisea***	Thuringia	Hainich-Duen	51,2050	10,3900
EUBUG_728_f_Elasgris1	**KM022828**	Acanthosomatidae	***Elasmucha grisea***	Rhineland Palatinate	Fischbach bei Dahn	49,0860	7,7230
BC_ZSM_HETA_0912	**KM022052**	Alydidae	***Alydus calcaratus***	Bavaria	Siegenburg, Dassfeld	48,7589	11,8383
BC_ZSM_HETA_0913	**KM022706**	Alydidae	***Alydus calcaratus***	Bavaria	Munich, Rangierbahnhof	48,1906	11,5269
BC_ZSM_HETA_0914	**KM022710**	Alydidae	***Alydus calcaratus***	Bavaria	Erlangen, Exerzierplatz	49,5861	11,0308
BC_ZSM_HETA_0915	**KM022472**	Alydidae	***Alydus calcaratus***	Bavaria	Roth, Hofstetten	49,1669	11,1783
BFB_Heteroptera_Kuechler_0226	**KM021546**	Alydidae	***Alydus calcaratus***	Bavaria	Bayreuth, Mistelbach	49,9310	11,5370
BFB_Heteroptera_Kuechler_0280	**KM022069**	Alydidae	***Alydus calcaratus***	Bavaria	Kronach, Rennesberg	50,2570	11,3700
BFB_Heteroptera_Schmolke_0514	**KM022978**	Alydidae	***Alydus calcaratus***	Brandenburg	Mallnow Oderhaenge	52,4701	14,4781
EUBUG_424_juv_Alydcalc1	**KM022983**	Alydidae	***Alydus calcaratus***	Thuringia	Hainich-Duen	51,2070	10,7540
EUBUG_730_m_Alydcalc2	**KM023040**	Alydidae	***Alydus calcaratus***	Rhineland Palatinate	Fischbach bei Dahn	49,1040	7,6830
EUBUG_731_m_Alydcalc3	**KM022192**	Alydidae	***Alydus calcaratus***	Rhineland Palatinate	Fischbach bei Dahn	49,1040	7,6830
EUBUG_867_f_Alydcalc4	**KM022840**	Alydidae	***Alydus calcaratus***	Rhineland Palatinate	Fischbach bei Dahn	49,0900	7,7150
EUBUG_868_m_Alydcalc5	**KM021666**	Alydidae	***Alydus calcaratus***	Rhineland Palatinate	Fischbach bei Dahn	49,0900	7,7150
BFB_Heteroptera_Schmolke_0507	**KM021497**	Anthocoridae	***Anthocoris amplicollis***	Bavaria	Munich, Allacher Heide	48,2008	11,4828
BFB_Heteroptera_Schmolke_0509	**KM023052**	Anthocoridae	***Anthocoris amplicollis***	Bavaria	Munich, Truderinger Wald	48,0933	11,6683
EUBUG_1199_m_Anthconf5	**KM023008**	Anthocoridae	***Anthocoris amplicollis***	Bavaria	Bavarian Forest, Rainer Wald	48,9110	12,4510

EUBUG_62_f_Anthampl2	**KM022807**	Anthocoridae	***Anthocoris amplicollis***	Bavaria	Neuburg an der Donau	48,7390	11,3010
EUBUG_63_m_Anthampl3	**KM021512**	Anthocoridae	***Anthocoris amplicollis***	Bavaria	Neuburg an der Donau	48,7420	11,2800
BFB_Heteroptera_Kuechler_0312	**KM022525**	Anthocoridae	***Anthocoris confusus***	Bavaria	Bayreuth, Botanic Garden	49,9230	11,5850
BFB_Heteroptera_Schmolke_0574	**KM022453**	Anthocoridae	***Anthocoris minki***	Bavaria	Munich, Korbinianihoelzl	48,2269	11,5444
BFB_Heteroptera_Kuechler_0074	**KM021933**	Anthocoridae	***Anthocoris nemoralis***	Bavaria	Bayreuth, Botanic Garden	49,9230	11,5830
BFB_Heteroptera_Kuechler_0096	**KM021825**	Anthocoridae	***Anthocoris nemoralis***	Bavaria	Bayreuth	49,9260	11,5620
BFB_Heteroptera_Schmolke_0334	**KM022697**	Anthocoridae	***Anthocoris nemoralis***	Bavaria	Pollanten	49,1511	11,4433
EUBUG_1332_f_Anthnemo8	**KM021552**	Anthocoridae	***Anthocoris nemoralis***	Thuringia	Hainich-Duen	51,3340	10,3680
BFB_Heteroptera_Kuechler_0064	**KM021780**	Anthocoridae	***Anthocoris nemorum***	Bavaria	Bayreuth	49,9290	11,5830
BFB_Heteroptera_Kuechler_0231	**KM022240**	Anthocoridae	***Anthocoris nemorum***	Bavaria	Bayreuth, Botanic Garden	49,9230	11,5850
BFB_Heteroptera_Schmolke_0335	**KM023079**	Anthocoridae	***Anthocoris nemorum***	Bavaria	Siegenburg	48,7589	11,8383
EUBUG_348_f_Anthneum1	**KM022208**	Anthocoridae	***Anthocoris nemorum***	Baden-Wuerttemberg	Fronreute	47,8476	9,6100
EUBUG_513_f_Anthneum2	**KM023096**	Anthocoridae	***Anthocoris nemorum***	Lower Saxony	Rastede	53,2430	8,2040
EUBUG_705_f_Anthneum3	**KM021761**	Anthocoridae	***Anthocoris nemorum***	Baden-Wuerttemberg	Pfrunger Ried	47,8990	9,3900
EUBUG_706_f_Anthneum4	**KM022515**	Anthocoridae	***Anthocoris nemorum***	Baden-Wuerttemberg	Pfrunger Ried	47,8990	9,3900
EUBUG_707_m_Anthneum5	**KM021581**	Anthocoridae	***Anthocoris nemorum***	Baden-Wuerttemberg	Pfrunger Ried	47,8990	9,3900
EUBUG_708_m_Anthneum6	**KM022431**	Anthocoridae	***Anthocoris nemorum***	Baden-Wuerttemberg	Pfrunger Ried	47,8990	9,3900
BFB_Heteroptera_Schmolke_0582	**KM022075**	Anthocoridae	***Anthocoris sarothamni***	Bavaria	Siegenburg, Dassfeld	48,7589	11,8383
BFB_Heteroptera_Schmolke_0584	**KM021692**	Anthocoridae	***Anthocoris sarothamni***	Bavaria	Pleinfeld, Seemannsmuehle	49,1217	11,0003
EUBUG_589_f_Oriulati1	**KM022338**	Anthocoridae	***Orius laticollis***	Baden-Wuerttemberg	Pfrunger Ried	47,8990	9,3900
EUBUG_591_f_Oriuminu8	**KM021961**	Anthocoridae	***Orius laticollis***	Baden-Wuerttemberg	Pfrunger Ried	47,8990	9,3900
BFB_Heteroptera_Kuechler_0047	**KM022868**	Anthocoridae	***Orius majusculus***	Bavaria	Bayreuth	49,9260	11,5620
EUBUG_626_f_Oriumaju1	**KM022033**	Anthocoridae	***Orius majusculus***	Baden-Wuerttemberg	Schenkenwald	47,8490	9,6220
EUBUG_645_f_Temngrac7	**KM022416**	Anthocoridae	***Orius majusculus***	Brandenburg	Kuestrin	52,5720	14,6270
EUBUG_980_m_Oriumaju2	**KM021573**	Anthocoridae	***Orius majusculus***	Baden-Wuerttemberg	Swabian Alb	48,4130	9,5420
BFB_Heteroptera_Schmolke_0602	**KM021917**	Anthocoridae	***Orius minutus***	Bavaria	Munich, Froettmaninger Heide	48,2114	11,6083
EUBUG_457_m_Oriuminu1	**KM022012**	Anthocoridae	***Orius minutus***	Brandenburg	Schorfheide-Chorin	53,1070	14,0000
EUBUG_460_m_Oriunige3	**KM021827**	Anthocoridae	***Orius minutus***	Brandenburg	Schorfheide-Chorin	53,1070	14,0000
EUBUG_577_f_Oriuminu5	**KM023084**	Anthocoridae	***Orius minutus***	Baden-Wuerttemberg	Pfrunger Ried	47,8990	9,3900
EUBUG_578_f_Oriuminu6	**KM021959**	Anthocoridae	***Orius minutus***	Baden-Wuerttemberg	Pfrunger Ried	47,8990	9,3900
EUBUG_590_f_Oriuminu7	**KM023012**	Anthocoridae	***Orius minutus***	Baden-Wuerttemberg	Pfrunger Ried	47,8990	9,3900
EUBUG_592_f_Oriuminu9	**KM023117**	Anthocoridae	***Orius minutus***	Baden-Wuerttemberg	Pfrunger Ried	47,8990	9,3900
EUBUG_593_f_Oriuminu10	**KM023104**	Anthocoridae	***Orius minutus***	Baden-Wuerttemberg	Pfrunger Ried	47,8990	9,3900

EUBUG_954_m_Oriuminu11	**KM021813**	Anthocoridae	***Orius minutus***	Thuringia	Jena	50,9510	11,6240
EUBUG_959_m_Oriuminu12	**KM022802**	Anthocoridae	***Orius minutus***	Thuringia	Jena	50,9510	11,6240
EUBUG_967_m_Oriuminu13	**KM021966**	Anthocoridae	***Orius minutus***	Rhineland Palatinate	Fischbach bei Dahn	49,0900	7,7150
EUBUG_968_m_Oriuminu14	**KM022224**	Anthocoridae	***Orius minutus***	Rhineland Palatinate	Fischbach bei Dahn	49,0900	7,7150
EUBUG_969_m_Oriuminu15	**KM023083**	Anthocoridae	***Orius minutus***	Rhineland Palatinate	Fischbach bei Dahn	49,0900	7,7150
EUBUG_970_f_Oriuminu16	**KM022677**	Anthocoridae	***Orius minutus***	Rhineland Palatinate	Fischbach bei Dahn	49,0900	7,7150
EUBUG_971_f_Oriuminu17	**KM022748**	Anthocoridae	***Orius minutus***	Rhineland Palatinate	Fischbach bei Dahn	49,0900	7,7150
EUBUG_981_m_Oriuminu18	**KM022730**	Anthocoridae	***Orius minutus***	Baden-Wuerttemberg	Swabian Alb	48,3830	9,5210
BFB_Heteroptera_Schmolke_0606	**KM022999**	Anthocoridae	***Orius niger***	Bavaria	Munich, Oberschleissheim	48,2269	11,5444
BFB_Heteroptera_Schmolke_0608	**KM022882**	Anthocoridae	***Orius niger***	Bavaria	Reichelsdorf, Koenigshof	49,4235	11,0825
EUBUG_459_m_Oriunige2	**KM022414**	Anthocoridae	***Orius niger***	Brandenburg	Schorfheide-Chorin	53,1070	14,0000
EUBUG_605_f_Oriunige4	**KM023138**	Anthocoridae	***Orius niger***	Rhineland Palatinate	Fischbach bei Dahn	49,1040	7,6830
EUBUG_606_m_Oriunige5	**KM022126**	Anthocoridae	***Orius niger***	Rhineland Palatinate	Fischbach bei Dahn	49,1040	7,6830
EUBUG_607_m_Oriunige6	**KM021890**	Anthocoridae	***Orius niger***	Rhineland Palatinate	Fischbach bei Dahn	49,1040	7,6830
EUBUG_608_m_Oriunige7	**KM021745**	Anthocoridae	***Orius niger***	Rhineland Palatinate	Fischbach bei Dahn	49,1040	7,6830
EUBUG_609_m_Oriunige8	**KM021482**	Anthocoridae	***Orius niger***	Rhineland Palatinate	Fischbach bei Dahn	49,1040	7,6830
EUBUG_952_f_Oriunige9	**KM022760**	Anthocoridae	***Orius niger***	Thuringia	Jena	50,9510	11,6240
EUBUG_965_m_Oriunige10	**KM022197**	Anthocoridae	***Orius niger***	Thuringia	Jena	50,9510	11,6240
BFB_Heteroptera_Kuechler_0364	**KM022138**	Anthocoridae	***Temnostethus gracilis***	Bavaria	Bayreuth, Schlehenmuehle	49,9050	11,6240
BFB_Heteroptera_Schmolke_0587	**KM022614**	Anthocoridae	***Temnostethus gracilis***	Bavaria	Munich, Allacher Heide	48,2008	11,4828
BFB_Heteroptera_Schmolke_0588	**KM022403**	Anthocoridae	***Temnostethus gracilis***	Bavaria	Erlangen	49,5947	10,9836
EUBUG_1039_m_Temngrac12	**KM023076**	Anthocoridae	***Temnostethus gracilis***	Bavaria	Bavarian Forest, Jochenstein	48,5170	13,7250
EUBUG_1040_f_Temngrac13	**KM022622**	Anthocoridae	***Temnostethus gracilis***	Bavaria	Bavarian Forest, Jochenstein	48,5170	13,7250
EUBUG_571_f_Temngrac7	**KM022732**	Anthocoridae	***Temnostethus gracilis***	Bavaria	Bavarian Forest, Solla	48,8200	13,3050
EUBUG_588_f_Temngrac7	**KM021948**	Anthocoridae	***Temnostethus gracilis***	Baden-Wuerttemberg	Pfrunger Ried	47,8990	9,3900
EUBUG_1014_f_Temnpusi10	**KM022023**	Anthocoridae	***Temnostethus pusillus***	Bavaria	Bavarian Forest, Jochenstein	48,5190	13,7280
BFB_Heteroptera_Kuechler_0306	**KM022346**	Anthocoridae	***Xylocoris cursitans***	Rhineland Palatinate	Fischbach bei Dahn	49,0910	7,7150
BFB_Heteroptera_Kuechler_0313	**KM022293**	Anthocoridae	***Xylocoris cursitans***	Rhineland Palatinate	Fischbach bei Dahn	49,1290	7,6900
EUBUG_624_f_Xylocurs3	**KM022560**	Anthocoridae	***Xylocoris cursitans***	Baden-Wuerttemberg	Schenkenwald	47,8490	9,6220
EUBUG_625_f_Xylocurs4	**KM021507**	Anthocoridae	***Xylocoris cursitans***	Baden-Wuerttemberg	Schenkenwald	47,8490	9,6220
EUBUG_768_f_Xylogala1	**KM022005**	Anthocoridae	***Xylocoris galactinus***	Thuringia	Hainich-Duen	47,8990	9,3900
BFB_Heteroptera_Schmolke_0625	**KM022852**	Aradidae	***Aneurus avenius***	Bavaria	Lenggries am Sylvensteinsee	47,5950	11,5536
BFB_Heteroptera_Schmolke_0626	**KM023094**	Aradidae	***Aneurus avenius***	Bavaria	Kallmuenz, Mailerberg	49,1842	11,9450

BFB_Heteroptera_Schmolke_0628	**KM022827**	Aradidae	***Aneurus avenius***	Bavaria	Schoengeising	48,1483	11,1958
EUBUG_1157_f_Aneuaven6	**KM022676**	Aradidae	***Aneurus avenius***	Bavaria	Bavarian Forest, Jochenstein	48,5190	13,7280
EUBUG_915_f_Aneuaven1	**KM022668**	Aradidae	***Aneurus avenius***	France	Le Trois Becs	44,6450	5,2140
EUBUG_916_m_Aneuaven2	**KM021501**	Aradidae	***Aneurus avenius***	France	Le Trois Becs	44,6450	5,2140
EUBUG_966_juv_Aneuaven3	**KM022921**	Aradidae	***Aneurus avenius***	France	Le Trois Becs	44,6450	5,2140
BFB_Heteroptera_Schmolke_0629	**KM021643**	Aradidae	***Aradus betulae***	Bavaria	Munich, Angerlohe	48,1914	11,4847
EUBUG_1412_f_Aradbetu2	**KM022968**	Aradidae	***Aradus betulae***	Bavaria	Spessart	49,9210	9,4300
EUBUG_1413_m_Aradbetu3	**KM022074**	Aradidae	***Aradus betulae***	Bavaria	Spessart	49,9310	9,3950
EUBUG_831_f_Aradbetu1	**KM022042**	Aradidae	***Aradus betulae***	Bavaria	Spessart	49,1040	7,6830
BFB_Heteroptera_Schmolke_0634	**KM022651**	Aradidae	***Aradus cinnamomeus***	Bavaria	Munich, Froettmaninger Heide	48,2114	11,6083
EUBUG_669_f_Aradcinn1	**KM022443**	Aradidae	***Aradus cinnamomeus***	Bavaria	Germering	48,1130	11,3880
EUBUG_670_f_Aradcinn2	**KM021532**	Aradidae	***Aradus cinnamomeus***	Bavaria	Germering	48,1130	11,3880
EUBUG_671_f_Aradcinn3	**KM022864**	Aradidae	***Aradus cinnamomeus***	Bavaria	Germering	48,1130	11,3880
EUBUG_1410_f_Aradcons1	**KM022916**	Aradidae	***Aradus conspicuus***	Bavaria	Spessart	49,8600	9,3800
EUBUG_1414_f_Aradcons3	**KM021491**	Aradidae	***Aradus conspicuus***	Bavaria	Steigerwald	49,9280	10,6120
EUBUG_1415_m_Aradcons4	**KM021935**	Aradidae	***Aradus conspicuus***	Bavaria	Steigerwald	49,9190	10,5680
EUBUG_117_f_Araddepr2	**KM022626**	Aradidae	***Aradus depressus***	Thuringia	Hainich-Duen	51,2680	10,2390
EUBUG_118_f_Araddepr3	**KM022610**	Aradidae	***Aradus depressus***	Thuringia	Hainich-Duen	51,2680	10,2390
EUBUG_1229_m_Araddepr7	**KM023004**	Aradidae	***Aradus depressus***	Bavaria	Bavarian Forest, Isar estuary	48,7780	12,9980
EUBUG_854_m_Mezitrem1	**KM021910**	Aradidae	***Mezira tremulae***	Hesse	Gross-Gerau	49,9470	8,5080
EUBUG_855_m_Mezitrem2	**KM021470**	Aradidae	***Mezira tremulae***	Hesse	Gross-Gerau	49,9470	8,5080
EUBUG_856_m_Mezitrem3	**KM022506**	Aradidae	***Mezira tremulae***	Hesse	Gross-Gerau	49,9470	8,5080
BFB_Heteroptera_Kuechler_0077	**KM022144**	Artheneidae	***Chilacis typhae***	Bavaria	Neunkirchen am Main	49,9240	11,6320
BFB_Heteroptera_Kuechler_0078	**KM022575**	Artheneidae	***Chilacis typhae***	Bavaria	Neunkirchen am Main	49,9240	11,6320
BFB_Heteroptera_Schmolke_0699	**KM022583**	Artheneidae	***Chilacis typhae***	Bavaria	Munich, Rangierbahnhof	48,1906	11,5269
BFB_Heteroptera_Schmolke_0700	**KM021524**	Artheneidae	***Chilacis typhae***	Bavaria	Pleinfeld, Mandlesmuehle	49,1267	10,9778
BFB_Heteroptera_Schmolke_0701	**KM021647**	Artheneidae	***Chilacis typhae***	Bavaria	Spalt, Huegelmuehle	49,1731	10,9847
BFB_Heteroptera_Schmolke_0702	**KM021769**	Artheneidae	***Chilacis typhae***	Bavaria	Munich, northern Angerlohe	48,1914	11,4847
EUBUG_917_m_Chiltyph1	**KM022055**	Artheneidae	***Chilacis typhae***	Thuringia	Jena	50,9510	11,6240
BC_ZSM_HETA_0894	**KM021848**	Berytidae	***Berytinus clavipes***	Bavaria	Oberrimbach, Einsiedeln	49,8274	10,5524
BC_ZSM_HETA_0895	**KM021558**	Berytidae	***Berytinus clavipes***	Bavaria	Kallmuenz, Mailerberg	49,1842	11,9450
BFB_Heteroptera_Kuechler_0140	**KM022503**	Berytidae	***Berytinus clavipes***	Bavaria	Bayreuth, Hohenmirsberger-Platte	49,8150	11,4450
BFB_Heteroptera_Kuechler_0141	**KM021571**	Berytidae	***Berytinus clavipes***	Bavaria	Bayreuth, Hohenmirsberger-Platte	49,8150	11,4450

EUBUG_280_f_Beryclavi2	**KM022858**	Berytidae	***Berytinus clavipes***	Thuringia	Hainich-Duen	51,2240	10,3810
EUBUG_281_m_Beryclavi3	**KM022081**	Berytidae	***Berytinus clavipes***	Thuringia	Hainich-Duen	51,2240	10,3810
BC_ZSM_HETA_0899	**KM022490**	Berytidae	***Berytinus crassipes***	Bavaria	Nuernberg, Reichelsdorf	49,3861	11,0303
BC_ZSM_HETA_0900	**KM021937**	Berytidae	***Berytinus crassipes***	Bavaria	Siegenburg, Dassfeld	48,7589	11,8383
EUBUG_264_m_Berycras1	**KM022045**	Berytidae	***Berytinus crassipes***	Thuringia	Hainich-Duen	51,2810	10,4560
EUBUG_265_m_Berycras2	**KM023113**	Berytidae	***Berytinus crassipes***	Thuringia	Hainich-Duen	51,2810	10,4560
EUBUG_266_m_Berycras3	**KM021664**	Berytidae	***Berytinus crassipes***	Thuringia	Hainich-Duen	51,2810	10,4560
BC_ZSM_HETA_0896	**KM021656**	Berytidae	***Berytinus minor***	Bavaria	Munich, Rangierbahnhof	48,1906	11,5269
BC_ZSM_HETA_0897	**KM023030**	Berytidae	***Berytinus minor***	Bavaria	Munich, northern Angerlohe	48,1914	11,4847
BC_ZSM_HETA_0898	**KM021681**	Berytidae	***Berytinus minor***	Bavaria	Munich, Truderinger Wald	48,0933	11,6683
BFB_Heteroptera_Schmolke_0348	**KM021448**	Berytidae	***Berytinus minor***	Bavaria	Pollanten	49,1511	11,4433
EUBUG_1209_m_Berymino4	**KM022833**	Berytidae	***Berytinus minor***	Bavaria	Bavarian Forest, Rainer Wald	48,9150	12,4430
EUBUG_294_m_Berymino1	**KM021660**	Berytidae	***Berytinus minor***	Thuringia	Hainich-Duen	51,0010	10,4300
EUBUG_296_f_Berymino3	**KM021782**	Berytidae	***Berytinus minor***	Thuringia	Hainich-Duen	51,0010	10,4300
EUBUG_Seq26_m_Berymino	**KM021929**	Berytidae	***Berytinus minor***	Thuringia	Hainich-Duen	51,0007	10,4299
BC_ZSM_HETA_0901	**KM021758**	Berytidae	***Berytinus montivagus***	Bavaria	Nuernberg, Reichelsdorf	49,3861	11,0303
BC_ZSM_HETA_0902	**KM022928**	Berytidae	***Berytinus montivagus***	Bavaria	Erlangen, Exerzierplatz	49,5861	11,0308
BC_ZSM_HETA_0903	**KM021518**	Berytidae	***Berytinus signoreti***	Austria	Hinterriss	47,4723	11,4668
EUBUG_Seq33_m_Berysign	**KM021861**	Berytidae	***Berytinus signoreti***	Thuringia	Hainich-Duen	51,2836	10,4246
BFB_Heteroptera_Kuechler_0331	**KM022708**	Berytidae	***Gampsocoris punctipes***	France	La Garde-Freinet	43,3300	6,4700
BFB_Heteroptera_Kuechler_0332	**KM021995**	Berytidae	***Gampsocoris punctipes***	France	La Garde-Freinet	43,3300	6,4700
EUBUG_Seq5_f_Gamppunc	**KM023109**	Berytidae	***Gampsocoris punctipes***	Baden-Wuerttemberg	Swabian Alb	48,3914	9,3768
BC_ZSM_HETA_0907	**KM022846**	Berytidae	***Metatropis rufescens***	Bavaria	Munich, Kapuzinerhoelzl	48,1672	11,4919
BFB_Heteroptera_Kuechler_0286	**KM022366**	Berytidae	***Metatropis rufescens***	Bavaria	Bayreuth, University	49,9290	11,5830
BFB_Heteroptera_Kuechler_0287	**KM022777**	Berytidae	***Metatropis rufescens***	Bavaria	Schweinfurt	50,0510	10,2540
EUBUG_379_f_Metarufe1	**KM022737**	Berytidae	***Metatropis rufescens***	Bavaria	Neuburg an der Donau	48,7460	11,2710
BC_ZSM_HETA_0890	**KM022501**	Berytidae	***Neides tipularius***	Bavaria	Fuerth, nahe Hainberg	49,4744	10,9959
BC_ZSM_HETA_0891	**KM022707**	Berytidae	***Neides tipularius***	Bavaria	Erlangen, Exerzierplatz	49,5861	11,0308
BC_ZSM_HETA_0892	**KM022120**	Berytidae	***Neides tipularius***	Austria	Oberweiden	48,3014	16,8165
BC_ZSM_HETA_0893	**KM021972**	Berytidae	***Neides tipularius***	Austria	Droesing, Waltersdorf, Noe	48,5289	16,8883
BFB_Heteroptera_Schmolke_0517	**KM022271**	Berytidae	***Neides tipularius***	Brandenburg	Mallnow Oderhaenge	52,4701	14,4781
BFB_Heteroptera_Schmolke_0520	**KM021810**	Berytidae	***Neides tipularius***	Brandenburg	Berlin, Spandau	52,5221	13,1902
BFB_Heteroptera_Schmolke_0339	**KM021592**	Blissidae	***Dimorphopterus spinolae***	Austria	Oggau	47,8754	16,7782

BFB_Heteroptera_Kuechler_0008	**KM023047**	Blissidae	***Ischnodemus sabuleti***	Bavaria	Bayreuth, Mistelbach	49,9310	11,5370
BFB_Heteroptera_Kuechler_0009	**KM022287**	Blissidae	***Ischnodemus sabuleti***	Bavaria	Bayreuth, Mistelbach	49,9310	11,5370
BFB_Heteroptera_Schmolke_0691	**KM022084**	Blissidae	***Ischnodemus sabuleti***	Bavaria	Pleinfeld, Mandlesmuehle	49,1267	10,9778
BFB_Heteroptera_Schmolke_0692	**KM022645**	Blissidae	***Ischnodemus sabuleti***	Bavaria	Kallmuenz, Mailerberg	49,1842	11,9450
BFB_Heteroptera_Schmolke_0693	**KM022070**	Blissidae	***Ischnodemus sabuleti***	Bavaria	Erlangen	49,5947	10,9836
BFB_Heteroptera_Schmolke_0694	**KM023123**	Blissidae	***Ischnodemus sabuleti***	Bavaria	Huegelmuehle	49,1731	10,9847
EUBUG_1395_m_Ischsabu6	**KM023071**	Blissidae	***Ischnodemus sabuleti***	Baden-Wuerttemberg	Swabian Alb	48,3970	9,4350
EUBUG_688_f_Ischsabu2	**KM022485**	Blissidae	***Ischnodemus sabuleti***	Baden-Wuerttemberg	Pfrunger Ried	47,8990	9,3900
EUBUG_689_f_Ischsabu3	**KM022054**	Blissidae	***Ischnodemus sabuleti***	Baden-Wuerttemberg	Pfrunger Ried	47,8990	9,3900
EUBUG_690_m_Ischsabu4	**KM021545**	Blissidae	***Ischnodemus sabuleti***	Baden-Wuerttemberg	Pfrunger Ried	47,8990	9,3900
EUBUG_691_m_Ischsabu5	**KM022127**	Blissidae	***Ischnodemus sabuleti***	Baden-Wuerttemberg	Pfrunger Ried	47,8990	9,3900
BC_ZSM_HETA_0939	**KM022981**	Coreidae	***Arenocoris fallenii***	Bavaria	Pollanten	49,1511	11,4433
BC_ZSM_HETA_0940	**KM021555**	Coreidae	***Arenocoris fallenii***	Bavaria	Fuerth, nahe Hainberg	49,4744	10,9959
BC_ZSM_HETA_0941	**KM021963**	Coreidae	***Arenocoris fallenii***	Bavaria	Nuernberg, Reichelsdorf	49,3861	11,0303
BC_ZSM_HETA_0942	**KM022477**	Coreidae	***Bathysolen nubilus***	Bavaria	Muehlhausen	49,2150	11,4569
BC_ZSM_HETA_0943	**KM021746**	Coreidae	***Bathysolen nubilus***	Bavaria	Muehlhausen	48,7822	11,7858
BC_ZSM_HETA_0944	**KM022325**	Coreidae	***Bathysolen nubilus***	Bavaria	Erlangen, Exerzierplatz	49,5861	11,0308
BC_ZSM_HETA_0945	**KM022863**	Coreidae	***Bathysolen nubilus***	Bavaria	Obereichstaett	48,8978	11,1228
BC_ZSM_HETA_0946	**KM022362**	Coreidae	***Ceraleptus gracilicornis***	Bavaria	Scheinfeld	49,6739	10,4669
BFB_Heteroptera_Schmolke_0351	**KM022542**	Coreidae	***Ceraleptus gracilicornis***	Bavaria	Pollanten	49,1511	11,4433
BC_ZSM_HETA_0947	**KM022873**	Coreidae	***Ceraleptus lividus***	Bavaria	Offenstetten	48,8111	11,9106
BC_ZSM_HETA_0917	**KM021642**	Coreidae	***Coreus marginatus***	Bavaria	Pollanten, Reismuehle	49,1511	11,4433
BC_ZSM_HETA_0919	**KM021461**	Coreidae	***Coreus marginatus***	Bavaria	Munich, Allacher Heide	48,2008	11,4828
BC_ZSM_HETA_0920	**KM022035**	Coreidae	***Coreus marginatus***	Austria	Droesing, Waltersdorf, Noe	48,5289	16,8883
BFB_Heteroptera_Kuechler_0097	**KM022347**	Coreidae	***Coreus marginatus***	Bavaria	Bayreuth, Saas	49,9110	11,5520
BFB_Heteroptera_Kuechler_0098	**KM022692**	Coreidae	***Coreus marginatus***	Bavaria	Bayreuth, Saas	49,9110	11,5520
EUBUG_111_m_Coremarg1	**KM022191**	Coreidae	***Coreus marginatus***	North Rhine-Westphalia	Bonn, Mehlem	50,6620	7,1910
EUBUG_112_m_Coremarg2	**KM022555**	Coreidae	***Coreus marginatus***	North Rhine-Westphalia	Bonn, Mehlem	50,6620	7,1910
EUBUG_191_f_Coremarg3	**KM021437**	Coreidae	***Coreus marginatus***	Baden-Wuerttemberg	Swabian Alb	48,3810	9,4190
BC_ZSM_HETA_0948	**KM022218**	Coreidae	***Coriomeris denticulatus***	Bavaria	Munich, Rangierbahnhof	48,1906	11,5269
BC_ZSM_HETA_0949	**KM021934**	Coreidae	***Coriomeris denticulatus***	Bavaria	Bamberg, Boerstig	49,9046	10,9012
BC_ZSM_HETA_0950	**KM022764**	Coreidae	***Coriomeris denticulatus***	Bavaria	Karlstadt, Wiesenfeld	49,9842	9,7069
BFB_Heteroptera_Schmolke_0352	**KM021531**	Coreidae	***Coriomeris denticulatus***	Bavaria	Pollanten	49,1511	11,4433

EUBUG_101_m_Corident1	**KM022586**	Coreidae	***Coriomeris denticulatus***	Lower Saxony	Wahnbek	53,2050	8,2460
EUBUG_423_m_Corident3	**KM022275**	Coreidae	***Coriomeris denticulatus***	Thuringia	Hainich-Duen	51,3020	10,4360
EUBUG_890_m_Corident4	**KM022816**	Coreidae	***Coriomeris denticulatus***	Thuringia	Jena	50,9510	11,6240
EUBUG_891_f_Corident5	**KM022067**	Coreidae	***Coriomeris denticulatus***	Thuringia	Jena	50,9510	11,6240
EUBUG_892_m_Corident6	**KM022050**	Coreidae	***Coriomeris denticulatus***	Thuringia	Jena	50,9510	11,6240
BC_ZSM_HETA_0921	**KM021533**	Coreidae	***Enoplops scapha***	Bavaria	Schernfeld	48,9114	11,1164
BC_ZSM_HETA_0922	**KM021720**	Coreidae	***Enoplops scapha***	Bavaria	Lengries am Sylvensteinsee	47,5950	11,5536
BC_ZSM_HETA_0923	**KM022141**	Coreidae	***Enoplops scapha***	Bavaria	Weichs, Eglersried	48,4117	11,4031
BC_ZSM_HETA_0924	**KM021912**	Coreidae	***Enoplops scapha***	Bavaria	Schwaig, Fuererstein	49,4733	11,2631
BFB_Heteroptera_Schmolke_0516	**KM022785**	Coreidae	***Enoplops scapha***	Brandenburg	Mallnow Oderhaenge	52,4701	14,4781
BC_ZSM_HETA_0925	**KM021526**	Coreidae	***Gonocerus acuteangulatus***	Bavaria	Munich, Rangierbahnhof	48,1906	11,5269
BC_ZSM_HETA_0926	**KM022772**	Coreidae	***Gonocerus acuteangulatus***	Bavaria	Kallmuenz, Mailerberg	49,1842	11,9450
BC_ZSM_HETA_0927	**KM022300**	Coreidae	***Gonocerus acuteangulatus***	Bavaria	Munich, Moosach	48,1808	11,5244
BC_ZSM_HETA_0928	**KM022188**	Coreidae	***Gonocerus acuteangulatus***	Bavaria	Martinsried	48,1122	11,4622
EUBUG_334_m_Gonoacut1	**KM022835**	Coreidae	***Gonocerus acuteangulatus***	Baden-Wuerttemberg	Fronreute	47,8390	9,6060
EUBUG_508_f_Gonoacut3	**KM022806**	Coreidae	***Gonocerus acuteangulatus***	Lower Saxony	Wangerooge	53,7940	7,8970
BC_ZSM_HETA_0929	**KM021908**	Coreidae	***Gonocerus juniperi***	Bavaria	Munich, Moosach	48,1808	11,5244
BC_ZSM_HETA_0930	**KM022253**	Coreidae	***Gonocerus juniperi***	Bavaria	Koenigsbrunn, Hasenheide	48,2744	10,9033
BC_ZSM_HETA_0931	**KM022935**	Coreidae	***Gonocerus juniperi***	Bavaria	Obereichstaett	48,8978	11,1228
BC_ZSM_HETA_0932	**KM021818**	Coreidae	***Gonocerus juniperi***	Bavaria	Kallmuenz, Mailerberg	49,1842	11,9450
BFB_Heteroptera_Kuechler_0154	**KM021913**	Coreidae	***Gonocerus juniperi***	Bavaria	Bayreuth, Bindlacher Berg	50,0070	11,6140
BFB_Heteroptera_Kuechler_0217	**KM022046**	Coreidae	***Gonocerus juniperi***	Bavaria	Bayreuth, Bindlacher Berg	50,0070	11,6140
EUBUG_771_m_Leptocci1	**KM022489**	Coreidae	***Leptoglossus occidentalis***	Bavaria	Munich	47,8990	9,3900
EUBUG_772_m_Leptocci2	**KM022493**	Coreidae	***Leptoglossus occidentalis***	Bavaria	Munich	47,8990	9,3900
BC_ZSM_HETA_0934	**KM022719**	Coreidae	***Spathocera dalmanii***	Bavaria	Straubing	48,9019	12,6675
BFB_Heteroptera_Schmolke_0349	**KM022957**	Coreidae	***Spathocera dalmanii***	Bavaria	Pollanten	49,1511	11,4433
BFB_Heteroptera_Schmolke_0350	**KM021701**	Coreidae	***Spathocera dalmanii***	Bavaria	Siegenburg	48,7589	11,8383
BC_ZSM_HETA_0936	**KM021921**	Coreidae	***Syromastus rhombeus***	Bavaria	Erlangen, Exerzierplatz	49,5861	11,0308
BC_ZSM_HETA_0937	**KM022979**	Coreidae	***Syromastus rhombeus***	Bavaria	Muehlhausen	48,7822	11,7858
BC_ZSM_HETA_0938	**KM023045**	Coreidae	***Syromastus rhombeus***	Bavaria	Pleinfeld, Heinzenmuehle	49,1378	11,0158
BFB_Heteroptera_Schmolke_0542	**KM022699**	Coreidae	***Syromastus rhombeus***	Brandenburg	Berlin, Tegel	52,5874	13,2842
BC ZSM AQU 00307	**KM022441**	Corixidae	***Arctocorisa carinata***	Bavaria	Rosenheim	47,6470	12,0650
BC ZSM AQU 00203	**HM421991**	Corixidae	***Callicorixa praeusta***	Bavaria	Weilheim-Schongau	47,7310	11,3120

BCZSMAQU001076	**KM022519**	Corixidae	***Corixa punctata***	Bavaria	Dingolfing-Landau	48,5700	12,5100
BCZSMAQU001077	**KM021617**	Corixidae	***Cymatia coleoptrata***	Bavaria	Dingolfing-Landau	48,7300	12,7500
BCZSMAQU001078	**KM021613**	Corixidae	***Cymatia coleoptrata***	Bavaria	Rosenheim	47,8900	12,3600
BCZSMAQU001079	**KM022834**	Corixidae	***Cymatia rogenhoferi***	Bavaria	Dingolfing-Landau	48,6500	12,5100
BFB_Heteroptera_Schmolke_0546	**KM022522**	Corixidae	***Glaenocorisa propinqua***	Bavaria	Lake Murner	49,3483	12,2108
BFB_Heteroptera_Schmolke_0547	**KM022715**	Corixidae	***Glaenocorisa propinqua***	Bavaria	Lake Murner	49,3483	12,2108
BFB_Heteroptera_Schmolke_0548	**KM021511**	Corixidae	***Glaenocorisa propinqua***	Bavaria	Lake Murner	49,3483	12,2108
BFB_Heteroptera_Schmolke_0549	**KM021539**	Corixidae	***Glaenocorisa propinqua***	Bavaria	Lake Murner	49,3483	12,2108
BFB_Heteroptera_Schmolke_0550	**KM022611**	Corixidae	***Glaenocorisa propinqua***	Bavaria	Lake Murner	49,3483	12,2108
BCZSMAQU001084	**KM022319**	Corixidae	***Hesperocorixa linnaei***	Bavaria	Rosenheim	47,8900	12,3600
BC ZSM AQU 00206	**KM022788**	Corixidae	***Hesperocorixa sahlbergi***	Bavaria	Ansbach	49,0320	10,3690
BC ZSM AQU 00704	**HQ563133**	Corixidae	***Hesperocorixa sahlbergi***	Bavaria	Erding	48,3930	11,9100
BCZSMAQU001085	**KM021595**	Corixidae	***Hesperocorixa sahlbergi***	Bavaria	Rosenheim	47,8900	12,3600
GBOL00361	**KM022623**	Corixidae	***Hesperocorixa sahlbergi***	Brandenburg	Zerwelinsee	53,3001	13,6214
BC ZSM AQU 00001	**HM376113**	Corixidae	***Micronecta griseola***	Bavaria	Freising	48,4030	11,7411
BCZSMAQU001093	**KM021954**	Corixidae	***Micronecta griseola***	Bavaria	Dillingen an der Donau	48,5300	10,3900
BCZSMAQU001094	**KM022072**	Corixidae	***Micronecta poweri***	Bavaria	Dillingen an der Donau	48,5100	10,3900
BC ZSM AQU 00077	**HM376150**	Corixidae	***Micronecta scholtzi***	Bavaria	Neuburg-Schrobenhausen	48,6987	11,1936
BCZSMAQU001101	**KM023136**	Corixidae	***Paracorixa concinna***	Bavaria	Starnberg	47,9700	11,3500
BC ZSM AQU 00016	**GU682186**	Corixidae	***Sigara falleni***	Bavaria	Traunstein	48,0000	12,8400
BC ZSM AQU 00162	**GU682177**	Corixidae	***Sigara falleni***	Bavaria	Neustadt an der Aisch	49,5300	10,3400
BC ZSM AQU 00712	**HQ563140**	Corixidae	***Sigara falleni***	Bavaria	Landsberg am Lech	48,0130	10,8760
BCZSMAQU001102	**KM021936**	Corixidae	***Sigara falleni***	Bavaria	Dillingen an der Donau	48,5100	10,3900
BC ZSM AQU 00210	**HM421994**	Corixidae	***Sigara fossarum***	Bavaria	Regen	49,0990	13,1550
BCZSMAQU001103	**KM022318**	Corixidae	***Sigara fossarum***	Bavaria	Freising	48,4600	11,7300
BC ZSM AQU 00164	**HQ948123**	Corixidae	***Sigara lateralis***	Bavaria	Neustadt an der Aisch	49,5300	10,3400
BCZSMAQU001106	**KM021983**	Corixidae	***Sigara nigrolineata***	Bavaria	Rosenheim	47,7500	12,2600
BCZSMAQU001107	**KM021618**	Corixidae	***Sigara nigrolineata***	Bavaria	Eichstaett	48,8200	11,5000
BCZSMAQU001108	**KM022152**	Corixidae	***Sigara semistriata***	Bavaria	Rosenheim	47,8900	12,3600
BC ZSM AQU 00211	**HM421995**	Corixidae	***Sigara striata***	Bavaria	Ansbach	49,0320	10,3690
EUBUG_567_f_Sigavenu1	**KM021675**	Corixidae	***Sigara venusta***	Rhineland Palatinate	NSG Moosbachtal	49,1610	7,7560
EUBUG_568_m_Sigavenu2	**KM021928**	Corixidae	***Sigara venusta***	Rhineland Palatinate	NSG Moosbachtal	49,1610	7,7560
BFB_Heteroptera_Schmolke_0512	**KM021835**	Cydnidae	***Legnotus limbosus***	Brandenburg	Berlin, Tegel	52,5874	13,2842

BFB_Heteroptera_Schmolke_0525	**KM022746**	Cydnidae	***Microporus nigrita***	Brandenburg	Berlin, Spandau	52,5221	13,1902
BFB_Heteroptera_Kuechler_0060	**KM022113**	Cydnidae	***Tritomegas bicolor***	Bavaria	Schweinfurt	50,0460	10,2400
BFB_Heteroptera_Kuechler_0113	**KM022290**	Cydnidae	***Tritomegas bicolor***	Bavaria	Bayreuth	49,9260	11,5620
BFB_Heteroptera_Schmolke_0358	**KM022521**	Cydnidae	***Tritomegas sexmaculatus***	Austria	Oggau	47,8754	16,7782
BFB_Heteroptera_Schmolke_0526	**KM021915**	Cydnidae	***Tritomegas sexmaculatus***	Brandenburg	Berlin, Spandau	52,5221	13,1902
BFB_Heteroptera_Kuechler_0046	**KM022871**	Cymidae	***Cymus aurescens***	Bavaria	Bayreuth, Studentenwald	49,9160	11,5700
BFB_Heteroptera_Kuechler_0052	**KM022895**	Cymidae	***Cymus aurescens***	Bavaria	Bayreuth, Botanic Garden	49,9230	11,5830
BFB_Heteroptera_Schmolke_0678	**KM022574**	Cymidae	***Cymus aurescens***	Bavaria	Achslach	48,9742	12,9362
EUBUG_1202_m_Cymuaure3	**KM022822**	Cymidae	***Cymus aurescens***	Bavaria	Bavarian Forest	49,0540	13,2480
EUBUG_864_m_Cymuaure1	**KM022670**	Cymidae	***Cymus aurescens***	Rhineland Palatinate	Fischbach bei Dahn	49,0860	7,7230
EUBUG_865_f_Cymuaure2	**KM022162**	Cymidae	***Cymus aurescens***	Rhineland Palatinate	Fischbach bei Dahn	49,0860	7,7230
BFB_Heteroptera_Kuechler_0051	**KM022410**	Cymidae	***Cymus claviculus***	Bavaria	Bayreuth, Botanic Garden	49,9230	11,5830
BFB_Heteroptera_Kuechler_0053	**KM022953**	Cymidae	***Cymus claviculus***	Bavaria	Bayreuth, Botanic Garden	49,9230	11,5830
EUBUG_470_f_Cymuclav1	**KM022041**	Cymidae	***Cymus claviculus***	Brandenburg	Schorfheide-Chorin	53,0980	13,6260
BFB_Heteroptera_Kuechler_0050	**KM022438**	Cymidae	***Cymus glandicolor***	Bavaria	Bayreuth, Botanic Garden	49,9230	11,5830
EUBUG_1200_m_Cymuglan3	**KM021608**	Cymidae	***Cymus glandicolor***	Bavaria	Bavarian Forest	49,0540	13,2480
EUBUG_1201_f_Cymuglan4	**KM021902**	Cymidae	***Cymus glandicolor***	Bavaria	Bavarian Forest	49,0540	13,2480
EUBUG_530_f_Cymuglan1	**KM022361**	Cymidae	***Cymus glandicolor***	Baden-Wuerttemberg	Pfrunger Ried	47,8990	9,3900
EUBUG_531_m_Cymuglan2	**KM022825**	Cymidae	***Cymus glandicolor***	Baden-Wuerttemberg	Pfrunger Ried	47,8990	9,3900
BFB_Heteroptera_Kuechler_0049	**KM022545**	Cymidae	***Cymus melanocephalus***	Bavaria	Bayreuth, Botanic Garden	49,9230	11,5830
BFB_Heteroptera_Kuechler_0070	**KM022580**	Cymidae	***Cymus melanocephalus***	Bavaria	Creussen	49,8490	11,5960
BFB_Heteroptera_Schmolke_0340	**KM021578**	Cymidae	***Cymus melanocephalus***	Bavaria	Pollanten	49,1511	11,4433
BFB_Heteroptera_Kuechler_0308	**KM022926**	Geocoridae	***Geocoris ater***	Rhineland Palatinate	Fischbach bei Dahn	49,1290	7,6900
BFB_Heteroptera_Kuechler_0222	**KM022291**	Geocoridae	***Geocoris dispar***	Bavaria	Bayreuth, Botanic Garden	49,9230	11,5850
BFB_Heteroptera_Kuechler_0328	**KM021499**	Geocoridae	***Geocoris erythrocephalus***	France	La Garde-Freinet	43,3300	6,4700
BFB_Heteroptera_Kuechler_0291	**KM021943**	Geocoridae	***Geocoris grylloides***	Rhineland Palatinate	Fischbach bei Dahn	49,0910	7,7150
BFB_Heteroptera_Kuechler_0292	**KM022838**	Geocoridae	***Geocoris grylloides***	Rhineland Palatinate	Fischbach bei Dahn	49,0910	7,7150
BFB_Heteroptera_Schmolke_0696	**KM021986**	Geocoridae	***Geocoris grylloides***	Bavaria	Pleinfeld, Mandlesmuehle	49,1267	10,9778
EUBUG_870_f_Geocgryl1	**KM022381**	Geocoridae	***Geocoris grylloides***	Rhineland Palatinate	Fischbach bei Dahn	49,0900	7,7150
BC ZSM AQU 00697	**HQ563129**	Gerridae	***Aquarius najas***	Bavaria	Freising	48,4900	11,9480
BC ZSM AQU 00078	**GU682185**	Gerridae	***Aquarius paludum***	Bavaria	Neuburg-Schrobenhausen	48,6987	11,1936
BC ZSM AQU 00698	**HQ563130**	Gerridae	***Aquarius paludum***	Bavaria	Schwandorf	49,0380	12,1870
BC ZSM AQU 00204	**KM022591**	Gerridae	***Gerris argentatus***	Bavaria	Traunstein	48,0210	12,8430

BC ZSM AQU 00699	**HQ563131**	Gerridae	***Gerris asper***	Bavaria	Erding	48,3930	11,9100
BCZSMAQU001080	**KM022885**	Gerridae	***Gerris costae***	Bavaria	Miesbach	47,5900	11,7300
BCZSMAQU001081	**KM023053**	Gerridae	***Gerris gibbifer***	Bavaria	Weilheim-Schongau	47,7600	11,1700
BC ZSM AQU 00021	**KM022763**	Gerridae	***Gerris lacustris***	Bavaria	Traunstein	48,0000	12,8400
BC ZSM AQU 00163	**KM021899**	Gerridae	***Gerris lacustris***	Bavaria	Traunstein	48,0000	12,8400
BFB_Heteroptera_Schmolke_012	**KM021956**	Gerridae	***Gerris lacustris***	Bavaria	Munich	48,1906	11,5269
BFB_Heteroptera_Schmolke_013	**KM021498**	Gerridae	***Gerris lacustris***	Bavaria	Lenggries	47,5950	11,5536
BFB_Heteroptera_Schmolke_014	**KM022608**	Gerridae	***Gerris lacustris***	Bavaria	Munich	48,1914	11,4847
EUBUG_527_m_Gerrlacu1	**KM021620**	Gerridae	***Gerris lacustris***	Baden-Wuerttemberg	Pfrunger Ried	47,8990	9,3900
BC ZSM AQU 00171	**KM022818**	Gerridae	***Gerris odontogaster***	Bavaria	Neustadt an der Aisch	49,5300	10,3400
BCZSMAQU001082	**KM022343**	Gerridae	***Gerris odontogaster***	Bavaria	Munich	48,2000	11,7100
BFB_Heteroptera_Schmolke_009	**KM022516**	Gerridae	***Gerris odontogaster***	Bavaria	Sachsenkam	47,8008	11,6025
BCZSMAQU001083	**KM021632**	Gerridae	***Gerris thoracicus***	Bavaria	Munich	48,0900	11,5500
BFB_Heteroptera_Schmolke_010	**KM021828**	Gerridae	***Gerris thoracicus***	Bavaria	Munich	48,2114	11,6083
BFB_Heteroptera_Schmolke_011	**KM022136**	Gerridae	***Gerris thoracicus***	Bavaria	Munich	48,2008	11,4828
BCZSMAQU001090	**KM022793**	Gerridae	***Limnoporus rufoscutellatus***	Bavaria	Rosenheim	47,8900	12,3600
GBOL00360	**KM021794**	Gerridae	***Limnoporus rufoscutellatus***	Brandenburg	Schlalach	52,1468	12,8450
BC ZSM AQU 00703	**HQ563132**	Hebridae	***Hebrus pusillus***	Bavaria	Erding	48,3930	11,9100
BFB_Heteroptera_Kuechler_0114	**KM021514**	Hebridae	***Hebrus pusillus***	Bavaria	Bayreuth, Botanic Garden	49,9230	11,5830
BC ZSM AQU 00096	**GU682178**	Hebridae	***Hebrus ruficeps***	Bavaria	Ingolstadt	48,7665	11,4257
BC ZSM AQU 00205	**GU682189**	Hebridae	***Hebrus ruficeps***	Bavaria	Ansbach	49,0320	10,3690
BC ZSM AQU 00705	**HQ563134**	Hydrometridae	***Hydrometra gracilenta***	Bavaria	Rosenheim	47,8960	12,1660
BCZSMAQU001086	**KM021938**	Hydrometridae	***Hydrometra gracilenta***	Bavaria	Weilheim-Schongau	47,9200	11,2000
BFB_Heteroptera_Kuechler_0068	**KM022739**	Hydrometridae	***Hydrometra gracilenta***	Bavaria	Creussen	49,8490	11,5960
BFB_Heteroptera_Kuechler_0069	**KM023023**	Hydrometridae	***Hydrometra gracilenta***	Bavaria	Creussen	49,8490	11,5960
GBOL00363	**KM021900**	Hydrometridae	***Hydrometra gracilenta***	Brandenburg	Zerwelinsee	53,3001	13,6214
BC ZSM AQU 00034	**HM376130**	Hydrometridae	***Hydrometra stagnorum***	Bavaria	Traunstein	48,0000	12,8400
BCZSMAQU001087	**KM022870**	Hydrometridae	***Hydrometra stagnorum***	Bavaria	Eichstaett	49,0300	11,4800
BCZSMAQU001088	**KM022097**	Hydrometridae	***Hydrometra stagnorum***	Bavaria	Landshut	48,6200	12,1800
BFB_Heteroptera_Schmolke_004	**KM021920**	Hydrometridae	***Hydrometra stagnorum***	Bavaria	Munich	48,2114	11,6083
BFB_Heteroptera_Schmolke_0529	**KM022352**	Hydrometridae	***Hydrometra stagnorum***	Brandenburg	Berlin, Spandau	52,5221	13,1902
BFB_Heteroptera_Kuechler_0010	**KM021456**	Lygaeidae	***Arocatus longiceps***	Bavaria	Schweinfurt	50,0480	10,2170
BFB_Heteroptera_Kuechler_0011	**KM021453**	Lygaeidae	***Arocatus longiceps***	Bavaria	Schweinfurt	50,0480	10,2170

BFB_Heteroptera_Schmolke_0556	**KM022767**	Lygaeidae	***Arocatus longiceps***	North Rhine-Westphalia	Cologne	50,9584	6,9612
BFB_Heteroptera_Schmolke_0557	**KM022837**	Lygaeidae	***Arocatus longiceps***	North Rhine-Westphalia	Cologne	50,9584	6,9612
BFB_Heteroptera_Schmolke_0558	**KM023035**	Lygaeidae	***Arocatus longiceps***	North Rhine-Westphalia	Cologne	50,9584	6,9612
BFB_Heteroptera_Schmolke_0559	**KM022467**	Lygaeidae	***Arocatus longiceps***	North Rhine-Westphalia	Cologne	50,9584	6,9612
BFB_Heteroptera_Schmolke_0560	**KM022881**	Lygaeidae	***Arocatus longiceps***	North Rhine-Westphalia	Cologne	50,9584	6,9612
EUBUG_761_f_Aroclong1	**KM022482**	Lygaeidae	***Arocatus longiceps***	Bavaria	Munich	48,1130	11,3880
EUBUG_762_f_Aroclong2	**KM022373**	Lygaeidae	***Arocatus longiceps***	Bavaria	Munich	48,1130	11,3880
EUBUG_763_f_Aroclong3	**KM021509**	Lygaeidae	***Arocatus longiceps***	Bavaria	Munich	48,1130	11,3880
EUBUG_764_m_Aroclong4	**KM022865**	Lygaeidae	***Arocatus longiceps***	Bavaria	Munich	48,1130	11,3880
EUBUG_765_m_Aroclong5	**KM022272**	Lygaeidae	***Arocatus longiceps***	Bavaria	Munich	47,8990	9,3900
BFB_Heteroptera_Schmolke_0510	**KM023050**	Lygaeidae	***Arocatus roeselii***	North Rhine-Westphalia	Cologne	50,9584	6,9612
BFB_Heteroptera_Schmolke_0511	**KM022856**	Lygaeidae	***Arocatus roeselii***	North Rhine-Westphalia	Cologne	50,9584	6,9612
BFB_Heteroptera_Kuechler_0344	**KM022627**	Lygaeidae	***Belonochilus numenius***	France	La Garde-Freinet	43,3300	6,4700
BFB_Heteroptera_Kuechler_0345	**KM021622**	Lygaeidae	***Henestaris halophilus***	France	La Garde-Freinet	43,3300	6,4700
BFB_Heteroptera_Kuechler_0346	**KM022462**	Lygaeidae	***Henestaris laticeps***	France	L`Escalet	43,1880	6,6410
BFB_Heteroptera_Kuechler_0374	**KM021738**	Lygaeidae	***Kleidocerys ericae***	Austria	Fitunser Huette	46,7900	12,5620
BFB_Heteroptera_Kuechler_0004	**KM022383**	Lygaeidae	***Kleidocerys resedae***	Bavaria	Destuben	49,9020	11,5680
BFB_Heteroptera_Kuechler_0018	**KM022281**	Lygaeidae	***Kleidocerys resedae***	Bavaria	Bayreuth, Botanic Garden	49,9230	11,5830
BFB_Heteroptera_Schmolke_0676	**KM022334**	Lygaeidae	***Kleidocerys resedae***	Bavaria	Erlangen	49,5947	10,9836
EUBUG_337_m_Kleirese4	**KM023121**	Lygaeidae	***Kleidocerys resedae***	Baden-Wuerttemberg	Fronreute	47,8390	9,6060
EUBUG_338_m_Kleirese5	**KM022847**	Lygaeidae	***Kleidocerys resedae***	Baden-Wuerttemberg	Fronreute	47,8390	9,6060
EUBUG_339_m_Kleirese6	**KM023114**	Lygaeidae	***Kleidocerys resedae***	Baden-Wuerttemberg	Fronreute	47,8390	9,6060
EUBUG_340_f_Kleirese7	**KM022655**	Lygaeidae	***Kleidocerys resedae***	Baden-Wuerttemberg	Fronreute	47,8390	9,6060
EUBUG_341_f_Kleirese8	**KM022734**	Lygaeidae	***Kleidocerys resedae***	Baden-Wuerttemberg	Fronreute	47,8390	9,6060
EUBUG_510_f_Kleidrese9	**KM022728**	Lygaeidae	***Kleidocerys resedae***	Lower Saxony	Wahnbek	53,7940	7,8970
EUBUG_737_f_Kleieric1	**KM022176**	Lygaeidae	***Kleidocerys resedae***	Rhineland Palatinate	Fischbach bei Dahn	49,1040	7,6830
EUBUG_738_f_Kleieric2	**KM023118**	Lygaeidae	***Kleidocerys resedae***	Rhineland Palatinate	Fischbach bei Dahn	49,1040	7,6830
EUBUG_739_f_Kleieric3	**KM022958**	Lygaeidae	***Kleidocerys resedae***	Rhineland Palatinate	Fischbach bei Dahn	49,1040	7,6830
EUBUG_740_m_Kleieric4	**KM022096**	Lygaeidae	***Kleidocerys resedae***	Rhineland Palatinate	Fischbach bei Dahn	49,1040	7,6830
EUBUG_741_m_Kleieric5	**KM022233**	Lygaeidae	***Kleidocerys resedae***	Rhineland Palatinate	Fischbach bei Dahn	49,1040	7,6830
BFB_Heteroptera_Kuechler_0108	**KM021798**	Lygaeidae	***Lygaeus equestris***	Bavaria	Bayreuth, Neubuerg	49,8920	11,4020
BFB_Heteroptera_Kuechler_0109	**KM023009**	Lygaeidae	***Lygaeus equestris***	Bavaria	Bayreuth, Neubuerg	49,8920	11,4020
BFB_Heteroptera_Kuechler_0339	**KM021919**	Lygaeidae	***Lygaeus equestris***	France	La Garde-Freinet	43,3300	6,4700

BFB_Heteroptera_Schmolke_0337	**KM022264**	Lygaeidae	***Lygaeus equestris***	Bavaria	Kelheimwinzer	48,9175	11,9178
BFB_Heteroptera_Schmolke_0636	**KM023135**	Lygaeidae	***Lygaeus equestris***	Bavaria	Siegenburg, Dassfeld	48,7589	11,8383
BFB_Heteroptera_Schmolke_0637	**KM022143**	Lygaeidae	***Lygaeus equestris***	Bavaria	Munich, Moosach	48,1808	11,5244
BFB_Heteroptera_Schmolke_0638	**KM022263**	Lygaeidae	***Lygaeus equestris***	Bavaria	Kallmuenz, Mailerberg	49,1842	11,9450
BFB_Heteroptera_Schmolke_0639	**KM021733**	Lygaeidae	***Lygaeus simulans***	Bavaria	Obereichstaett	48,8978	11,1228
BFB_Heteroptera_Kuechler_0372	**KM022630**	Lygaeidae	***Nithecus jacobaeae***	Bavaria	Bayreuth, Botanic Garden	49,9230	11,5850
BFB_Heteroptera_Kuechler_0373	**KM021490**	Lygaeidae	***Nithecus jacobaeae***	Bavaria	Bayreuth, Botanic Garden	49,9230	11,5850
BFB_Heteroptera_Schmolke_0647	**KM022932**	Lygaeidae	***Nithecus jacobaeae***	Austria	Oberweiden	48,3325	16,8647
BFB_Heteroptera_Schmolke_0648	**KM022010**	Lygaeidae	***Nithecus jacobaeae***	Bavaria	Ruhpolding, Brand	47,7406	12,5942
BFB_Heteroptera_Schmolke_0649	**KM021770**	Lygaeidae	***Nithecus jacobaeae***	Bavaria	Lenggries, Sylvensteinsee	47,5950	11,5536
EUBUG_Seq16_f_Nysicymo	**KM022387**	Lygaeidae	***Nysius cymoides***	Baden-Wuerttemberg	Swabian Alb	48,3757	9,5186
BFB_Heteroptera_Schmolke_0650	**KM021892**	Lygaeidae	***Nysius ericae***	Bavaria	Urfeld	47,6200	11,3428
BFB_Heteroptera_Schmolke_0651	**KM021881**	Lygaeidae	***Nysius ericae***	Bavaria	Grainau, Friedergries	47,5654	11,0248
BFB_Heteroptera_Schmolke_0653	**KM022105**	Lygaeidae	***Nysius ericae***	Bavaria	Munich, Oberschleissheim	48,2269	11,5444
BFB_Heteroptera_Kuechler_0342	**KM022087**	Lygaeidae	***Nysius graminicola***	France	L`Escalet	43,1880	6,6410
BFB_Heteroptera_Kuechler_0343	**KM021691**	Lygaeidae	***Nysius graminicola***	France	L`Escalet	43,1880	6,6410
BFB_Heteroptera_Schmolke_0654	**KM021814**	Lygaeidae	***Nysius helveticus***	Bavaria	Pollanten, Reismuehle	49,1511	11,4433
BFB_Heteroptera_Schmolke_0655	**KM021882**	Lygaeidae	***Nysius helveticus***	Bavaria	Munich, Theresienwiese	48,1272	11,5464
BFB_Heteroptera_Schmolke_0657	**KM022940**	Lygaeidae	***Nysius helveticus***	Bavaria	Roth, Hofstetten	49,1669	11,1783
BFB_Heteroptera_Schmolke_0658	**KM022374**	Lygaeidae	***Nysius senecionis***	Bavaria	Leinburg, Fuchsmuehle	49,4425	11,2822
BFB_Heteroptera_Schmolke_0659	**KM021799**	Lygaeidae	***Nysius senecionis***	Bavaria	Pollanten, Reismuehle	49,1511	11,4433
BFB_Heteroptera_Schmolke_0661	**KM022754**	Lygaeidae	***Nysius senecionis***	Bavaria	Schernfeld	48,9114	11,1164
BFB_Heteroptera_Schmolke_0338	**KM021726**	Lygaeidae	***Nysius thymi***	Bavaria	Siegenburg	48,7589	11,8383
BFB_Heteroptera_Schmolke_0652	**KM022057**	Lygaeidae	***Nysius thymi***	Bavaria	Spalt, Muehlstetten	49,1543	11,0052
BFB_Heteroptera_Schmolke_0662	**KM021791**	Lygaeidae	***Nysius thymi***	Bavaria	Munich, Allacher Heide	48,2008	11,4828
BFB_Heteroptera_Schmolke_0663	**KM022653**	Lygaeidae	***Nysius thymi***	Bavaria	Offenstetten	48,8111	11,9106
BFB_Heteroptera_Schmolke_0664	**KM021999**	Lygaeidae	***Nysius thymi***	Bavaria	Munich, Allacher Heide	48,2008	11,4828
EUBUG_246_f_Nysithym1	**KM021751**	Lygaeidae	***Nysius thymi***	Brandenburg	Schorfheide-Chorin	53,1350	13,8760
EUBUG_247_m_Nysithym2	**KM022382**	Lygaeidae	***Nysius thymi***	Brandenburg	Schorfheide-Chorin	53,1350	13,8760
EUBUG_250_m_Nysithym3	**KM022620**	Lygaeidae	***Nysius thymi***	Brandenburg	Schorfheide-Chorin	53,1350	13,8760
EUBUG_766_m_Nysisene1	**KM022163**	Lygaeidae	***Nysius thymi***	Brandenburg	Schorfheide-Chorin	47,8990	9,3900
BFB_Heteroptera_Schmolke_0668	**KM022182**	Lygaeidae	***Orsillus depressus***	Bavaria	Kallmuenz, Mailerberg	49,1842	11,9450
BFB_Heteroptera_Kuechler_0249	**KM021605**	Lygaeidae	***Ortholomus punctipennis***	Bavaria	Bayreuth, Botanic Garden	49,9230	11,5850

BFB_Heteroptera_Schmolke_0656	**KM022936**	Lygaeidae	***Ortholomus punctipennis***	Bavaria	Nuernberg, Reichelsdorf	49,3822	11,0321
BFB_Heteroptera_Schmolke_0672	**KM021740**	Lygaeidae	***Ortholomus punctipennis***	Bavaria	Munich, northern Angerlohe	48,1914	11,4847
BFB_Heteroptera_Kuechler_0188	**KM022601**	Lygaeidae	***Platyplax salviae***	Bavaria	Staffelberg	50,0920	11,0240
BFB_Heteroptera_Kuechler_0189	**KM022884**	Lygaeidae	***Platyplax salviae***	Bavaria	Staffelberg	50,0920	11,0240
BFB_Heteroptera_Schmolke_0341	**KM022077**	Lygaeidae	***Platyplax salviae***	Bavaria	Pollanten	49,1511	11,4433
BFB_Heteroptera_Schmolke_0709	**KM022356**	Lygaeidae	***Platyplax salviae***	Bavaria	Isarauen nahe Tierpark	48,0961	11,5514
BFB_Heteroptera_Kuechler_0329	**KM022483**	Lygaeidae	***Spilostethus pandurus***	France	La Garde-Freinet	43,3300	6,4700
BFB_Heteroptera_Kuechler_0330	**KM021481**	Lygaeidae	***Spilostethus pandurus***	France	La Garde-Freinet	43,3300	6,4700
BFB_Heteroptera_Kuechler_0347	**KM022131**	Lygaeidae	***Spilostethus saxatilis***	Italy	Talamone	42,5610	11,1390
BFB_Heteroptera_Kuechler_0348	**KM022797**	Lygaeidae	***Spilostethus saxatilis***	Italy	Talamone	42,5610	11,1390
BFB_Heteroptera_Schmolke_0640	**KM022476**	Lygaeidae	***Spilostethus saxatilis***	Bavaria	Wegscheid	48,6036	13,7892
BFB_Heteroptera_Schmolke_0641	**KM023115**	Lygaeidae	***Spilostethus saxatilis***	Austria	Hainburg, Hundsheimer Berge	48,1268	16,9370
BFB_Heteroptera_Schmolke_0642	**KM022648**	Lygaeidae	***Spilostethus saxatilis***	Bavaria	Munich, Rangierbahnhof	48,1906	11,5269
BFB_Heteroptera_Schmolke_0643	**KM021987**	Lygaeidae	***Spilostethus saxatilis***	Bavaria	Maising	47,9828	11,2883
EUBUG_726_f_Spilsaxa1	**KM022349**	Lygaeidae	***Spilostethus saxatilis***	Rhineland Palatinate	Fischbach bei Dahn	49,0860	7,7230
BFB_Heteroptera_Schmolke_0644	**KM023017**	Lygaeidae	***Tropidothorax leucopterus***	Bavaria	Munich, Allacher Heide	48,2008	11,4828
BFB_Heteroptera_Schmolke_0645	**KM022044**	Lygaeidae	***Tropidothorax leucopterus***	Bavaria	Ascholding	47,8958	11,5050
BC ZSM AQU 00706	**HQ563135**	Mesoveliidae	***Mesovelia furcata***	Bavaria	Rosenheim	47,8960	12,1660
BCZSMAQU001091	**KM023061**	Mesoveliidae	***Mesovelia furcata***	Bavaria	Dingolfing-Landau	48,7300	12,7500
EUBUG_587_f_Lorieleg7	**KM022867**	Microphysidae	***Loricula elegantula***	Baden-Wuerttemberg	Pfrunger Ried	47,8990	9,3900
EUBUG_387_f_Acetcari2	**KM023125**	Miridae	***Acetropis carinata***	Thuringia	Hainich-Duen	51,2070	10,7540
EUBUG_Seq27_f_Acetcari	**KM022681**	Miridae	***Acetropis carinata***	Thuringia	Hainich-Duen	51,2069	10,7535
BFB_Heteroptera_Schmolke_130	**KM022753**	Miridae	***Adelphocoris detritus***	Bavaria	Lenggries	47,5950	11,5536
BFB_Heteroptera_Kuechler_0246	**KM022151**	Miridae	***Adelphocoris lineolatus***	Bavaria	Bayreuth, Botanic Garden	49,9230	11,5850
BFB_Heteroptera_Kuechler_0252	**KM021911**	Miridae	***Adelphocoris lineolatus***	Bavaria	Bayreuth, Botanic Garden	49,9230	11,5850
BFB_Heteroptera_Schmolke_0531	**KM023062**	Miridae	***Adelphocoris lineolatus***	Brandenburg	Berlin, Tegel	52,5874	13,2842
BFB_Heteroptera_Schmolke_131	**KM021797**	Miridae	***Adelphocoris lineolatus***	Bavaria	Munich	48,2008	11,4828
BFB_Heteroptera_Schmolke_132	**KM022533**	Miridae	***Adelphocoris lineolatus***	Bavaria	Lenggries	47,5950	11,5536
BFB_Heteroptera_Schmolke_133	**KM021750**	Miridae	***Adelphocoris lineolatus***	Bavaria	Siegenburg	48,7589	11,8383
BFB_Heteroptera_Schmolke_134	**KM021772**	Miridae	***Adelphocoris lineolatus***	Bavaria	Pollanten	49,1511	11,4433
EUBUG_237_m_Adelline1	**KM022905**	Miridae	***Adelphocoris lineolatus***	Thuringia	Hainich-Duen	51,2140	10,3870
EUBUG_251_f_Adelline2	**KM022904**	Miridae	***Adelphocoris lineolatus***	Brandenburg	Schorfheide-Chorin	53,1350	13,8760
EUBUG_278_f_Adelline3	**KM021576**	Miridae	***Adelphocoris lineolatus***	Thuringia	Hainich-Duen	51,2240	10,3810

EUBUG_756_f_Adelline4	**KM021630**	Miridae	***Adelphocoris lineolatus***	Rhineland Palatinate	Fischbach bei Dahn	49,1040	7,6830
BFB_Heteroptera_Schmolke_135	**KM022500**	Miridae	***Adelphocoris quadripunctatus***	Bavaria	Spalt	49,1731	10,9847
BFB_Heteroptera_Schmolke_136	**KM021860**	Miridae	***Adelphocoris quadripunctatus***	Bavaria	Munich	48,1914	11,4847
BFB_Heteroptera_Schmolke_137	**KM022262**	Miridae	***Adelphocoris quadripunctatus***	Bavaria	Munich	48,2114	11,6083
BFB_Heteroptera_Schmolke_138	**KM023036**	Miridae	***Adelphocoris quadripunctatus***	Bavaria	Uebersee	47,8381	12,4778
EUBUG_458_m_Adelquad1	**KM021535**	Miridae	***Adelphocoris quadripunctatus***	Brandenburg	Schorfheide-Chorin	53,1070	14,0000
EUBUG_676_juv_Panttuni4	**KM022582**	Miridae	***Adelphocoris quadripunctatus***	Baden-Wuerttemberg	Pfrunger Ried	47,8990	9,3900
EUBUG_700_m_Adelquad3	**KM022246**	Miridae	***Adelphocoris quadripunctatus***	Baden-Wuerttemberg	Pfrunger Ried	47,8990	9,3900
BFB_Heteroptera_Schmolke_139	**KM021671**	Miridae	***Adelphocoris reichelii***	Bavaria	Leinburg	49,4425	11,2822
BFB_Heteroptera_Schmolke_140	**KM022302**	Miridae	***Adelphocoris reichelii***	Bavaria	Roth	49,1669	11,1783
BFB_Heteroptera_Kuechler_0250	**KM022457**	Miridae	***Adelphocoris seticornis***	Bavaria	Bayreuth, Botanic Garden	49,9230	11,5850
BFB_Heteroptera_Kuechler_0251	**KM021574**	Miridae	***Adelphocoris seticornis***	Bavaria	Bayreuth, Botanic Garden	49,9230	11,5850
BFB_Heteroptera_Schmolke_143	**KM022015**	Miridae	***Adelphocoris seticornis***	Bavaria	Maising	47,9828	11,2883
BFB_Heteroptera_Schmolke_144	**KM022459**	Miridae	***Adelphocoris seticornis***	Bavaria	Munich	48,1914	11,4847
EUBUG_907_f_Adelseti2	**KM022534**	Miridae	***Adelphocoris seticornis***	Thuringia	Jena	50,9510	11,6240
EUBUG_913_m_Adelseti3	**KM021756**	Miridae	***Adelphocoris seticornis***	Thuringia	Jena	50,9510	11,6240
EUBUG_929_m_Adelseti4	**KM021543**	Miridae	***Adelphocoris seticornis***	Thuringia	Jena	50,9510	11,6240
EUBUG_930_m_Adelseti5	**KM022866**	Miridae	***Adelphocoris seticornis***	Thuringia	Jena	50,9510	11,6240
EUBUG_931_m_Adelseti6	**KM021819**	Miridae	***Adelphocoris seticornis***	Thuringia	Jena	50,9510	11,6240
EUBUG_335_m_Agnorecl5	**KM022632**	Miridae	***Agnocoris reclairei***	Baden-Wuerttemberg	Fronreute	47,8390	9,6060
EUBUG_336_f_Agnorecl6	**KM022505**	Miridae	***Agnocoris reclairei***	Baden-Wuerttemberg	Fronreute	47,8510	9,6040
EUBUG_550_m_Agnorubi1	**KM022251**	Miridae	***Agnocoris rubicundus***	Baden-Wuerttemberg	Pfrunger Ried	47,8990	9,3900
EUBUG_559_f_Agnorubi2	**KM023042**	Miridae	***Agnocoris rubicundus***	Baden-Wuerttemberg	Pfrunger Ried	47,8990	9,3900
EUBUG_566_m_Agnorubi3	**KM022803**	Miridae	***Agnocoris rubicundus***	Baden-Wuerttemberg	Pfrunger Ried	47,8990	9,3900
EUBUG_724_m_Agnorubi4	**KM022173**	Miridae	***Agnocoris rubicundus***	Baden-Wuerttemberg	Pfrunger Ried	47,8990	9,3900
BFB_Heteroptera_Schmolke_112	**KM022037**	Miridae	***Alloeotomus germanicus***	Bavaria	Obereichstaett	48,8978	11,1228
BFB_Heteroptera_Kuechler_0161	**KM021654**	Miridae	***Amblytylus nasutus***	Bavaria	Bayreuth, Bindlacher Berg	50,0070	11,6140
BFB_Heteroptera_Kuechler_0207	**KM022931**	Miridae	***Amblytylus nasutus***	Bavaria	Bayreuth, Botanic Garden	49,9230	11,5850
BFB_Heteroptera_Kuechler_0208	**KM021460**	Miridae	***Amblytylus nasutus***	Bavaria	Bayreuth, Botanic Garden	49,9230	11,5850
BFB_Heteroptera_Schmolke_0283	**KM022484**	Miridae	***Amblytylus nasutus***	Bavaria	Erlangen	49,5947	10,9836
BFB_Heteroptera_Schmolke_0284	**KM023133**	Miridae	***Amblytylus nasutus***	Bavaria	Pollanten	49,1511	11,4433
BFB_Heteroptera_Schmolke_0286	**KM022572**	Miridae	***Amblytylus nasutus***	Bavaria	Siegenburg	48,7589	11,8383
EUBUG_197_m_Amblnasu1	**KM021616**	Miridae	***Amblytylus nasutus***	Thuringia	Hainich-Duen	51,0340	10,4590

EUBUG_198_f_Amblnasu2	**KM021901**	Miridae	***Amblytylus nasutus***	Thuringia	Hainich-Duen	51,0340	10,4590
EUBUG_552_f_Apollimb1	**KM022413**	Miridae	***Apolygus limbatus***	Baden-Wuerttemberg	Pfrunger Ried	47,8990	9,3900
EUBUG_553_f_Apollimb2	**KM023057**	Miridae	***Apolygus limbatus***	Baden-Wuerttemberg	Pfrunger Ried	47,8990	9,3900
EUBUG_564_f_Apollimb3	**KM021473**	Miridae	***Apolygus limbatus***	Baden-Wuerttemberg	Pfrunger Ried	47,8990	9,3900
EUBUG_709_f_Apollimb4	**KM022424**	Miridae	***Apolygus limbatus***	Baden-Wuerttemberg	Pfrunger Ried	47,8990	9,3900
EUBUG_714_f_Neolviri2	**KM022713**	Miridae	***Apolygus limbatus***	Baden-Wuerttemberg	Pfrunger Ried	47,8990	9,3900
BFB_Heteroptera_Schmolke_0374	**KM022912**	Miridae	***Apolygus lucorum***	Bavaria	Martinsried	48,1122	11,4622
EUBUG_679_m_Apolrham1	**KM022842**	Miridae	***Apolygus rhamnicola***	Baden-Wuerttemberg	Pfrunger Ried	47,8990	9,3900
BFB_Heteroptera_Kuechler_0266	**KM021431**	Miridae	***Atractotomus magnicornis***	Bavaria	Paradiestal bei Treunitz	49,9830	11,1890
BFB_Heteroptera_Kuechler_0315	**KM021650**	Miridae	***Atractotomus magnicornis***	Rhineland Palatinate	Fischbach bei Dahn	49,1290	7,6900
BFB_Heteroptera_Schmolke_0287	**KM022669**	Miridae	***Atractotomus magnicornis***	Bavaria	Dietramszell	47,8550	11,6056
BFB_Heteroptera_Schmolke_0289	**KM022495**	Miridae	***Atractotomus magnicornis***	Bavaria	Kelheimwinzer	48,9175	11,9178
BFB_Heteroptera_Schmolke_0290	**KM022741**	Miridae	***Atractotomus magnicornis***	Bavaria	Munich	48,1808	11,5244
EUBUG_1003_f_Atramagn8	**KM022161**	Miridae	***Atractotomus magnicornis***	Bavaria	Bavarian Forest, Riedelhuette	48,9210	13,4450
EUBUG_998_f_Atramagn6	**KM021628**	Miridae	***Atractotomus magnicornis***	Bavaria	Bavarian Forest, Rainer Wald	48,9110	12,4510
EUBUG_999_m_Atramagn7	**KM022685**	Miridae	***Atractotomus magnicornis***	Bavaria	Bavarian Forest, Rainer Wald	48,9110	12,4510
BFB_Heteroptera_Kuechler_0202	**KM022756**	Miridae	***Atractotomus mali***	Bavaria	Bayreuth, Botanic Garden	49,9230	11,5850
BFB_Heteroptera_Kuechler_0203	**KM022017**	Miridae	***Atractotomus mali***	Bavaria	Bayreuth, Botanic Garden	49,9230	11,5850
BFB_Heteroptera_Kuechler_0360	**KM021752**	Miridae	***Blepharidopterus angulatus***	Bavaria	Bayreuth, Schlehenmuehle	49,9050	11,6240
BFB_Heteroptera_Schmolke_184	**KM022266**	Miridae	***Blepharidopterus angulatus***	Bavaria	Kallmuenz	49,1367	11,9481
BFB_Heteroptera_Schmolke_185	**KM023039**	Miridae	***Blepharidopterus angulatus***	Bavaria	Erlangen	49,5947	10,9836
BFB_Heteroptera_Schmolke_186	**KM021838**	Miridae	***Blepharidopterus angulatus***	Bavaria	Munich	48,1672	11,4919
BFB_Heteroptera_Schmolke_187	**KM022423**	Miridae	***Blepharidopterus angulatus***	Bavaria	Siegenburg	48,7589	11,8383
EUBUG_1187_m_Bothpilo1	**KM022172**	Miridae	***Bothynotus pilosus***	Bavaria	Bavarian Forest	49,0580	13,2910
EUBUG_1188_m_Bothpilo2	**KM022815**	Miridae	***Bothynotus pilosus***	Bavaria	Bavarian Forest	49,0580	13,2910
EUBUG_1189_m_Bothpilo3	**KM021923**	Miridae	***Bothynotus pilosus***	Bavaria	Bavarian Forest	49,0580	13,2910
EUBUG_1190_m_Bothpilo4	**KM022223**	Miridae	***Bothynotus pilosus***	Bavaria	Bavarian Forest	49,0580	13,2910
EUBUG_1191_m_Bothpilo5	**KM022915**	Miridae	***Bothynotus pilosus***	Bavaria	Bavarian Forest	49,0580	13,2910
BFB_Heteroptera_Schmolke_188	**KM022357**	Miridae	***Brachynotocoris puncticornis***	Bavaria	Munich	48,2269	11,5444
BFB_Heteroptera_Schmolke_088	**KM022378**	Miridae	***Bryocoris pteridis***	Bavaria	Grafenau	48,9264	13,4700
EUBUG_595_f_ Bryopter1	**KM022862**	Miridae	***Bryocoris pteridis***	Baden-Wuerttemberg	Pfrunger Ried	47,8990	9,3900
EUBUG_596_f_ Bryopter2	**KM022494**	Miridae	***Bryocoris pteridis***	Baden-Wuerttemberg	Pfrunger Ried	47,8990	9,3900
EUBUG_597_f_ Bryopter3	**KM022286**	Miridae	***Bryocoris pteridis***	Baden-Wuerttemberg	Pfrunger Ried	47,8990	9,3900

EUBUG_598_f_ Bryopter4	**KM022526**	Miridae	***Bryocoris pteridis***	Baden-Wuerttemberg	Pfrunger Ried	47,8990	9,3900
EUBUG_599_f_ Bryopter5	**KM022421**	Miridae	***Bryocoris pteridis***	Baden-Wuerttemberg	Pfrunger Ried	47,8990	9,3900
BFB_Heteroptera_Kuechler_0268	**KM022475**	Miridae	***Calocoris affinis***	Bavaria	Paradiestal bei Treunitz	49,9830	11,1890
BFB_Heteroptera_Kuechler_0269	**KM021802**	Miridae	***Calocoris affinis***	Bavaria	Paradiestal bei Treunitz	49,9830	11,1890
EUBUG_1338_m_Caloaffi1	**KM021626**	Miridae	***Calocoris affinis***	Thuringia	Hainich-Duen	51,2430	10,3130
BFB_Heteroptera_Kuechler_0150	**KM022449**	Miridae	***Calocoris alpestris***	Bavaria	Wunsiedel, Kleinwendern	50,0000	12,0190
EUBUG_290_f_Calorose1	**KM021722**	Miridae	***Calocoris roseomaculatus***	Baden-Wuerttemberg	Swabian Alb	48,3910	9,3770
EUBUG_1346_f_Campvirg8	**KM022436**	Miridae	***Campyloneura virgula***	Thuringia	Hainich-Duen	51,3560	10,5170
BFB_Heteroptera_Kuechler_0111	**KM022137**	Miridae	***Capsodes gothicus***	Bavaria	Bayreuth, Neubuerg	49,8920	11,4020
BFB_Heteroptera_Kuechler_0127	**KM022799**	Miridae	***Capsodes gothicus***	Bavaria	Bayreuth, Hohenmirsberger-Platte	49,8150	11,4450
BFB_Heteroptera_Kuechler_0128	**KM022243**	Miridae	***Capsodes gothicus***	Bavaria	Bayreuth, Hohenmirsberger-Platte	49,8150	11,4450
BFB_Heteroptera_Schmolke_0381	**KM023014**	Miridae	***Capsodes gothicus***	Bavaria	Pollanten	49,1511	11,4433
EUBUG_1129_m_Capswagn1	**KM023005**	Miridae	***Capsus wagneri***	Bavaria	Bavarian Forest, Isar estuary	48,7780	12,9980
EUBUG_1130_m_Capswagn2	**KM022971**	Miridae	***Capsus wagneri***	Bavaria	Bavarian Forest, Isar estuary	48,7780	12,9980
BFB_Heteroptera_Kuechler_0093	**KM022684**	Miridae	***Charagochilus gyllenhalii***	Bavaria	Bayreuth, Botanic Garden	49,9260	11,5830
EUBUG_442_m_Chargyll2	**KM022849**	Miridae	***Charagochilus gyllenhalii***	Baden-Wuerttemberg	Swabian Alb	48,4590	9,4600
EUBUG_532_f_Chargyll3	**KM021743**	Miridae	***Charagochilus gyllenhalii***	Baden-Wuerttemberg	Pfrunger Ried	47,8990	9,3900
BFB_Heteroptera_Kuechler_0303	**KM021607**	Miridae	***Charagochilus spiralifer***	Rhineland Palatinate	Fischbach bei Dahn	49,0910	7,7150
EUBUG_1137_f_Chargyll6	**KM022156**	Miridae	***Charagochilus spiralifer***	Bavaria	Bavarian Forest, Isar estuary	48,7780	12,9980
EUBUG_1138_f_Chargyll7	**KM022062**	Miridae	***Charagochilus spiralifer***	Bavaria	Bavarian Forest, Isar estuary	48,7780	12,9980
EUBUG_1404_f_Charspir3	**KM021492**	Miridae	***Charagochilus spiralifer***	Rhineland Palatinate	Fischbach bei Dahn	49,0910	7,6780
EUBUG_1405_m_Charspir4	**KM022465**	Miridae	***Charagochilus spiralifer***	Rhineland Palatinate	Fischbach bei Dahn	49,0910	7,6780
EUBUG_1406_m_Charspir5	**KM022219**	Miridae	***Charagochilus spiralifer***	Rhineland Palatinate	Fischbach bei Dahn	49,0910	7,6780
EUBUG_569_m_Charspir1	**KM021530**	Miridae	***Charagochilus spiralifer***	Rhineland Palatinate	Fischbach bei Dahn	49,0910	7,6780
EUBUG_570_f_Charspir2	**KM021450**	Miridae	***Charagochilus spiralifer***	Rhineland Palatinate	Fischbach bei Dahn	49,0910	7,6780
EUBUG_694_f_Chargyll4	**KM022455**	Miridae	***Charagochilus spiralifer***	Baden-Wuerttemberg	Pfrunger Ried	47,8990	9,3900
EUBUG_695_f_Chargyll5	**KM022890**	Miridae	***Charagochilus spiralifer***	Baden-Wuerttemberg	Pfrunger Ried	47,8990	9,3900
EUBUG_1329_m_Chargyll8	**KM021525**	Miridae	***Charagochilus weberi***	Thuringia	Hainich-Duen	51,3370	10,3590
EUBUG_1397_m_Charwebe1	**KM022952**	Miridae	***Charagochilus weberi***	Swiss	Gampel	46,3170	7,7330
EUBUG_1398_m_Charwebe2	**KM021434**	Miridae	***Charagochilus weberi***	Swiss	Gampel	46,3170	7,7330
EUBUG_1399_m_Charwebe3	**KM022216**	Miridae	***Charagochilus weberi***	Swiss	Gampel	46,3170	7,7330
EUBUG_1400_f_Charwebe4	**KM021903**	Miridae	***Charagochilus weberi***	Swiss	Gampel	46,3170	7,7330
EUBUG_1401_f_Charwebe5	**KM023019**	Miridae	***Charagochilus weberi***	Swiss	Gampel	46,3170	7,7330

BFB_Heteroptera_Kuechler_0091	**KM021442**	Miridae	***Chlamydatus evanescens***	Bavaria	Bayreuth, University	49,9290	11,5830
BFB_Heteroptera_Schmolke_0540	**KM022549**	Miridae	***Chlamydatus evanescens***	Brandenburg	Berlin, Tegel	52,5874	13,2842
BFB_Heteroptera_Kuechler_0137	**KM021930**	Miridae	***Chlamydatus pulicarius***	Bavaria	Bayreuth, Hohenmirsberger-Platte	49,8150	11,4450
BFB_Heteroptera_Kuechler_0138	**KM023129**	Miridae	***Chlamydatus pulicarius***	Bavaria	Bayreuth, Hohenmirsberger-Platte	49,8150	11,4450
BFB_Heteroptera_Schmolke_0299	**KM022901**	Miridae	***Chlamydatus pulicarius***	Bavaria	Munich	48,2008	11,4828
BFB_Heteroptera_Schmolke_0301	**KM021812**	Miridae	***Chlamydatus pulicarius***	Bavaria	Siegenburg	48,7589	11,8383
EUBUG_249_m_Chlapull2	**KM023043**	Miridae	***Chlamydatus pulicarius***	Brandenburg	Schorfheide-Chorin	53,1350	13,8760
EUBUG_972_f_Chlapuli1	**KM022517**	Miridae	***Chlamydatus pulicarius***	Rhineland Palatinate	Fischbach bei Dahn	49,0900	7,7150
EUBUG_973_m_Chlapuli2	**KM022924**	Miridae	***Chlamydatus pulicarius***	Rhineland Palatinate	Fischbach bei Dahn	49,0900	7,7150
EUBUG_248_m_Chlapull1	**KM021572**	Miridae	***Chlamydatus pullus***	Brandenburg	Schorfheide-Chorin	53,1350	13,8760
EUBUG_975_m_Chlapull4	**KM022995**	Miridae	***Chlamydatus pullus***	Thuringia	Jena	50,9510	11,6240
EUBUG_976_f_Chlapull5	**KM022761**	Miridae	***Chlamydatus pullus***	Thuringia	Jena	50,9510	11,6240
EUBUG_977_m_Chlapull6	**KM022231**	Miridae	***Chlamydatus pullus***	Thuringia	Jena	50,9510	11,6240
EUBUG_978_m_Chlapull7	**KM021697**	Miridae	***Chlamydatus pullus***	Thuringia	Jena	50,9510	11,6240
EUBUG_Seq25_m_Chlampull	**KM022217**	Miridae	***Chlamydatus pullus***	Brandenburg	Schorfheide-Chorin	53,1349	13,8759
BFB_Heteroptera_Kuechler_0135	**KM022401**	Miridae	***Chlamydatus saltitans***	Bavaria	Bayreuth, Hohenmirsberger-Platte	49,8150	11,4450
BFB_Heteroptera_Kuechler_0136	**KM022399**	Miridae	***Chlamydatus saltitans***	Bavaria	Bayreuth, Hohenmirsberger-Platte	49,8150	11,4450
BFB_Heteroptera_Kuechler_0125	**KM021887**	Miridae	***Closterotomus biclavatus***	Bavaria	Bayreuth, Hohenmirsberger-Platte	49,8150	11,4450
BFB_Heteroptera_Kuechler_0155	**KM022043**	Miridae	***Closterotomus biclavatus***	Bavaria	Bayreuth, Bindlacher Berg	50,0070	11,6140
EUBUG_1168_f_Closbicl5	**KM022679**	Miridae	***Closterotomus biclavatus***	Bavaria	Bavarian Forest, Jochenstein	48,5170	13,7250
EUBUG_1219_f_Closbicl6	**KM022305**	Miridae	***Closterotomus biclavatus***	Bavaria	Bavarian Forest, Solla	48,8200	13,3050
EUBUG_1339_f_Closbicl7	**KM023034**	Miridae	***Closterotomus biclavatus***	Thuringia	Hainich-Duen	51,2050	10,3900
EUBUG_794_m_Closbicl4	**KM022448**	Miridae	***Closterotomus biclavatus***	Baden-Wuerttemberg	Fronreute	47,8990	9,3900
BFB_Heteroptera_Kuechler_0123	**KM021661**	Miridae	***Closterotomus fulvomaculatus***	Bavaria	Bayreuth, Hohenmirsberger-Platte	49,8150	11,4450
BFB_Heteroptera_Kuechler_0124	**KM022204**	Miridae	***Closterotomus fulvomaculatus***	Bavaria	Bayreuth, Hohenmirsberger-Platte	49,8150	11,4450
EUBUG_1131_m_Closfulv2	**KM022452**	Miridae	***Closterotomus fulvomaculatus***	Bavaria	Bavarian Forest, Isar estuary	48,7780	12,9980
EUBUG_1132_f_Closfulv3	**KM022003**	Miridae	***Closterotomus fulvomaculatus***	Bavaria	Bavarian Forest, Isar estuary	48,7780	12,9980
EUBUG_492_f_Closnorw1	**KM023139**	Miridae	***Closterotomus norvegicus***	Bavaria	Bavarian Forest, Koetzting	49,1820	12,8443
EUBUG_493_f_Closnorw2	**KM022296**	Miridae	***Closterotomus norvegicus***	Bavaria	Bavarian Forest, Koetzting	49,1820	12,8443
EUBUG_494_f_Closnorw3	**KM022083**	Miridae	***Closterotomus norvegicus***	Bavaria	Bavarian Forest, Koetzting	49,1820	12,8443
EUBUG_495_f_Closnorw4	**KM023092**	Miridae	***Closterotomus norvegicus***	Bavaria	Bavarian Forest, Koetzting	49,1820	12,8443
BFB_Heteroptera_Schmolke_0306	**KM022187**	Miridae	***Compsidolon salicellum***	Bavaria	Munich	48,2008	11,4828
BFB_Heteroptera_Schmolke_0307	**KM022950**	Miridae	***Compsidolon salicellum***	Bavaria	Erlangen	49,5861	11,0308

BFB_Heteroptera_Schmolke_0308	**KM021455**	Miridae	***Compsidolon salicellum***	Bavaria	Martinsried	48,1122	11,4622
BFB_Heteroptera_Schmolke_0273	**KM022993**	Miridae	***Cremnocephalus albolineatus***	Bavaria	Munich	48,2114	11,6083
BFB_Heteroptera_Schmolke_0274	**KM022886**	Miridae	***Cremnocephalus alpestris***	Bavaria	Grafenau	48,9050	13,4658
BFB_Heteroptera_Schmolke_0275	**KM021615**	Miridae	***Cremnocephalus alpestris***	Bavaria	Munich	48,0933	11,6683
BFB_Heteroptera_Schmolke_0276	**KM021787**	Miridae	***Cremnocephalus alpestris***	Bavaria	Drachselsried	49,1294	13,0678
BFB_Heteroptera_Schmolke_0277	**KM021945**	Miridae	***Cremnocephalus alpestris***	Bavaria	Munich	48,0961	11,5514
EUBUG_1217_f_Cremalpe6	**KM022792**	Miridae	***Cremnocephalus alpestris***	Bavaria	Bavarian Forest, Solla	48,8200	13,3050
EUBUG_1218_f_Cremalpe7	**KM023119**	Miridae	***Cremnocephalus alpestris***	Bavaria	Bavarian Forest, Solla	48,8200	13,3050
BFB_Heteroptera_Schmolke_0309	**KM021602**	Miridae	***Criocoris crassicornis***	Bavaria	Munich	48,2008	11,4828
EUBUG_541_f_Criocras1	**KM022784**	Miridae	***Criocoris crassicornis***	Baden-Wuerttemberg	Pfrunger Ried	47,8990	9,3900
EUBUG_542_m_Criocras2	**KM021844**	Miridae	***Criocoris crassicornis***	Baden-Wuerttemberg	Pfrunger Ried	47,8990	9,3900
EUBUG_543_m_Criocras3	**KM022328**	Miridae	***Criocoris crassicornis***	Baden-Wuerttemberg	Pfrunger Ried	47,8990	9,3900
EUBUG_544_m_Criocras4	**KM022115**	Miridae	***Criocoris crassicornis***	Baden-Wuerttemberg	Pfrunger Ried	47,8990	9,3900
EUBUG_545_m_Criocras5	**KM021748**	Miridae	***Criocoris crassicornis***	Baden-Wuerttemberg	Pfrunger Ried	47,8990	9,3900
BFB_Heteroptera_Schmolke_190	**KM021764**	Miridae	***Cyllecoris histrionius***	Bavaria	Treuchtlingen	48,9672	10,9214
EUBUG_Seq31_f_Deraannu	**KM021884**	Miridae	***Deraeocoris annulipes***	Brandenburg	Schorfheide-Chorin	53,0893	13,9800
BFB_Heteroptera_Kuechler_0200	**KM022960**	Miridae	***Deraeocoris flavilinea***	Bavaria	Bayreuth, Botanic Garden	49,9230	11,5850
BFB_Heteroptera_Kuechler_0201	**KM022089**	Miridae	***Deraeocoris flavilinea***	Bavaria	Bayreuth, Botanic Garden	49,9230	11,5850
BFB_Heteroptera_Schmolke_113	**KM022164**	Miridae	***Deraeocoris flavilinea***	Bavaria	Erlangen	49,5947	10,9836
BFB_Heteroptera_Schmolke_114	**KM022817**	Miridae	***Deraeocoris flavilinea***	Bavaria	Munich	48,1672	11,4919
BFB_Heteroptera_Schmolke_115	**KM022998**	Miridae	***Deraeocoris flavilinea***	Bavaria	Volkach	49,8547	10,2086
BFB_Heteroptera_Schmolke_116	**KM022579**	Miridae	***Deraeocoris flavilinea***	Bavaria	Munich	48,2114	11,6083
BFB_Heteroptera_Kuechler_0253	**KM023072**	Miridae	***Deraeocoris lutescens***	Bavaria	Bayreuth, Botanic Garden	49,9230	11,5850
BFB_Heteroptera_Kuechler_0254	**KM022930**	Miridae	***Deraeocoris lutescens***	Bavaria	Bayreuth, Botanic Garden	49,9230	11,5850
BFB_Heteroptera_Schmolke_126	**KM023051**	Miridae	***Deraeocoris lutescens***	Bavaria	Munich	48,1808	11,5244
BFB_Heteroptera_Schmolke_127	**KM022687**	Miridae	***Deraeocoris lutescens***	Bavaria	Schoengeising	48,1483	11,1958
BFB_Heteroptera_Schmolke_128	**KM022064**	Miridae	***Deraeocoris lutescens***	Bavaria	Munich	48,2269	11,5444
BFB_Heteroptera_Schmolke_129	**KM022024**	Miridae	***Deraeocoris lutescens***	Bavaria	Uebersee	47,8381	12,4778
BFB_Heteroptera_Schmolke_117	**KM022959**	Miridae	***Deraeocoris morio***	Bavaria	Koenigsbrunn	48,2714	10,9167
BFB_Heteroptera_Kuechler_0210	**KM021962**	Miridae	***Deraeocoris olivaceus***	Bavaria	Hasberge, Vorbach	50,1050	10,7190
BFB_Heteroptera_Schmolke_118	**KM021821**	Miridae	***Deraeocoris olivaceus***	Bavaria	Karlstadt	49,9842	9,7069
BFB_Heteroptera_Schmolke_119	**KM022154**	Miridae	***Deraeocoris olivaceus***	Bavaria	Straubing	48,9019	12,6675
EUBUG_1403_f_Deraoliv2	**KM021727**	Miridae	***Deraeocoris olivaceus***	Thuringia	Hainich-Duen	51,0930	10,4660

BFB_Heteroptera_Kuechler_0235	**KM022165**	Miridae	***Deraeocoris ruber***	Bavaria	Bayreuth, Botanic Garden	49,9230	11,5850
BFB_Heteroptera_Kuechler_0255	**KM021872**	Miridae	***Deraeocoris ruber***	Bavaria	Bayreuth, Botanic Garden	49,9230	11,5850
BFB_Heteroptera_Schmolke_122	**KM023112**	Miridae	***Deraeocoris ruber***	Bavaria	Munich	48,2114	11,6083
BFB_Heteroptera_Schmolke_123	**KM021815**	Miridae	***Deraeocoris ruber***	Bavaria	Munich	48,1906	11,5269
BFB_Heteroptera_Schmolke_124	**KM022701**	Miridae	***Deraeocoris ruber***	Bavaria	Uebersee	47,8381	12,4778
BFB_Heteroptera_Schmolke_125	**KM022027**	Miridae	***Deraeocoris ruber***	Bavaria	Munich	48,1914	11,4847
EUBUG_404_f_Derarube1	**KM022355**	Miridae	***Deraeocoris ruber***	Thuringia	Hainich-Duen	51,0230	10,3220
EUBUG_417_f_Derarube2	**KM021529**	Miridae	***Deraeocoris ruber***	Thuringia	Hainich-Duen	51,2150	10,3910
EUBUG_693_f_Derarube3	**KM023110**	Miridae	***Deraeocoris ruber***	Baden-Wuerttemberg	Pfrunger Ried	47,8990	9,3900
BFB_Heteroptera_Kuechler_0211	**KM023064**	Miridae	***Deraeocoris trifasciatus***	Bavaria	Hasberge, Vorbach	50,1050	10,7190
BFB_Heteroptera_Kuechler_0196	**KM021624**	Miridae	***Dichrooscytus gustavi***	Bavaria	Bayreuth, Botanic Garden	49,9230	11,5850
EUBUG_1159_m_Dichinte2	**KM021786**	Miridae	***Dichrooscytus intermedius***	Bavaria	Bavarian Forest, Riedelhuette	48,9210	13,4125
EUBUG_1192_m_Dichinte3	**KM022309**	Miridae	***Dichrooscytus intermedius***	Bavaria	Bavarian Forest, National Park	49,0580	13,2910
EUBUG_1194_m_Dichinte4	**KM022458**	Miridae	***Dichrooscytus intermedius***	Bavaria	Bavarian Forest, Riedelhuette	48,9210	13,4450
BFB_Heteroptera_Kuechler_0334	**KM021564**	Miridae	***Dicyphus annulatus***	France	La Garde-Freinet	43,3310	6,4740
BFB_Heteroptera_Schmolke_094	**KM022672**	Miridae	***Dicyphus annulatus***	Bavaria	Munich	48,1906	11,5269
BFB_Heteroptera_Schmolke_095	**KM022249**	Miridae	***Dicyphus annulatus***	Bavaria	Munich	48,1914	11,4847
BFB_Heteroptera_Schmolke_096	**KM022605**	Miridae	***Dicyphus annulatus***	Bavaria	Kallmuenz	49,1842	11,9450
BFB_Heteroptera_Kuechler_0307	**KM022092**	Miridae	***Dicyphus epilobii***	Rhineland Palatinate	Fischbach bei Dahn	49,1290	7,6900
BFB_Heteroptera_Schmolke_097	**KM022434**	Miridae	***Dicyphus epilobii***	Bavaria	Munich	48,1906	11,5269
BFB_Heteroptera_Kuechler_0145	**KM022796**	Miridae	***Dicyphus errans***	Bavaria	Bayreuth, Botanic Garden	49,9230	11,5870
BFB_Heteroptera_Kuechler_0184	**KM022157**	Miridae	***Dicyphus errans***	Bavaria	Bayreuth, Botanic Garden	49,9230	11,5870
BFB_Heteroptera_Kuechler_0185	**KM021759**	Miridae	***Dicyphus errans***	Bavaria	Bayreuth, Botanic Garden	49,9230	11,5870
BFB_Heteroptera_Kuechler_0271	**KM021796**	Miridae	***Dicyphus errans***	Bavaria	Bayreuth, University	49,9290	11,5830
BFB_Heteroptera_Schmolke_098	**KM021731**	Miridae	***Dicyphus errans***	Bavaria	Munich	48,1906	11,5269
BFB_Heteroptera_Schmolke_099	**KM021587**	Miridae	***Dicyphus errans***	Bavaria	Munich	48,2114	11,6180
BFB_Heteroptera_Schmolke_100	**KM022250**	Miridae	***Dicyphus errans***	Bavaria	Duernhart	48,7792	11,8392
BFB_Heteroptera_Schmolke_101	**KM023099**	Miridae	***Dicyphus errans***	Bavaria	Munich	48,2008	11,4828
EUBUG_1197_m_Dicyerra1	**KM022469**	Miridae	***Dicyphus errans***	Bavaria	Bavarian Forest, Riedelhuette	48,9210	13,4450
BFB_Heteroptera_Schmolke_102	**KM022917**	Miridae	***Dicyphus globulifer***	Bavaria	Uebersee	47,8381	12,4778
BFB_Heteroptera_Schmolke_103	**KM022415**	Miridae	***Dicyphus globulifer***	Bavaria	Offenstetten	48,8111	11,9106
BFB_Heteroptera_Schmolke_104	**KM022604**	Miridae	***Dicyphus globulifer***	Bavaria	Munich	48,2114	11,6083
BFB_Heteroptera_Schmolke_105	**KM022102**	Miridae	***Dicyphus globulifer***	Bavaria	Erlangen	49,5861	11,0308

EUBUG_716_f_Dicyglob1	**KM022330**	Miridae	***Dicyphus globulifer***	Baden-Wuerttemberg	Pfrunger Ried	47,8990	9,3900
EUBUG_717_f_Dicyglob2	**KM022909**	Miridae	***Dicyphus globulifer***	Baden-Wuerttemberg	Pfrunger Ried	47,8990	9,3900
EUBUG_718_m_Dicyglob3	**KM021842**	Miridae	***Dicyphus globulifer***	Baden-Wuerttemberg	Pfrunger Ried	47,8990	9,3900
EUBUG_719_m_Dicyglob4	**KM022568**	Miridae	***Dicyphus globulifer***	Baden-Wuerttemberg	Pfrunger Ried	47,8990	9,3900
BFB_Heteroptera_Schmolke_106	**KM022599**	Miridae	***Dicyphus hyalinipennis***	Bavaria	Treuchtlingen	48,9672	10,9214
BFB_Heteroptera_Schmolke_107	**KM021599**	Miridae	***Dicyphus pallidus***	Bavaria	Uebersee	47,8381	12,4778
BFB_Heteroptera_Schmolke_108	**KM023041**	Miridae	***Dicyphus pallidus***	Bavaria	Dietramszell	47,8550	11,6056
BFB_Heteroptera_Schmolke_109	**KM021788**	Miridae	***Dicyphus pallidus***	Bavaria	Pleinfeld	49,1217	11,0003
BFB_Heteroptera_Schmolke_110	**KM022146**	Miridae	***Dicyphus pallidus***	Bavaria	Urfeld	47,6200	11,3428
EUBUG_715_m_Dicypall1	**KM022312**	Miridae	***Dicyphus pallidus***	Baden-Wuerttemberg	Pfrunger Ried	47,8990	9,3900
EUBUG_1205_f_Dryoflav6	**KM022076**	Miridae	***Dryophilocoris flavoquadrimaculatus***	Bavaria	Bavarian Forest, Rainer Wald	48,9150	12,4430
EUBUG_538_f_Euroalpi1	**KM021774**	Miridae	***Europiella alpina***	Baden-Wuerttemberg	Pfrunger Ried	47,8990	9,3900
EUBUG_565_m_Euroalpi2	**KM023073**	Miridae	***Europiella alpina***	Baden-Wuerttemberg	Pfrunger Ried	47,8990	9,3900
BFB_Heteroptera_Schmolke_0319	**KM022409**	Miridae	***Europiella artemisiae***	Bavaria	Munich	48,1914	11,4847
BFB_Heteroptera_Schmolke_0321	**KM022313**	Miridae	***Europiella artemisiae***	Bavaria	Munich	48,1808	11,5244
BFB_Heteroptera_Kuechler_0220	**KM022304**	Miridae	***Globiceps flavomaculatus***	Bavaria	Bayreuth, Botanic Garden	49,9230	11,5850
BFB_Heteroptera_Kuechler_0221	**KM021879**	Miridae	***Globiceps flavomaculatus***	Bavaria	Bayreuth, Botanic Garden	49,9230	11,5850
EUBUG_1193_m_Globflav6	**KM021478**	Miridae	***Globiceps flavomaculatus***	Bavaria	Bavarian Forest, National Park	49,0580	13,2910
BFB_Heteroptera_Kuechler_0192	**KM023003**	Miridae	***Globiceps fulvicollis***	Bavaria	Staffelberg	50,0920	11,0240
BFB_Heteroptera_Kuechler_0248	**KM022006**	Miridae	***Globiceps fulvicollis***	Bavaria	Bayreuth, Botanic Garden	49,9230	11,5850
BFB_Heteroptera_Schmolke_0196	**KM021699**	Miridae	***Globiceps fulvicollis***	Bavaria	Leinburg	49,4425	11,2822
BFB_Heteroptera_Schmolke_0197	**KM021809**	Miridae	***Globiceps fulvicollis***	Bavaria	Siegenburg	48,7589	11,8383
BFB_Heteroptera_Schmolke_0200	**KM022946**	Miridae	***Globiceps fulvicollis***	Bavaria	Leinburg	49,4425	11,2822
BFB_Heteroptera_Schmolke_0203	**KM023056**	Miridae	***Globiceps fulvicollis***	Bavaria	Kallmuenz	49,1842	11,9450
EUBUG_389_m_Globfulv1	**KM022492**	Miridae	***Globiceps fulvicollis***	Thuringia	Hainich-Duen	51,2070	10,7540
EUBUG_390_m_Globfulv2	**KM021979**	Miridae	***Globiceps fulvicollis***	Thuringia	Hainich-Duen	51,2070	10,7540
EUBUG_391_m_Globfulv3	**KM022786**	Miridae	***Globiceps fulvicollis***	Thuringia	Hainich-Duen	51,2070	10,7540
BFB_Heteroptera_Kuechler_0190	**KM021495**	Miridae	***Hadrodemus m-flavum***	Bavaria	Staffelberg	50,0920	11,0240
BFB_Heteroptera_Kuechler_0191	**KM022327**	Miridae	***Hadrodemus m-flavum***	Bavaria	Staffelberg	50,0920	11,0240
EUBUG_448_m_Hadrmfla1	**KM021665**	Miridae	***Hadrodemus m-flavum***	Baden-Wuerttemberg	Swabian Alb	48,4560	9,4570
BFB_Heteroptera_Schmolke_169	**KM021704**	Miridae	***Halticus apterus***	Bavaria	Munich	48,1914	11,4847
EUBUG_274_f_Haltapte3	**KM022412**	Miridae	***Halticus apterus***	Brandenburg	Schorfheide-Chorin	53,1030	13,9860
EUBUG_580_m_Haltapte5	**KM021993**	Miridae	***Halticus apterus***	Baden-Wuerttemberg	Pfrunger Ried	47,8990	9,3900

EUBUG_581_m_Haltapte6	**KM022133**	Miridae	***Halticus apterus***	Baden-Wuerttemberg	Pfrunger Ried	47,8990	9,3900
EUBUG_583_juv_Haltapte8	**KM022456**	Miridae	***Halticus apterus***	Baden-Wuerttemberg	Pfrunger Ried	47,8990	9,3900
BFB_Heteroptera_Schmolke_171	**KM022051**	Miridae	***Halticus luteicollis***	Bavaria	Munich	48,0961	11,5514
EUBUG_1035_f_Haltlute3	**KM022129**	Miridae	***Halticus luteicollis***	Bavaria	Bavarian Forest, Isar estuary	48,7930	12,9700
EUBUG_994_m_Haltlute2	**KM021662**	Miridae	***Halticus luteicollis***	Bavaria	Bavarian Forest, Isar estuary	48,7780	12,9980
BFB_Heteroptera_Schmolke_0389	**KM022125**	Miridae	***Harpocera thoracica***	Bavaria	Diessen	47,9486	11,1142
BFB_Heteroptera_Schmolke_0390	**KM021614**	Miridae	***Harpocera thoracica***	Bavaria	Munich	48,2008	11,4828
BFB_Heteroptera_Schmolke_0391	**KM022972**	Miridae	***Harpocera thoracica***	Bavaria	Munich	48,1672	11,4919
EUBUG_1203_m_Harpthor10	**KM022028**	Miridae	***Harpocera thoracica***	Bavaria	Bavarian Forest, Rainer Wald	48,9150	12,4430
EUBUG_1204_f_Harpthor11	**KM021645**	Miridae	***Harpocera thoracica***	Bavaria	Bavarian Forest, Rainer Wald	48,9150	12,4430
EUBUG_509_f_Harpothor5	**KM021841**	Miridae	***Harpocera thoracica***	Lower Saxony	Wahnbek	53,7940	7,8970
BFB_Heteroptera_Schmolke_0204	**KM022016**	Miridae	***Heterocordylus erythropthalmus***	Bavaria	Munich	48,0933	11,6683
BFB_Heteroptera_Schmolke_0205	**KM022585**	Miridae	***Heterocordylus erythropthalmus***	Bavaria	Kallmuenz	49,1842	11,9450
BFB_Heteroptera_Schmolke_0206	**KM022435**	Miridae	***Heterocordylus erythropthalmus***	Bavaria	Kelheimwinzer	48,9175	11,9178
BFB_Heteroptera_Schmolke_0207	**KM021867**	Miridae	***Heterocordylus genistae***	Bavaria	Koenigsbrunn	48,2714	10,9167
BFB_Heteroptera_Schmolke_0210	**KM022964**	Miridae	***Heterocordylus tibialis***	Bavaria	Elsendorf	48,7164	11,7981
BFB_Heteroptera_Kuechler_0197	**KM021433**	Miridae	***Hoplomachus thunbergii***	Bavaria	Bayreuth, Botanic Garden	49,9230	11,5850
BFB_Heteroptera_Schmolke_0322	**KM022800**	Miridae	***Hoplomachus thunbergii***	Bavaria	Pollanten	49,1511	11,4433
BFB_Heteroptera_Schmolke_0323	**KM021904**	Miridae	***Hoplomachus thunbergii***	Bavaria	Kelheimwinzer	48,9175	11,9178
BFB_Heteroptera_Schmolke_0392	**KM022082**	Miridae	***Hoplomachus thunbergii***	Bavaria	Munich	48,1914	11,4847
BFB_Heteroptera_Schmolke_0393	**KM022171**	Miridae	***Hoplomachus thunbergii***	Bavaria	Uebersee	47,8381	12,4778
EUBUG_270_f_Haplthun1	**KM022464**	Miridae	***Hoplomachus thunbergii***	Baden-Wuerttemberg	Swabian Alb	48,4010	9,4580
EUBUG_271_f_Haplthun2	**KM021569**	Miridae	***Hoplomachus thunbergii***	Baden-Wuerttemberg	Swabian Alb	48,4010	9,4580
EUBUG_272_f_Haplthun3	**KM022790**	Miridae	***Hoplomachus thunbergii***	Baden-Wuerttemberg	Swabian Alb	48,4010	9,4580
BFB_Heteroptera_Schmolke_0382	**KM021465**	Miridae	***Horistus orientalis***	Bavaria	Pollanten	49,1511	11,4433
EUBUG_261_m_Horiorie1	**KM022887**	Miridae	***Horistus orientalis***	Baden-Wuerttemberg	Swabian Alb	48,3980	9,3420
EUBUG_262_f_Horiorie2	**KM022973**	Miridae	***Horistus orientalis***	Baden-Wuerttemberg	Swabian Alb	48,3980	9,3420
EUBUG_263_f_Horiorie3	**KM021505**	Miridae	***Horistus orientalis***	Baden-Wuerttemberg	Swabian Alb	48,3980	9,3420
EUBUG_195_f_Leptdola3	**KM022757**	Miridae	***Leptopterna dolabrata***	Baden-Wuerttemberg	Swabian Alb	48,3810	9,4190
BFB_Heteroptera_Schmolke_0386	**KM022124**	Miridae	***Leptopterna ferrugata***	Bavaria	Pollanten	49,1511	11,4433
EUBUG_381_f_Leptferr1	**KM022245**	Miridae	***Leptopterna ferrugata***	Baden-Wuerttemberg	Swabian Alb	48,4190	9,4800
EUBUG_382_f_Leptferr2	**KM021707**	Miridae	***Leptopterna ferrugata***	Baden-Wuerttemberg	Swabian Alb	48,4190	9,4800
EUBUG_383_f_Leptferr3	**KM022048**	Miridae	***Leptopterna ferrugata***	Baden-Wuerttemberg	Swabian Alb	48,4190	9,4800

BFB_Heteroptera_Kuechler_0076	**KM022502**	Miridae	***Liocoris tripustulatus***	Bavaria	Neunkirchen am Main	49,9240	11,6320
BFB_Heteroptera_Schmolke_0376	**KM022782**	Miridae	***Liocoris tripustulatus***	Bavaria	Siegenburg	48,7589	11,8383
EUBUG_453_m_Lioctrip1	**KM022920**	Miridae	***Liocoris tripustulatus***	Brandenburg	Schorfheide-Chorin	53,1070	14,0000
EUBUG_454_f_Lioctrip2	**KM022535**	Miridae	***Liocoris tripustulatus***	Brandenburg	Schorfheide-Chorin	53,1070	14,0000
EUBUG_701_f_Lioctrip4	**KM022539**	Miridae	***Liocoris tripustulatus***	Baden-Wuerttemberg	Pfrunger Ried	47,8990	9,3900
EUBUG_702_f_Lioctrip5	**KM022888**	Miridae	***Liocoris tripustulatus***	Baden-Wuerttemberg	Pfrunger Ried	47,8990	9,3900
BFB_Heteroptera_Schmolke_0394	**KM022567**	Miridae	***Lopus decolor***	Bavaria	Roth	49,1669	11,1783
BFB_Heteroptera_Schmolke_0397	**KM021651**	Miridae	***Lopus decolor***	Bavaria	Pollanten	49,1511	11,4433
EUBUG_256_m_Lopudeco1	**KM021801**	Miridae	***Lopus decolor***	Thuringia	Hainich-Duen	51,0010	10,4300
EUBUG_257_m_Lopudeco2	**KM021520**	Miridae	***Lopus decolor***	Thuringia	Hainich-Duen	51,0010	10,4300
EUBUG_258_m_Lopudeco3	**KM021454**	Miridae	***Lopus decolor***	Thuringia	Hainich-Duen	51,0010	10,4300
EUBUG_Seq8_m_Lopudeco	**KM021736**	Miridae	***Lopus decolor***	Thuringia	Hainich-Duen	51,0007	10,4299
EUBUG_1336_f_Dichrufi1	**KM022360**	Miridae	***Lygocoris pabulinus***	Thuringia	Hainich-Duen	51,1280	10,3800
EUBUG_144_f_Lygopabu1	**KM022925**	Miridae	***Lygocoris pabulinus***	Baden-Wuerttemberg	Altshausen	47,9340	9,5380
EUBUG_512_m_Lygopabu3	**KM022658**	Miridae	***Lygocoris pabulinus***	Lower Saxony	Rastede	53,2430	8,2040
EUBUG_863_f_Lygopabu5	**KM022481**	Miridae	***Lygocoris pabulinus***	Rhineland Palatinate	Fischbach bei Dahn	49,0860	7,7230
BFB_Heteroptera_Kuechler_0119	**KM023105**	Miridae	***Lygus gemellatus***	Bavaria	Bayreuth, Botanic Garden	49,9230	11,5830
EUBUG_1139_f_Lygugeme2	**KM022922**	Miridae	***Lygus gemellatus***	Baden-Wuerttemberg	Swabian Alb	48,4590	9,4600
BFB_Heteroptera_Kuechler_0120	**KM023049**	Miridae	***Lygus pratensis***	Bavaria	Bayreuth, Botanic Garden	49,9230	11,5830
BFB_Heteroptera_Kuechler_0121	**KM021926**	Miridae	***Lygus pratensis***	Bavaria	Bayreuth, Botanic Garden	49,9230	11,5830
EUBUG_225_m_Lyguprat3	**KM021854**	Miridae	***Lygus pratensis***	Thuringia	Hainich-Duen	51,2140	10,3870
EUBUG_735_m_Lyguprat6	**KM022498**	Miridae	***Lygus pratensis***	Rhineland Palatinate	Fischbach bei Dahn	49,1040	7,6830
EUBUG_736_m_Lyguprat7	**KM022170**	Miridae	***Lygus pratensis***	Rhineland Palatinate	Fischbach bei Dahn	49,1040	7,6830
EUBUG_880_m_Lygugeme1	**KM023020**	Miridae	***Lygus pratensis***	Rhineland Palatinate	Fischbach bei Dahn	49,0900	7,7150
EUBUG_881_m_Lyguprat8	**KM023018**	Miridae	***Lygus pratensis***	Rhineland Palatinate	Fischbach bei Dahn	49,0900	7,7150
BFB_Heteroptera_Kuechler_0005	**KM022988**	Miridae	***Lygus rugulipennis***	Bavaria	Bayreuth, Destuben	49,9020	11,5680
BFB_Heteroptera_Kuechler_0264	**KM022239**	Miridae	***Lygus rugulipennis***	Bavaria	Bayreuth	49,9260	11,5620
EUBUG_1418_m_Lygugeme3	**KM021762**	Miridae	***Lygus rugulipennis***	Bavaria	Rosenau	48,6600	12,5740
EUBUG_216_m_Lygurugu1	**KM021513**	Miridae	***Lygus rugulipennis***	Baden-Wuerttemberg	Swabian Alb	48,4010	9,4580
EUBUG_218_m_Lygurugu2	**KM022805**	Miridae	***Lygus rugulipennis***	Brandenburg	Schorfheide-Chorin	53,0930	14,0060
EUBUG_703_f_Lygurugu5	**KM022398**	Miridae	***Lygus rugulipennis***	Baden-Wuerttemberg	Pfrunger Ried	47,8990	9,3900
EUBUG_704_f_Lygurugu6	**KM022898**	Miridae	***Lygus rugulipennis***	Baden-Wuerttemberg	Pfrunger Ried	47,8990	9,3900
EUBUG_903_f_Lygurugu8	**KM022596**	Miridae	***Lygus rugulipennis***	Thuringia	Jena	50,9510	11,6240

EUBUG_Seq9_f_Lyguwagn	**KM021504**	Miridae	***Lygus wagneri***	Baden-Wuerttemberg	Swabian Alb	48,4088	9,5323
BFB_Heteroptera_Schmolke_111	**KM022848**	Miridae	***Macrolophus pygmaeus***	Bavaria	Volkach	49,8547	10,2086
EUBUG_710_f_Macrrubi1	**KM022470**	Miridae	***Macrolophus rubi***	Baden-Wuerttemberg	Pfrunger Ried	47,8990	9,3900
BFB_Heteroptera_Schmolke_0324	**KM021732**	Miridae	***Macrotylus herrichi***	Bavaria	Kelheimwinzer	48,9175	11,9178
EUBUG_395_m_Macrpayk1	**KM022203**	Miridae	***Macrotylus paykullii***	Thuringia	Hainich-Duen	51,3020	10,4360
EUBUG_396_m_Macrpayk2	**KM022181**	Miridae	***Macrotylus paykullii***	Thuringia	Hainich-Duen	51,3020	10,4360
EUBUG_397_m_Macrpayk3	**KM022323**	Miridae	***Macrotylus paykullii***	Thuringia	Hainich-Duen	51,3020	10,4360
BFB_Heteroptera_Schmolke_0405	**KM021985**	Miridae	***Macrotylus quadrilineatus***	Bavaria	Urfeld	47,6200	11,3428
BFB_Heteroptera_Schmolke_0219	**KM022195**	Miridae	***Malacocoris chlorizans***	Bavaria	Erlangen	49,5947	10,9836
BFB_Heteroptera_Schmolke_0220	**KM021877**	Miridae	***Malacocoris chlorizans***	Bavaria	Munich	48,1914	11,4847
BFB_Heteroptera_Schmolke_0221	**KM022206**	Miridae	***Malacocoris chlorizans***	Bavaria	Obereichstaett	48,8978	11,1228
BFB_Heteroptera_Schmolke_0222	**KM022671**	Miridae	***Malacocoris chlorizans***	Bavaria	Munich	48,2114	11,6083
BFB_Heteroptera_Schmolke_0407	**KM022949**	Miridae	***Megalocoleus molliculus***	Bavaria	Ebern	50,0872	10,7533
BFB_Heteroptera_Schmolke_0410	**KM021451**	Miridae	***Megalocoleus molliculus***	Bavaria	Offenstetten	48,8111	11,9106
BFB_Heteroptera_Schmolke_0413	**KM021686**	Miridae	***Megalocoleus molliculus***	Bavaria	Munich	48,1914	11,4847
EUBUG_1140_m_Megamoll2	**KM022384**	Miridae	***Megalocoleus molliculus***	Thuringia	Hainich-Duen	51,0230	10,3220
EUBUG_1141_m_Megamoll3	**KM022813**	Miridae	***Megalocoleus molliculus***	Thuringia	Hainich-Duen	51,0230	10,3220
EUBUG_411_f_Megamoll1	**KM022201**	Miridae	***Megalocoleus molliculus***	Thuringia	Hainich-Duen	51,2640	10,4980
EUBUG_937_m_Megatana1	**KM022733**	Miridae	***Megalocoleus molliculus***	Baden-Wuerttemberg	Swabian Alb	48,4510	9,4910
BFB_Heteroptera_Kuechler_0122	**KM021483**	Miridae	***Miris striatus***	Bavaria	Bayreuth, Hohenmirsberger-Platte	49,8150	11,4450
BFB_Heteroptera_Kuechler_0159	**KM022716**	Miridae	***Miris striatus***	Bavaria	Bayreuth, Bindlacher Berg	50,0070	11,6140
EUBUG_315_f_Miristri1	**KM022933**	Miridae	***Miris striatus***	Thuringia	Hainich-Duen	51,1300	10,3810
BFB_Heteroptera_Schmolke_090	**KM022130**	Miridae	***Monalocoris filicis***	Bavaria	Grafenau	48,9264	13,4700
BFB_Heteroptera_Schmolke_091	**KM023010**	Miridae	***Monalocoris filicis***	Bavaria	Uebersee	47,8381	12,4778
EUBUG_1000_f_Monafili3	**KM021631**	Miridae	***Monalocoris filicis***	Bavaria	Bavarian Forest, Rainer Wald	48,9110	12,4510
EUBUG_1004_f_Monafili4	**KM021690**	Miridae	***Monalocoris filicis***	Bavaria	Bavarian Forest, Riedelhuette	48,9210	13,4450
EUBUG_1007_f_Monafili5	**KM021753**	Miridae	***Monalocoris filicis***	Bavaria	Bavarian Forest, Riedelhuette	48,9170	13,3980
EUBUG_1017_m_Monafili5	**KM022836**	Miridae	***Monalocoris filicis***	Bavaria	Bavarian Forest, Rainer Wald	48,9150	12,4550
EUBUG_594_m_Monafili2	**KM021730**	Miridae	***Monalocoris filicis***	Baden-Wuerttemberg	Pfrunger Ried	47,8990	9,3900
BFB_Heteroptera_Schmolke_0414	**KM022984**	Miridae	***Monosynamma bohemanni***	Austria	Hinterriss	47,4723	11,4668
EUBUG_Seq13_f_Myrmgrac	**KM021777**	Miridae	***Myrmecoris gracilis***	Thuringia	Hainich-Duen	51,2641	10,4983
BFB_Heteroptera_Kuechler_0165	**KM022720**	Miridae	***Neolygus contaminatus***	Bavaria	Bayreuth, University	49,9290	11,5830
BFB_Heteroptera_Kuechler_0166	**KM022637**	Miridae	***Neolygus contaminatus***	Bavaria	Bayreuth, University	49,9290	11,5830

EUBUG_711_m_Neolcont1	**KM022996**	Miridae	***Neolygus contaminatus***	Baden-Wuerttemberg	Pfrunger Ried	47,8990	9,3900
EUBUG_712_f_Neolcont2	**KM022738**	Miridae	***Neolygus contaminatus***	Baden-Wuerttemberg	Pfrunger Ried	47,8990	9,3900
EUBUG_1407_f_Neolcont4	**KM021561**	Miridae	***Neolygus viridis***	Thuringia	Hainich-Duen	51,0840	10,4590
EUBUG_713_m_Neolviri1	**KM022277**	Miridae	***Neolygus viridis***	Baden-Wuerttemberg	Pfrunger Ried	47,8990	9,3900
BFB_Heteroptera_Schmolke_0388	**KM022766**	Miridae	***Notostira elongata***	Austria	Oggau	47,8324	16,6642
EUBUG_202_m_Notoelon1	**KM022269**	Miridae	***Notostira elongata***	Baden-Wuerttemberg	Swabian Alb	48,4590	9,4600
EUBUG_203_m_Notoelon2	**KM022209**	Miridae	***Notostira elongata***	Baden-Wuerttemberg	Swabian Alb	48,4590	9,4600
EUBUG_204_m_Notoelon3	**KM022341**	Miridae	***Notostira elongata***	Baden-Wuerttemberg	Swabian Alb	48,4590	9,4600
EUBUG_884_f_Notoerra6	**KM021536**	Miridae	***Notostira elongata***	Rhineland Palatinate	Fischbach bei Dahn	49,0900	7,7150
EUBUG_919_m_Notoelon5	**KM022396**	Miridae	***Notostira elongata***	Thuringia	Jena	50,9510	11,6240
EUBUG_927_f_Notoelon6	**KM022938**	Miridae	***Notostira elongata***	Thuringia	Jena	50,9510	11,6240
BFB_Heteroptera_Kuechler_0356	**KM021637**	Miridae	***Notostira erratica***	Bavaria	Bayreuth, Botanical Garden	49,9230	11,5850
EUBUG_207_m_Notoerra1	**KM022629**	Miridae	***Notostira erratica***	Brandenburg	Schorfheide-Chorin	53,1060	14,0180
EUBUG_208_m_Notoerra2	**KM022166**	Miridae	***Notostira erratica***	Brandenburg	Schorfheide-Chorin	53,1060	14,0180
EUBUG_882_f_Notoerra4	**KM022982**	Miridae	***Notostira erratica***	Rhineland Palatinate	Fischbach bei Dahn	49,0900	7,7150
EUBUG_883_f_Notoerra5	**KM021858**	Miridae	***Notostira erratica***	Rhineland Palatinate	Fischbach bei Dahn	49,0900	7,7150
EUBUG_914_f_Notoelon4	**KM021840**	Miridae	***Notostira erratica***	Thuringia	Jena	50,9510	11,6240
BFB_Heteroptera_Schmolke_0278	**KM023066**	Miridae	***Omphalonotus quadriguttatus***	Bavaria	Kallmuenz	49,1842	11,9450
BFB_Heteroptera_Schmolke_0418	**KM022238**	Miridae	***Oncotylus punctipes***	Bavaria	Spalt	49,1731	10,9847
BFB_Heteroptera_Schmolke_0419	**KM022471**	Miridae	***Oncotylus punctipes***	Bavaria	Erlangen	49,5861	11,0308
BFB_Heteroptera_Schmolke_0420	**KM021767**	Miridae	***Oncotylus punctipes***	Bavaria	Kallmuenz	49,1842	11,9450
BFB_Heteroptera_Schmolke_172	**KM022647**	Miridae	***Orthocephalus brevis***	Bavaria	Munich	48,2008	11,4828
BFB_Heteroptera_Schmolke_173	**KM022523**	Miridae	***Orthocephalus brevis***	Bavaria	Obereichstaett	48,8978	11,1228
BFB_Heteroptera_Kuechler_0156	**KM021636**	Miridae	***Orthocephalus coriaceus***	Bavaria	Bayreuth, Bindlacher Berg	50,0070	11,6140
BFB_Heteroptera_Kuechler_0157	**KM021718**	Miridae	***Orthocephalus coriaceus***	Bavaria	Bayreuth, Bindlacher Berg	50,0070	11,6140
BFB_Heteroptera_Kuechler_0158	**KM022665**	Miridae	***Orthocephalus coriaceus***	Bavaria	Bayreuth, Bindlacher Berg	50,0070	11,6140
BFB_Heteroptera_Schmolke_174	**KM021836**	Miridae	***Orthocephalus coriaceus***	Bavaria	Karlstadt	50,0466	9,7559
BFB_Heteroptera_Schmolke_175	**KM022646**	Miridae	***Orthocephalus coriaceus***	Bavaria	Munich	48,2008	11,4828
BFB_Heteroptera_Schmolke_176	**KM022872**	Miridae	***Orthocephalus coriaceus***	Bavaria	Leinburg	49,4425	11,2822
BFB_Heteroptera_Schmolke_177	**KM023046**	Miridae	***Orthocephalus coriaceus***	Bavaria	Treuchtlingen	48,9672	10,9214
EUBUG_434_f_Orthcori1	**KM021856**	Miridae	***Orthocephalus coriaceus***	Baden-Wuerttemberg	Swabian Alb	48,4100	9,5670
EUBUG_435_f_Orthcori2	**KM022364**	Miridae	***Orthocephalus coriaceus***	Baden-Wuerttemberg	Swabian Alb	48,4100	9,5670
EUBUG_228_f_Orthsalt1	**KM022508**	Miridae	***Orthocephalus saltator***	Thuringia	Hainich-Duen	51,2140	10,3870

EUBUG_229_f_Orthsalt2	**KM022359**	Miridae	***Orthocephalus saltator***	Thuringia	Hainich-Duen	51,2140	10,3870
EUBUG_230_f_Orthsalt3	**KM021641**	Miridae	***Orthocephalus saltator***	Thuringia	Hainich-Duen	51,2140	10,3870
EUBUG_905_m_Orthsalt4	**KM021683**	Miridae	***Orthocephalus saltator***	Thuringia	Jena	50,9510	11,6240
BFB_Heteroptera_Kuechler_0169	**KM022851**	Miridae	***Orthonotus rufifrons***	Bavaria	Bayreuth, Botanical Garden	49,9230	11,5830
BFB_Heteroptera_Kuechler_0183	**KM021869**	Miridae	***Orthonotus rufifrons***	Bavaria	Bayreuth, Botanical Garden	49,9230	11,5870
BFB_Heteroptera_Kuechler_0153	**KM022617**	Miridae	***Orthops basalis***	Bavaria	Wunsiedel, Kleinwendern	50,0000	12,0190
BFB_Heteroptera_Kuechler_0283	**KM022731**	Miridae	***Orthops basalis***	Bavaria	Kronach, Rennesberg	50,2570	11,3700
EUBUG_275_f_Orthbasa1	**KM021703**	Miridae	***Orthops basalis***	Brandenburg	Schorfheide-Chorin	53,1030	13,9860
EUBUG_276_f_Orthbasa2	**KM023132**	Miridae	***Orthops basalis***	Brandenburg	Schorfheide-Chorin	53,1030	13,9860
EUBUG_466_f_Orthcamp2	**KM021843**	Miridae	***Orthops basalis***	Brandenburg	Schorfheide-Chorin	53,1070	13,9980
EUBUG_934_m_Orthkalm2	**KM022026**	Miridae	***Orthops basalis***	Baden-Wuerttemberg	Swabian Alb	48,4100	9,5670
BFB_Heteroptera_Kuechler_0262	**KM022513**	Miridae	***Orthops campestris***	Bavaria	Bayreuth	49,9260	11,5620
EUBUG_347_m_Orthcamp1	**KM022148**	Miridae	***Orthops campestris***	Baden-Wuerttemberg	Fronreute	47,8470	9,6100
BFB_Heteroptera_Kuechler_0112	**KM022509**	Miridae	***Orthops kalmii***	Bavaria	Bayreuth, Neubuerg	49,8920	11,4020
BFB_Heteroptera_Kuechler_0282	**KM021449**	Miridae	***Orthops kalmii***	Bavaria	Kronach, Rennesberg	50,2570	11,3700
EUBUG_277_f_Orthbasa3	**KM021997**	Miridae	***Orthops kalmii***	Brandenburg	Schorfheide-Chorin	53,1030	13,9860
EUBUG_666_f_Orthcamp3	**KM022234**	Miridae	***Orthops kalmii***	Bavaria	Germering	48,1130	11,3880
BFB_Heteroptera_Schmolke_0244	**KM021924**	Miridae	***Orthotylus concolor***	Bavaria	Elsendorf	48,7164	11,7981
BFB_Heteroptera_Kuechler_0272	**KM022686**	Miridae	***Orthotylus ericetorum***	Bavaria	Bayreuth, Botanical Garden	49,9230	11,5850
BFB_Heteroptera_Kuechler_0273	**KM022899**	Miridae	***Orthotylus ericetorum***	Bavaria	Bayreuth, Botanical Garden	49,9230	11,5850
BFB_Heteroptera_Schmolke_0223	**KM023024**	Miridae	***Orthotylus ericetorum***	Bavaria	Koenigsdorf	47,8198	11,4826
BFB_Heteroptera_Schmolke_0224	**KM023074**	Miridae	***Orthotylus ericetorum***	Bavaria	Munich	47,9661	11,5247
BFB_Heteroptera_Schmolke_0225	**KM022496**	Miridae	***Orthotylus ericetorum***	Bavaria	Sachsenkam	47,8008	11,6025
BFB_Heteroptera_Schmolke_0226	**KM022265**	Miridae	***Orthotylus ericetorum***	Bavaria	Weyarn	47,8303	11,8292
EUBUG_742_f_Ortheric1	**KM022227**	Miridae	***Orthotylus ericetorum***	Rhineland Palatinate	Fischbach bei Dahn	49,1040	7,6830
EUBUG_743_f_Ortheric2	**KM021549**	Miridae	***Orthotylus ericetorum***	Rhineland Palatinate	Fischbach bei Dahn	49,1040	7,6830
EUBUG_744_m_Ortheric3	**KM021984**	Miridae	***Orthotylus ericetorum***	Rhineland Palatinate	Fischbach bei Dahn	49,1040	7,6830
EUBUG_745_m_Ortheric4	**KM021590**	Miridae	***Orthotylus ericetorum***	Rhineland Palatinate	Fischbach bei Dahn	49,1040	7,6830
EUBUG_747_m_Ortheric5	**KM021876**	Miridae	***Orthotylus ericetorum***	Rhineland Palatinate	Fischbach bei Dahn	49,1040	7,6830
BFB_Heteroptera_Schmolke_0228	**KM022194**	Miridae	***Orthotylus flavinervis***	Bavaria	Munich	48,1672	11,4919
BFB_Heteroptera_Schmolke_0229	**KM022571**	Miridae	***Orthotylus flavinervis***	Bavaria	Martinsried	48,1122	11,4622
BFB_Heteroptera_Schmolke_0250	**KM021716**	Miridae	***Orthotylus fuscescens***	Bavaria	Munich	48,1914	11,4847
BFB_Heteroptera_Schmolke_0230	**KM022139**	Miridae	***Orthotylus interpositus***	Bavaria	Munich	48,0933	11,6683

BFB_Heteroptera_Schmolke_0233	**KM022774**	Miridae	***Orthotylus interpositus***	Bavaria	Pollanten	49,1511	11,4433
BFB_Heteroptera_Schmolke_0234	**KM022765**	Miridae	***Orthotylus interpositus***	Bavaria	Siegenburg	48,7589	11,8383
EUBUG_560_m_Orthinte1	**KM022099**	Miridae	***Orthotylus interpositus***	Baden-Wuerttemberg	Pfrunger Ried	47,8990	9,3900
EUBUG_561_m_Orthinte2	**KM021980**	Miridae	***Orthotylus interpositus***	Baden-Wuerttemberg	Pfrunger Ried	47,8990	9,3900
EUBUG_562_f_Orthinte3	**KM021596**	Miridae	***Orthotylus interpositus***	Baden-Wuerttemberg	Pfrunger Ried	47,8990	9,3900
EUBUG_563_f_Orthinte4	**KM023022**	Miridae	***Orthotylus interpositus***	Baden-Wuerttemberg	Pfrunger Ried	47,8990	9,3900
BFB_Heteroptera_Kuechler_0160	**KM022530**	Miridae	***Orthotylus marginalis***	Bavaria	Bayreuth, Bindlacher Berg	50,0070	11,6140
BFB_Heteroptera_Schmolke_0231	**KM022159**	Miridae	***Orthotylus marginalis***	Bavaria	Munich	48,2008	11,4828
BFB_Heteroptera_Schmolke_0232	**KM022880**	Miridae	***Orthotylus marginalis***	Bavaria	Munich	48,1906	11,5269
EUBUG_533_f_Orthmarg1	**KM022288**	Miridae	***Orthotylus marginalis***	Baden-Wuerttemberg	Pfrunger Ried	47,8990	9,3900
EUBUG_534_m_Orthmarg2	**KM022944**	Miridae	***Orthotylus marginalis***	Baden-Wuerttemberg	Pfrunger Ried	47,8990	9,3900
EUBUG_535_m_Orthmarg3	**KM022440**	Miridae	***Orthotylus marginalis***	Baden-Wuerttemberg	Pfrunger Ried	47,8990	9,3900
EUBUG_536_m_Orthmarg4	**KM021711**	Miridae	***Orthotylus marginalis***	Baden-Wuerttemberg	Pfrunger Ried	47,8990	9,3900
EUBUG_537_m_Orthmarg5	**KM022854**	Miridae	***Orthotylus marginalis***	Baden-Wuerttemberg	Pfrunger Ried	47,8990	9,3900
EUBUG_677_f_Lygorugi1	**KM022388**	Miridae	***Orthotylus marginalis***	Baden-Wuerttemberg	Pfrunger Ried	47,8990	9,3900
EUBUG_678_f_Lygorugi2	**KM023031**	Miridae	***Orthotylus marginalis***	Baden-Wuerttemberg	Pfrunger Ried	47,8990	9,3900
EUBUG_1011_f_Plespine4	**KM022445**	Miridae	***Orthotylus obscurus***	Bavaria	Bavarian Forest, Hinterreckenberg	48,7260	13,0980
EUBUG_1012_f_Plespine5	**KM021939**	Miridae	***Orthotylus obscurus***	Bavaria	Bavarian Forest, Hinterreckenberg	48,7260	13,0980
EUBUG_1013_f_Plespine6	**KM022080**	Miridae	***Orthotylus obscurus***	Bavaria	Bavarian Forest, Hinterreckenberg	48,7260	13,0980
EUBUG_1027_f_Plespine8	**KM022712**	Miridae	***Orthotylus obscurus***	Bavaria	Bavarian Forest, Hinterreckenberg	48,7260	13,0980
EUBUG_1028_f_Plespine9	**KM022375**	Miridae	***Orthotylus obscurus***	Bavaria	Bavarian Forest, Hinterreckenberg	48,7260	13,0980
BFB_Heteroptera_Kuechler_0236	**KM021785**	Miridae	***Orthotylus prasinus***	Bavaria	Bayreuth, Botanical Garden	49,9230	11,5850
BFB_Heteroptera_Schmolke_0235	**KM022593**	Miridae	***Orthotylus prasinus***	Bavaria	Munich	48,2008	11,4828
BFB_Heteroptera_Schmolke_0236	**KM022429**	Miridae	***Orthotylus prasinus***	Bavaria	Munich	48,2114	11,6083
BFB_Heteroptera_Schmolke_0237	**KM022690**	Miridae	***Orthotylus prasinus***	Bavaria	Munich	48,1906	11,5269
BFB_Heteroptera_Schmolke_0238	**KM022179**	Miridae	***Orthotylus prasinus***	Bavaria	Munich	48,0961	11,5514
EUBUG_502_f_Orthviri2	**KM021537**	Miridae	***Orthotylus prasinus***	Bavaria	Bavarian Forest, Koetzting	49,1820	12,8443
BFB_Heteroptera_Schmolke_0227	**KM023108**	Miridae	***Orthotylus schoberiae***	Austria	Podersdorf	47,8550	16,8371
BFB_Heteroptera_Schmolke_0239	**KM023078**	Miridae	***Orthotylus tenellus***	Bavaria	Munich	48,1672	11,4919
BFB_Heteroptera_Schmolke_0240	**KM023137**	Miridae	***Orthotylus tenellus***	Bavaria	Maising	47,9828	11,2883
BFB_Heteroptera_Schmolke_0241	**KM021694**	Miridae	***Orthotylus tenellus***	Bavaria	Munich	48,1906	11,5269
EUBUG_1224_f_Orthpras1	**KM021776**	Miridae	***Orthotylus tenellus***	Bavaria	Bavarian Forest, Rainer Wald	48,9150	12,4550
BFB_Heteroptera_Schmolke_0247	**KM021880**	Miridae	***Orthotylus virescens***	Bavaria	Pleinfeld	49,1217	11,0003

BFB_Heteroptera_Schmolke_0249	**KM021446**	Miridae	***Orthotylus virescens***	Bavaria	Nuernberg	49,4733	11,1362
BFB_Heteroptera_Schmolke_0242	**KM022002**	Miridae	***Orthotylus viridinervis***	Bavaria	Neuses	49,8442	10,3594
BFB_Heteroptera_Schmolke_0243	**KM022804**	Miridae	***Orthotylus viridinervis***	Bavaria	Erlangen	49,5947	10,9836
EUBUG_501_m_Orthviri1	**KM021594**	Miridae	***Orthotylus viridinervis***	Bavaria	Bavarian Forest, Koetzting	49,1820	12,8443
EUBUG_1009_m_Pachpara3	**KM022486**	Miridae	***Pachytomella parallela***	Bavaria	Bavarian Forest, Riedelhuette	48,9170	13,3980
BFB_Heteroptera_Kuechler_0285	**KM022211**	Miridae	***Pantilius tunicatus***	Bavaria	Schweinfurt	50,0510	10,2540
BFB_Heteroptera_Kuechler_0309	**KM021646**	Miridae	***Pantilius tunicatus***	Rhineland Palatinate	Fischbach bei Dahn	49,1290	7,6900
BFB_Heteroptera_Kuechler_0310	**KM021493**	Miridae	***Pantilius tunicatus***	Rhineland Palatinate	Fischbach bei Dahn	49,1290	7,6900
EUBUG_862_f_Panttuni5	**KM022295**	Miridae	***Pantilius tunicatus***	Rhineland Palatinate	Fischbach bei Dahn	49,0860	7,7230
BFB_Heteroptera_Schmolke_0330	**KM021805**	Miridae	***Parapsallus vitellinus***	Bavaria	Kelheimwinzer	48,9175	11,9178
BFB_Heteroptera_Schmolke_0423	**KM021909**	Miridae	***Parapsallus vitellinus***	Bavaria	Drachselsried	49,1294	13,0678
BFB_Heteroptera_Schmolke_0425	**KM022274**	Miridae	***Parapsallus vitellinus***	Bavaria	Munich	48,0961	11,5514
EUBUG_306_m_Paravite1	**KM022259**	Miridae	***Parapsallus vitellinus***	Thuringia	Hainich-Duen	51,2100	10,3700
BFB_Heteroptera_Kuechler_0172	**KM022689**	Miridae	***Phoenicocoris modestus***	Bavaria	Bayreuth, Botanical Garden	49,9230	11,5830
BFB_Heteroptera_Kuechler_0173	**KM022678**	Miridae	***Phoenicocoris modestus***	Bavaria	Bayreuth, Botanical Garden	49,9230	11,5830
BFB_Heteroptera_Schmolke_0427	**KM021625**	Miridae	***Phoenicocoris modestus***	Bavaria	Erlangen	49,5947	10,9836
EUBUG_1010_m_Phoemode5	**KM023086**	Miridae	***Phoenicocoris modestus***	Bavaria	Bavarian Forest, Rainer Wald	48,9150	12,4430
EUBUG_1016_m_Phoemode6	**KM022013**	Miridae	***Phoenicocoris modestus***	Bavaria	Bavarian Forest, Rainer Wald	48,9150	12,4550
EUBUG_1020_f_Phoemode7	**KM021435**	Miridae	***Phoenicocoris modestus***	Bavaria	Bavarian Forest, Solla	48,8200	13,3050
EUBUG_1029_m_Phoemode8	**KM021606**	Miridae	***Phoenicocoris modestus***	Bavaria	Bavarian Forest, Hinterreckenberg	48,7260	13,0980
EUBUG_1030_m_Phoemode9	**KM022896**	Miridae	***Phoenicocoris modestus***	Bavaria	Bavarian Forest, Hinterreckenberg	48,7260	13,0980
EUBUG_1031_m_Phoemode10	**KM022226**	Miridae	***Phoenicocoris modestus***	Bavaria	Bavarian Forest, Hinterreckenberg	48,7260	13,0980
BFB_Heteroptera_Kuechler_0174	**KM022695**	Miridae	***Phoenicocoris obscurellus***	Bavaria	Bayreuth, Botanical Garden	49,9230	11,5830
BFB_Heteroptera_Kuechler_0175	**KM021560**	Miridae	***Phoenicocoris obscurellus***	Bavaria	Bayreuth, Botanical Garden	49,9230	11,5830
BFB_Heteroptera_Kuechler_0176	**KM022558**	Miridae	***Phoenicocoris obscurellus***	Bavaria	Bayreuth, Botanical Garden	49,9230	11,5830
EUBUG_1032_f_Phoemode11	**KM022019**	Miridae	***Phoenicocoris obscurellus***	Bavaria	Bavarian Forest, Hinterreckenberg	48,7260	13,0980
BFB_Heteroptera_Schmolke_0325	**KM022260**	Miridae	***Phylus coryli***	Bavaria	Martinsried	48,1122	11,4622
BFB_Heteroptera_Schmolke_0433	**KM022303**	Miridae	***Phylus coryli***	Bavaria	Munich	48,2008	11,4828
BFB_Heteroptera_Schmolke_0434	**KM022112**	Miridae	***Phylus coryli***	Bavaria	Munich	48,1906	11,5269
BFB_Heteroptera_Schmolke_0435	**KM022147**	Miridae	***Phylus coryli***	Bavaria	Munich	48,0933	11,6683
BFB_Heteroptera_Schmolke_0436	**KM022029**	Miridae	***Phylus melanocephalus***	Bavaria	Munich	48,0328	11,5117
BFB_Heteroptera_Schmolke_0437	**KM021687**	Miridae	***Phylus melanocephalus***	Bavaria	Erlangen	49,5947	10,9836
BFB_Heteroptera_Schmolke_0438	**KM021670**	Miridae	***Phylus melanocephalus***	Bavaria	Munich	48,2008	11,4828

EUBUG_1176_f_Phylmela11	**KM022879**	Miridae	***Phylus melanocephalus***	Bavaria	Bavarian Forest, Rainer Wald	48,9150	12,4430
EUBUG_1177_m_Phylmela12	**KM021553**	Miridae	***Phylus melanocephalus***	Bavaria	Bavarian Forest, Rainer Wald	48,9150	12,4430
BFB_Heteroptera_Schmolke_0440	**KM021916**	Miridae	***Phylus plagiatus***	Bavaria	Lenggries	47,5950	11,5536
BFB_Heteroptera_Schmolke_0441	**KM022367**	Miridae	***Phylus plagiatus***	Bavaria	Munich	48,0933	11,6683
BFB_Heteroptera_Schmolke_0442	**KM021749**	Miridae	***Phylus plagiatus***	Bavaria	Munich	48,0961	11,5514
EUBUG_1169_m_Phytaust1	**KM021719**	Miridae	***Phytocoris austriacus***	Bavaria	Bavarian Forest, Jochenstein	48,5170	13,7250
EUBUG_1182_f_Phytdimi5	**KM021994**	Miridae	***Phytocoris dimidiatus***	Bavaria	Bavarian Forest, Rainer Wald	48,9150	12,4430
EUBUG_796_f_Phytdimi2	**KM021832**	Miridae	***Phytocoris dimidiatus***	Baden-Wuerttemberg	Fronreute	47,8990	9,3900
BFB_Heteroptera_Kuechler_0357	**KM022548**	Miridae	***Phytocoris longipennis***	Bavaria	Bayreuth, Botanical Garden	49,9230	11,5850
EUBUG_1158_f_Phytintr6	**KM022636**	Miridae	***Phytocoris longipennis***	Bavaria	Bavarian Forest, Riedelhuette	48,9170	13,3980
EUBUG_1170_f_Phytlong8	**KM022633**	Miridae	***Phytocoris longipennis***	Bavaria	Bavarian Forest, Solla	48,8200	13,3050
EUBUG_1216_f_Phytintr7	**KM021970**	Miridae	***Phytocoris longipennis***	Bavaria	Bavarian Forest, Solla	48,8200	13,3050
EUBUG_1221_f_Phytintr8	**KM022612**	Miridae	***Phytocoris longipennis***	Bavaria	Bavarian Forest, Hinterreckenberg	48,7260	13,0980
EUBUG_1344_m_Phytlong9	**KM022992**	Miridae	***Phytocoris longipennis***	Thuringia	Hainich-Duen	51,3420	10,5090
EUBUG_1402_f_Phytlong10	**KM022140**	Miridae	***Phytocoris longipennis***	Thuringia	Hainich-Duen	51,0840	10,4590
EUBUG_725_f_Phytlong6	**KM022460**	Miridae	***Phytocoris longipennis***	Rhineland Palatinate	Fischbach bei Dahn	49,0860	7,7230
EUBUG_1222_m_Phytpini1	**KM022174**	Miridae	***Phytocoris pini***	Bavaria	Bavarian Forest, Hinterreckenberg	48,7260	13,0980
EUBUG_1333_m_Phytpini2	**KM022543**	Miridae	***Phytocoris pini***	Thuringia	Hainich-Duen	51,2430	10,3130
EUBUG_1171_f_Phytpopu1	**KM021563**	Miridae	***Phytocoris populi***	Bavaria	Bavarian Forest, Solla	48,8200	13,3050
EUBUG_1172_f_Phytpopu2	**KM022247**	Miridae	***Phytocoris populi***	Bavaria	Bavarian Forest, Solla	48,8200	13,3050
EUBUG_1173_f_Phytpopu3	**KM021766**	Miridae	***Phytocoris populi***	Bavaria	Bavarian Forest, Solla	48,8200	13,3050
EUBUG_1174_f_Phytpopu4	**KM022086**	Miridae	***Phytocoris populi***	Bavaria	Bavarian Forest, Solla	48,8200	13,3050
EUBUG_1213_f_Phytpopu5	**KM022634**	Miridae	***Phytocoris populi***	Bavaria	Bavarian Forest, Solla	48,8200	13,3050
EUBUG_1214_f_Phytpopu6	**KM022225**	Miridae	***Phytocoris populi***	Bavaria	Bavarian Forest, Solla	48,8200	13,3050
EUBUG_1175_f_Phyttili4	**KM021452**	Miridae	***Phytocoris tiliae***	Bavaria	Bavarian Forest, Solla	48,8200	13,3050
EUBUG_1340_f_Phyttili5	**KM023124**	Miridae	***Phytocoris tiliae***	Thuringia	Hainich-Duen	51,1300	10,3810
EUBUG_1341_f_Phyttili6	**KM022106**	Miridae	***Phytocoris tiliae***	Thuringia	Hainich-Duen	51,2830	10,2270
EUBUG_1342_f_Phyttili7	**KM021905**	Miridae	***Phytocoris tiliae***	Thuringia	Hainich-Duen	51,3420	10,5090
EUBUG_1343_m_Phyttili8	**KM023065**	Miridae	***Phytocoris tiliae***	Thuringia	Hainich-Duen	51,3420	10,5090
BFB_Heteroptera_Kuechler_0239	**KM022985**	Miridae	***Phytocoris ulmi***	Bavaria	Bayreuth, Botanical Garden	49,9230	11,5850
BFB_Heteroptera_Kuechler_0260	**KM022185**	Miridae	***Phytocoris ulmi***	Bavaria	Bayreuth	49,9260	11,5620
EUBUG_1147_f_Phytvari4	**KM021721**	Miridae	***Phytocoris varipes***	Thuringia	Hainich-Duen	51,0690	10,4650
EUBUG_288_m_Phytovari1	**KM022641**	Miridae	***Phytocoris varipes***	Thuringia	Hainich-Duen	51,0680	10,4860

BFB_Heteroptera_Kuechler_0304	**KM021789**	Miridae	***Pilophorus cinnamopterus***	Rhineland Palatinate	Fischbach bei Dahn	49,0910	7,7150
BFB_Heteroptera_Schmolke_0255	**KM022561**	Miridae	***Pilophorus cinnamopterus***	Bavaria	Munich	48,1914	11,4847
BFB_Heteroptera_Schmolke_0257	**KM021831**	Miridae	***Pilophorus cinnamopterus***	Bavaria	Lenggries	47,5950	11,5536
BFB_Heteroptera_Schmolke_0258	**KM022877**	Miridae	***Pilophorus cinnamopterus***	Bavaria	Schernfeld	48,9114	11,1164
BFB_Heteroptera_Kuechler_0256	**KM022718**	Miridae	***Pilophorus clavatus***	Bavaria	Bayreuth, Botanical Garden	49,9230	11,5850
BFB_Heteroptera_Schmolke_0260	**KM022085**	Miridae	***Pilophorus clavatus***	Bavaria	Erlangen	49,5947	10,9836
BFB_Heteroptera_Schmolke_0261	**KM022333**	Miridae	***Pilophorus clavatus***	Bavaria	Munich	48,2114	11,6083
BFB_Heteroptera_Schmolke_0262	**KM022704**	Miridae	***Pilophorus clavatus***	Bavaria	Munich	48,2269	11,5444
EUBUG_505_m_Piloclav1	**KM022135**	Miridae	***Pilophorus clavatus***	Bavaria	Bavarian Forest, Solla	48,8200	13,3050
BFB_Heteroptera_Schmolke_0263	**KM021467**	Miridae	***Pilophorus confusus***	Bavaria	Munich	48,1906	11,5269
BFB_Heteroptera_Schmolke_0266	**KM022590**	Miridae	***Pilophorus confusus***	Bavaria	Vorderriss	47,5612	11,4368
BFB_Heteroptera_Kuechler_0215	**KM022111**	Miridae	***Pilophorus perplexus***	Bavaria	Rothenburg ob der Tauber	49,3780	10,1840
BFB_Heteroptera_Kuechler_0274	**KM021992**	Miridae	***Pilophorus perplexus***	Bavaria	Bayreuth, Botanical Garden	49,9230	11,5850
BFB_Heteroptera_Kuechler_0366	**KM022603**	Miridae	***Pilophorus perplexus***	Bavaria	Bayreuth, Schlehenmuehle	49,9050	11,6240
BFB_Heteroptera_Kuechler_0367	**KM021611**	Miridae	***Pilophorus perplexus***	Bavaria	Bayreuth, Schlehenmuehle	49,9050	11,6240
BFB_Heteroptera_Schmolke_0267	**KM021633**	Miridae	***Pilophorus perplexus***	Bavaria	Pleinfeld	49,1217	11,0003
BFB_Heteroptera_Schmolke_0268	**KM022618**	Miridae	***Pilophorus perplexus***	Bavaria	Nuernberg	49,3861	11,0303
BFB_Heteroptera_Schmolke_0269	**KM022680**	Miridae	***Pilophorus perplexus***	Bavaria	Munich	48,1914	11,4847
BFB_Heteroptera_Schmolke_0270	**KM022703**	Miridae	***Pilophorus perplexus***	Bavaria	Muehlhausen	51,5224	10,5249
BFB_Heteroptera_Schmolke_0272	**KM021600**	Miridae	***Pilophorus simulans***	Bavaria	Erlangen	49,5861	11,0308
BFB_Heteroptera_Kuechler_0230	**KM022769**	Miridae	***Pinalitus atomarius***	Bavaria	Bayreuth	49,9260	11,5620
BFB_Heteroptera_Kuechler_0378	**KM023111**	Miridae	***Pinalitus atomarius***	Bavaria	Bayreuth, Botanical Garden	49,9230	11,5850
EUBUG_1228_f_Pinaatom1	**KM021967**	Miridae	***Pinalitus atomarius***	Bavaria	Bavarian Forest, Riedelhuette	48,9170	13,3980
EUBUG_1160_m_Pinarubr4	**KM022768**	Miridae	***Pinalitus rubricatus***	Bavaria	Bavarian Forest, Riedelhuette	48,9210	13,4125
EUBUG_436_f_Pinarubr1	**KM022967**	Miridae	***Pinalitus rubricatus***	Baden-Wuerttemberg	Swabian Alb	48,3990	9,5180
EUBUG_866_f_Pinavisc1	**KM022461**	Miridae	***Pinalitus viscicola***	Baden-Wuerttemberg	Wangen	47,7000	9,8000
BFB_Heteroptera_Schmolke_145	**KM022155**	Miridae	***Pithanus maerkelii***	Bavaria	Munich	48,1906	11,5269
EUBUG_398_f_Pithmaer1	**KM021922**	Miridae	***Pithanus maerkelii***	Thuringia	Hainich-Duen	51,2710	10,4180
EUBUG_399_f_Pithmaer2	**KM021503**	Miridae	***Pithanus maerkelii***	Thuringia	Hainich-Duen	51,2710	10,4180
EUBUG_Seq19_f_Pithmaer	**KM022714**	Miridae	***Pithanus maerkelii***	Thuringia	Hainich-Duen	51,2712	10,4179
BFB_Heteroptera_Kuechler_0243	**KM022900**	Miridae	***Plagiognathus arbustorum***	Bavaria	Bayreuth, Botanical Garden	49,9230	11,5850
BFB_Heteroptera_Kuechler_0244	**KM022177**	Miridae	***Plagiognathus arbustorum***	Bavaria	Bayreuth, Botanical Garden	49,9230	11,5850
BFB_Heteroptera_Schmolke_0328	**KM022376**	Miridae	***Plagiognathus arbustorum***	Bavaria	Martinsried	48,1122	11,4622

EUBUG_1164_m_Plagarbu10	**KM021942**	Miridae	***Plagiognathus arbustorum***	Bavaria	Bavarian Forest, National Park	49,0540	13,2480
EUBUG_1165_m_Plagarbu11	**KM022351**	Miridae	***Plagiognathus arbustorum***	Bavaria	Bavarian Forest, National Park	49,0540	13,2480
EUBUG_1166_f_Plagarbu12	**KM022242**	Miridae	***Plagiognathus arbustorum***	Bavaria	Bavarian Forest, National Park	49,0540	13,2480
EUBUG_1167_f_Plagarbu13	**KM021874**	Miridae	***Plagiognathus arbustorum***	Bavaria	Bavarian Forest, National Park	49,0540	13,2480
EUBUG_450_f_Plagarbu1	**KM022698**	Miridae	***Plagiognathus arbustorum***	Brandenburg	Schorfheide-Chorin	53,1070	14,0000
EUBUG_451_f_Plagarbu2	**KM021623**	Miridae	***Plagiognathus arbustorum***	Brandenburg	Schorfheide-Chorin	53,1070	14,0000
EUBUG_452_f_Plagarbu3	**KM022372**	Miridae	***Plagiognathus arbustorum***	Brandenburg	Schorfheide-Chorin	53,1070	14,0000
EUBUG_539_m_Plagarbu4	**KM023011**	Miridae	***Plagiognathus arbustorum***	Baden-Wuerttemberg	Pfrunger Ried	47,8990	9,3900
EUBUG_540_m_Plagarbu5	**KM023085**	Miridae	***Plagiognathus arbustorum***	Baden-Wuerttemberg	Pfrunger Ried	47,8990	9,3900
EUBUG_672_f_Plagarbu6	**KM022744**	Miridae	***Plagiognathus arbustorum***	Baden-Wuerttemberg	Pfrunger Ried	47,8990	9,3900
EUBUG_673_f_Plagarbu7	**KM022232**	Miridae	***Plagiognathus arbustorum***	Baden-Wuerttemberg	Pfrunger Ried	47,8990	9,3900
EUBUG_674_m_Plagarbu8	**KM022923**	Miridae	***Plagiognathus arbustorum***	Baden-Wuerttemberg	Pfrunger Ried	47,8990	9,3900
EUBUG_675_m_Plagarbu9	**KM021477**	Miridae	***Plagiognathus arbustorum***	Baden-Wuerttemberg	Pfrunger Ried	47,8990	9,3900
BFB_Heteroptera_Schmolke_0331	**KM023001**	Miridae	***Plagiognathus chrysanthemi***	Bavaria	Siegenburg	48,7589	11,8383
EUBUG_231_m_Plagchry1	**KM022058**	Miridae	***Plagiognathus chrysanthemi***	Thuringia	Hainich-Duen	51,2140	10,3870
EUBUG_232_m_Plagchry2	**KM021609**	Miridae	***Plagiognathus chrysanthemi***	Thuringia	Hainich-Duen	51,2140	10,3870
EUBUG_233_m_Plagchry3	**KM022103**	Miridae	***Plagiognathus chrysanthemi***	Thuringia	Hainich-Duen	51,2140	10,3870
EUBUG_551_m_Plagchry4	**KM023013**	Miridae	***Plagiognathus chrysanthemi***	Baden-Wuerttemberg	Pfrunger Ried	47,8990	9,3900
EUBUG_958_m_Plagchry5	**KM022340**	Miridae	***Plagiognathus chrysanthemi***	Thuringia	Jena	50,9510	11,6240
EUBUG_960_m_Plagchry5	**KM022320**	Miridae	***Plagiognathus chrysanthemi***	Thuringia	Jena	50,9510	11,6240
EUBUG_961_m_Plagchry6	**KM021669**	Miridae	***Plagiognathus chrysanthemi***	Thuringia	Jena	50,9510	11,6240
EUBUG_962_m_Plagchry7	**KM022752**	Miridae	***Plagiognathus chrysanthemi***	Thuringia	Jena	50,9510	11,6240
EUBUG_963_m_Plagchry8	**KM023127**	Miridae	***Plagiognathus chrysanthemi***	Thuringia	Jena	50,9510	11,6240
EUBUG_964_f_Plagchry9	**KM021568**	Miridae	***Plagiognathus chrysanthemi***	Thuringia	Jena	50,9510	11,6240
EUBUG_1146_m_Plagfulv1	**KM022386**	Miridae	***Plagiognathus fulvipennis***	Brandenburg	Schorfheide-Chorin	52,8800	13,9650
BFB_Heteroptera_Kuechler_0170	**KM022031**	Miridae	***Plesiodema pinetella***	Bavaria	Bayreuth, Botanical Garden	49,9230	11,5830
BFB_Heteroptera_Kuechler_0171	**KM021927**	Miridae	***Plesiodema pinetella***	Bavaria	Bayreuth, Botanical Garden	49,9230	11,5830
BFB_Heteroptera_Schmolke_0445	**KM022787**	Miridae	***Plesiodema pinetella***	Bavaria	Pipinsried	48,4144	11,3247
EUBUG_1026_m_Plespine7	**KM022724**	Miridae	***Plesiodema pinetella***	Bavaria	Bavarian Forest, Hinterreckenberg	48,7260	13,0980
EUBUG_1420_m_Polyaspe2	**KM022380**	Miridae	***Polymerus asperulae***	Bavaria	Rosenau	48,6610	12,5800
EUBUG_443_f_Polymicr1	**KM023098**	Miridae	***Polymerus microphthalmus***	Baden-Wuerttemberg	Schwaebische Alb	48,3980	9,3420
EUBUG_549_f_Polymicr2	**KM022207**	Miridae	***Polymerus microphthalmus***	Baden-Wuerttemberg	Pfrunger Ried	47,8990	9,3900
EUBUG_1156_m_Polynigr9	**KM022554**	Miridae	***Polymerus nigrita***	Bavaria	Bavarian Forest, National Park	49,0540	13,2480

EUBUG_259_m_Polynigr1	**KM022986**	Miridae	***Polymerus nigrita***	Baden-Wuerttemberg	Swabian Alb	48,4090	9,5320
EUBUG_260_f_Polynigr2	**KM023033**	Miridae	***Polymerus nigrita***	Baden-Wuerttemberg	Swabian Alb	48,4090	9,5320
EUBUG_548_f_Polynigr3	**KM022335**	Miridae	***Polymerus nigrita***	Baden-Wuerttemberg	Pfrunger Ried	47,8990	9,3900
EUBUG_696_f_Polynigr4	**KM022682**	Miridae	***Polymerus nigrita***	Baden-Wuerttemberg	Pfrunger Ried	47,8990	9,3900
EUBUG_697_f_Polynigr5	**KM021436**	Miridae	***Polymerus nigrita***	Baden-Wuerttemberg	Pfrunger Ried	47,8990	9,3900
EUBUG_698_m_Polynigr6	**KM021485**	Miridae	***Polymerus nigrita***	Baden-Wuerttemberg	Pfrunger Ried	47,8990	9,3900
EUBUG_699_m_Polynigr7	**KM022528**	Miridae	***Polymerus nigrita***	Baden-Wuerttemberg	Pfrunger Ried	47,8990	9,3900
EUBUG_911_f_Polynigr8	**KM021991**	Miridae	***Polymerus nigrita***	Thuringia	Jena	50,9510	11,6240
BFB_Heteroptera_Schmolke_0377	**KM022876**	Miridae	***Polymerus palustris***	Bavaria	Pollanten	49,1511	11,4433
BFB_Heteroptera_Kuechler_0110	**KM021846**	Miridae	***Polymerus unifasciatus***	Bavaria	Bayreuth, Neubuerg	49,8920	11,4020
BFB_Heteroptera_Kuechler_0162	**KM021893**	Miridae	***Polymerus unifasciatus***	Bavaria	Bayreuth, Hohenmirsberger-Platte	49,8150	11,4450
BFB_Heteroptera_Kuechler_0281	**KM022061**	Miridae	***Polymerus unifasciatus***	Bavaria	Kronach, Rennesberg	50,2570	11,3700
EUBUG_283_f_Polyunif3	**KM022487**	Miridae	***Polymerus unifasciatus***	Baden-Wuerttemberg	Swabian Alb	48,3950	9,5030
EUBUG_556_m_Polyunif4	**KM022109**	Miridae	***Polymerus unifasciatus***	Baden-Wuerttemberg	Pfrunger Ried	47,8990	9,3900
EUBUG_885_m_Polyunif5	**KM022536**	Miridae	***Polymerus unifasciatus***	Rhineland Palatinate	Fischbach bei Dahn	49,0900	7,7150
EUBUG_886_f_Polyunif6	**KM021965**	Miridae	***Polymerus unifasciatus***	Rhineland Palatinate	Fischbach bei Dahn	49,0900	7,7150
EUBUG_1001_m_Psalalbi10	**KM022783**	Miridae	***Psallus albicinctus***	Bavaria	Bavarian Forest, National Park	49,0540	13,2480
BFB_Heteroptera_Kuechler_0147	**KM021744**	Miridae	***Psallus ambiguus***	Bavaria	Bayreuth, Botanical Garden	49,9230	11,5850
BFB_Heteroptera_Kuechler_0148	**KM021667**	Miridae	***Psallus ambiguus***	Bavaria	Bayreuth, Botanical Garden	49,9230	11,5850
BFB_Heteroptera_Schmolke_0332	**KM021918**	Miridae	***Psallus ambiguus***	Bavaria	Siegenburg	48,7589	11,8383
BFB_Heteroptera_Schmolke_0454	**KM021528**	Miridae	***Psallus ambiguus***	Bavaria	Munich	48,2114	11,6083
BFB_Heteroptera_Schmolke_0455	**KM022094**	Miridae	***Psallus ambiguus***	Bavaria	Martinsried	48,1122	11,4622
BFB_Heteroptera_Schmolke_0456	**KM022385**	Miridae	***Psallus ambiguus***	Bavaria	Munich	48,0961	11,5514
EUBUG_500_f_Psalambig1	**KM022222**	Miridae	***Psallus ambiguus***	Bavaria	Bavarian Forest, Koetzting	49,1820	12,8443
EUBUG_546_f_Psalambi1	**KM021795**	Miridae	***Psallus ambiguus***	Baden-Wuerttemberg	Pfrunger Ried	47,8990	9,3900
EUBUG_1015_m_Psalflav2	**KM022997**	Miridae	***Psallus flavellus***	Bavaria	Bavarian Forest, Jochenstein	48,5190	13,7280
BFB_Heteroptera_Kuechler_0233	**KM023100**	Miridae	***Psallus haematodes***	Bavaria	Bayreuth, Botanical Garden	49,9230	11,5850
BFB_Heteroptera_Kuechler_0234	**KM022193**	Miridae	***Psallus haematodes***	Bavaria	Bayreuth, Botanical Garden	49,9230	11,5850
EUBUG_554_f_Psalhaem1	**KM022965**	Miridae	***Psallus haematodes***	Baden-Wuerttemberg	Pfrunger Ried	47,8990	9,3900
EUBUG_555_m_Psalhaem2	**KM022831**	Miridae	***Psallus haematodes***	Baden-Wuerttemberg	Pfrunger Ried	47,8990	9,3900
EUBUG_557_m_Psalhaem3	**KM022824**	Miridae	***Psallus haematodes***	Baden-Wuerttemberg	Pfrunger Ried	47,8990	9,3900
BFB_Heteroptera_Schmolke_0457	**KM021506**	Miridae	***Psallus lepidus***	Bavaria	Munich	48,0961	11,5514
BFB_Heteroptera_Schmolke_0458	**KM022358**	Miridae	***Psallus mollis***	Bavaria	Munich	48,0933	11,6683

EUBUG_1041_f_Psalhaem4	**KM022071**	Miridae	***Psallus mollis***	Bavaria	Bavarian Forest, Jochenstein	48,5170	13,7250
BFB_Heteroptera_Schmolke_0447	**KM021974**	Miridae	***Psallus montanus***	Bavaria	Erlangen	49,5947	10,9836
EUBUG_1037_m_Psalvabi3	**KM021519**	Miridae	***Psallus variabilis***	Bavaria	Bavarian Forest, Isar estuary	48,7930	12,9700
EUBUG_1178_m_Psalvabi5	**KM021895**	Miridae	***Psallus variabilis***	Bavaria	Bavarian Forest, Rainer Wald	48,9150	12,4430
EUBUG_1179_m_Psalvabi6	**KM022444**	Miridae	***Psallus variabilis***	Bavaria	Bavarian Forest, Rainer Wald	48,9150	12,4430
EUBUG_1180_m_Psalvabi7	**KM022975**	Miridae	***Psallus variabilis***	Bavaria	Bavarian Forest, Rainer Wald	48,9150	12,4430
EUBUG_1181_m_Psalvabi8	**KM022394**	Miridae	***Psallus variabilis***	Bavaria	Bavarian Forest, Rainer Wald	48,9150	12,4430
EUBUG_1210_m_Psalvabi9	**KM022683**	Miridae	***Psallus variabilis***	Bavaria	Bavarian Forest, Rainer Wald	48,9150	12,4430
EUBUG_1212_m_Psalvabi11	**KM023059**	Miridae	***Psallus variabilis***	Bavaria	Bavarian Forest, Rainer Wald	48,9150	12,4430
BFB_Heteroptera_Kuechler_0163	**KM022927**	Miridae	***Psallus varians***	Bavaria	Bayreuth, Hohenmirsberger-Platte	49,8150	11,4450
BFB_Heteroptera_Kuechler_0164	**KM022619**	Miridae	***Psallus varians***	Bavaria	Bayreuth, Hohenmirsberger-Platte	49,8150	11,4450
BFB_Heteroptera_Schmolke_0459	**KM022755**	Miridae	***Psallus varians***	Bavaria	Rothenbuch	49,9658	9,3881
BFB_Heteroptera_Schmolke_0460	**KM022770**	Miridae	***Psallus varians***	Bavaria	Treuchtlingen	48,9672	10,9214
EUBUG_1148_f_Psalvari8	**KM021773**	Miridae	***Psallus varians***	Bavaria	Bavarian Forest, Hinterreckenberg	48,7260	13,0980
EUBUG_1149_f_Psalvari9	**KM021839**	Miridae	***Psallus varians***	Bavaria	Bavarian Forest, Hinterreckenberg	48,7260	13,0980
EUBUG_1150_f_Psalvari10	**KM021768**	Miridae	***Psallus varians***	Bavaria	Bayreuth, Hohenmirsberger-Platte	48,7260	13,0980
EUBUG_1151_f_Psalvari11	**KM021462**	Miridae	***Psallus varians***	Bavaria	Bavarian Forest, Hinterreckenberg	48,7260	13,0980
EUBUG_1152_m_Psalvari12	**KM022943**	Miridae	***Psallus varians***	Bavaria	Bayreuth, Hohenmirsberger-Platte	48,7260	13,0980
EUBUG_1153_m_Psalvari13	**KM022298**	Miridae	***Psallus varians***	Bavaria	Bavarian Forest, Hinterreckenberg	48,7260	13,0980
EUBUG_1154_m_Psalvari14	**KM021845**	Miridae	***Psallus varians***	Bavaria	Bavarian Forest, Hinterreckenberg	48,7260	13,0980
EUBUG_1155_m_Psalvari15	**KM022235**	Miridae	***Psallus varians***	Bavaria	Bayreuth, Hohenmirsberger-Platte	48,7260	13,0980
EUBUG_1163_m_Psalvari16	**KM021741**	Miridae	***Psallus varians***	Bavaria	Bavarian Forest, Riedelhuette	48,9210	13,4125
EUBUG_425_m_Psallvari1	**KM022811**	Miridae	***Psallus varians***	Thuringia	Hainich-Duen	51,2160	10,3220
BFB_Heteroptera_Schmolke_0251	**KM021682**	Miridae	***Pseudoloxops coccineus***	Bavaria	Munich	48,1914	11,4847
BFB_Heteroptera_Schmolke_0252	**KM022615**	Miridae	***Pseudoloxops coccineus***	Bavaria	Ebern	50,0872	10,7533
EUBUG_1207_m_Rhabstri8	**KM022132**	Miridae	***Rhabdomiris striatellus***	Bavaria	Bavarian Forest, Rainer Wald	48,9150	12,4430
EUBUG_1208_f_Rhabstri9	**KM022600**	Miridae	***Rhabdomiris striatellus***	Bavaria	Bavarian Forest, Rainer Wald	48,9150	12,4430
EUBUG_791_f_Rhabstri6	**KM022363**	Miridae	***Rhabdomiris striatellus***	Thuringia	NSG Isserstedter Hol	47,8990	9,3900
EUBUG_792_f_Rhabstri7	**KM022315**	Miridae	***Rhabdomiris striatellus***	Baden-Wuerttemberg	Fronreute	47,8990	9,3900
BFB_Heteroptera_Schmolke_0463	**KM022878**	Miridae	***Salicarus roseri***	Bavaria	Maising	47,9828	11,2883
BFB_Heteroptera_Schmolke_0466	**KM022079**	Miridae	***Salicarus roseri***	Bavaria	Munich	48,0961	11,5514
BFB_Heteroptera_Kuechler_0014	**KM021949**	Miridae	***Stenodema calcarata***	Bavaria	Bayreuth, Botanical Garden	49,9230	11,5830
BFB_Heteroptera_Kuechler_0072	**KM022595**	Miridae	***Stenodema calcarata***	Bavaria	Creussen	49,8490	11,5960

BFB_Heteroptera_Schmolke_147	**KM022025**	Miridae	***Stenodema calcarata***	Bavaria	Munich	48,1906	11,5269
BFB_Heteroptera_Schmolke_149	**KM022316**	Miridae	***Stenodema calcarata***	Austria	Walchsee	47,6534	12,3122
BFB_Heteroptera_Schmolke_150	**KM022989**	Miridae	***Stenodema calcarata***	Bavaria	Uebersee	47,8381	12,4778
EUBUG_212_m_Stencalc2	**KM021898**	Miridae	***Stenodema calcarata***	Brandenburg	Schorfheide-Chorin	53,1060	14,0180
EUBUG_213_f_Stencalc3	**KM022918**	Miridae	***Stenodema calcarata***	Brandenburg	Schorfheide-Chorin	53,1060	14,0180
EUBUG_528_f_Stencalc4	**KM021515**	Miridae	***Stenodema calcarata***	Baden-Wuerttemberg	Pfrunger Ried	47,8990	9,3900
EUBUG_529_m_Stencalc6	**KM023021**	Miridae	***Stenodema calcarata***	Baden-Wuerttemberg	Pfrunger Ried	47,8990	9,3900
EUBUG_682_m_Stencalc7	**KM021960**	Miridae	***Stenodema calcarata***	Baden-Wuerttemberg	Pfrunger Ried	47,8990	9,3900
EUBUG_683_f_Stencalc8	**KM023068**	Miridae	***Stenodema calcarata***	Baden-Wuerttemberg	Pfrunger Ried	47,8990	9,3900
EUBUG_684_f_Stencalc9	**KM022869**	Miridae	***Stenodema calcarata***	Baden-Wuerttemberg	Pfrunger Ried	47,8990	9,3900
BFB_Heteroptera_Kuechler_0151	**KM022857**	Miridae	***Stenodema holsata***	Bavaria	Wunsiedel, Kleinwendern	50,0000	12,0190
BFB_Heteroptera_Kuechler_0152	**KM022402**	Miridae	***Stenodema holsata***	Bavaria	Wunsiedel, Kleinwendern	50,0000	12,0190
BFB_Heteroptera_Schmolke_151	**KM022942**	Miridae	***Stenodema holsata***	Bavaria	Rothenbuch	49,9658	9,3881
BFB_Heteroptera_Schmolke_152	**KM022551**	Miridae	***Stenodema holsata***	Bavaria	Lenggries	47,5950	11,5536
BFB_Heteroptera_Schmolke_153	**KM021735**	Miridae	***Stenodema holsata***	Bavaria	Uebersee	47,8381	12,4778
BFB_Heteroptera_Schmolke_154	**KM021829**	Miridae	***Stenodema holsata***	Bavaria	Urfeld	47,6203	11,3456
EUBUG_1226_m_Stenhol5	**KM021489**	Miridae	***Stenodema holsata***	Bavaria	Bavarian Forest, Riedelhuette	48,9230	13,4150
EUBUG_1227_m_Stenhol6	**KM022581**	Miridae	***Stenodema holsata***	Bavaria	Bavarian Forest, Riedelhuette	48,9230	13,4150
BFB_Heteroptera_Kuechler_0015	**KM022395**	Miridae	***Stenodema laevigata***	Bavaria	Bayreuth, Botanical Garden	49,9230	11,5830
BFB_Heteroptera_Kuechler_0092	**KM022947**	Miridae	***Stenodema laevigata***	Bavaria	Bayreuth, Botanical Garden	49,9230	11,5870
BFB_Heteroptera_Schmolke_155	**KM021476**	Miridae	***Stenodema laevigata***	Bavaria	Windheim	49,8850	9,5697
BFB_Heteroptera_Schmolke_156	**KM021700**	Miridae	***Stenodema laevigata***	Bavaria	Munich	48,1906	11,5269
BFB_Heteroptera_Schmolke_157	**KM021771**	Miridae	***Stenodema laevigata***	Bavaria	Munich	48,2114	11,6083
BFB_Heteroptera_Schmolke_158	**KM022371**	Miridae	***Stenodema laevigata***	Bavaria	Munich	48,2008	11,4828
EUBUG_234_f_Stenlaev1	**KM023087**	Miridae	***Stenodema laevigata***	Thuringia	Hainich-Duen	51,2140	10,3870
EUBUG_235_f_Stenlaev2	**KM022751**	Miridae	***Stenodema laevigata***	Thuringia	Hainich-Duen	51,2140	10,3870
EUBUG_236_m_Stenlaev3	**KM022142**	Miridae	***Stenodema laevigata***	Thuringia	Hainich-Duen	51,2140	10,3870
BFB_Heteroptera_Schmolke_159	**KM022559**	Miridae	***Stenodema sericans***	Bavaria	Lenggries	47,5950	11,5536
BFB_Heteroptera_Kuechler_0206	**KM022150**	Miridae	***Stenotus binotatus***	Bavaria	Bayreuth, Botanical Garden	49,9230	11,5850
BFB_Heteroptera_Schmolke_0373	**KM021579**	Miridae	***Stenotus binotatus***	Bavaria	Martinsried	48,1122	11,4622
EUBUG_217_m_Stenbino	**KM022221**	Miridae	***Stenotus binotatus***	Baden-Wuerttemberg	Swabian Alb	48,4010	9,4580
EUBUG_291_f_Stenbino1	**KM021713**	Miridae	***Stenotus binotatus***	Thuringia	Hainich-Duen	51,2740	10,4100
EUBUG_292_f_Stenbino2	**KM021968**	Miridae	***Stenotus binotatus***	Thuringia	Hainich-Duen	51,2740	10,4100

EUBUG_680_f_Stenbino5	**KM022745**	Miridae	***Stenotus binotatus***	Baden-Wuerttemberg	Pfrunger Ried	47,8990	9,3900
EUBUG_681_f_Stenbino6	**KM023095**	Miridae	***Stenotus binotatus***	Baden-Wuerttemberg	Pfrunger Ried	47,8990	9,3900
BFB_Heteroptera_Kuechler_0133	**KM021582**	Miridae	***Strongylocoris leucocephalus***	Bavaria	Hohenmirsberger-Platte	49,8150	11,4450
BFB_Heteroptera_Kuechler_0134	**KM022009**	Miridae	***Strongylocoris leucocephalus***	Bavaria	Hohenmirsberger-Platte	49,8150	11,4450
BFB_Heteroptera_Schmolke_178	**KM023134**	Miridae	***Strongylocoris leucocephalus***	Bavaria	Schernfeld	48,9114	11,1164
EUBUG_769_m_Stroleuc1	**KM022248**	Miridae	***Strongylocoris leucocephalus***	Baden-Wuerttemberg	Swabian Alb	47,8990	9,3900
EUBUG_Seq30_m_Stroleuc	**KM022178**	Miridae	***Strongylocoris leucocephalus***	Baden-Wuerttemberg	Swabian Alb	48,3946	9,5028
BFB_Heteroptera_Schmolke_180	**KM022589**	Miridae	***Strongylocoris steganoides***	Bavaria	Koenigsbrunn	48,2744	10,9033
BFB_Heteroptera_Schmolke_182	**KM022913**	Miridae	***Strongylocoris steganoides***	Bavaria	Treuchtlingen	48,9672	10,9214
BFB_Heteroptera_Schmolke_183	**KM022970**	Miridae	***Strongylocoris steganoides***	Bavaria	Munich	48,1914	11,4847
EUBUG_933_m_Strosteg1	**KM021663**	Miridae	***Strongylocoris steganoides***	Baden-Wuerttemberg	Swabian Alb	48,3950	9,2600
EUBUG_Seq22_m_Systtrig	**KM022987**	Miridae	***Systellonotus triguttatus***	Thuringia	Hainich-Duen	51,0324	10,5144
EUBUG_255_m_Tetrpalu1	**KM023106**	Miridae	***Teratocoris paludum***	Brandenburg	Schorfheide-Chorin	53,1070	14,0000
EUBUG_Seq23_f_Teraante	**KM021996**	Miridae	***Teratocoris paludum***	Brandenburg	Schorfheide-Chorin	53,0931	14,0060
BFB_Heteroptera_Kuechler_0245	**KM021823**	Miridae	***Trigonotylus caelestialium***	Bavaria	Bayreuth, Botanical Garden	49,9230	11,5850
BFB_Heteroptera_Kuechler_0362	**KM022257**	Miridae	***Trigonotylus caelestialium***	Bavaria	Schlehenmuehle	49,9050	11,6240
BFB_Heteroptera_Schmolke_160	**KM021710**	Miridae	***Trigonotylus caelestialium***	Bavaria	Nuernberg	49,4189	11,3425
BFB_Heteroptera_Schmolke_161	**KM021807**	Miridae	***Trigonotylus caelestialium***	Austria	Podersdorf	47,8527	16,8423
BFB_Heteroptera_Schmolke_162	**KM022419**	Miridae	***Trigonotylus caelestialium***	Bavaria	Munich	48,1914	11,4847
BFB_Heteroptera_Schmolke_163	**KM022510**	Miridae	***Trigonotylus caelestialium***	Bavaria	Munich	48,2269	11,5444
EUBUG_199_f_Trigcael1	**KM021868**	Miridae	***Trigonotylus caelestialium***	Baden-Wuerttemberg	Swabian Alb	48,4590	9,4600
EUBUG_200_f_Trigcael2	**KM021715**	Miridae	***Trigonotylus caelestialium***	Baden-Wuerttemberg	Swabian Alb	48,4590	9,4600
EUBUG_832_m_Trigcael4	**KM023037**	Miridae	***Trigonotylus caelestialium***	Baden-Wuerttemberg	Limpach	49,1040	7,6830
EUBUG_833_f_Trigcael5	**KM022478**	Miridae	***Trigonotylus caelestialium***	Baden-Wuerttemberg	Limpach	49,1040	7,6830
BFB_Heteroptera_Schmolke_164	**KM022039**	Miridae	***Trigonotylus pulchellus***	Bavaria	Erlangen	49,5861	11,0308
BFB_Heteroptera_Schmolke_166	**KM021859**	Miridae	***Trigonotylus pulchellus***	Bavaria	Tennenlohe	49,5556	11,0439
BFB_Heteroptera_Kuechler_0225	**KM021585**	Miridae	***Tytthus pygmaeus***	Bavaria	Bayreuth, Botanical Garden	49,9230	11,5850
EUBUG_476_m_Tyttpygm1	**KM021696**	Miridae	***Tytthus pygmaeus***	Brandenburg	Schorfheide-Chorin	52,9870	13,8280
EUBUG_979_m_Tyttpygm2	**KM022447**	Miridae	***Tytthus pygmaeus***	Baden-Wuerttemberg	Schwaebische Alb	48,3810	9,4190
BFB_Heteroptera_Schmolke_0478	**KM021586**	Nabidae	***Himacerus apterus***	Bavaria	Oberndorf	48,9481	12,0269
BFB_Heteroptera_Schmolke_0479	**KM021941**	Nabidae	***Himacerus apterus***	Bavaria	Ernhofen	49,4189	11,3425
BFB_Heteroptera_Schmolke_0480	**KM021914**	Nabidae	***Himacerus apterus***	Bavaria	Uebersee	47,8381	12,4778
BFB_Heteroptera_Schmolke_0536	**KM022532**	Nabidae	***Himacerus apterus***	Brandenburg	Berlin, Spandau	52,5221	13,1902

EUBUG_1408_f_Himaapte11	**KM021790**	Nabidae	***Himacerus apterus***	Thuringia	Hainich-Duen	51,1940	10,3190
EUBUG_1409_m_Himaapte12	**KM022324**	Nabidae	***Himacerus apterus***	Thuringia	Hainich-Duen	51,1940	10,3190
EUBUG_66_m_Himaapte1	**KM022000**	Nabidae	***Himacerus apterus***	Bavaria	Neuburg an der Donau	48,7320	11,2840
BFB_Heteroptera_Schmolke_0472	**KM022047**	Nabidae	***Himacerus major***	Bavaria	Roth	49,1669	11,1783
BFB_Heteroptera_Kuechler_0016	**KM022956**	Nabidae	***Himacerus mirmicoides***	Bavaria	Bayreuth, Botanical Garden	49,9230	11,5830
BFB_Heteroptera_Kuechler_0017	**KM021826**	Nabidae	***Himacerus mirmicoides***	Bavaria	Bayreuth, Botanical Garden	49,9230	11,5830
BFB_Heteroptera_Schmolke_0473	**KM022974**	Nabidae	***Himacerus mirmicoides***	Bavaria	Bamberg	49,9268	10,9052
BFB_Heteroptera_Schmolke_0474	**KM023026**	Nabidae	***Himacerus mirmicoides***	Bavaria	Munich	48,2008	11,4828
BFB_Heteroptera_Schmolke_0475	**KM022696**	Nabidae	***Himacerus mirmicoides***	Bavaria	Munich	48,2114	11,6083
BFB_Heteroptera_Schmolke_0537	**KM022175**	Nabidae	***Himacerus mirmicoides***	Brandenburg	Berlin, Spandau	52,5221	13,1902
EUBUG_507_f_Himamirm3	**KM021557**	Nabidae	***Himacerus mirmicoides***	Lower Saxony	Wangerooge	53,7940	7,8970
EUBUG_685_juv_Himamirm4	**KM021548**	Nabidae	***Himacerus mirmicoides***	Baden-Wuerttemberg	Pfrunger Ried	47,8990	9,3900
EUBUG_686_juv_Himamirm5	**KM023028**	Nabidae	***Himacerus mirmicoides***	Baden-Wuerttemberg	Pfrunger Ried	47,8990	9,3900
EUBUG_687_juv_Himamirm6	**KM022726**	Nabidae	***Himacerus mirmicoides***	Baden-Wuerttemberg	Pfrunger Ried	47,8990	9,3900
EUBUG_746_f_Himamirm7	**KM022256**	Nabidae	***Himacerus mirmicoides***	Rhineland Palatinate	Fischbach bei Dahn	49,1040	7,6830
EUBUG_748_m_Himamirm8	**KM021432**	Nabidae	***Himacerus mirmicoides***	Rhineland Palatinate	Fischbach bei Dahn	49,1040	7,6830
BFB_Heteroptera_Kuechler_0001	**KM022059**	Nabidae	***Nabis brevis***	Bavaria	Destuben	49,9020	11,5680
BFB_Heteroptera_Kuechler_0020	**KM022903**	Nabidae	***Nabis brevis***	Bavaria	Bayreuth, Botanical Garden	49,9230	11,5830
BFB_Heteroptera_Schmolke_0489	**KM022196**	Nabidae	***Nabis brevis***	Bavaria	Munich, Rangierbahnhof	48,1906	11,5269
BFB_Heteroptera_Schmolke_0490	**KM021870**	Nabidae	***Nabis brevis***	Bavaria	Rothenstein	48,9753	11,0589
BFB_Heteroptera_Schmolke_0491	**KM022454**	Nabidae	***Nabis brevis***	Bavaria	Duerrer Ast	48,3343	10,9093
BFB_Heteroptera_Schmolke_0492	**KM022299**	Nabidae	***Nabis brevis***	Bavaria	Munich, Korbinianihoelzl	48,2269	11,5444
EUBUG_1394_m_Nabibrev5	**KM022578**	Nabidae	***Nabis brevis***	Baden-Wuerttemberg	Swabian Alb	48,4900	9,3560
EUBUG_426_m_Nabibrev1	**KM022941**	Nabidae	***Nabis brevis***	Baden-Wuerttemberg	Swabian Alb	48,4900	9,3560
EUBUG_427_m_Nabibrev2	**KM022566**	Nabidae	***Nabis brevis***	Baden-Wuerttemberg	Swabian Alb	48,4900	9,3560
EUBUG_901_f_Nabibrev3	**KM022823**	Nabidae	***Nabis brevis***	Thuringia	Jena	50,9510	11,6240
EUBUG_932_f_Nabibrev4	**KM023048**	Nabidae	***Nabis brevis***	Thuringia	Jena	50,9510	11,6240
BFB_Heteroptera_Kuechler_0030	**KM022289**	Nabidae	***Nabis ericetorum***	Bavaria	Bayreuth, Destuben	49,9020	11,5680
BFB_Heteroptera_Schmolke_0495	**KM022639**	Nabidae	***Nabis ericetorum***	Bavaria	Lenggries, Sylvensteinsee	47,5950	11,5536
BFB_Heteroptera_Schmolke_0496	**KM022808**	Nabidae	***Nabis ericetorum***	Bavaria	Muehlhausen, Kastenmuehle	49,2183	11,4433
EUBUG_749_f_Nabieric1	**KM022278**	Nabidae	***Nabis ericetorum***	Rhineland Palatinate	Fischbach bei Dahn	49,1040	7,6830
EUBUG_750_m_Nabieric2	**KM022446**	Nabidae	***Nabis ericetorum***	Rhineland Palatinate	Fischbach bei Dahn	49,1040	7,6830
BFB_Heteroptera_Schmolke_0497	**KM021940**	Nabidae	***Nabis ferus***	Austria	Hainburg, Hundsheimer Berge	48,1268	16,9369

BFB_Heteroptera_Schmolke_0498	**KM021866**	Nabidae	***Nabis ferus***	Bavaria	Munich, Theresienwiese	48,1272	11,5464
EUBUG_210_m_Nabiferu1	**KM022270**	Nabidae	***Nabis ferus***	Brandenburg	Schorfheide-Chorin	53,1060	14,0180
EUBUG_220_m_Nabiferu2	**KM021673**	Nabidae	***Nabis ferus***	Brandenburg	Schorfheide-Chorin	53,1090	14,0090
EUBUG_221_m_Nabiferu3	**KM021521**	Nabidae	***Nabis ferus***	Brandenburg	Schorfheide-Chorin	53,1090	14,0090
BFB_Heteroptera_Schmolke_0485	**KM022694**	Nabidae	***Nabis flavomarginatus***	Bavaria	Oberndorf	48,9481	12,0269
BFB_Heteroptera_Schmolke_0486	**KM021695**	Nabidae	***Nabis flavomarginatus***	Bavaria	Maising	47,9828	11,2883
BFB_Heteroptera_Schmolke_0487	**KM021640**	Nabidae	***Nabis flavomarginatus***	Bavaria	Mailerberg	49,1842	11,9450
BFB_Heteroptera_Schmolke_0488	**KM021577**	Nabidae	***Nabis flavomarginatus***	Bavaria	Fahrenberg	47,6056	11,3142
EUBUG_385_f_Nabiflav1	**KM022389**	Nabidae	***Nabis flavomarginatus***	Thuringia	Hainich-Duen	51,2210	10,3670
EUBUG_394_m_Nabiflav3	**KM022914**	Nabidae	***Nabis flavomarginatus***	Thuringia	Hainich-Duen	51,3020	10,4360
EUBUG_547_f_Nabiflavo4	**KM021527**	Nabidae	***Nabis flavomarginatus***	Baden-Wuerttemberg	Pfrunger Ried	47,8990	9,3900
BFB_Heteroptera_Kuechler_0376	**KM022961**	Nabidae	***Nabis limbatus***	Italy	Follonica	42,9070	10,7760
BFB_Heteroptera_Schmolke_0483	**KM022439**	Nabidae	***Nabis limbatus***	Bavaria	Froettmaninger Heide	48,2114	11,6083
BFB_Heteroptera_Schmolke_0484	**KM022244**	Nabidae	***Nabis limbatus***	Bavaria	Mailerberg	49,1842	11,9450
EUBUG_102_f_Nabilimb6	**KM021971**	Nabidae	***Nabis limbatus***	Lower Saxony	Westerburg	53,2050	8,2460
EUBUG_1335_f_Nabilimb12	**KM021824**	Nabidae	***Nabis limbatus***	Thuringia	Hainich-Duen	51,3340	10,3680
EUBUG_1337_f_Nabilimb13	**KM022832**	Nabidae	***Nabis limbatus***	Thuringia	Hainich-Duen	51,0470	10,4910
EUBUG_720_f_Nabilimb7	**KM021705**	Nabidae	***Nabis limbatus***	Baden-Wuerttemberg	Pfrunger Ried	47,8990	9,3900
EUBUG_721_f_Nabilimb8	**KM022001**	Nabidae	***Nabis limbatus***	Baden-Wuerttemberg	Pfrunger Ried	47,8990	9,3900
EUBUG_722_m_Nabilimb9	**KM021863**	Nabidae	***Nabis limbatus***	Baden-Wuerttemberg	Pfrunger Ried	47,8990	9,3900
EUBUG_723_m_Nabilimb10	**KM022307**	Nabidae	***Nabis limbatus***	Baden-Wuerttemberg	Pfrunger Ried	47,8990	9,3900
EUBUG_729_f_Nabilimb11	**KM022527**	Nabidae	***Nabis limbatus***	Rhineland Palatinate	Fischbach bei Dahn	49,0860	7,7230
EUBUG_Seq15_f_Nabilimb	**KM021649**	Nabidae	***Nabis limbatus***	Baden-Wuerttemberg	Swabian Alb	48,3969	9,4348
BFB_Heteroptera_Kuechler_0019	**KM022625**	Nabidae	***Nabis pseudoferus***	Bavaria	Bayreuth, Botanical Garden	49,9230	11,5830
BFB_Heteroptera_Kuechler_0058	**KM022662**	Nabidae	***Nabis pseudoferus***	Bavaria	Bayreuth, Botanical Garden	49,9230	11,5830
BFB_Heteroptera_Kuechler_0059	**KM022511**	Nabidae	***Nabis pseudoferus***	Bavaria	Bayreuth, Botanical Garden	49,9230	11,5830
BFB_Heteroptera_Schmolke_0500	**KM021906**	Nabidae	***Nabis pseudoferus***	Bavaria	Froettmaninger Heide	48,2114	11,6083
BFB_Heteroptera_Schmolke_0501	**KM022654**	Nabidae	***Nabis pseudoferus***	Bavaria	Siegenburg, Dassfeld	48,7589	11,8383
BFB_Heteroptera_Schmolke_0502	**KM022405**	Nabidae	***Nabis pseudoferus***	Bavaria	Munich, northern Angerlohe	48,1914	11,4847
EUBUG_219_m_Nabipseu3	**KM021792**	Nabidae	***Nabis pseudoferus***	Brandenburg	Schorfheide-Chorin	53,0930	14,0060
EUBUG_252_m_Nabipseu4	**KM021947**	Nabidae	***Nabis pseudoferus***	Brandenburg	Schorfheide-Chorin	53,1350	13,8760
EUBUG_253_m_Nabipseu5	**KM022474**	Nabidae	***Nabis pseudoferus***	Brandenburg	Schorfheide-Chorin	53,1350	13,8760
EUBUG_667_f_Nabipseu6	**KM022014**	Nabidae	***Nabis pseudoferus***	Bavaria	Germering	48,1130	11,3880

EUBUG_668_m_Nabipseu7	**KM022576**	Nabidae	***Nabis pseudoferus***	Bavaria	Germering	48,1130	11,3880
BFB_Heteroptera_Kuechler_0056	**KM021627**	Nabidae	***Nabis rugosus***	Bavaria	Bayreuth, Botanical Garden	49,9230	11,5830
BFB_Heteroptera_Kuechler_0057	**KM022200**	Nabidae	***Nabis rugosus***	Bavaria	Bayreuth, Botanical Garden	49,9230	11,5830
BFB_Heteroptera_Schmolke_0333	**KM022040**	Nabidae	***Nabis rugosus***	Bavaria	Pollanten	49,1511	11,4433
BFB_Heteroptera_Schmolke_0505	**KM021554**	Nabidae	***Nabis rugosus***	Bavaria	Oberes Hafenlohrtal	49,8850	9,5697
EUBUG_834_m_Nabirugo5	**KM022874**	Nabidae	***Nabis rugosus***	Baden-Wuerttemberg	Limpach	49,1040	7,6830
EUBUG_835_f_Nabirugo6	**KM021852**	Nabidae	***Nabis rugosus***	Baden-Wuerttemberg	Limpach	49,1040	7,6830
BC ZSM AQU 00014	**HM376118**	Naucoridae	***Ilyocoris cimicoides***	Bavaria	Traunstein	48,0000	12,8400
BC ZSM AQU 00207	**KM022116**	Naucoridae	***Ilyocoris cimicoides***	Bavaria	Ansbach	49,0320	10,3690
BC ZSM AQU 00209	**HM421993**	Nepidae	***Nepa cinerea***	Bavaria	Weilheim-Schongau	47,7310	11,3120
BC ZSM AQU 00181	**HM376198**	Nepidae	***Ranatra linearis***	Bavaria	Ansbach	49,0320	10,3690
BC ZSM AQU 00711	**HQ563139**	Nepidae	***Ranatra linearis***	Bavaria	Schwandorf	49,0380	12,1870
BC ZSM AQU 00028	**HM376124**	Notonectidae	***Notonecta glauca***	Bavaria	Traunstein	48,0000	12,8400
BC ZSM AQU 00708	**HQ563136**	Notonectidae	***Notonecta glauca***	Bavaria	Freising	48,4860	11,9440
BFB_Heteroptera_Schmolke_001	**KM022276**	Notonectidae	***Notonecta glauca***	Bavaria	Munich	48,1906	11,5269
BFB_Heteroptera_Schmolke_0760	**KM023116**	Notonectidae	***Notonecta glauca***	Mecklenburg-Vorpommern	Himmelreichsee	53,1744	12,8411
BC ZSM AQU 00709	**HQ563137**	Notonectidae	***Notonecta lutea***	Bavaria	Rosenheim	47,8960	12,1660
BCZSMAQU001097	**KM022771**	Notonectidae	***Notonecta lutea***	Bavaria	Munich	48,2000	11,7200
BFB_Heteroptera_Schmolke_0567	**KM022160**	Notonectidae	***Notonecta lutea***	Mecklenburg-Vorpommern	Teufelssee	53,1833	12,8701
BFB_Heteroptera_Schmolke_0568	**KM021538**	Notonectidae	***Notonecta lutea***	Mecklenburg-Vorpommern	Teufelssee	53,1833	12,8701
BFB_Heteroptera_Schmolke_0750	**KM021973**	Notonectidae	***Notonecta lutea***	Brandenburg	Lychen, Kolbatzer Muehle	53,2431	13,3964
BFB_Heteroptera_Schmolke_0751	**KM021638**	Notonectidae	***Notonecta lutea***	Mecklenburg-Vorpommern	Teufelssee	53,1831	12,8700
BFB_Heteroptera_Schmolke_0752	**KM022145**	Notonectidae	***Notonecta lutea***	Mecklenburg-Vorpommern	Teufelssee	53,1831	12,8700
BFB_Heteroptera_Schmolke_0753	**KM022666**	Notonectidae	***Notonecta lutea***	Mecklenburg-Vorpommern	Teufelssee	53,1831	12,8700
BFB_Heteroptera_Schmolke_0755	**KM023055**	Notonectidae	***Notonecta lutea***	Mecklenburg-Vorpommern	Teufelssee	53,1831	12,8700
BC ZSM AQU 00710	**HQ563138**	Notonectidae	***Notonecta maculata***	Bavaria	Freising	48,4860	11,9440
BCZSMAQU001098	**KM022426**	Notonectidae	***Notonecta maculata***	Bavaria	Rosenheim	47,8900	12,3500
BFB_Heteroptera_Schmolke_002	**KM021672**	Notonectidae	***Notonecta maculata***	Bavaria	Munich	48,2008	11,4828
BFB_Heteroptera_Schmolke_003	**KM022642**	Notonectidae	***Notonecta maculata***	Bavaria	Munich	48,2114	11,6083
BFB_Heteroptera_Schmolke_0566	**KM021990**	Notonectidae	***Notonecta reuteri***	Mecklenburg-Vorpommern	Teufelssee	53,1833	12,8701
BFB_Heteroptera_Schmolke_0754	**KM021583**	Notonectidae	***Notonecta reuteri***	Mecklenburg-Vorpommern	Teufelssee	53,1831	12,8700
BFB_Heteroptera_Schmolke_0757	**KM022721**	Notonectidae	***Notonecta reuteri***	Mecklenburg-Vorpommern	Sanitz, Horster Moor	54,0503	12,4153
BFB_Heteroptera_Schmolke_0758	**KM021800**	Notonectidae	***Notonecta reuteri***	Mecklenburg-Vorpommern	Sanitz, Horster Moor	54,0503	12,4153

BCZSMAQU001099	**KM022030**	Notonectidae	***Notonecta viridis***	Bavaria	Dingolfing-Landau	48,5700	12,5100
BCZSMAQU001100	**KM022308**	Notonectidae	***Notonecta viridis***	Bavaria	Munich	48,2000	11,7200
BFB_Heteroptera_Schmolke_0715	**KM021982**	Oxycarenidae	***Macroplax preyssleri***	Bavaria	Wintershof	48,9067	11,1753
EUBUG_1422_f_Macrprey1	**KM022859**	Oxycarenidae	***Macroplax preyssleri***	Bavaria	Rosenau	48,6610	12,5800
EUBUG_Seq10_m_Macrprey	**KM021488**	Oxycarenidae	***Macroplax preyssleri***	Baden-Wuerttemberg	Swabian Alb	48,3914	9,3768
BFB_Heteroptera_Kuechler_0335	**KM022344**	Oxycarenidae	***Oxycarenus lavaterae***	France	La Garde-Freinet	43,3310	6,4740
BFB_Heteroptera_Kuechler_0336	**KM023038**	Oxycarenidae	***Oxycarenus lavaterae***	France	La Garde-Freinet	43,3310	6,4740
BFB_Heteroptera_Schmolke_0342	**KM021657**	Oxycarenidae	***Oxycarenus pallens***	Austria	Oggau	47,8754	16,7782
BFB_Heteroptera_Schmolke_0722	**KM021589**	Oxycarenidae	***Oxycarenus pallens***	Austria	Obersiebenbrunn	48,2259	16,6913
BC_ZSM_HETA_0885	**KM022512**	Oxycarenidae	***Tropidophlebia costalis***	Austria	Droesing, Waltersdorf, Noe	48,5289	16,8883
BFB_Heteroptera_Kuechler_0054	**KM022297**	Pentatomidae	***Aelia acuminata***	Bavaria	Bayreuth, Botanical Garden	49,9230	11,5830
BFB_Heteroptera_Kuechler_0055	**KM023101**	Pentatomidae	***Aelia acuminata***	Bavaria	Bayreuth, Botanical Garden	49,9230	11,5830
BFB_Heteroptera_Schmolke_0361	**KM022853**	Pentatomidae	***Aelia acuminata***	Bavaria	Pollanten	49,1511	11,4433
BFB_Heteroptera_Schmolke_0515	**KM021463**	Pentatomidae	***Aelia acuminata***	Brandenburg	Mallnow Oderhaenge	52,4701	14,4781
BFB_Heteroptera_Schmolke_0564	**KM022791**	Pentatomidae	***Aelia acuminata***	Brandenburg	Fuerstenberg/Spree, Loecknitztal	52,4317	13,8967
BFB_Heteroptera_Schmolke_0565	**KM022430**	Pentatomidae	***Aelia acuminata***	Brandenburg	Fuerstenberg/Spree, Loecknitztal	52,4317	13,8967
EUBUG_239_f_Aeliaccu1	**KM022514**	Pentatomidae	***Aelia acuminata***	Brandenburg	Schorfheide-Chorin	53,1350	13,8760
EUBUG_282_m_Aeliaccu2	**KM022056**	Pentatomidae	***Aelia acuminata***	Brandenburg	Schorfheide-Chorin	53,0870	13,9700
EUBUG_797_m_Aeliacum3	**KM022499**	Pentatomidae	***Aelia acuminata***	Brandenburg	Schorfheide-Chorin	47,8990	9,3900
EUBUG_893_m_Aeliacum6	**KM022425**	Pentatomidae	***Aelia acuminata***	Thuringia	Jena	50,9510	11,6240
EUBUG_770_m_Aeliklug1	**KM022034**	Pentatomidae	***Aelia klugii***	Thuringia	Hainich-Duen	47,8990	9,3900
BFB_Heteroptera_Kuechler_0353	**KM022736**	Pentatomidae	***Arma custos***	Bavaria	Creussen	49,8490	11,5960
BFB_Heteroptera_Schmolke_0364	**KM022090**	Pentatomidae	***Carpocoris fuscispinus***	Austria	Oggau	47,8754	16,7782
EUBUG_415_f_Carpfusc2	**KM023007**	Pentatomidae	***Carpocoris fuscispinus***	Thuringia	Hainich-Duen	51,2870	10,3800
EUBUG_734_m_Carpfusc5	**KM022597**	Pentatomidae	***Carpocoris fuscispinus***	Rhineland Palatinate	Fischbach bei Dahn	49,1040	7,6830
EUBUG_Seq29_f_Carpfusc	**KM021447**	Pentatomidae	***Carpocoris fuscispinus***	Thuringia	Hainich-Duen	51,2868	10,3801
BFB_Heteroptera_Kuechler_0104	**KM021778**	Pentatomidae	***Carpocoris purpureipennis***	Bavaria	Bayreuth, Botanical Garden	49,9230	11,5870
BFB_Heteroptera_Kuechler_0105	**KM022717**	Pentatomidae	***Carpocoris purpureipennis***	Bavaria	Bayreuth, Botanical Garden	49,9230	11,5870
BFB_Heteroptera_Kuechler_0180	**KM022063**	Pentatomidae	***Carpocoris purpureipennis***	Bavaria	Bayreuth, Botanical Garden	49,9230	11,5870
BFB_Heteroptera_Kuechler_0302	**KM022547**	Pentatomidae	***Carpocoris purpureipennis***	Rhineland Palatinate	Fischbach bei Dahn	49,0910	7,7150
BFB_Heteroptera_Schmolke_0365	**KM022450**	Pentatomidae	***Carpocoris purpureipennis***	Bavaria	Pollanten	49,1511	11,4433
BFB_Heteroptera_Kuechler_0288	**KM021680**	Pentatomidae	***Chlorochroa juniperina***	Bavaria	Gerolfinger Eichenwald	48,7560	11,3260
BFB_Heteroptera_Kuechler_0321	**KM022994**	Pentatomidae	***Chlorochroa pinicola***	Rhineland Palatinate	Fischbach bei Dahn	49,1290	7,6900

EUBUG_1116_m_Chlopini3	**KM022844**	Pentatomidae	***Chlorochroa pinicola***	Baden-Wuerttemberg	Fronreute	47,8600	9,5510
BFB_Heteroptera_Kuechler_0029	**KM021658**	Pentatomidae	***Dolycoris baccarum***	Bavaria	Destuben	49,9020	11,5680
BFB_Heteroptera_Schmolke_0534	**KM022268**	Pentatomidae	***Dolycoris baccarum***	Brandenburg	Berlin, Spandau	52,5221	13,1902
EUBUG_240_f_Dolybacc1	**KM022158**	Pentatomidae	***Dolycoris baccarum***	Brandenburg	Schorfheide-Chorin	53,1350	13,8760
EUBUG_273_m_Dolybacc2	**KM022845**	Pentatomidae	***Dolycoris baccarum***	Brandenburg	Schorfheide-Chorin	53,0890	13,9800
EUBUG_299_f_Dolybacc2	**KM022727**	Pentatomidae	***Dolycoris baccarum***	Thuringia	Hainich-Duen	51,0900	10,4620
EUBUG_300_m_Dolybacc3	**KM022775**	Pentatomidae	***Dolycoris baccarum***	Thuringia	Hainich-Duen	51,0900	10,4620
EUBUG_301_m_Dolybacc4	**KM022004**	Pentatomidae	***Dolycoris baccarum***	Thuringia	Hainich-Duen	51,0900	10,4620
EUBUG_302_f_Dolybacc5	**KM022809**	Pentatomidae	***Dolycoris baccarum***	Thuringia	Hainich-Duen	51,0900	10,4620
BFB_Heteroptera_Kuechler_0265	**KM022693**	Pentatomidae	***Eurydema dominulus***	Bavaria	Paradiestal bei Treunitz	49,9830	11,1890
BFB_Heteroptera_Kuechler_0083	**KM022032**	Pentatomidae	***Eurydema oleracea***	Bavaria	Bayreuth, Botanical Garden	49,9230	11,5830
BFB_Heteroptera_Kuechler_0084	**KM022673**	Pentatomidae	***Eurydema oleracea***	Bavaria	Bayreuth, Botanical Garden	49,9230	11,5830
BFB_Heteroptera_Schmolke_0367	**KM021709**	Pentatomidae	***Eurydema oleracea***	Bavaria	Pollanten	49,1511	11,4433
BFB_Heteroptera_Schmolke_0561	**KM021944**	Pentatomidae	***Eurydema oleracea***	Brandenburg	Fuerstenberg/Spree, Loecknitztal	52,4317	13,8967
BFB_Heteroptera_Schmolke_0562	**KM022053**	Pentatomidae	***Eurydema oleracea***	Brandenburg	Fuerstenberg/Spree, Loecknitztal	52,4317	13,8967
BFB_Heteroptera_Schmolke_0563	**KM021679**	Pentatomidae	***Eurydema oleracea***	Brandenburg	Fuerstenberg/Spree, Loecknitztal	52,4317	13,8967
BFB_Heteroptera_Kuechler_0324	**KM022254**	Pentatomidae	***Eurydema ornata***	France	La Garde-Freinet	43,3300	6,4700
BFB_Heteroptera_Kuechler_0325	**KM022292**	Pentatomidae	***Eurydema ornata***	France	La Garde-Freinet	43,3300	6,4700
BFB_Heteroptera_Kuechler_0290	**KM022606**	Pentatomidae	***Eysarcoris aeneus***	Bavaria	Kulmbach, Kainach	49,9590	11,2860
EUBUG_858_m_Eysaaene1	**KM021998**	Pentatomidae	***Eysarcoris aeneus***	Rhineland Palatinate	Fischbach bei Dahn	49,0860	7,7230
EUBUG_859_m_Eysaaene2	**KM022573**	Pentatomidae	***Eysarcoris aeneus***	Rhineland Palatinate	Fischbach bei Dahn	49,0860	7,7230
EUBUG_860_m_Eysaaene3	**KM022729**	Pentatomidae	***Eysarcoris aeneus***	Rhineland Palatinate	Fischbach bei Dahn	49,0860	7,7230
EUBUG_861_f_Eysaaene4	**KM022068**	Pentatomidae	***Eysarcoris aeneus***	Rhineland Palatinate	Fischbach bei Dahn	49,0860	7,7230
BFB_Heteroptera_Kuechler_0181	**KM021471**	Pentatomidae	***Eysarcoris venustissimus***	Bavaria	Bayreuth, Botanical Garden	49,9230	11,5870
BFB_Heteroptera_Kuechler_0182	**KM022722**	Pentatomidae	***Eysarcoris venustissimus***	Bavaria	Bayreuth, Botanical Garden	49,9230	11,5870
BFB_Heteroptera_Kuechler_0177	**KM022369**	Pentatomidae	***Graphosoma lineatum***	Bavaria	Bayreuth, Botanical Garden	49,9230	11,5870
BFB_Heteroptera_Kuechler_0178	**KM022779**	Pentatomidae	***Graphosoma lineatum***	Bavaria	Bayreuth, Botanical Garden	49,9230	11,5870
BFB_Heteroptera_Schmolke_0368	**KM022404**	Pentatomidae	***Graphosoma lineatum***	Bavaria	Pollanten	49,1511	11,4433
BFB_Heteroptera_Schmolke_0569	**KM022214**	Pentatomidae	***Graphosoma lineatum***	Brandenburg	Fuerstenberg/Spree, Loecknitztal	52,4317	13,8967
BFB_Heteroptera_Schmolke_0570	**KM021891**	Pentatomidae	***Graphosoma lineatum***	Brandenburg	Fuerstenberg/Spree, Loecknitztal	52,4317	13,8967
EUBUG_401_f_Grapline1	**KM021728**	Pentatomidae	***Graphosoma lineatum***	Thuringia	Hainich-Duen	51,0230	10,3220
EUBUG_402_m_Grapline2	**KM022702**	Pentatomidae	***Graphosoma lineatum***	Thuringia	Hainich-Duen	51,0230	10,3220
EUBUG_403_m_Grapline3	**KM021885**	Pentatomidae	***Graphosoma lineatum***	Thuringia	Hainich-Duen	51,0230	10,3220

EUBUG_523_m_Grapline4	**KM021441**	Pentatomidae	***Graphosoma lineatum***	Lower Saxony	Wahnbek	53,2080	8,2310
BFB_Heteroptera_Kuechler_0099	**KM022937**	Pentatomidae	***Holcostethus strictus***	Bavaria	Saas/Bayreuth	49,9110	11,5520
BFB_Heteroptera_Kuechler_0320	**KM021723**	Pentatomidae	***Holcostethus strictus***	Rhineland Palatinate	Fischbach bei Dahn	49,1290	7,6900
BFB_Heteroptera_Schmolke_0533	**KM021830**	Pentatomidae	***Holcostethus strictus***	Brandenburg	Berlin, Spandau	52,5221	13,1902
EUBUG_384_f_Perivern1	**KM022213**	Pentatomidae	***Holcostethus strictus***	Thuringia	Hainich-Duen	51,1910	10,7530
BFB_Heteroptera_Schmolke_0362	**KM022331**	Pentatomidae	***Neottiglossa leporina***	Bavaria	Pollanten	49,1511	11,4433
BFB_Heteroptera_Schmolke_0363	**KM022613**	Pentatomidae	***Neottiglossa leporina***	Austria	Oggau	47,8754	16,7782
BFB_Heteroptera_Kuechler_0012	**KM022889**	Pentatomidae	***Neottiglossa pusilla***	Bavaria	Bayreuth, Botanical Garden	49,9230	11,5830
BFB_Heteroptera_Kuechler_0026	**KM021925**	Pentatomidae	***Neottiglossa pusilla***	Bavaria	Bayreuth, Botanical Garden	49,9230	11,5830
BFB_Heteroptera_Kuechler_0024	**KM021472**	Pentatomidae	***Palomena prasina***	Bavaria	Bayreuth, Botanical Garden	49,9240	11,5840
BFB_Heteroptera_Kuechler_0025	**KM022826**	Pentatomidae	***Palomena prasina***	Bavaria	Bayreuth, Botanical Garden	49,9240	11,5840
BFB_Heteroptera_Schmolke_0527	**KM022897**	Pentatomidae	***Palomena prasina***	Brandenburg	Berlin, Spandau	52,5221	13,1902
EUBUG_10_m_Palopras5	**KM021951**	Pentatomidae	***Palomena prasina***	Bavaria	Neuburg an der Donau	48,7380	11,2760
EUBUG_110_juv_Palopras6	**KM022107**	Pentatomidae	***Palomena prasina***	Lower Saxony	Westerburg	53,2050	8,2460
EUBUG_6_m_Palopras1	**KM022428**	Pentatomidae	***Palomena prasina***	Bavaria	Neuburg an der Donau	48,7390	11,2780
EUBUG_657_juv_Pentsp2	**KM022229**	Pentatomidae	***Palomena prasina***	Brandenburg	Kuestrin	52,5720	14,6270
EUBUG_727_m_Palopras7	**KM021668**	Pentatomidae	***Palomena prasina***	Rhineland Palatinate	Fischbach bei Dahn	49,0860	7,7230
BFB_Heteroptera_Kuechler_0214	**KM022353**	Pentatomidae	***Pentatoma rufipes***	Bavaria	Bayreuth, Botanical Garden	49,9230	11,5850
EUBUG_103_m_Pentrufi7	**KM022121**	Pentatomidae	***Pentatoma rufipes***	Rhineland Palatinate	Rolandswerth	50,6460	7,2030
EUBUG_104_m_Pentrufi1	**KM021544**	Pentatomidae	***Pentatoma rufipes***	Bavaria	Neuburg an der Donau	48,7420	11,2800
EUBUG_105_m_Pentrufi2	**KM022750**	Pentatomidae	***Pentatoma rufipes***	Bavaria	Neuburg an der Donau	48,7420	11,2800
EUBUG_106_m_Pentrufi3	**KM021597**	Pentatomidae	***Pentatoma rufipes***	Bavaria	Neuburg an der Donau	48,7420	11,2800
EUBUG_107_m_Pentrufi4	**KM021648**	Pentatomidae	***Pentatoma rufipes***	Bavaria	Neuburg an der Donau	48,7420	11,2800
EUBUG_108_m_Pentrufi5	**KM022368**	Pentatomidae	***Pentatoma rufipes***	Bavaria	Neuburg an der Donau	48,7420	11,2800
EUBUG_109_f_Pentrufi6	**KM021725**	Pentatomidae	***Pentatoma rufipes***	Bavaria	Neuburg an der Donau	48,7490	11,2790
BFB_Heteroptera_Kuechler_0351	**KM021458**	Pentatomidae	***Picromerus bidens***	Italy	Talamone	42,5610	11,1390
BFB_Heteroptera_Kuechler_0352	**KM021896**	Pentatomidae	***Picromerus bidens***	Italy	Talamone	42,5610	11,1390
BFB_Heteroptera_Kuechler_0032	**KM021760**	Pentatomidae	***Piezodorus lituratus***	Bavaria	Destuben	49,9020	11,5680
BFB_Heteroptera_Kuechler_0224	**KM022892**	Pentatomidae	***Piezodorus lituratus***	Bavaria	Bayreuth, Botanical Garden	49,9230	11,5850
BFB_Heteroptera_Schmolke_0366	**KM022391**	Pentatomidae	***Piezodorus lituratus***	Bavaria	Pollanten	49,1511	11,4433
EUBUG_298_m_Piezlitu1	**KM022101**	Pentatomidae	***Piezodorus lituratus***	Thuringia	Hainich-Duen	51,0900	10,4620
EUBUG_418_f_Piezlitu1	**KM022066**	Pentatomidae	***Piezodorus lituratus***	Thuringia	Hainich-Duen	51,0230	10,3220
EUBUG_419_m_Piezlitu2	**KM021724**	Pentatomidae	***Piezodorus lituratus***	Thuringia	Hainich-Duen	51,0230	10,3220

EUBUG_889_f_Podoinun1	**KM023102**	Pentatomidae	***Podops inunctus***	Thuringia	Jena	50,9510	11,6240
BFB_Heteroptera_Kuechler_0227	**KM022180**	Pentatomidae	***Rhacognathus punctatus***	Bavaria	Bayreuth, Botanical Garden	49,9230	11,5850
BFB_Heteroptera_Kuechler_0038	**KM022255**	Pentatomidae	***Rhaphigaster nebulosa***	Bavaria	Bayreuth, University	49,9290	11,5830
BFB_Heteroptera_Kuechler_0039	**KM022252**	Pentatomidae	***Rhaphigaster nebulosa***	Bavaria	Bayreuth, University	49,9290	11,5830
EUBUG_1347_m_Rhapnebu3	**KM023025**	Pentatomidae	***Rhaphigaster nebulosa***	Bavaria	Freising	48,4010	11,7170
EUBUG_1348_m_Rhapnebu4	**KM022652**	Pentatomidae	***Rhaphigaster nebulosa***	Bavaria	Freising	48,4010	11,7170
EUBUG_1349_m_Rhapnebu5	**KM021698**	Pentatomidae	***Rhaphigaster nebulosa***	Bavaria	Freising	48,4010	11,7170
EUBUG_1350_f_Rhapnebu6	**KM022520**	Pentatomidae	***Rhaphigaster nebulosa***	Bavaria	Freising	48,4010	11,7170
EUBUG_1351_f_Rhapnebu7	**KM021634**	Pentatomidae	***Rhaphigaster nebulosa***	Bavaria	Freising	48,4010	11,7170
EUBUG_157_m_Rhapnebu2	**KM021580**	Pentatomidae	***Rhaphigaster nebulosa***	Rhineland Palatinate	Rolandswerth	50,6460	7,2030
BFB_Heteroptera_Kuechler_0295	**KM021444**	Pentatomidae	***Sciocoris cursitans***	Rhineland Palatinate	Fischbach bei Dahn	49,0910	7,7150
BFB_Heteroptera_Kuechler_0296	**KM022468**	Pentatomidae	***Sciocoris cursitans***	Rhineland Palatinate	Fischbach bei Dahn	49,0910	7,7150
BFB_Heteroptera_Schmolke_0535	**KM023063**	Pentatomidae	***Sciocoris cursitans***	Brandenburg	Berlin, Spandau	52,5221	13,1902
EUBUG_449_f_Sciocurs1	**KM022433**	Pentatomidae	***Sciocoris cursitans***	Baden-Wuerttemberg	Münsingen	48,4220	9,4990
EUBUG_871_f_Sciocurs2	**KM022628**	Pentatomidae	***Sciocoris cursitans***	Rhineland Palatinate	Fischbach bei Dahn	49,0900	7,7150
BFB_Heteroptera_Kuechler_0275	**KM022427**	Pentatomidae	***Sciocoris homalonotus***	Bavaria	Bayreuth, Botanical Garden	49,9230	11,5850
BFB_Heteroptera_Kuechler_0187	**KM022202**	Pentatomidae	***Sciocoris umbrinus***	Bavaria	Staffelberg	50,0920	11,0240
BFB_Heteroptera_Kuechler_0033	**KM022544**	Pentatomidae	***Troilus luridus***	Bavaria	Destuben	49,9020	11,5680
EUBUG_1330_m_Troiluri2	**KM021946**	Pentatomidae	***Troilus luridus***	Thuringia	Hainich-Duen	51,3370	10,3590
EUBUG_857_f_Zicrcaer1	**KM023126**	Pentatomidae	***Zicrona caerulea***	Rhineland Palatinate	Fischbach bei Dahn	49,0860	7,7230
BFB_Heteroptera_Kuechler_0107	**KM022437**	Phymatidae	***Phymata crassipes***	Bavaria	Bayreuth, Neubuerg	49,8920	11,4020
BFB_Heteroptera_Schmolke_0336	**KM022422**	Phymatidae	***Phymata crassipes***	Bavaria	Kelheimwinzer	48,9175	11,9178
BFB_Heteroptera_Schmolke_0622	**KM022466**	Phymatidae	***Phymata crassipes***	Bavaria	Kallmuenz	49,1871	11,9641
BFB_Heteroptera_Schmolke_0623	**KM021865**	Phymatidae	***Phymata crassipes***	Bavaria	Kelheimwinzer	48,9175	11,9178
BFB_Heteroptera_Schmolke_0624	**KM022184**	Phymatidae	***Phymata crassipes***	Bavaria	Schernfeld	48,9114	11,1164
GBOL_Col_FK_4059	**KM021734**	Phymatidae	***Phymata crassipes***	Rhineland Palatinate	Bad Kreuznach	49,9020	7,8650
BC_ZSM_HETA_0886	**KM022954**	Piesmatidae	***Parapiesma quadratum***	Austria	Podersdorf, Seewinkel	47,8597	16,8352
BC_ZSM_HETA_0888	**KM023000**	Piesmatidae	***Piesma maculatum***	Bavaria	Offenstetten	48,8111	11,9106
BC_ZSM_HETA_0889	**KM021932**	Piesmatidae	***Piesma maculatum***	Austria	Droesing, Waltersdorf, Noe	48,5289	16,8883
BFB_Heteroptera_Kuechler_0379	**KM022635**	Piesmatidae	***Piesma maculatum***	Bavaria	Bayreuth, Botanical Garden	49,9230	11,5850
BFB_Heteroptera_Kuechler_0380	**KM022631**	Piesmatidae	***Piesma maculatum***	Bavaria	Bayreuth	49,9260	11,5620
EUBUG_346_m_Piesmacu1	**KM022894**	Piesmatidae	***Piesma maculatum***	Baden-Wuerttemberg	Fronreute	47,8450	9,6100
EUBUG_572_f_Piesmmacu2	**KM022365**	Piesmatidae	***Piesma maculatum***	Bavaria	Bavarian Forest, Koetzting	49,1820	12,8440

EUBUG_573_f_Piesmmacu3	**KM021494**	Piesmatidae	***Piesma maculatum***	Bavaria	Bavarian Forest, Koetzting	49,1820	12,8440
EUBUG_574_m_Piesmmacu4	**KM021964**	Piesmatidae	***Piesma maculatum***	Bavaria	Bavarian Forest, Koetzting	49,1820	12,8440
EUBUG_575_m_Piesmmacu5	**KM022479**	Piesmatidae	***Piesma maculatum***	Bavaria	Bavarian Forest, Koetzting	49,1820	12,8440
EUBUG_576_m_Piesmmacu6	**KM022473**	Piesmatidae	***Piesma maculatum***	Bavaria	Bavarian Forest, Koetzting	49,1820	12,8440
BFB_Heteroptera_Kuechler_0126	**KM022607**	Plataspidae	***Coptosoma scutellatum***	Bavaria	Bayreuth, Hohenmirsberger-Platte	49,8150	11,4450
BFB_Heteroptera_Schmolke_0357	**KM023090**	Plataspidae	***Coptosoma scutellatum***	Bavaria	Pollanten	49,1511	11,4433
BC ZSM AQU 00022	**GU682184**	Pleidae	***Plea minutissima***	Bavaria	Traunstein	48,0000	12,8400
BC_ZSM_HETA_0908	**KM021804**	Pyrrhocoridae	***Pyrrhocoris apterus***	Bavaria	Munich, Moosach	48,1808	11,5244
BC_ZSM_HETA_0909	**KM022504**	Pyrrhocoridae	***Pyrrhocoris apterus***	Bavaria	Traunstein	47,8749	12,6336
BC_ZSM_HETA_0911	**KM021717**	Pyrrhocoridae	***Pyrrhocoris apterus***	Bavaria	Allacher Heide	48,2008	11,4828
BFB_Heteroptera_Kuechler_0002	**KM023058**	Pyrrhocoridae	***Pyrrhocoris apterus***	Bavaria	Bayreuth	49,9260	11,5620
BFB_Heteroptera_Kuechler_0003	**KM021837**	Pyrrhocoridae	***Pyrrhocoris apterus***	Bavaria	Bayreuth	49,9260	11,5620
BFB_Heteroptera_Schmolke_0521	**KM021457**	Pyrrhocoridae	***Pyrrhocoris apterus***	Brandenburg	Berlin, Spandau	52,5221	13,1902
EUBUG_100_m_Phyrapte5	**KM022550**	Pyrrhocoridae	***Pyrrhocoris apterus***	North Rhine-Westphalia	Bonn, Mehlem	50,6620	7,1910
EUBUG_515_m_Pyrrapte6	**KM022091**	Pyrrhocoridae	***Pyrrhocoris apterus***	North Rhine-Westphalia	Oer-Erkenschwick	51,6440	7,2660
EUBUG_521_m_Pyrrapte7	**KM022919**	Pyrrhocoridae	***Pyrrhocoris apterus***	Bavaria	Regensburg, Uni-Campus	48,9980	12,0950
EUBUG_522_f_Pyrrapte8	**KM021878**	Pyrrhocoridae	***Pyrrhocoris apterus***	Bavaria	Regensburg, Uni-Campus	48,9980	12,0950
EUBUG_96_m_Phyrapte1	**KM022114**	Pyrrhocoridae	***Pyrrhocoris apterus***	North Rhine-Westphalia	Bonn, Mehlem	50,6620	7,1910
EUBUG_97_m_Phyrapte2	**KM021508**	Pyrrhocoridae	***Pyrrhocoris apterus***	North Rhine-Westphalia	Bonn, Mehlem	50,6620	7,1910
EUBUG_98_m_Phyrapte3	**KM022991**	Pyrrhocoridae	***Pyrrhocoris apterus***	North Rhine-Westphalia	Bonn, Mehlem	50,6620	7,1910
EUBUG_99_m_Phyrapte4	**KM022759**	Pyrrhocoridae	***Pyrrhocoris apterus***	North Rhine-Westphalia	Bonn, Mehlem	50,6620	7,1910
BFB_Heteroptera_Schmolke_0614	**KM022149**	Reduviidae	***Coranus subapterus***	Bavaria	Munich, Truderinger Wald	48,0933	11,6683
EUBUG_1421_m_Corasuba1	**KM022891**	Reduviidae	***Coranus subapterus***	Bavaria	Rosenau	48,6610	12,5800
BFB_Heteroptera_Kuechler_0368	**KM021474**	Reduviidae	***Empicoris vagabundus***	Bavaria	Bayreuth, Schlehenmuehle	49,9050	11,6240
EUBUG_524_f_Empivaga1	**KM022640**	Reduviidae	***Empicoris vagabundus***	Baden-Wuerttemberg	Pfrunger Ried	47,8990	9,3900
EUBUG_795_juv_Redupers1	**KM022945**	Reduviidae	***Reduvius personatus***	Baden-Wuerttemberg	Fronreute	47,8990	9,3900
BFB_Heteroptera_Kuechler_0103	**KM022667**	Reduviidae	***Rhynocoris annulatus***	Bavaria	Bayreuth, Botanical Garden	49,9230	11,5870
BFB_Heteroptera_Schmolke_0616	**KM022962**	Reduviidae	***Rhynocoris annulatus***	Bavaria	Kirchseemoor	47,8008	11,6025
BFB_Heteroptera_Schmolke_0618	**KM022134**	Reduviidae	***Rhynocoris annulatus***	Bavaria	Elsendorf	48,7164	11,7981
BFB_Heteroptera_Schmolke_0619	**KM022128**	Reduviidae	***Rhynocoris iracundus***	Bavaria	Obereichstaett	48,8978	11,1228
BFB_Heteroptera_Schmolke_0620	**KM021855**	Reduviidae	***Rhynocoris iracundus***	Bavaria	Kirchseemoor	47,8008	11,6025
BFB_Heteroptera_Schmolke_0621	**KM022088**	Reduviidae	***Rhynocoris iracundus***	Bavaria	Hasenheide	49,2983	11,4546
BFB_Heteroptera_Kuechler_0118	**KM021875**	Rhopalidae	***Brachycarenus tigrinus***	Bavaria	Bayreuth, Botanical Garden	49,9230	11,5830

BFB_Heteroptera_Kuechler_0322	**KM022117**	Rhopalidae	***Chlorosoma schillingii***	Rhineland Palatinate	Fischbach bei Dahn	49,1290	7,6900
BFB_Heteroptera_Kuechler_0090	**KM022552**	Rhopalidae	***Corizus hyoscyami***	Bavaria	Bayreuth, University	49,9290	11,5830
BFB_Heteroptera_Kuechler_0106	**KM021784**	Rhopalidae	***Corizus hyoscyami***	Bavaria	Bayreuth, Botanical Garden	49,9230	11,5870
BFB_Heteroptera_Schmolke_0528	**KM021975**	Rhopalidae	***Corizus hyoscyami***	Brandenburg	Berlin, Spandau	52,5221	13,1902
EUBUG_732_f_Corihyos3	**KM021674**	Rhopalidae	***Corizus hyoscyami***	Rhineland Palatinate	Fischbach bei Dahn	49,1040	7,6830
EUBUG_869_f_Corihyos4	**KM022565**	Rhopalidae	***Corizus hyoscyami***	Rhineland Palatinate	Fischbach bei Dahn	49,0900	7,7150
EUBUG_899_f_Corihyos4	**KM022350**	Rhopalidae	***Corizus hyoscyami***	Thuringia	Jena	50,9510	11,6240
EUBUG_922_f_Corihyos5	**KM021889**	Rhopalidae	***Corizus hyoscyami***	Thuringia	Jena	50,9510	11,6240
EUBUG_923_f_Corihyos6	**KM023093**	Rhopalidae	***Corizus hyoscyami***	Thuringia	Jena	50,9510	11,6240
BFB_Heteroptera_Kuechler_0316	**KM021653**	Rhopalidae	***Liorhyssus hyalinus***	Rhineland Palatinate	Fischbach bei Dahn	49,1290	7,6900
BFB_Heteroptera_Kuechler_0323	**KM023097**	Rhopalidae	***Liorhyssus hyalinus***	Rhineland Palatinate	Fischbach bei Dahn	49,1290	7,6900
EUBUG_918_m_Liorhyal3	**KM022038**	Rhopalidae	***Liorhyssus hyalinus***	Thuringia	Jena	50,9510	11,6240
BFB_Heteroptera_Kuechler_0193	**KM021635**	Rhopalidae	***Myrmus miriformis***	Bavaria	Bayreuth, Botanical Garden	49,9230	11,5850
BFB_Heteroptera_Kuechler_0194	**KM022910**	Rhopalidae	***Myrmus miriformis***	Bavaria	Bayreuth, Botanical Garden	49,9230	11,5850
BFB_Heteroptera_Schmolke_0353	**KM021754**	Rhopalidae	***Myrmus miriformis***	Bavaria	Siegenburg	48,7589	11,8383
EUBUG_245_f_Myrmmiri1	**KM022588**	Rhopalidae	***Myrmus miriformis***	Brandenburg	Schorfheide-Chorin	53,1350	13,8760
EUBUG_287_m_Myrmmiri1	**KM023089**	Rhopalidae	***Myrmus miriformis***	Brandenburg	Schorfheide-Chorin	53,0880	13,9770
EUBUG_872_m_Myrmmiri3	**KM022570**	Rhopalidae	***Myrmus miriformis***	Rhineland Palatinate	Fischbach bei Dahn	49,0900	7,7150
EUBUG_873_m_Myrmmiri4	**KM022210**	Rhopalidae	***Myrmus miriformis***	Rhineland Palatinate	Fischbach bei Dahn	49,0900	7,7150
BFB_Heteroptera_Kuechler_0301	**KM021793**	Rhopalidae	***Rhopalus conspersus***	Rhineland Palatinate	Fischbach bei Dahn	49,0910	7,7150
BFB_Heteroptera_Kuechler_0071	**KM021712**	Rhopalidae	***Rhopalus maculatus***	Bavaria	Bayreuth, Creussen	49,8490	11,5960
BFB_Heteroptera_Kuechler_0297	**KM021523**	Rhopalidae	***Rhopalus maculatus***	Rhineland Palatinate	Fischbach bei Dahn	49,0910	7,7150
BFB_Heteroptera_Kuechler_0298	**KM023131**	Rhopalidae	***Rhopalus maculatus***	Rhineland Palatinate	Fischbach bei Dahn	49,0910	7,7150
BFB_Heteroptera_Kuechler_0088	**KM022279**	Rhopalidae	***Rhopalus parumpunctatus***	Bavaria	Bayreuth, Botanical Garden	49,9230	11,5830
BFB_Heteroptera_Kuechler_0129	**KM021817**	Rhopalidae	***Rhopalus parumpunctatus***	Bavaria	Bayreuth, Hohenmirsberger-Platte	49,8150	11,4450
BFB_Heteroptera_Schmolke_0356	**KM022339**	Rhopalidae	***Rhopalus parumpunctatus***	Bavaria	Pollanten	49,1511	11,4433
BFB_Heteroptera_Schmolke_0522	**KM023029**	Rhopalidae	***Rhopalus parumpunctatus***	Brandenburg	Berlin, Spandau	52,5221	13,1902
EUBUG_241_m_Rhopparu1	**KM022841**	Rhopalidae	***Rhopalus parumpunctatus***	Brandenburg	Schorfheide-Chorin	53,1350	13,8760
EUBUG_242_m_Rhopparu2	**KM021484**	Rhopalidae	***Rhopalus parumpunctatus***	Brandenburg	Schorfheide-Chorin	53,1350	13,8760
EUBUG_243_f_Rhopparu3	**KM021957**	Rhopalidae	***Rhopalus parumpunctatus***	Brandenburg	Schorfheide-Chorin	53,1350	13,8760
EUBUG_900_f_Rhopparu4	**KM021864**	Rhopalidae	***Rhopalus parumpunctatus***	Thuringia	Jena	50,9510	11,6240
BFB_Heteroptera_Kuechler_0013	**KM021977**	Rhopalidae	***Rhopalus subrufus***	Bavaria	Bayreuth, Botanical Garden	49,9230	11,5830
EUBUG_1334_f_Rhopsubr2	**KM022189**	Rhopalidae	***Rhopalus subrufus***	Thuringia	Hainich-Duen	51,1420	10,3770

BFB_Heteroptera_Kuechler_0089	**KM022564**	Rhopalidae	***Stictopleurus abutilon***	Bavaria	Bayreuth, Botanical Garden	49,9230	11,5830
BFB_Heteroptera_Kuechler_0317	**KM022638**	Rhopalidae	***Stictopleurus abutilon***	Rhineland Palatinate	Fischbach bei Dahn	49,1290	7,6900
BFB_Heteroptera_Schmolke_0354	**KM022743**	Rhopalidae	***Stictopleurus abutilon***	Bavaria	Pollanten	49,1511	11,4433
EUBUG_267_f_Sticabut1	**KM022342**	Rhopalidae	***Stictopleurus abutilon***	Brandenburg	Schorfheide-Chorin	53,1350	13,8760
EUBUG_268_m_Sticabut2	**KM021808**	Rhopalidae	***Stictopleurus abutilon***	Brandenburg	Schorfheide-Chorin	53,1350	13,8760
EUBUG_269_f_Sticabut3	**KM021500**	Rhopalidae	***Stictopleurus abutilon***	Brandenburg	Schorfheide-Chorin	53,1350	13,8760
BFB_Heteroptera_Kuechler_0299	**KM022609**	Rhopalidae	***Stictopleurus crassicornis***	Rhineland Palatinate	Fischbach bei Dahn	49,0910	7,7150
BFB_Heteroptera_Kuechler_0300	**KM021575**	Rhopalidae	***Stictopleurus crassicornis***	Rhineland Palatinate	Fischbach bei Dahn	49,0910	7,7150
EUBUG_446_f_Sticcras1	**KM021598**	Rhopalidae	***Stictopleurus crassicornis***	Baden-Wuerttemberg	Schwaebische Alb	48,3950	9,2600
EUBUG_757_f_Sticpict1	**KM022241**	Rhopalidae	***Stictopleurus crassicornis***	Rhineland Palatinate	Fischbach bei Dahn	49,1040	7,6830
EUBUG_874_f_Sticcras2	**KM022411**	Rhopalidae	***Stictopleurus crassicornis***	Rhineland Palatinate	Fischbach bei Dahn	49,0900	7,7150
EUBUG_875_f_Sticcras3	**KM021430**	Rhopalidae	***Stictopleurus crassicornis***	Rhineland Palatinate	Fischbach bei Dahn	49,0900	7,7150
EUBUG_876_f_Sticcras4	**KM022758**	Rhopalidae	***Stictopleurus crassicornis***	Rhineland Palatinate	Fischbach bei Dahn	49,0900	7,7150
EUBUG_877_m_Sticcras5	**KM021676**	Rhopalidae	***Stictopleurus crassicornis***	Rhineland Palatinate	Fischbach bei Dahn	49,0900	7,7150
BFB_Heteroptera_Kuechler_0311	**KM022691**	Rhopalidae	***Stictopleurus pictus***	Bavaria	Bayreuth, Botanical Garden	49,9230	11,5850
EUBUG_758_m_Sticpict2	**KM022183**	Rhopalidae	***Stictopleurus pictus***	Rhineland Palatinate	Fischbach bei Dahn	49,1040	7,6830
BFB_Heteroptera_Kuechler_0209	**KM021685**	Rhopalidae	***Stictopleurus punctatonervosus***	Bavaria	Bayreuth, Botanical Garden	49,9230	11,5850
BFB_Heteroptera_Kuechler_0318	**KM022621**	Rhopalidae	***Stictopleurus punctatonervosus***	Rhineland Palatinate	Fischbach bei Dahn	49,1290	7,6900
BFB_Heteroptera_Kuechler_0319	**KM021849**	Rhopalidae	***Stictopleurus punctatonervosus***	Rhineland Palatinate	Fischbach bei Dahn	49,1290	7,6900
BFB_Heteroptera_Schmolke_0355	**KM021551**	Rhopalidae	***Stictopleurus punctatonervosus***	Bavaria	Pollanten	49,1511	11,4433
EUBUG_244_m_Sticpunc1	**KM022060**	Rhopalidae	***Stictopleurus punctatonervosus***	Brandenburg	Schorfheide-Chorin	53,1350	13,8760
BC_ZSM_HETA_0869	**KM021894**	Rhyparochromidae	***Acompus rufipes***	Austria	Walchsee in Tirol	47,6564	12,3177
BC_ZSM_HETA_0870	**KM022507**	Rhyparochromidae	***Acompus rufipes***	Bavaria	Uebersee	47,8381	12,4778
BC_ZSM_HETA_0871	**KM023070**	Rhyparochromidae	***Acompus rufipes***	Bavaria	Ruhpolding, Brand	47,7406	12,5942
BFB_Heteroptera_Kuechler_0073	**KM021678**	Rhyparochromidae	***Acompus rufipes***	Bavaria	Bayreuth, Botanical Garden	49,9230	11,5830
BC_ZSM_HETA_0827	**KM022929**	Rhyparochromidae	***Aellopus atratus***	Bavaria	Volkach, Astheim	49,8547	10,2086
BC_ZSM_HETA_0831	**KM021541**	Rhyparochromidae	***Beosus maritimus***	Bavaria	Bamberg, Boaerstig	49,9231	10,9133
BC_ZSM_HETA_0834	**KM023130**	Rhyparochromidae	***Beosus maritimus***	Bavaria	Sandharlander Heide	48,8503	11,7922
BFB_Heteroptera_Schmolke_0523	**KM022098**	Rhyparochromidae	***Beosus maritimus***	Brandenburg	Berlin, Spandau	52,5221	13,1902
BFB_Heteroptera_Kuechler_0021	**KM022711**	Rhyparochromidae	***Drymus brunneus***	Bavaria	Bayreuth, Botanical Garden	49,9230	11,5830
BFB_Heteroptera_Kuechler_0022	**KM021464**	Rhyparochromidae	***Drymus brunneus***	Bavaria	Bayreuth, Botanical Garden	49,9230	11,5830
EUBUG_1120_f_Drymbrun4	**KM021459**	Rhyparochromidae	***Drymus brunneus***	Bavaria	Neuburg an der Donau	48,7390	11,3010
BFB_Heteroptera_Kuechler_0023	**KM022280**	Rhyparochromidae	***Drymus ryeii***	Bavaria	Bayreuth, Botanical Garden	49,9230	11,5830

EUBUG_254_f_Drymsylv3	**KM022311**	Rhyparochromidae	***Drymus sylvaticus***	Brandenburg	Schorfheide-Chorin	53,1070	14,0000
BC_ZSM_HETA_0784	**KM021621**	Rhyparochromidae	***Emblethis verbasci***	Bavaria	Kelheimwinzer	48,9175	11,9178
BFB_Heteroptera_Kuechler_0277	**KM023128**	Rhyparochromidae	***Emblethis verbasci***	Bavaria	Kronach, Rennesberg	50,2570	11,3700
EUBUG_1423_m_Emblverb1	**KM022258**	Rhyparochromidae	***Emblethis verbasci***	Bavaria	Rosenau	48,6620	12,5780
BFB_Heteroptera_Schmolke_0731	**KM022285**	Rhyparochromidae	***Eremocoris abietis***	Bavaria	Pleinfeld, Seemannsmuehle	49,1217	11,0003
BFB_Heteroptera_Kuechler_0085	**KM022541**	Rhyparochromidae	***Eremocoris plebejus***	Bavaria	Bayreuth, Botanical Garden	49,9230	11,5830
BFB_Heteroptera_Kuechler_0100	**KM022267**	Rhyparochromidae	***Eremocoris plebejus***	Bavaria	Bayreuth, Saas	49,9110	11,5520
BFB_Heteroptera_Kuechler_0212	**KM023080**	Rhyparochromidae	***Eremocoris plebejus***	Bavaria	Vorbach	50,1050	10,7190
BFB_Heteroptera_Schmolke_0732	**KM021584**	Rhyparochromidae	***Eremocoris plebejus***	Bavaria	Dassfeld	48,7589	11,8383
BFB_Heteroptera_Schmolke_0734	**KM021747**	Rhyparochromidae	***Eremocoris plebejus***	Bavaria	Eglersried	48,4117	11,4031
BFB_Heteroptera_Schmolke_0735	**KM021781**	Rhyparochromidae	***Eremocoris plebejus***	Bavaria	Uebersee	47,8381	12,4778
EUBUG_1119_m_Erempoda6	**KM022587**	Rhyparochromidae	***Eremocoris plebejus***	Baden-Wuerttemberg	Fronreute	47,8600	9,5510
EUBUG_119_f_Erempleb1	**KM023032**	Rhyparochromidae	***Eremocoris plebejus***	Baden-Wuerttemberg	Altshausen	49,9230	9,5390
BFB_Heteroptera_Kuechler_0063	**KM022228**	Rhyparochromidae	***Eremocoris podagricus***	Bavaria	Bayreuth	49,9260	11,5620
BFB_Heteroptera_Kuechler_0042	**KM022976**	Rhyparochromidae	***Gastrodes abietum***	Bavaria	Bayreuth, Studentenwald	49,9230	11,5730
BFB_Heteroptera_Kuechler_0043	**KM023054**	Rhyparochromidae	***Gastrodes abietum***	Bavaria	Bayreuth, Studentenwald	49,9230	11,5730
BFB_Heteroptera_Schmolke_0544	**KM022011**	Rhyparochromidae	***Gastrodes abietum***	Brandenburg	Berlin, Tegel	52,5874	13,2842
BFB_Heteroptera_Schmolke_0738	**KM021953**	Rhyparochromidae	***Gastrodes abietum***	Bavaria	Grainau	47,4745	11,0303
EUBUG_11_m_Gastabie1	**KM021550**	Rhyparochromidae	***Gastrodes abietum***	Baden-Wuerttemberg	Altshausen	49,9230	9,5390
EUBUG_12_f_Gastabie2	**KM022480**	Rhyparochromidae	***Gastrodes abietum***	Baden-Wuerttemberg	Altshausen	49,9230	9,5390
EUBUG_13_f_Gastabie3	**KM021517**	Rhyparochromidae	***Gastrodes abietum***	Baden-Wuerttemberg	Altshausen	49,9230	9,5390
EUBUG_14_f_Gastabie4	**KM022022**	Rhyparochromidae	***Gastrodes abietum***	Baden-Wuerttemberg	Altshausen	49,9230	9,5390
EUBUG_15_f_Gastabie5	**KM022169**	Rhyparochromidae	***Gastrodes abietum***	Baden-Wuerttemberg	Altshausen	49,9230	9,5390
EUBUG_514_m_Gastabie6	**KM022705**	Rhyparochromidae	***Gastrodes abietum***	Lower Saxony	Wahnbek	53,7940	7,8970
BFB_Heteroptera_Kuechler_0082	**KM022406**	Rhyparochromidae	***Gastrodes grossipes***	Bavaria	Bayreuth, Botanical Garden	49,9230	11,5830
BFB_Heteroptera_Kuechler_0354	**KM021822**	Rhyparochromidae	***Gastrodes grossipes***	Bavaria	Bayreuth, Creussen	49,8490	11,5960
BFB_Heteroptera_Kuechler_0355	**KM021689**	Rhyparochromidae	***Gastrodes grossipes***	Bavaria	Bayreuth, Botanical Garden	49,9230	11,5850
BFB_Heteroptera_Schmolke_0739	**KM021439**	Rhyparochromidae	***Gastrodes grossipes***	Bavaria	Munich, Korbinianihoelzl	48,2269	11,5444
BFB_Heteroptera_Schmolke_0740	**KM022377**	Rhyparochromidae	***Gastrodes grossipes***	Bavaria	Koenigsbrunner Heide	48,2725	10,9081
BFB_Heteroptera_Schmolke_0741	**KM021969**	Rhyparochromidae	***Gastrodes grossipes***	Bavaria	Ruhpolding, Brand	47,7406	12,5942
BFB_Heteroptera_Schmolke_0742	**KM021988**	Rhyparochromidae	***Gastrodes grossipes***	Bavaria	Munich, Froettmaninger Heide	48,2114	11,6083
EUBUG_1118_m_Gastgros1	**KM022843**	Rhyparochromidae	***Gastrodes grossipes***	Baden-Wuerttemberg	Fronreute	47,8600	9,5510
BC_ZSM_HETA_0835	**KM021479**	Rhyparochromidae	***Graptopeltus lynceus***	Bavaria	Pleinfeld, Heinzenmuehle	49,1378	11,0158

BC_ZSM_HETA_0836	**KM022963**	Rhyparochromidae	***Graptopeltus lynceus***	Bavaria	Siegenburg, Dassfeld	48,7589	11,8383
BC_ZSM_HETA_0838	**KM022778**	Rhyparochromidae	***Graptopeltus lynceus***	Bavaria	Nuernberg, Reichelsdorf	49,3861	11,0303
BFB_Heteroptera_Schmolke_0343	**KM022592**	Rhyparochromidae	***Ischnocoris angustulus***	Bavaria	Pollanten	49,1511	11,4433
BFB_Heteroptera_Schmolke_0745	**KM022018**	Rhyparochromidae	***Ischnocoris angustulus***	Bavaria	Pleinfeld, Heinzenmuehle	49,1217	11,0003
BFB_Heteroptera_Kuechler_0293	**KM022531**	Rhyparochromidae	***Ischnocoris hemipterus***	Rhineland Palatinate	Fischbach bei Dahn	49,0910	7,7150
BFB_Heteroptera_Kuechler_0294	**KM022657**	Rhyparochromidae	***Ischnocoris hemipterus***	Rhineland Palatinate	Fischbach bei Dahn	49,0910	7,7150
BFB_Heteroptera_Schmolke_0747	**KM021907**	Rhyparochromidae	***Ischnocoris hemipterus***	Austria	Hundsheimer Berge	48,1267	16,9371
EUBUG_974_m_Ischhemi1	**KM021570**	Rhyparochromidae	***Ischnocoris hemipterus***	Rhineland Palatinate	Fischbach bei Dahn	49,0900	7,7150
BC_ZSM_HETA_0788	**KM022283**	Rhyparochromidae	***Macrodema microptera***	Bavaria	Fuchsmuehle	49,4425	11,2822
BFB_Heteroptera_Schmolke_0344	**KM021850**	Rhyparochromidae	***Macrodema microptera***	Bavaria	Pollanten	49,1511	11,4433
EUBUG_342_m_Macrmicr1	**KM022553**	Rhyparochromidae	***Macrodema microptera***	North Rhine-Westphalia	Haltern, Westruper Heide	51,7360	7,2380
EUBUG_343_m_Macrmicr2	**KM022326**	Rhyparochromidae	***Macrodema microptera***	North Rhine-Westphalia	Haltern, Westruper Heide	51,7360	7,2380
EUBUG_344_m_Macrmicr3	**KM023122**	Rhyparochromidae	***Macrodema microptera***	North Rhine-Westphalia	Haltern, Westruper Heide	51,7360	7,2380
EUBUG_345_f_Macrmicr4	**KM022902**	Rhyparochromidae	***Macrodema microptera***	North Rhine-Westphalia	Haltern, Westruper Heide	51,7360	7,2380
EUBUG_753_f_Macrmicr5	**KM022392**	Rhyparochromidae	***Macrodema microptera***	Rhineland Palatinate	Fischbach bei Dahn	49,1040	7,6830
BFB_Heteroptera_Kuechler_0044	**KM022306**	Rhyparochromidae	***Megalonotus antennatus***	Bavaria	Bayreuth, Creussen	49,8490	11,5960
BFB_Heteroptera_Kuechler_0139	**KM022420**	Rhyparochromidae	***Megalonotus chiragra***	Bavaria	Bayreuth, Hohenmirsberger-Platte	49,8150	11,4450
BFB_Heteroptera_Kuechler_0257	**KM022212**	Rhyparochromidae	***Megalonotus chiragra***	Bavaria	Bayreuth, Botanical Garden	49,9230	11,5850
EUBUG_477_f_Megachir1	**KM022168**	Rhyparochromidae	***Megalonotus chiragra***	Thuringia	Hainich-Duen	51,3020	10,4360
BC_ZSM_HETA_0808	**KM021566**	Rhyparochromidae	***Megalonotus dilatatus***	Bavaria	Pleinfeld, Seemannsmuehle	49,1217	11,0003
BC_ZSM_HETA_0809	**KM022821**	Rhyparochromidae	***Megalonotus dilatatus***	Bavaria	Eining, Sandharlander Heide	48,8503	11,7922
BC_ZSM_HETA_0810	**KM022875**	Rhyparochromidae	***Megalonotus praetextatus***	Bavaria	Offenstetten	48,8111	11,9106
BC_ZSM_HETA_0811	**KM022049**	Rhyparochromidae	***Megalonotus praetextatus***	Bavaria	Pleinfeld, Seemannsmuehle	49,1217	11,0003
BFB_Heteroptera_Kuechler_0258	**KM022820**	Rhyparochromidae	***Megalonotus sabulicola***	Bavaria	Bayreuth, Botanical Garden	49,9230	11,5850
BC_ZSM_HETA_0817	**KM022021**	Rhyparochromidae	***Pachybrachius fracticollis***	Austria	Walchsee in Tirol	47,6538	12,3188
BFB_Heteroptera_Kuechler_0066	**KM022688**	Rhyparochromidae	***Pachybrachius fracticollis***	Bavaria	Bayreuth, Creussen	49,8490	11,5960
BFB_Heteroptera_Kuechler_0067	**KM022801**	Rhyparochromidae	***Pachybrachius fracticollis***	Bavaria	Bayreuth, Creussen	49,8490	11,5960
BC_ZSM_HETA_0842	**KM021612**	Rhyparochromidae	***Panaorus adspersus***	Bavaria	Lenggries, Sylvensteinsee	47,5950	11,5536
BFB_Heteroptera_Kuechler_0045	**KM022407**	Rhyparochromidae	***Peritrechus geniculatus***	Bavaria	Bayreuth, Creussen	49,8490	11,5960
BFB_Heteroptera_Kuechler_0363	**KM021871**	Rhyparochromidae	***Peritrechus geniculatus***	Bavaria	Bayreuth, Schlehenmuehle	49,9050	11,6240
BFB_Heteroptera_Schmolke_0346	**KM021833**	Rhyparochromidae	***Peritrechus geniculatus***	Bavaria	Pollanten	49,1511	11,4433
EUBUG_1393_f_Perigeni6	**KM021469**	Rhyparochromidae	***Peritrechus geniculatus***	Thuringia	Hainich-Duen	51,2750	10,4470
EUBUG_407_f_Perigeni2	**KM021847**	Rhyparochromidae	***Peritrechus geniculatus***	Thuringia	Hainich-Duen	51,0230	10,3220

EUBUG_408_f_Perigeni3	**KM022598**	Rhyparochromidae	***Peritrechus geniculatus***	Thuringia	Hainich-Duen	51,0230	10,3220
EUBUG_752_f_Perigeni4	**KM022397**	Rhyparochromidae	***Peritrechus geniculatus***	Rhineland Palatinate	Fischbach bei Dahn	49,1040	7,6830
EUBUG_752_m_Perigeni5	**KM021588**	Rhyparochromidae	***Peritrechus geniculatus***	Rhineland Palatinate	Fischbach bei Dahn	49,1040	7,6830
BFB_Heteroptera_Kuechler_0081	**KM021763**	Rhyparochromidae	***Peritrechus gracilicornis***	Bavaria	Bayreuth, Creussen	49,8490	11,5960
BFB_Heteroptera_Kuechler_0350	**KM021989**	Rhyparochromidae	***Peritrechus gracilicornis***	Italy	Florence, San Piero a Sieve	43,9610	11,3120
BC_ZSM_HETA_0846	**KM022725**	Rhyparochromidae	***Peritrechus lundii***	Bavaria	Pleinfeld, Heinzenmuehle	49,1378	11,0158
BC_ZSM_HETA_0847	**KM022036**	Rhyparochromidae	***Peritrechus lundii***	Bavaria	Bad Abbach, Oberndorf	48,9481	12,0269
BFB_Heteroptera_Kuechler_0075	**KM022007**	Rhyparochromidae	***Peritrechus lundii***	Bavaria	Bayreuth, Botanical Garden	49,9230	11,5830
BFB_Heteroptera_Kuechler_0349	**KM022524**	Rhyparochromidae	***Peritrechus meridionalis***	Italy	Florence, San Piero a Sieve	43,9610	11,3120
BFB_Heteroptera_Schmolke_0345	**KM022814**	Rhyparochromidae	***Pionosomus varius***	Bavaria	Siegenburg	48,7589	11,8383
BC_ZSM_HETA_0821	**KM022379**	Rhyparochromidae	***Plinthisus pusillus***	Austria	Podersdorf, Seewinkel	47,8615	16,8348
BC_ZSM_HETA_0822	**KM023060**	Rhyparochromidae	***Plinthisus pusillus***	Bavaria	Muehlhausen	48,7822	11,7858
BC_ZSM_HETA_0795	**KM023006**	Rhyparochromidae	***Pterotmetus staphyliniformis***	Bavaria	Muehlhausen	48,7822	11,7858
BFB_Heteroptera_Kuechler_0048	**KM021486**	Rhyparochromidae	***Pterotmetus staphyliniformis***	Bavaria	Bayreuth, Botanical Garden	49,9230	11,5830
EUBUG_752_f_Pterstap1	**KM022780**	Rhyparochromidae	***Pterotmetus staphyliniformis***	Rhineland Palatinate	Fischbach bei Dahn	49,1040	7,6830
BC_ZSM_HETA_0850	**KM022829**	Rhyparochromidae	***Raglius alboacuminatus***	Bavaria	Kallmuenz, Mailerberg	49,1842	11,9450
BFB_Heteroptera_Schmolke_0347	**KM021702**	Rhyparochromidae	***Raglius alboacuminatus***	Bavaria	Kelheimwinzer	48,9175	11,9178
BFB_Heteroptera_Schmolke_0519	**KM023067**	Rhyparochromidae	***Raglius alboacuminatus***	Brandenburg	Berlin, Spandau	52,5221	13,1902
BC_ZSM_HETA_0853	**KM023107**	Rhyparochromidae	***Rhyparochromus phoeniceus***	Bavaria	Munich, Froaettmaninger Heide	48,2114	11,6083
BC_ZSM_HETA_0854	**KM022540**	Rhyparochromidae	***Rhyparochromus phoeniceus***	Bavaria	Lenggries, Isarauen	47,6947	11,5633
BC_ZSM_HETA_0855	**KM021655**	Rhyparochromidae	***Rhyparochromus phoeniceus***	Bavaria	Lenggries, Sylvensteinsee	47,5950	11,5536
BC_ZSM_HETA_0857	**KM023015**	Rhyparochromidae	***Rhyparochromus pini***	Bavaria	Schoengeising	48,1483	11,1958
BC_ZSM_HETA_0858	**KM022301**	Rhyparochromidae	***Rhyparochromus pini***	Bavaria	Muehlhausen	48,7822	11,7858
BC_ZSM_HETA_0859	**KM022557**	Rhyparochromidae	***Rhyparochromus pini***	Bavaria	Munich, Froettmaninger Heide	48,2114	11,6083
BFB_Heteroptera_Kuechler_0031	**KM022408**	Rhyparochromidae	***Rhyparochromus pini***	Bavaria	Bayreuth, Destuben	49,9020	11,5680
BFB_Heteroptera_Kuechler_0278	**KM021445**	Rhyparochromidae	***Rhyparochromus pini***	Bavaria	Kronach, Rennesberg	50,2570	11,3700
BFB_Heteroptera_Schmolke_0541	**KM022795**	Rhyparochromidae	***Rhyparochromus pini***	Brandenburg	Berlin, Tegel	52,5874	13,2842
EUBUG_751_m_Rhyppini2	**KM022951**	Rhyparochromidae	***Rhyparochromus pini***	Rhineland Palatinate	Fischbach bei Dahn	49,1040	7,6830
EUBUG_887_m_Rhyppini3	**KM021659**	Rhyparochromidae	***Rhyparochromus pini***	Thuringia	Jena	50,9510	11,6240
EUBUG_904_f_Rhyppini4	**KM022432**	Rhyparochromidae	***Rhyparochromus pini***	Thuringia	Jena	50,9510	11,6240
EUBUG_920_m_Rhyppini5	**KM021547**	Rhyparochromidae	***Rhyparochromus pini***	Thuringia	Jena	50,9510	11,6240
EUBUG_921_m_Rhyppini6	**KM021601**	Rhyparochromidae	***Rhyparochromus pini***	Thuringia	Jena	50,9510	11,6240
BC_ZSM_HETA_0862	**KM022538**	Rhyparochromidae	***Rhyparochromus vulgaris***	Bavaria	Siegenburg, Dassfeld	48,7589	11,8383

BC_ZSM_HETA_0863	**KM022616**	Rhyparochromidae	***Rhyparochromus vulgaris***	Bavaria	Munich, Allacher Heide	48,2008	11,4828
BFB_Heteroptera_Kuechler_0034	**KM021755**	Rhyparochromidae	***Rhyparochromus vulgaris***	Bavaria	Bayreuth	49,9260	11,5620
BFB_Heteroptera_Kuechler_0062	**KM022123**	Rhyparochromidae	***Rhyparochromus vulgaris***	Bavaria	Bayreuth	49,9260	11,5620
BFB_Heteroptera_Schmolke_0538	**KM022906**	Rhyparochromidae	***Rhyparochromus vulgaris***	Brandenburg	Berlin, Spandau	52,5221	13,1902
EUBUG_906_f_Rhypvulg3	**KM022709**	Rhyparochromidae	***Rhyparochromus vulgaris***	Thuringia	Jena	50,9510	11,6240
EUBUG_924_m_Rhypvulg4	**KM022624**	Rhyparochromidae	***Rhyparochromus vulgaris***	Thuringia	Jena	50,9510	11,6240
EUBUG_925_f_Rhypvulg5	**KM022966**	Rhyparochromidae	***Rhyparochromus vulgaris***	Thuringia	Jena	50,9510	11,6240
EUBUG_926_m_Rhypvulg6	**KM021737**	Rhyparochromidae	***Rhyparochromus vulgaris***	Thuringia	Jena	50,9510	11,6240
BFB_Heteroptera_Schmolke_0539	**KM021897**	Rhyparochromidae	***Scolopostethus affinis***	Brandenburg	Berlin, Tegel	52,5874	13,2842
BFB_Heteroptera_Kuechler_0036	**KM023016**	Rhyparochromidae	***Scolopostethus decoratus***	Bavaria	Bayreuth, Botanical Garden	49,9230	11,5830
BFB_Heteroptera_Kuechler_0040	**KM022948**	Rhyparochromidae	***Scolopostethus decoratus***	Bavaria	Bayreuth, Botanical Garden	49,9230	11,5830
BFB_Heteroptera_Kuechler_0035	**KM021684**	Rhyparochromidae	***Scolopostethus pictus***	Bavaria	Bayreuth, University	49,9290	11,5830
BFB_Heteroptera_Kuechler_0061	**KM021688**	Rhyparochromidae	***Scolopostethus pictus***	Bavaria	Schweinfurt	50,0530	10,2290
EUBUG_158_f_Scolpict1	**KM022660**	Rhyparochromidae	***Scolopostethus pictus***	Thuringia	Hainich-Duen	51,1210	10,5080
EUBUG_511_f_Scolthom3	**KM022700**	Rhyparochromidae	***Scolopostethus pictus***	Lower Saxony	Wahnbek	53,7940	7,8970
BC_ZSM_HETA_0771	**KM022230**	Rhyparochromidae	***Scolopostethus thomsoni***	Bavaria	Munich, Allacher Heide	48,2008	11,4828
BC_ZSM_HETA_0772	**KM022812**	Rhyparochromidae	***Scolopostethus thomsoni***	Bavaria	Munich, Maising	47,9828	11,2883
BFB_Heteroptera_Kuechler_0041	**KM021976**	Rhyparochromidae	***Scolopostethus thomsoni***	Bavaria	Bayreuth, Botanical Garden	49,9230	11,5830
BFB_Heteroptera_Kuechler_0065	**KM022463**	Rhyparochromidae	***Scolopostethus thomsoni***	Bavaria	Bayreuth, Creussen	49,8490	11,5960
BFB_Heteroptera_Kuechler_0079	**KM022529**	Rhyparochromidae	***Scolopostethus thomsoni***	Bavaria	Bayreuth, Creussen	49,8490	11,5960
BFB_Heteroptera_Kuechler_0117	**KM022546**	Rhyparochromidae	***Scolopostethus thomsoni***	Bavaria	Bayreuth, Botanical Garden	49,9230	11,5830
BFB_Heteroptera_Schmolke_0543	**KM021779**	Rhyparochromidae	***Scolopostethus thomsoni***	Brandenburg	Berlin, Tegel	52,5874	13,2842
EUBUG_440_m_Scolthom1	**KM021562**	Rhyparochromidae	***Scolopostethus thomsoni***	Baden-Wuerttemberg	Swabian Alb	48,3760	9,5190
BFB_Heteroptera_Schmolke_0545	**KM022911**	Rhyparochromidae	***Sphragisticus nebulosus***	Brandenburg	Berlin, Tegel	52,5874	13,2842
BC_ZSM_HETA_0872	**KM022294**	Rhyparochromidae	***Stygnocoris cimbricus***	Bavaria	Lenggries, Sylvensteinsee	47,5950	11,5536
BC_ZSM_HETA_0874	**KM021978**	Rhyparochromidae	***Stygnocoris fuligineus***	Bavaria	Nuernberg, Reichelsdorf	49,3861	11,0303
BC_ZSM_HETA_0875	**KM022390**	Rhyparochromidae	***Stygnocoris fuligineus***	Bavaria	Volkach, Astheim	49,8547	10,2086
BC_ZSM_HETA_0876	**KM022497**	Rhyparochromidae	***Stygnocoris fuligineus***	Bavaria	Siegenburg, Dassfeld	48,7589	11,8383
BC_ZSM_HETA_0878	**KM022332**	Rhyparochromidae	***Stygnocoris rusticus***	Bavaria	Munich, Allacher Heide	48,2008	11,4828
BC_ZSM_HETA_0879	**KM021440**	Rhyparochromidae	***Stygnocoris rusticus***	Bavaria	Obereichstaett	48,8978	11,1228
BC_ZSM_HETA_0880	**KM022198**	Rhyparochromidae	***Stygnocoris rusticus***	Bavaria	Urfeld	47,6200	11,3428
BFB_Heteroptera_Kuechler_0361	**KM021739**	Rhyparochromidae	***Stygnocoris rusticus***	Bavaria	Bayreuth, Schlehenmuehle	49,9050	11,6240
EUBUG_1396_f_Stygrust3	**KM021629**	Rhyparochromidae	***Stygnocoris rusticus***	Baden-Wuerttemberg	Swabian Alb	48,3990	9,5180

EUBUG_437_m_Stygrust1	**KM022855**	Rhyparochromidae	***Stygnocoris rusticus***	Baden-Wuerttemberg	Swabian Alb	48,4170	9,3580
BC_ZSM_HETA_0881	**KM022664**	Rhyparochromidae	***Stygnocoris sabulosus***	Bavaria	Obereichstaett	48,8978	11,1228
BC_ZSM_HETA_0882	**KM021873**	Rhyparochromidae	***Stygnocoris sabulosus***	Bavaria	Friedergries	47,4903	10,9508
BC_ZSM_HETA_0883	**KM022282**	Rhyparochromidae	***Stygnocoris sabulosus***	Bavaria	Leinburg, Fuchsmuehle	49,4425	11,2822
BC_ZSM_HETA_0884	**KM022215**	Rhyparochromidae	***Stygnocoris sabulosus***	Bavaria	Kallmuenz, Mailerberg	49,1842	11,9450
BFB_Heteroptera_Kuechler_0223	**KM021487**	Rhyparochromidae	***Stygnocoris sabulosus***	Bavaria	Bayreuth, Botanical Garden	49,9230	11,5850
BFB_Heteroptera_Kuechler_0369	**KM022199**	Rhyparochromidae	***Stygnocoris sabulosus***	Bavaria	Bayreuth, Schlehenmuehle	49,9050	11,6240
EUBUG_445_m_Stygsabu1	**KM022108**	Rhyparochromidae	***Stygnocoris sabulosus***	Baden-Wuerttemberg	Swabian Alb	48,4900	9,3560
BFB_Heteroptera_Schmolke_0524	**KM022789**	Rhyparochromidae	***Trapezonotus arenarius***	Brandenburg	Berlin, Spandau	52,5221	13,1902
EUBUG_1044_f_Traparen1	**KM022661**	Rhyparochromidae	***Trapezonotus arenarius***	Baden-Wuerttemberg	Fronreute	47,8390	9,6060
EUBUG_516_f_Trapdisp1	**KM023081**	Rhyparochromidae	***Trapezonotus dispar***	North Rhine-Westphalia	Oer-Erkenschwick	51,6440	7,2660
EUBUG_517_m_Trapdisp2	**KM022830**	Rhyparochromidae	***Trapezonotus dispar***	North Rhine-Westphalia	Oer-Erkenschwick	51,6440	7,2660
EUBUG_518_f_Trapdisp3	**KM022417**	Rhyparochromidae	***Trapezonotus dispar***	North Rhine-Westphalia	Oer-Erkenschwick	51,6440	7,2660
EUBUG_519_m_Trapdisp4	**KM022969**	Rhyparochromidae	***Trapezonotus dispar***	North Rhine-Westphalia	Oer-Erkenschwick	51,6440	7,2660
EUBUG_520_m_Trapdisp5	**KM022518**	Rhyparochromidae	***Trapezonotus dispar***	North Rhine-Westphalia	Oer-Erkenschwick	51,6440	7,2660
EUBUG_949_f_Tropholo1	**KM022675**	Rhyparochromidae	***Tropistethus holosericus***	Thuringia	Jena	50,9510	11,6240
BC_ZSM_HETA_0864	**KM022008**	Rhyparochromidae	***Xanthochilus quadratus***	Bavaria	Muehlhausen, Schlierferhaide	49,2150	11,4569
BC_ZSM_HETA_0866	**KM022418**	Rhyparochromidae	***Xanthochilus quadratus***	Bavaria	Schwaig, Fuererstein	49,4733	11,2631
BC_ZSM_HETA_0867	**KM023120**	Rhyparochromidae	***Xanthochilus quadratus***	Bavaria	Pleinfeld, Hohenweiler	49,1308	10,9867
BFB_Heteroptera_Schmolke_0518	**KM022354**	Rhyparochromidae	***Xanthochilus quadratus***	Brandenburg	Mallnow Oderhaenge	52,4701	14,4781
BFB_Heteroptera_Kuechler_0006	**KM022977**	Saldidae	***Chartoscirta cincta***	Bavaria	Bayreuth, Mistelbach	49,9310	11,5370
BFB_Heteroptera_Kuechler_0007	**KM022110**	Saldidae	***Chartoscirta cincta***	Bavaria	Bayreuth, Creussen	49,8490	11,5960
EUBUG_1144_f_Charcinc1	**KM022556**	Saldidae	***Chartoscirta cincta***	Brandenburg	Schorfheide-Chorin	53,1230	13,7070
EUBUG_1145_m_Charcinc2	**KM022781**	Saldidae	***Chartoscirta cincta***	Brandenburg	Schorfheide-Chorin	53,1230	13,7070
BFB_Heteroptera_Kuechler_0080	**KM022337**	Saldidae	***Chartoscirta elegantula***	Bavaria	Bayreuth, Hoerhof	49,8710	11,6100
BFB_Heteroptera_Schmolke_016	**KM021639**	Saldidae	***Macrosaldula scotica***	Bavaria	Naring	47,7128	11,8358
BFB_Heteroptera_Schmolke_018	**KM022810**	Saldidae	***Saldula arenicola***	Bavaria	Spalt	49,1731	10,9847
BFB_Heteroptera_Schmolke_019	**KM022563**	Saldidae	***Saldula c-album***	Bavaria	Ruhpolding	47,7394	12,6028
BFB_Heteroptera_Schmolke_017	**KM022569**	Saldidae	***Saldula melanoscela***	Bavaria	Weichs	48,4117	11,4031
BFB_Heteroptera_Kuechler_0146	**KM023075**	Saldidae	***Saldula orthochila***	Bavaria	Muenchberg	50,1810	11,8140
BFB_Heteroptera_Schmolke_020	**KM023077**	Saldidae	***Saldula pallipes***	Bavaria	Munich	48,2114	11,6083
BFB_Heteroptera_Schmolke_021	**KM022663**	Saldidae	***Saldula pallipes***	Bavaria	Roth	49,1669	11,1783
BFB_Heteroptera_Schmolke_022	**KM023002**	Saldidae	***Saldula pallipes***	Bavaria	Maising	47,9828	11,2883

BFB_Heteroptera_Schmolke_023	**KM022773**	Saldidae	***Saldula pallipes***	Bavaria	Munich	48,1906	11,5269
BFB_Heteroptera_Schmolke_024	**KM022747**	Saldidae	***Saldula saltatoria***	Bavaria	Offenstetten	48,8111	11,9106
BFB_Heteroptera_Schmolke_025	**KM021955**	Saldidae	***Saldula saltatoria***	Bavaria	Spalt	49,1731	10,9847
EUBUG_1195_m_Saldsalt5	**KM021886**	Saldidae	***Saldula saltatoria***	Bavaria	Bavarian Forest, Riedelhuette	48,9210	13,4450
EUBUG_1196_m_Saldsalt6	**KM022798**	Saldidae	***Saldula saltatoria***	Bavaria	Bavarian Forest, Riedelhuette	48,9210	13,4450
EUBUG_1198_f_Saldsalt7	**KM022393**	Saldidae	***Saldula saltatoria***	Bavaria	Bavarian Forest, Rainer Wald	48,9110	12,4510
EUBUG_378_m_Saldsalt1	**KM021775**	Saldidae	***Saldula saltatoria***	Bavaria	Neuburg an der Donau	48,7370	11,2930
BFB_Heteroptera_Kuechler_0289	**KM022119**	Scutelleridae	***Eurygaster austriaca***	Bavaria	Ingolstadt, Gerolfinger Eichenwald	48,7560	11,3260
BFB_Heteroptera_Kuechler_0028	**KM022659**	Scutelleridae	***Eurygaster maura***	Bavaria	Bayreuth, Botanical Garden	49,9230	11,5830
BFB_Heteroptera_Schmolke_0359	**KM021534**	Scutelleridae	***Eurygaster maura***	Bavaria	Pollanten	49,1511	11,4433
EUBUG_196_m_Eurymaur1	**KM022073**	Scutelleridae	***Eurygaster maura***	Thuringia	Hainich-Duen	51,0340	10,4590
EUBUG_226_f_Eurymaur2	**KM022776**	Scutelleridae	***Eurygaster maura***	Thuringia	Hainich-Duen	51,2140	10,3870
EUBUG_227_f_Eurymaur3	**KM022584**	Scutelleridae	***Eurygaster maura***	Thuringia	Hainich-Duen	51,2140	10,3870
BFB_Heteroptera_Kuechler_0027	**KM022602**	Scutelleridae	***Eurygaster testudinaria***	Bavaria	Bayreuth, Botanical Garden	49,9230	11,5830
EUBUG_1223_m_Eurytest10	**KM021610**	Scutelleridae	***Eurygaster testudinaria***	Bavaria	Bavarian Forest, Rainer Wald	48,9150	12,4550
EUBUG_438_f_Eurytest2	**KM022329**	Scutelleridae	***Eurygaster testudinaria***	Baden-Wuerttemberg	Swabian Alb	48,3960	9,4390
EUBUG_439_m_Eurytest3	**KM021619**	Scutelleridae	***Eurygaster testudinaria***	Baden-Wuerttemberg	Swabian Alb	48,3960	9,4390
EUBUG_558_f_Eurytest4	**KM022883**	Scutelleridae	***Eurygaster testudinaria***	Baden-Wuerttemberg	Pfrunger Ried	47,8990	9,3900
BFB_Heteroptera_Schmolke_0360	**KM022336**	Scutelleridae	***Odontotarsus purpureolineatus***	Austria	Oggau	47,8754	16,7782
BFB_Heteroptera_Kuechler_0142	**KM022762**	Stenocephalidae	***Dicranocephalus medius***	Bavaria	Bayreuth, Botanical Garden	49,9230	11,5850
EUBUG_114_juv_Acalmusc2	**KM021468**	Tingidae	***Acalypta carinata***	Baden-Wuerttemberg	Altshausen	49,9230	9,5390
EUBUG_115_juv_Acalmusc3	**KM022442**	Tingidae	***Acalypta carinata***	Baden-Wuerttemberg	Altshausen	49,9230	9,5390
EUBUG_149_f_Acalplat5	**KM022261**	Tingidae	***Acalypta carinata***	Bavaria	Neuburg an der Donau	48,7460	11,2710
BFB_Heteroptera_Schmolke_028	**KM021958**	Tingidae	***Acalypta gracilis***	Bavaria	Siegenburg	48,7589	11,8383
BFB_Heteroptera_Schmolke_031	**KM021706**	Tingidae	***Acalypta marginata***	Bavaria	Langwied	48,1872	11,4011
BFB_Heteroptera_Schmolke_032	**KM021729**	Tingidae	***Acalypta marginata***	Bavaria	Siegenburg	48,7589	11,8383
EUBUG_950_m_Acalnigr4	**KM022740**	Tingidae	***Acalypta marginata***	Thuringia	Jena	50,9510	11,6240
EUBUG_951_f_Acalnigr5	**KM022314**	Tingidae	***Acalypta marginata***	Thuringia	Jena	50,9510	11,6240
EUBUG_113_m_Acalmusc1	**KM021542**	Tingidae	***Acalypta musci***	Baden-Wuerttemberg	Altshausen	49,9230	9,5390
EUBUG_602_f_Acalmusc4	**KM021708**	Tingidae	***Acalypta musci***	Rhineland Palatinate	Fischbach bei Dahn	49,0860	7,7230
EUBUG_603_f_Acalmusc5	**KM022934**	Tingidae	***Acalypta musci***	Rhineland Palatinate	Fischbach bei Dahn	49,0860	7,7230
EUBUG_982_juv_Acalsp1	**KM022095**	Tingidae	***Acalypta musci***	Baden-Wuerttemberg	Altshausen	49,9230	9,5390
BFB_Heteroptera_Schmolke_033	**KM022491**	Tingidae	***Acalypta nigrina***	Bavaria	Siegenburg	48,7589	11,8383

EUBUG_584_m_Acanigr1	**KM021475**	Tingidae	***Acalypta nigrina***	Baden-Wuerttemberg	Pfrunger Ried	47,8990	9,3900
EUBUG_585_f_Acanigr2	**KM021806**	Tingidae	***Acalypta nigrina***	Baden-Wuerttemberg	Pfrunger Ried	47,8990	9,3900
EUBUG_586_f_Acanigr3	**KM022650**	Tingidae	***Acalypta nigrina***	Baden-Wuerttemberg	Pfrunger Ried	47,8990	9,3900
BFB_Heteroptera_Kuechler_0037	**KM022310**	Tingidae	***Acalypta parvula***	Bavaria	Bayreuth, Botanical Garden	49,9230	11,5830
EUBUG_604_f_Agraconf1	**KM022345**	Tingidae	***Agramma confusum***	Rhineland Palatinate	Fischbach bei Dahn	49,0860	7,7230
BFB_Heteroptera_Schmolke_034	**KM021593**	Tingidae	***Agramma ruficorne***	Bavaria	Weyarn	47,8594	11,8139
BFB_Heteroptera_Schmolke_035	**KM021466**	Tingidae	***Agramma ruficorne***	Bavaria	Ascholding	47,8958	11,5050
EUBUG_767_m_Catofabr1	**KM022370**	Tingidae	***Catoplatus fabricii***	Baden-Wuerttemberg	Swabian Alb	47,8990	9,3900
EUBUG_894_m_Catofabr2	**KM022749**	Tingidae	***Catoplatus fabricii***	Thuringia	Jena	50,9510	11,6240
EUBUG_895_m_Catofabr3	**KM021888**	Tingidae	***Catoplatus fabricii***	Thuringia	Jena	50,9510	11,6240
EUBUG_896_f_Catofabr4	**KM022643**	Tingidae	***Catoplatus fabricii***	Thuringia	Jena	50,9510	11,6240
BFB_Heteroptera_Schmolke_036	**KM022488**	Tingidae	***Catoplatus nigriceps***	Austria	Hainburg	48,1340	16,9332
BFB_Heteroptera_Schmolke_037	**KM021604**	Tingidae	***Copium clavicorne***	Bavaria	Laaber	48,7783	12,0200
BFB_Heteroptera_Schmolke_038	**KM022644**	Tingidae	***Copium clavicorne***	Bavaria	Kallmuenz	49,1842	11,9450
BFB_Heteroptera_Schmolke_039	**KM022237**	Tingidae	***Copium clavicorne***	Bavaria	Pfuenz	48,8872	11,2711
BFB_Heteroptera_Schmolke_040	**KM021502**	Tingidae	***Copium clavicorne***	Bavaria	Obereichstaett	48,8978	11,1228
BFB_Heteroptera_Kuechler_0340	**KM022742**	Tingidae	***Corythucha ciliata***	France	La Garde-Freinet	43,3300	6,4700
BFB_Heteroptera_Kuechler_0341	**KM022819**	Tingidae	***Corythucha ciliata***	France	La Garde-Freinet	43,3300	6,4700
EUBUG_759_f_Corycili1	**KM021816**	Tingidae	***Corythucha ciliata***	Bavaria	Munich	48,1130	11,3880
EUBUG_760_m_Corycili2	**KM022100**	Tingidae	***Corythucha ciliata***	Bavaria	Munich	48,1130	11,3880
BFB_Heteroptera_Kuechler_0241	**KM021811**	Tingidae	***Derephysia foliacea***	Bavaria	Muenchberg	50,1810	11,8140
BFB_Heteroptera_Kuechler_0242	**KM021693**	Tingidae	***Derephysia foliacea***	Bavaria	Muenchberg	50,1810	11,8140
EUBUG_503_m_Derefoli1	**KM021556**	Tingidae	***Derephysia foliacea***	Bavaria	Bavarian Forest, Solla	48,8200	13,3050
BFB_Heteroptera_Kuechler_0261	**KM023027**	Tingidae	***Dictyla echii***	Bavaria	Bayreuth	49,9260	11,5620
BFB_Heteroptera_Schmolke_042	**KM021677**	Tingidae	***Dictyla echii***	Bavaria	Erlangen	49,5861	11,0308
BFB_Heteroptera_Schmolke_043	**KM022104**	Tingidae	***Dictyla echii***	Bavaria	Volkach	49,8547	10,2086
BFB_Heteroptera_Schmolke_044	**KM021644**	Tingidae	***Dictyla echii***	Bavaria	Munich	48,2114	11,6083
BFB_Heteroptera_Schmolke_045	**KM021834**	Tingidae	***Dictyla echii***	Bavaria	Munich	48,1914	11,4847
EUBUG_1416_f_Dictechi1	**KM021853**	Tingidae	***Dictyla echii***	Bavaria	Rosenau	48,6610	12,5800
BFB_Heteroptera_Schmolke_046	**KM021783**	Tingidae	***Dictyla humuli***	Bavaria	Munich	48,1906	11,5269
BFB_Heteroptera_Schmolke_047	**KM021540**	Tingidae	***Dictyla humuli***	Bavaria	Kallmuenz	49,1367	11,9481
BFB_Heteroptera_Schmolke_048	**KM022400**	Tingidae	***Dictyla humuli***	Bavaria	Elsendorf	48,7164	11,7981
BFB_Heteroptera_Schmolke_049	**KM021931**	Tingidae	***Dictyla humuli***	Bavaria	Munich	48,2008	11,4828

EUBUG_464_f_Dicthumu2	**KM021443**	Tingidae	***Dictyla humuli***	Brandenburg	Schorfheide-Chorin	53,1160	13,9990
EUBUG_465_f_Dicthumu3	**KM022980**	Tingidae	***Dictyla humuli***	Brandenburg	Schorfheide-Chorin	53,1160	13,9990
BFB_Heteroptera_Schmolke_052	**KM021591**	Tingidae	***Dictyonota strichnocera***	Bavaria	Roth	49,1669	11,1783
BFB_Heteroptera_Schmolke_053	**KM022317**	Tingidae	***Dictyonota strichnocera***	Bavaria	Elsendorf	48,7164	11,7981
BFB_Heteroptera_Schmolke_054	**KM022190**	Tingidae	***Dictyonota strichnocera***	Bavaria	Pleinfeld	49,1217	11,0003
BFB_Heteroptera_Schmolke_055	**KM022735**	Tingidae	***Dictyonota strichnocera***	Bavaria	Erlangen	49,5861	11,0308
BFB_Heteroptera_Schmolke_056	**KM022861**	Tingidae	***Galeatus affinis***	Bavaria	Siegenburg	48,7589	11,8383
BFB_Heteroptera_Kuechler_0314	**KM021714**	Tingidae	***Kalama tricornis***	Rhineland Palatinate	Fischbach bei Dahn	49,1290	7,6900
BFB_Heteroptera_Schmolke_0532	**KM023069**	Tingidae	***Kalama tricornis***	Brandenburg	Berlin, Spandau	52,5221	13,1902
BFB_Heteroptera_Schmolke_057	**KM021567**	Tingidae	***Kalama tricornis***	Bavaria	Erlangen	49,5861	11,0308
BFB_Heteroptera_Schmolke_058	**KM021516**	Tingidae	***Kalama tricornis***	Bavaria	Nuernberg	49,3861	11,0303
BFB_Heteroptera_Schmolke_059	**KM021438**	Tingidae	***Kalama tricornis***	Bavaria	Munich	48,2324	11,4707
BFB_Heteroptera_Schmolke_060	**KM021952**	Tingidae	***Kalama tricornis***	Bavaria	Koenigsbrunn	48,2744	10,9033
BFB_Heteroptera_Schmolke_061	**KM022348**	Tingidae	***Kalama tricornis***	Bavaria	Munich	48,1906	11,5269
EUBUG_297_m_Kalatric1	**KM023082**	Tingidae	***Kalama tricornis***	Thuringia	Hainich-Duen	51,2840	10,4250
EUBUG_754_f_Kalatric4	**KM021803**	Tingidae	***Kalama tricornis***	Rhineland Palatinate	Fischbach bei Dahn	49,1040	7,6830
EUBUG_755_f_Kalatric5	**KM022093**	Tingidae	***Kalama tricornis***	Rhineland Palatinate	Fischbach bei Dahn	49,1040	7,6830
BFB_Heteroptera_Schmolke_062	**KM022907**	Tingidae	***Lasiacantha capucina***	Bavaria	Munich	48,2114	11,6083
BFB_Heteroptera_Schmolke_063	**KM021820**	Tingidae	***Lasiacantha capucina***	Bavaria	Kallmuenz	49,1842	11,9450
BFB_Heteroptera_Schmolke_064	**KM022321**	Tingidae	***Lasiacantha capucina***	Bavaria	Obereichstaett	48,8978	11,1228
BFB_Heteroptera_Schmolke_065	**KM022990**	Tingidae	***Lasiacantha capucina***	Bavaria	Munich	48,2008	11,4828
EUBUG_935_f_Lasicapu1	**KM021950**	Tingidae	***Lasiacantha capucina***	Baden-Wuerttemberg	Swabian Alb	48,3950	9,2600
EUBUG_Seq7_f_Lasicapu	**KM022649**	Tingidae	***Lasiacantha capucina***	Baden-Wuerttemberg	Swabian Alb	48,3954	9,2596
BFB_Heteroptera_Schmolke_066	**KM022674**	Tingidae	***Lasiacantha hermani***	Bavaria	Koenigsbrunn	48,2725	10,9081
BFB_Heteroptera_Kuechler_0131	**KM021765**	Tingidae	***Oncochila simplex***	Bavaria	Bayreuth, Hohenmirsberger-Platte	49,8150	11,4450
BFB_Heteroptera_Kuechler_0132	**KM022020**	Tingidae	***Oncochila simplex***	Bavaria	Bayreuth, Hohenmirsberger-Platte	49,8150	11,4450
BFB_Heteroptera_Schmolke_067	**KM022794**	Tingidae	***Oncochila simplex***	Bavaria	Kallmuenz	49,1842	11,9450
BFB_Heteroptera_Schmolke_068	**KM022908**	Tingidae	***Oncochila simplex***	Bavaria	Siegenburg	48,7589	11,8383
BFB_Heteroptera_Schmolke_069	**KM022118**	Tingidae	***Oncochila simplex***	Bavaria	Nuernberg	49,3861	11,0303
BFB_Heteroptera_Schmolke_070	**KM023103**	Tingidae	***Oncochila simplex***	Bavaria	Munich	48,2269	11,5444
EUBUG_1142_f_Oncosimp1	**KM021496**	Tingidae	***Oncochila simplex***	Baden-Wuerttemberg	Swabian Alb	48,4620	9,4920
BFB_Heteroptera_Kuechler_0094	**KM022537**	Tingidae	***Physatocheila dumetorum***	Bavaria	Bayreuth	49,9260	11,5620
BFB_Heteroptera_Kuechler_0095	**KM022723**	Tingidae	***Physatocheila dumetorum***	Bavaria	Bayreuth	49,9260	11,5620

BFB_Heteroptera_Schmolke_074	**KM022065**	Tingidae	***Tingis ampliata***	Bavaria	Ascholding	47,8958	11,5050
BFB_Heteroptera_Schmolke_075	**KM023044**	Tingidae	***Tingis ampliata***	Bavaria	Offenstetten	48,8111	11,9106
EUBUG_Seq24_f_Tingampl	**KM022939**	Tingidae	***Tingis ampliata***	Brandenburg	Schorfheide-Chorin	53,1074	14,0004
BFB_Heteroptera_Kuechler_0130	**KM022284**	Tingidae	***Tingis cardui***	Bavaria	Bayreuth, Hohenmirsberger-Platte	49,8150	11,4450
BFB_Heteroptera_Schmolke_076	**KM021757**	Tingidae	***Tingis cardui***	Bavaria	Pleinfeld	49,1267	10,9778
BFB_Heteroptera_Schmolke_077	**KM021883**	Tingidae	***Tingis cardui***	Bavaria	Siegenburg	48,7589	11,8383
BFB_Heteroptera_Schmolke_078	**KM022577**	Tingidae	***Tingis cardui***	Bavaria	Munich	48,1906	11,5269
BFB_Heteroptera_Schmolke_079	**KM021559**	Tingidae	***Tingis cardui***	Bavaria	Erlangen	49,5861	11,0308
EUBUG_409_m_Tingcard4	**KM022078**	Tingidae	***Tingis cardui***	Thuringia	Hainich-Duen	51,2640	10,4980
BFB_Heteroptera_Kuechler_0115	**KM022236**	Tingidae	***Tingis crispata***	Bavaria	Bayreuth, University	49,9290	11,5830
BFB_Heteroptera_Kuechler_0116	**KM022955**	Tingidae	***Tingis crispata***	Bavaria	Bayreuth, University	49,9290	11,5830
BFB_Heteroptera_Schmolke_082	**KM021851**	Tingidae	***Tingis crispata***	Bavaria	Munich	48,2114	11,6083
BFB_Heteroptera_Schmolke_072	**KM022562**	Tingidae	***Tingis pilosa***	Bavaria	Weichs	48,4117	11,4031
BFB_Heteroptera_Schmolke_073	**KM022322**	Tingidae	***Tingis pilosa***	Bavaria	Munich	48,1914	11,4847
BFB_Heteroptera_Schmolke_084	**KM022850**	Tingidae	***Tingis reticulata***	Austria	Hinterriss	47,4672	11,4839
BFB_Heteroptera_Schmolke_085	**KM022205**	Tingidae	***Tingis reticulata***	Bavaria	Kallmuenz	49,1842	11,9450
BFB_Heteroptera_Schmolke_086	**KM022273**	Tingidae	***Tingis reticulata***	Bavaria	Kelheimwinzer	48,9175	11,9178
BC ZSM AQU 00020	**HM401270**	Veliidae	***Microvelia reticulata***	Bavaria	Traunstein	48,0000	12,8400
BC ZSM AQU 00208	**HM421992**	Veliidae	***Microvelia reticulata***	Bavaria	Ansbach	49,0320	10,3690
BFB_Heteroptera_Schmolke_005	**KM022860**	Veliidae	***Microvelia reticulata***	Bavaria	Munich	48,1906	11,5269
GBOL00364	**KM021652**	Veliidae	***Microvelia reticulata***	Brandenburg	Zerwelinsee	53,3001	13,6214
BC ZSM AQU 00715	**HQ563141**	Veliidae	***Velia caprai***	Bavaria	Eichstaett	49,0250	11,2460
BCZSMAQU001109	**KM021510**	Veliidae	***Velia caprai***	Bavaria	Starnberg	48,0300	11,3600
EUBUG_525_f_Velicapr1	**KM022656**	Veliidae	***Velia caprai***	Baden-Wuerttemberg	Pfrunger Ried	47,8990	9,3900
EUBUG_526_f_Velicapr2	**KM021480**	Veliidae	***Velia caprai***	Baden-Wuerttemberg	Pfrunger Ried	47,8990	9,3900
GBOL00362	**KM021742**	Veliidae	***Velia caprai***	Brandenburg	Zerwelinsee	53,3001	13,6214
BFB_Heteroptera_Schmolke_006	**KM023088**	Veliidae	***Velia saulii***	Bavaria	Eining	48,8503	11,7922
