# Supplementary material for: Building-Up of a DNA Barcode Library for True Bugs (Insecta: Hemiptera: Heteroptera) of Germany Reveals Taxonomic Uncertainties and Surprises
Source: PLoS One. 2014 Sep 9;9(9):e106940. doi: 10.1371/journal.pone.0106940 (PMC4159288; doi:10.1371/journal.pone.0106940)
Supplement: Appendix S3 — Supplementary information about the used PERL-Script APE and modified ABGD pipeline. Species of the Heteroptera with at least two corresponding BINs (see Table 3) are marked in bold. (DOCX) [file pone.0106940.s003.docx]

**ABGD Parameter Explorer (APE) Version 1.0**

**July 2014**

APE.pl has been written for remote controlling and analysis of results produced by the Automatic Barcode Gap Discovery program. It starts ABGD with the following arguments:

-n100 -a -d0 -v >>logfile.txt

This is done 100 times while the additional argument -X (relative gap width) increases from 0.1 to 10 in 0.1 steps. The output consists of image-plots that are computed by R. The file results/image.eps contains an image-plot of the whole run, showing how number of estimated groups differ, when using different parameters. Furthermore, files that are named in the pattern 'Alignmentname'_x='value'.eps show if species are recognized as single (red) or at least two (blue) groups. The files that are named in the pattern 'Alignmentname'_x='value'_merged.eps discriminate the cases i) species were put in one group with other species (blue) or ii) were not put together (red).

For examples see the files image.eps, Miridae_MG_MJR_x=0.1 and Miridae_MG_MJR_x=0.1_merged. All files are part of the script package.

***Comments***

This script has been only been tested on Linux ("Linux 3.7.20-2.36-desktop openSUSE 12.3(Dartmouth) (x86_64)") until now. APE will not run on Windows due to differences in directory path syntax. Please notice that APE will only work if all requirements have been fulfilled as specified under the topic "Installation" in this README file.

***Installation***

This script needs the ABGD command-line version installed on your system. Make sure you downloaded the C-sources from http://wwwabi.snv.jussieu.fr/public/abgd/last.tgz and build it in a local directory following the installation guide of ABGD.

- Copy the files "APE.pl" and "BANANA.pl" into the directory, where you built ABGD.
- Make sure the following Perl-modules are installed on your system:

"Statistics::R"

"List::Util"

"Math::BigFloat"

- To visualize the data, you need R installed on your system. It has been successfully tested with R version 3.1.0 (2014-04-10) -- "Spring Dance".

***ARGUMENTS***

- Add the Argument "-h" to see this README text.
- Try also “–monkey”.

***Starting the run***

- Copy the Alignment file into the ABGD-directory. Alignment-files need to be of type *.fasta. Sequences have to be named, following the pattern of the sample below.

>EUBUG056-11_Acanthosoma_haemorrhoidale

AACAATATATTTTATATTCGGAATATGAGCAGGAATAGTAGGAACCGCTATAAGATGAAT

TATTCGCATTGAATTAAGTCAACCTAGATCTTTTATTGGTGATGATCAAATTTATAATGT

AGTAGTAACTGCACATGCATTTATTATAATCTTTTTTATGGTAATACCTGTAATAATTGG

AGGTTTTGGAAATTGATTAGTGCCATTAATAATTGGAGCACCCGATATAGCATTTCCTCG

AATAAATAATATAAGATTTTGACTATTACCCCCTTCATTAACACTATTATTAATTAGAAG

ATTAGCAGAATCGGGAGTAGGTACTGGTTGAACTGTTTACCCTCCCTTATCAAGAAATTT

AGCCCACAGAGGAGCATCCGTAGACTTAGCTATCTTTTCATTACATTTAGCGGGTGTATC

TTCAATTCTAGGAGCTGTAAATTTTATTTCTACTATTATTAATATACGCCCTATGGGTAT

AATACCAGAACGTATCCCTTTATTCGTATGATCAGTAGGAATTACTGCCTTATTATTATT

ATTATCTTTACCTGTATTAGCAGGAGCTATTACTATACTACTAACAGATCGAAACTTTAA

TACATCTTTCTTTGACCCATCTGGGGGAGGAGACCCTATTCTATATCAACACTTATTT

- To start the script, navigate in your console to the directory where you installed ABGD APE.pl and BANANA.pl, type "perl APE.pl" and follow the instructions. For large datasets, APE will be relatively slow. Do not worry when you don't see changes in your console for a longer time …

***Troubleshooting***

APE will only run in the presence of all used components. Please follow installation instructions as mentioned above. If you receive error message relating to R, please check if your version is compatible with the used modules and try to get help from the R online help.

When using this script, please notice that you have to cite the original paper describing the Automatic Barcode Gap Discovery:

Puillandre N, Lambert A, Brouillet S, Achaz G (2012): ABGD, Automatic Barcode Gap Discovery for primary species delimitation. Molecular Ecology 21: 1864–1877.

For further information about this script please contact:

[fabian.deister@uni-oldenburg.de](mailto:fabian.deister@uni-oldenburg.de) or [mraupach@senckenberg.de](mailto:mraupach@senckenberg.de)
