# Supplementary material for: Building-Up of a DNA Barcode Library for True Bugs (Insecta: Hemiptera: Heteroptera) of Germany Reveals Taxonomic Uncertainties and Surprises
Source: PLoS One. 2014 Sep 9;9(9):e106940. doi: 10.1371/journal.pone.0106940 (PMC4159288; doi:10.1371/journal.pone.0106940)
Supplement: Appendix S6 — Barcode distances of the analyzed Heteroptera. Divergence values were calculated for all sequences >400 base pairs, using the Nearest Neighbour Summary as part of the Barcode Gap Analysis tool provided in the Barcode of Life Data System (BOLD). Used distance model: Kimura 2-paramter, align sequencing option: BOLD aligner (amino acid based HMM), ambiguous base/gap handling: pairwise deletion. ISD = intra-specific distance. (DOCX) [file pone.0106940.s006.docx]

| Species | BIN | *n* | Mean  ISD | Max  ISD | Nearest species (NS) | Distance  to NS |
| --- | --- | --- | --- | --- | --- | --- |
| Nepidae |  |  |  |  |  |  |
| *Nepa cinerea* Linnaeus, 1758 | AAK8359 | 1 | - | - | *Ranatra linearis* | 19.38 |
| *Ranatra linearis* (Linnaeus, 1758) | AAL1328 | 2 | 0.62 | 0.62 | *Nepa cinerea* | 19.38 |
| Corixidae |  |  |  |  |  |  |
| *Arctocorisa carinata* (C.R. Sahlberg, 1819) | AAJ7903 | 1 | - | - | *Sigara semistriata* | 8.25 |
| *Callicorixa praeusta* (Fieber, 1848) | AAK1938 | 1 | - | - | *Arctocorisa carinata* | 10.86 |
| *Corixa punctata* (Illiger, 1807) | ACB1799 | 1 | - | - | *Arctocorisa carinata* | 14.83 |
| *Cymatia coleoptrata* (Fabricius, 1777) | ACB1796 | 2 | 0 | 0 | *Cymatia rogenhoferi* | 12.71 |
| *Cymatia rogenhoferi* (Fieber, 1864) | ACB2132 | 1 | - | - | *Cymatia coleoptrata* | 12.71 |
| *Glaenocorisa propinqua* (Fieber 1861) | ABX4248 | 5 | 0.97 | 1.55 | *Sigara semistriata* | 9.88 |
| *Hesperocorixa linnaei* (Fieber, 1848) | ABX0448 | 1 | - | - | *Sigara venusta* | 11.98 |
| *Hesperocorixa sahlbergi* (Fieber, 1848) | AAN0795 | 4 | 1.03 | 1.52 | *Sigara fossarum* | 12.23 |
| *Micronecta griseola* Horváth, 1899 | AAK6480 | 2 | 0 | 0 | *Micronecta poweri* | 10.63 |
| *Micronecta poweri* (Douglas & Scott, 1869) | ACB1970 | 1 | - | - | *Micronecta griseola* | 10.63 |
| *Micronecta scholtzi* (Fieber, 1860) | AAK6479 | 1 | - | - | *Sigara venusta* | 18.85 |
| *Paracorixa concinna* (Fieber, 1848) | ABV3365 | 1 | - | - | *Sigara venusta* | 7.07 |
| *Sigara falleni* (Fieber, 1848) | AAH9524, ABY7152 | 4 | 1.45 | 2.24 | *Sigara fossarum* | 8 |
| *Sigara fossarum* (Leach, 1817) | AAJ6707 | 2 | 0.5 | 0.5 | *Sigara falleni* | 8 |
| *Sigara lateralis* (Leach, 1817) | AAJ6697 | 1 | - | - | *Sigara striata* | 10.04 |
| *Sigara nigrolineata* (Fieber, 1848) | ACB1978 | 2 | 0.15 | 0.15 | *Sigara venusta* | 10.55 |
| *Sigara semistriata* (Fieber, 1848) | ACB1949 | 1 | - | - | *Paracorixa concinna* | 8.11 |
| *Sigara striata* (Linnaeus, 1758) | AAJ6688 | 1 | - | - | *Sigara venusta* | 8.98 |
| *Sigara venusta* (Douglas & Scott, 1869) | ABA5309 | 2 | 0 | 0 | *Paracorixa concinna* | 7.07 |
| Naucoridae |  |  |  |  |  |  |
| *Ilyocoris cimicoides* (Linnaeus, 1758) | AAF2590 | 2 | 0.02 | 0.02 | *-* | - |
| Notonectidae |  |  |  |  |  |  |
| *Notonecta glauca* Linnaeus, 1758 | AAK4442 | 4 | 0.15 | 0.35 | *Notonecta lutea* | 5.4 |
| *Notonecta lutea* Müller, 1776 | AAN1701 | 9 | 0.17 | 0.33 | *Notonecta reuteri* | 1.24 |
| *Notonecta maculata* Fabricius, 1794 | AAN1703 | 4 | 0.58 | 0.85 | *Notonecta glauca* | 7.11 |
| *Notonecta reuteri* Hungerford, 1928 | ACE8526 | 4 | 0.08 | 0.16 | *Notonecta lutea* | 1.24 |
| *Notonecta viridis* Delcourt, 1909 | ABV0133 | 2 | 0.46 | 0.48 | *Notonecta glauca* | 5.41 |
| Pleidae |  |  |  |  |  |  |
| *Plea minutissima* Leach, 1817 | AAF3832 | 1 | - | - | *-* | - |
| Mesoveliidae |  |  |  |  |  |  |
| *Mesovelia furcata* Mulsant & Rey, 1852 | AAN2451 | 2 | 0.03 | 0.03 | *-* | - |
| Hebridae |  |  |  |  |  |  |
| *Hebrus pusillus* (Fallén, 1807) | AAN0981 | 2 | 0.15 | 0.15 | *Hebrus ruficeps* | 14.32 |
| *Hebrus ruficeps* Thomson, 1871 | AAI6967 | 2 | 0.15 | 0.15 | *Hebrus pusillus* | 14.32 |
| Hydrometridae |  |  |  |  |  |  |
| *Hydrometra gracilenta* Horváth, 1899 | AAN0857 | 5 | 0.18 | 0.31 | *Hydrometra stagnorum* | 13.61 |
| *Hydrometra stagnorum* (Linnaeus, 1758) | AAK5632 | 5 | 0.05 | 0.16 | *Hydrometra gracilenta* | 13.61 |
| Veliidae |  |  |  |  |  |  |
| *Microvelia reticulata* (Burmeister, 1835) | AAG4340, AAG4341 | 4 | 2.15 | 3.98 | *Velia saulii* | 17.91 |
| *Velia caprai* Tamanini, 1947 | AAN0455 | 5 | 0 | 0 | *Velia saulii* | 5.11 |
| *Velia saulii* Tamanini, 1947 | ABX0836 | 1 | - | - | *Velia caprai* | 5.11 |
| Gerridae |  |  |  |  |  |  |
| *Aquarius najas* (De Geer, 1773) | AAN1521 | 1 | - | - | *Gerris thoracicus* | 11.75 |
| *Aquarius paludum* (Fabricius, 1794) | AAI7450 | 2 | 0.33 | 0.33 | *Gerris asper* | 13.55 |
| *Gerris argentatus* Schummel, 1832 | - | 1 | - | - | *Gerris odontogaster* | 6.78 |
| *Gerris asper* (Fieber, 1860) | ABA3327 | 1 | - | - | *Gerris gibbifer* | 11.25 |
| *Gerris costae* (Herrich-Schaeffer, 1850) | ACI6181 | 1 | - | - | *Gerris thoracicus* | 7.72 |
| *Gerris gibbifer* Schummel, 1832 | ACB1756 | 1 | - | - | *Gerris costae* | 9.55 |
| *Gerris lacustris* (Linnaeus, 1758) | - | 6 | 0.08 | 0.25 | *Gerris gibbifer* | 9.6 |
| *Gerris odontogaster* (Zetterstedt, 1828) | ABU6679 | 3 | 1.01 | 1.53 | *Gerris argentatus* | 6.76 |
| *Gerris thoracicus* Schummel, 1832 | ACB1745 | 3 | 0 | 0 | *Gerris costae* | 7.72 |
| *Limnoporus rufoscutellatus* (Latreille, 1807) | ACJ5120 | 2 | 0.32 | 0.32 | *Gerris asper* | 11.86 |
| Saldidae |  |  |  |  |  |  |
| *Chartoscirta cincta* (Herrich-Schaeffer, 1841) | AAY9046 | 4 | 0 | 0 | *Chartoscirta elegantula* | 5.94 |
| *Chartoscirta elegantula* (Fallén, 1807) | AAY9439 | 1 | - | - | *Chartoscirta cincta* | 5.94 |
| *Macrosaldula scotica* (Curtis, 1835) | ABX0415 | 1 | - | - | *Chartoscirta cincta* | 13.36 |
| *Saldula arenicola* (Scholtz, 1847) | - | 1 | - | - | *Saldula melanoscela* | 10.5 |
| *Saldula c-album* (Fieber, 1859) | ABY0743 | 1 | - | - | *Saldula saltatoria* | 4.86 |
| *Saldula melanoscela* (Fieber, 1859) | ABX1081 | 1 | - | - | *Saldula saltatoria* | 5.38 |
| *Saldula orthochila* (Fieber, 1859) | AAY9314 | 1 | - | - | *Macrosaldula scotica* | 16.27 |
| *Saldula pallipes* (Fabricius, 1794) | AAI1011 | 4 | 0.28 | 0.76 | *Saldula saltatoria* | 10.59 |
| *Saldula saltatoria* (Linnaeus, 1758) | ABA2372 | 6 | 0.06 | 0.25 | *Saldula c-album* | 4.86 |
| Tingidae |  |  |  |  |  |  |
| *Acalypta carinata* (Panzer, 1806) | - | 3 | 0.34 | 0.5 | *Acalypta nigrina* | 13.19 |
| *Acalypta gracilis* (Fieber, 1844) | ABX1070 | 1 | - | - | *Tingis reticulata* | 16.74 |
| *Acalypta marginata* (Wolff, 1804) | ABU8673 | 4 | 0.51 | 0.76 | *Acalypta nigrina* | 12.04 |
| *Acalypta musci* (Schrank, 1781) | AAZ9767 | 4 | 0.2 | 0.31 | *Derephysia foliacea* | 18.91 |
| *Acalypta nigrina* (Fallén, 1807) | ABA3298 | 4 | 0.38 | 0.76 | *Acalypta marginata* | 12.04 |
| *Acalypta parvula* (Fallén, 1807) | ABY3100 | 1 | - | - | *Dictyonota strichnocera* | 18.51 |
| *Agramma confusum* (Puton, 1879) | ABU5791 | 1 | - | - | *Tingis reticulata* | 17.54 |
| *Agramma ruficorne* (Germar, 1835) | ABX1045 | 2 | 0.15 | 0.15 | *Derephysia foliacea* | 17.04 |
| *Catoplatus fabricii* (Stål, 1868) | ABU9007 | 4 | 0 | 0 | *Tingis pilosa* | 16.22 |
| *Catoplatus nigriceps* Horváth, 1905 | - | 1 | - | - | *Dictyla humuli* | 16.64 |
| *Copium clavicorne* (Linnaeus, 1758) | ABX0835 | 4 | 0 | 0 | *Derephysia foliacea* | 15.58 |
| *Corythucha ciliata* (Say, 1832) | ABU8597 | 4 | 0.08 | 0.15 | *Tingis cardui* | 17.31 |
| *Derephysia foliacea* (Fallén, 1807) | ABA3368 | 3 | 0 | 0 | *Copium claviocorne* | 15.58 |
| *Dictyla echii* (Schrank, 1782) | AAZ1208 | 6 | 0.35 | 0.76 | *Dictyla humuli* | 15.96 |
| *Dictyla humuli* (Fabricius, 1794) | ABV4788 | 6 | 0.36 | 0.75 | *Tingis reticulata* | 15.52 |
| *Dictyonota strichnocera* Fieber, 1844 | ABX0837 | 4 | 0.15 | 0.31 | *Copium claviocorne* | 16.99 |
| *Galeatus affinis* (Herrich-Schaeffer, 1835) | ABX1078 | 1 | - | - | *Agramma ruficorne* | 18 |
| *Kalama tricornis* (Schrank, 1801) | AAZ1069 | 10 | 0.06 | 0.31 | *Copium claviocorne* | 16.6 |
| *Lasiacantha capucina* (Germar, 1837) | ABV5634 | 6 | 0.23 | 0.54 | *Lasiacantha hermani* | 14.23 |
| *Lasiacantha hermani* Vásárhelyi, 1977 | ABX1063 | 1 | - | - | *Lasiacantha capucina* | 14.23 |
| *Oncochila simplex* (Herrich-Schaeffer, 1830) | AAY8965 | 7 | 0.12 | 0.31 | *Tingis reticulata* | 17.88 |
| *Physatocheila dumetorum* (Herrich-Schaeffer, 1838) | AAY8941 | 2 | 0 | 0 | *Tingis reticulata* | 15.12 |
| *Tingis ampliata* (Herrich-Schaeffer, 1838) | ABX1072 | 3 | 1.07 | 1.57 | *Tingis cardui* | 15.47 |
| *Tingis cardui* (Linnaeus, 1758) | AAY9344 | 6 | 0.64 | 1.87 | *Tingis ampliata* | 15.47 |
| *Tingis crispata* (Herrich-Schaeffer, 1838) | AAY8986 | 3 | 0.63 | 0.94 | *Tingis ampliata* | 16.84 |
| *Tingis pilosa* Hummel, 1825 | ABX1009 | 2 | 0 | 0 | *Catoplatus fabricii* | 16.22 |
| *Tingis reticulata* Herrich-Schaeffer, 1835 | ABX0843 | 3 | 0 | 0 | *Lasiacantha hermani* | 14.96 |
| Microphysidae |  |  |  |  |  |  |
| *Loricula elegantula* (Baerensprung, 1858) | ABU6426 | 1 | - | - | *-* | - |
| Miridae |  |  |  |  |  |  |
| *Acetropis carinata* (Herrich-Schaeffer, 1841) | ACD1473 | 2 | 0 | 0 | *Leptopterna ferrugata* | 13.51 |
| *Adelphocoris detritus* (Fieber, 1861) | ACF4925 | 1 | - | - | *Adelphocoris lineolatus* | 1.55 |
| *Adelphocoris lineolatus* (Goeze, 1778) | ACE7444, ACF1257 | 11 | 0.81 | 2.05 | *Adelphocoris detritus* | 1.55 |
| *Adelphocoris quadripunctatus* (Fabricius, 1794) | ABY7543 | 7 | 0.04 | 0.25 | *Adelphocoris reichelii* | 0.31 |
| *Adelphocoris reichelii* (Fieber, 1836) | ABY7543 | 2 | 0.15 | 0.15 | *Adelphocoris quadripunctatus* | 0.31 |
| *Adelphocoris seticornis* (Fabricius, 1775) | ABA6871 | 9 | 0.14 | 0.33 | *Adelphocoris lineolatus* | 3.84 |
| *Agnocoris reclairei* (Wagner, 1949) | AAZ9002 | 2 | 0 | 0 | *Agnocoris rubicundus* | 0.15 |
| *Agnocoris rubicundus* (Fallén, 1807) | AAZ9002 | 4 | 0.18 | 0.31 | *Agnocoris reclairei* | 0.15 |
| *Alloeotomus germanicus* Wagner, 1939 | - | 1 | - | - | *Pantilius tunicatus* | 17.84 |
| *Amblytylus nasutus* (Kirschbaum, 1856) | AAJ2399 | 8 | 0 | 0 | *Lopus decolor* | 15.9 |
| *Apolygus limbatus* (Fallén, 1807) | ABA3034 | 5 | 0 | 0 | *Apolygus lucorum* | 4.71 |
| *Apolygus lucorum* (Meyer-Dür, 1843) | - | 1 | - | - | *Apolygus rhamnicola* | 1.78 |
| *Apolygus rhamnicola* (Reuter, 1885) | ABW9275 | 1 | - | - | *Apolygus lucorum* | 1.78 |
| *Atractotomus magnicornis* (Fallén, 1807) | ABV9583 | 8 | 2.33 | 5.46 | *Phoenicocoris obscurellus* | 14.19 |
| *Atractotomus mali* (Meyer-Dür, 1843) | ABA4245 | 2 | 0 | 0 | *Psallus ambiguus* | 14.52 |
| *Blepharidopterus angulatus* (Fallén, 1807) | ABV8133 | 5 | 0.76 | 1.78 | *Orthotylus flavinervis* | 16.6 |
| *Bothynotus pilosus* (Boheman, 1852) | ABV8112 | 5 | 0 | 0 | *Phytocoris longipennis* | 19.07 |
| *Brachynotocoris puncticornis* Reuter, 1880 | - | 1 | - | - | *Orthotylus fuscescens* | 15.93 |
| *Bryocoris pteridis* (Fallén, 1807) | ABA2846 | 6 | 0.1 | 0.31 | *Lygus gemellatus* | 17.32 |
| *Calocoris affinis* (Herrich-Schaeffer, 1835) | ABA4055 | 3 | 0 | 0 | *Dichrooscytus intermedius* | 14.14 |
| *Calocoris alpestris* (Meyer-Dür, 1843) | AAY9456 | 1 | - | - | *Horistus orientalis* | 12.45 |
| *Calocoris roseomaculatus* (De Geer, 1773) | ABV5001 | 1 | - | - | *Adelphocoris quadripunctatus* | 12.89 |
| *Campyloneura virgula* (Herrich-Schaeffer, 1835) | - | 1 | - | - | *Phytocoris dimidiatus* | 15.24 |
| *Capsodes gothicus* (Linnaeus, 1758) | AAY8882 | 4 | 0.1 | 0.15 | *Phytocoris austriacus* | 13.29 |
| *Capsus wagneri* (Remane, 1950) | ABV3513 | 2 | 0 | 0 | *Pantilius tunicatus* | 14.11 |
| *Charagochilus gyllenhalii* (Fallén, 1807) | AAY9446 | 3 | 0 | 0 | *Charagochilus weberi* | 0 |
| *Charagochilus spiralifer* Kerzhner, 1988 | ABA2800 | 10 | 0.52 | 1.39 | *Charagochilus gyllenhalii* | 12.76 |
| *Charagochilus weberi* Wagner, 1953 | AAY9446 | 6 | 0.29 | 0.62 | *Charagochilus gyllenhalii* | 0 |
| *Chlamydatus evanescens* (Boheman, 1852) | AAY9369 | 2 | 0.83 | 0.83 | *Lygus gemellatus* | 13.96 |
| *Chlamydatus pulicarius* (Fallén, 1807) | AAY9054 | 7 | 0.46 | 0.76 | *Monosynamma bohemanni* | 13.25 |
| *Chlamydatus pullus* (Reuter, 1870) | ACD1511 | 6 | 0 | 0 | *Psallus montanus* | 13.48 |
| *Chlamydatus saltitans* (Fallén, 1807) | AAY9055 | 2 | 0 | 0 | *Psallus montanus* | 15.13 |
| *Closterotomus biclavatus* (Herrich-Schaeffer, 1835) | ABV4261 | 6 | 0.77 | 1.52 | *Lygus wagneri* | 13.56 |
| *Closterotomus fulvomaculatus* (De Geer, 1773) | AAY8889 | 4 | 1.2 | 1.89 | *Lygus gemellatus* | 11.97 |
| *Closterotomus norwegicus* (Gmelin, 1790) | AAG8966 | 4 | 0.08 | 0.15 | *Agnocoris rubicundus* | 13.23 |
| *Compsidolon salicellum* (Herrich-Schaeffer, 1841) | AAF0800 | 3 | 0.34 | 0.5 | *Psallus montanus* | 15.72 |
| *Cremnocephalus albolineatus* Reuter, 1875 | ABW9224 | 1 | - | - | *Cremnocephalus alpestris* | 14.48 |
| *Cremnocephalus alpestris* Wagner, 1941 | ABW9054 | 6 | 0 | 0 | *Cremnocephalus albolineatus* | 14.48 |
| *Criocoris crassicornis* (Hahn, 1834) | ABA2820 | 6 | 0.6 | 0.16 | *Psallus montanus* | 13.96 |
| *Cyllecoris histrionius* (Linnaeus, 1767) | ABX4823 | 1 | - | - | *Orthotylus fuscescens* | 18.21 |
| *Deraeocoris annulipes* (Herrich-Schaeffer, 1842) | ACD1179 | 1 | - | - | *Lygus rugulipennis* | 15.06 |
| *Deraeocoris flavilinea* (A. Costa, 1862) | ABA3933 | 6 | 0.49 | 0.93 | *Deraeocoris morio* | 14.81 |
| *Deraeocoris lutescens* (Schilling, 1837) | ABA3934 | 6 | 0.05 | 0.15 | *Adelphocoris quadripunctatus* | 15.04 |
| *Deraeocoris morio* (Boheman, 1852) | ABX3775 | 1 | - | - | *Deraeocoris flavilinea* | 14.81 |
| *Deraeocoris olivaceus* (Fabricius, 1777) | ABU6571 | 4 | 0 | 0 | *Deraeocoris trifasciatus* | 8.56 |
| *Deraeocoris ruber* (Linnaeus, 1758) | AAZ0136 | 9 | 0.4 | 1.02 | *Adelphocoris seticornis* | 16.98 |
| *Deraeocoris trifasciatus* (Linnaeus, 1767) | ABU6570 | 1 | - | - | *Deraeocoris olivaceus* | 8.56 |
| *Dichrooscytus gustavi* Josifov, 1981 | ABU6550 | 1 | - | - | *Dichrooscytus intermedius* | 11.87 |
| *Dichrooscytus intermedius* Reuter, 1885 | ABV9499 | 3 | 0.1 | 0.15 | *Dichrooscytus gustavi* | 11.87 |
| *Dicyphus annulatus* (Wolff, 1804) | ABV9494 | 4 | 0 | 0 | *Phytocoris populi* | 17.31 |
| *Dicyphus epilobii* Reuter, 1883 | ABV9495 | 2 | 0 | 0 | *Dicyphus errans* | 5.26 |
| *Dicyphus errans* (Wolff, 1804) | AAY8990 | 9 | 0.04 | 0.2 | *Dicyphus epilobii* | 5.26 |
| *Dicyphus globulifer* (Fallén, 1829) | ABA3082 | 8 | 0.33 | 1.33 | *Phytocoris austriacus* | 17.61 |
| *Dicyphus hyalinipennis* (Burmeister, 1835) | ABW9205 | 1 | - | - | *Dicyphus epilobii* | 8.17 |
| *Dicyphus pallidus* (Herrich-Schaeffer, 1836) | ABU6527 | 5 | 0.11 | 0.3 | *Dicyphus errans* | 14.38 |
| *Dryophilocoris flavoquadrimaculatus* (De Geer, 1773) | - | 1 | - | - | *Orthotylus viridinervis* | 17.61 |
| *Europiella alpina* (Reuter, 1875) | ABA7205 | 2 | 0.15 | 0.15 | *Europiella artemisiae* | 11.3 |
| *Europiella artemisiae* (Becker, 1864) | ABW7177 | 2 | 0.16 | 0.16 | *Europiella alpina* | 11.3 |
| *Globiceps flavomaculatus* (Fabricius, 1794) | ABU6740 | 3 | 0.1 | 0.15 | *Globiceps fulvicollis* | 1.27 |
| *Globiceps fulvicollis* Jakovlev, 1877 | ABU6740 | 9 | 1.39 | 2.31 | *Globiceps flavomaculatus* | 1.27 |
| *Hadrodemus m-flavum* (Goeze, 1778) | AAY9517 | 3 | 0 | 0 | *Miris striatus* | 12.09 |
| *Halticus apterus* (Linnaeus, 1758) | ABU8961 | 5 | 0.25 | 0.61 | *Halticus luteicollis* | 13.41 |
| *Halticus luteicollis* (Panzer, 1804) | ABV2865 | 3 | 0.05 | 0.15 | *Halticus apterus* | 13.41 |
| *Harpocera thoracica* (Fallén, 1807) | ABU6305 | 6 | 0.15 | 0.25 | *Psallus haematodes* | 13.83 |
| *Heterocordylus erythropthalmus* (Hahn, 1833) | - | 3 | 0 | 0 | *Orthotylus flavinervis* | 18.54 |
| *Heterocordylus genistae* (Scopoli, 1763) | ABW9553 | 1 | - | - | *Heterocordylus tibialis* | 15.85 |
| *Heterocordylus tibialis* (Hahn, 1833) | - | 1 | - | - | *Heterocordylus genistae* | 15.85 |
| *Heterocordylus thunbergii* (Fallén, 1807) | AAZ3173 | 8 | 0.19 | 0.77 | *Phoenicocoris obscurellus* | 16.44 |
| *Horistus orientalis* (Gmelin, 1790) | AAZ3172 | 4 | 0 | 0 | *Calocoris alpestris* | 12.45 |
| *Leptopterna dolobrata* (Linnaeus, 1758) | AAB5081 | 1 | - | - | *Leptopterna ferrugata* | 13.97 |
| *Leptopterna ferrugata* (Fallén, 1807) | AAZ2810 | 4 | 0 | 0 | *Acetropis carinata* | 13.51 |
| *Liocoris tripustulatus* (Fabricius, 1781) | AAY9524 | 6 | 0 | 0 | *Lygus wagneri* | 12.01 |
| *Lopus decolor* (Fallén, 1807) | AAV0220 | 6 | 0 | 0 | *Psallus montanus* | 15.21 |
| *Lygocoris pabulinus* (Linnaeus, 1761) | AAB2217 | 4 | 0 | 0 | *Pinalitus rubricatus* | 13.09 |
| *Lygus gemellatus* (Herrich-Schaeffer, 1835) | AAY8966 | 2 | 0.25 | 0.25 | *Lygus pratensis* | 0 |
| *Lygus pratensis* (Linnaeus, 1758) | AAY8966 | 7 | 0.17 | 0.5 | *Lygus gemellatus* | 0 |
| *Lygus rugulipennis* Poppius, 1911 | ACF4388 | 8 | 0 | 0 | *Lygus wagneri* | 5.43 |
| *Lygus wagneri* Remane, 1955 | AAY8966 | 1 | - | - | *Lygus gemellatus* | 0 |
| *Macrolophus pygmaeus* (Rambur, 1839) | ABX2934 | 1 | - | - | *Macrolophus rubi* | 8.04 |
| *Macrolophus rubi* Woodroffe, 1957 | ABU7198 | 1 | - | - | *Macrolophus pygmaeus* | 8.04 |
| *Macrotylus herrichi* (Reuter, 1873) | ABX0416 | 1 | - | - | *Macrotylus quadrilineatus* | 15.57 |
| *Macrotylus paykullii* (Fallén, 1807) | - | 3 | 4.34 | 6.05 | *Phoenicocoris obscurellus* | 17.27 |
| *Macrotylus quadrilineatus* (Schrank, 1785) | - | 1 | - | - | *Macrotylus herrichi* | 15.57 |
| *Malacocoris chlorizans* (Panzer, 1794) | - | 4 | 0.68 | 1.27 | *Phytocoris varipes* | 18.22 |
| *Megalocoleus molliculus* (Fallén, 1807) -1 | ABV5715 | 7 | 0.43 | 1.53 | *Lygus rugulipennis* | 17.8 |
| *Miris striatus* (Linnaeus, 1758) | AAY8958 | 3 | 0.1 | 0.15 | *Hadrodemus m-flavum* | 12.09 |
| *Monalocoris filicis* (Linnaeus, 1758) | ABU7165 | 7 | 0.58 | 1.86 | *Pantilius tunicatus* | 15.56 |
| *Monosynamma bohemanni* (Fallén, 1829) | ABX7428 | 1 | - | - | *Chlamydatus pulicarius* | 13.25 |
| *Myrmecoris gracilis* (R.F. Sahlberg, 1848) | ABX3811 | 1 | - | - | *Closterotomus fulvomaculatus* | 15.44 |
| *Neolygus contaminatus* (Fallén, 1807) | AAY9465 | 4 | 0.2 | 0.31 | *Neolygus viridis* | 3.6 |
| *Neolygus viridis* (Fallén, 1807) | AAZ0135 | 2 | 0.65 | 0.65 | *Neolygus contaminatus* | 3.6 |
| *Notostira elongata* (Geoffroy, 1785) | AAZ2266 | 7 | 0.4 | 0.77 | *Notostira erratica* | 4.49 |
| *Notostira erratica* (Linnaeus, 1758) | ABU8957 | 6 | 0.13 | 0.25 | *Notostira elongata* | 4.49 |
| *Omphalonotus quadriguttatus* (Kirschbaum, 1856) | - | 1 | - | - | *Systellonotus triguttatus* | 18.48 |
| *Oncotylus punctipes* Reuter, 1875 | ABX7453 | 3 | 0.1 | 0.15 | *Lygus gemellatus* | 13.37 |
| *Orthocephalus brevis* (Panzer, 1798) | ABW9514 | 2 | 1.3 | 1.3 | *Stenotus binotatus* | 14.72 |
| *Orthocephalus coriaceus* (Fabricius, 1777) | AAE1615 | 9 | 0.04 | 0.25 | *Orthocephalus saltator* | 14.66 |
| *Orthocephalus saltator* (Hahn, 1835) | AAI2327 | 4 | 0.37 | 0.77 | *Orthocephalus coriaceus* | 14.66 |
| *Orthonotus rufifrons* (Fallén, 1807) | ABU9790 | 2 | 0 | 0 | *Psallus varians* | 16.46 |
| *Orthops basalis* (A. Costa, 1853) | AAY9496 | 6 | 0.05 | 0.19 | *Orthops kalmii* | 4.09 |
| *Orthops campestris* (Linnaeus, 1758) | AAZ9999 | 2 | 0 | 0 | *Orthops basalis* | 7.52 |
| *Orthops kalmii* (Linnaeus, 1758) | ABA3164 | 4 | 0.14 | 0.25 | *Orthops basalis* | 4.09 |
| *Orthotylus concolor* (Kirschbaum, 1856) | - | 1 | - | - | *Orthotylus flavinervis* | 14.9 |
| *Orthotylus ericetorum* (Fallén, 1807) | ABA2513 | 11 | 0.11 | 0.61 | *Orthotylus interpositus* | 15.31 |
| *Orthotylus flavinervis* (Kirschbaum, 1856) | ABW9510 | 2 | 0 | 0 | *Orthotylus marginalis* | 8.53 |
| *Orthotylus fuscescens* (Kirschbaum, 1856) | - | 1 | - | - | *Brachynotocoris puncticornis* | 15.93 |
| *Orthotylus interpositus* K. Schmidt, 1938 | ABA3049 | 7 | 0.14 | 0.45 | *Orthotylus marginalis* | 8.91 |
| *Orthotylus marginalis* Reuter, 1883 | - | 10 | 0.19 | 0.5 | *Orthotylus flavinervis* | 8.53 |
| *Orthotylus obscurus* Reuter, 1875 | ABU8514 | 5 | 0.12 | 0.31 | *Adelphocoris lineolatus* | 14.16 |
| *Orthotylus prasinus* (Fallén, 1826) | - | 6 | 0 | 0 | *Orthotylus interpositus* | 16.6 |
| *Orthotylus schoberiae* Reuter, 1876 | ABA8098 | 1 | - | - | *Orthotylus flavinervis* | 15.43 |
| *Orthotylus tenellus* (Fallén, 1807) | ABW9511 | 4 | 0.51 | 1.01 | *Globiceps fulvicollis* | 16.31 |
| *Orthotylus virescens* (Douglas & Scott, 1865) | - | 2 | 0 | 0 | *Brachynotocoris puncticornis* | 19.27 |
| *Orthotylus viridinervis* (Kirschbaum, 1856) | ABU6904 | 3 | 0.84 | 1.26 | *Orthotylus flavinervis* | 17.52 |
| *Pachytomella parallela* (Meyer-Dür, 1843) | - | 1 | - | - | *Phoenicococoris modestus* | 20.45 |
| *Pantilius tunicatus* (Fabricius, 1781) | ABU6934 | 4 | 0.1 | 0.15 | *Lygus rugulipennis* | 12.45 |
| *Parapsallus vitellinus* (Scholtz, 1847) | AAF1995 | 4 | 0.13 | 0.28 | *Psallus variabilis* | 17.09 |
| *Phoenicocoris modestus* (Meyer-Dür, 1843) | AAY8940 | 9 | 0.03 | 0.16 | *Phoenicococoris obscurellus* | 13.97 |
| *Phoenicocoris obscurellus* (Fallén, 1829) | AAY8899 | 4 | 0.66 | 1.13 | *Phylus melanocephalus* | 12.14 |
| *Phylus coryli* (Linnaeus, 1758) | AAF5346 | 4 | 0 | 0 | *Phylus melanocephalus* | 11.33 |
| *Phylus melanocephalus* (Linnaeus, 1767) | ABV5420 | 5 | 0 | 0 | *Phylus coryli* | 11.33 |
| *Phylus plagiatus* (Herrich-Schaeffer, 1835) | ABX7405 | 3 | 0 | 0 | *Phylus coryli* | 13.41 |
| *Phytocoris austriacus* Wagner, 1954 | AAH9369 | 1 | - | - | *Phytocoris varipes* | 0.5 |
| *Phytocoris dimidiatus* Kirschbaum, 1856 | ABV5430, ABV8607 | 2 | 10.96 | 10.96 | *Phytocoris populi* | 8.21 |
| *Phytocoris longipennis* Flor, 1861 | ABU7068 | 8 | 0.15 | 0.51 | *Phytocoris tiliae* | 10.24 |
| *Phytocoris pini* Kirschbaum, 1856 | AAF5821 | 2 | 0.93 | 0.93 | *Phytocoris tiliae* | 1.4 |
| *Phytocoris populi* (Linnaeus, 1758) | ABV8127 | 6 | 0 | 0 | *Phytocoris dimidiatus* | 8.21 |
| *Phytocoris tiliae* (Fabricius, 1777) | AAF5821 | 5 | 1.74 | 3.24 | *Phytocoris pini* | 1.4 |
| *Phytocoris ulmi* (Linnaeus, 1758) | AAF3498 | 2 | 0 | 0 | *Phytocoris longipennis* | 11.9 |
| *Phytocoris varipes* Boheman, 1852 | AAH9369 | 2 | 1.53 | 1.53 | *Phytocoris austriacus* | 0.5 |
| *Pilophorus cinnamopterus* (Kirschbaum, 1856) | ABV8592 | 4 | 0.4 | 0.82 | *Pilophorus simulans* | 13.13 |
| *Pilophorus clavatus* (Linnaeus, 1767) | ABA3473, ABA3474 | 5 | 4.21 | 7.15 | *Pilophorus simulans* | 6.45 |
| *Pilophorus confusus* (Kirschbaum, 1856) | ABW8802 | 2 | 0.62 | 0.62 | *Pilophorus clavatus* | 7.64 |
| *Pilophorus perplexus* Douglas & Scott, 1875 | AAF7893 | 8 | 0.33 | 0.62 | *Pilophorus confusus* | 8.34 |
| *Pilophorus simulans* Josifov, 1989 | ABW9673 | 1 | - | - | *Pilophorus clavatus* | 6.45 |
| *Pinalitus atomarius* (Meyer-Dür, 1843) | ABU7140 | 3 | 0 | 0 | *Pinalitus rubricatus* | 9.98 |
| *Pinalitus rubricatus* (Fallén, 1807) | AAZ7215 | 2 | 0.16 | 0.16 | *Pinalitus atomarius* | 9.98 |
| *Pinalitus viscicola* (Puton, 1888) | ABV5404 | 1 | - | - | *Agnocoris reclairei* | 12.27 |
| *Pithanus maerkelii* (Herrich-Schaeffer, 1838) | AAG8967 | 4 | 0.35 | 1 | *Phytocoris varipes* | 13.49 |
| *Plagiognathus arbustorum* (Fabricius, 1794) | - | 16 | 0.16 | 0.5 | *Plagiognathus chrysanthemi* | 11.69 |
| *Plagiognathus chrysanthemi* (Wolff, 1804) | AAE3943 | 11 | 0.44 | 1.01 | *Plagiognathus fulvipennis* | 11.56 |
| *Plagiognathus fulvipennis* (Kirschbaum, 1856) | ABW9664 | 1 | - | - | *Plagiognathus chrysanthemi* | 11.56 |
| *Plesiodema pinetella* (Zetterstedt, 1828) | AAY8946, ABU8515 | 4 | 3.09 | 5.46 | *Oncotylus punctipes* | 18.11 |
| *Polymerus asperulae* (Fieber, 1861) | - | 1 | - | - | *Polymerus microphthalmus* | 5.81 |
| *Polymerus microphthalmus* (Wagner, 1951) | ABU7011 | 2 | 0 | 0 | *Polymerus asperulae* | 5.81 |
| *Polymerus nigrita* (Fallén, 1807) | ABU7009 | 9 | 0 | 0 | *Polymerus unifasciatus* | 12.03 |
| *Polymerus palustris* (Reuter, 1907) | - | 1 | - | - | *Polymerus asperulae* | 8.93 |
| *Polymerus unifasciatus* (Fabricius, 1794) | AAY9312, AAZ3255 | 7 | 1.27 | 3.97 | *Polymerus microphthalmus* | 6.74 |
| *Psallus albicinctus* (Kirschbaum, 1856) | - | 1 | - | - | *Psallus mollis* | 14.14 |
| *Psallus ambiguus* (Fallén, 1807) | AAY8936 | 8 | 0.84 | 3.19 | *Atractotomus mali* | 14.52 |
| *Psallus flavellus* Stichel, 1933 | ABV4449 | 1 | - | - | *Psallus lepidus* | 5.79 |
| *Psallus haematodes* (Gmelin, 1790) | ABA2745 | 5 | 0.16 | 0.46 | *Psallus lepidus* | 8.21 |
| *Psallus lepidus* Fieber, 1858 | ABV0872 | 1 | - | - | *Psallus flavellus* | 5.79 |
| *Psallus mollis* (Mulsant & Rey, 1852) | ABV0871 | 2 | 0 | 0 | *Psallus montanus* | 12.26 |
| *Psallus montanus* Josifov, 1973 | AAZ2092 | 1 | - | - | *Psallus variabilis* | 11.54 |
| *Psallus variabilis* (Fallén, 1807) | AAZ0137 | 7 | 0.11 | 0.25 | *Psallus montanus* | 11.54 |
| *Psallus varians* (Herrich-Schaeffer, 1841) | AAY8935 | 14 | 0.11 | 0.25 | *Psallus montanus* | 11.64 |
| *Pseudoloxops coccineus* (Meyer-Dürr, 1843) | ABW9445 | 2 | 0 | 0 | *Globiceps fluvicollis* | 17.33 |
| *Rhabdomiris striatellus* (Fabricius, 1794) | ABU9372 | 4 | 0.38 | 0.46 | *Adelphocoris quadripunctatus* | 10.7 |
| *Salicarus roseri* (Herrich-Schaeffer, 1838) | ABX7418 | 2 | 0.65 | 0.65 | *Stenodema sericans* | 14.38 |
| *Stenodema calcarata* (Fallén, 1807) | AAY9091, ACI8060, AAZ3133 | 12 | 3.03 | 8.63 | *Stenodema holsata* | 12.66 |
| *Stenodema holsata* (Fabricius, 1787) | AAY9090 | 8 | 0.11 | 0.31 | *Stenodema laevigata* | 8.95 |
| *Stenodema laevigata* (Linnaeus, 1758) | AAY9089 | 9 | 0.52 | 2.27 | *Stenodema holsata* | 8.95 |
| *Stenodema sericans* (Fieber, 1861) | ABW9220 | 1 | - | - | *Stenodema laevigata* | 9.51 |
| *Stenotus binotatus* (Fabricius, 1794) | AAC0635 | 7 | 0.49 | 1.52 | *Agnocoris reclairei* | 10.78 |
| *Strongylocoris leucocephalus* (Linnaeus, 1758) | ACD1310 | 5 | 0.45 | 0.76 | *Strongylocoris steganoides* | 0 |
| *Strongylocoris steganoides* (J. Sahlberg, 1875) | ACD1310 | 4 | 0.59 | 1.01 | *Strongylocoris leucocephalus* | 0 |
| *Systellonotus triguttatus* (Linnaeus, 1767) | ACD1514 | 1 | - | - | *Omphalonotus quadriguttatus* | 18.48 |
| *Teratocoris paludum* J. Sahlberg, 1870 | AAG8677 | 2 | 0 | 0 | *Hadrodemus m-flavum* | 17.22 |
| *Trigonotylus caelestialium* (Kirkaldy, 1902) | AAF9949 | 10 | 0.3 | 0.76 | *Trigonotylus pulchellus* | 0 |
| *Trigonotylus pulchellus* (Hahn, 1834) | AAF9949 | 2 | 0 | 0 | *Trigonotylus caelestialium* | 0 |
| *Tytthus pygmaeus* (Zetterstedt, 1838) | AAZ2721 | 3 | 0 | 0 | *Lygus rugulipennis* | 14.27 |
| Nabidae |  |  |  |  |  |  |
| *Himacerus apterus* (Fabricius, 1798) | AAZ2988 | 7 | 0.14 | 0.51 | *Himacerus mirmicoides* | 11.42 |
| *Himacerus major* (A. Costa, 1842) | ABX7362 | 1 | - | - | *Himacerus apterus* | 13.57 |
| *Himacerus mirmicoides* (O. Costa, 1834) | AAY9075 | 12 | 0.1 | 0.32 | *Himacerus apterus* | 11.42 |
| *Nabis brevis* Scholtz, 1847 | AAZ3346 | 11 | 0.13 | 0.5 | *Nabis rugosus* | 0 |
| *Nabis ericetorum* Scholtz, 1847 | AAZ3346 | 5 | 0.41 | 0.67 | *Nabis brevis* | 0 |
| *Nabis ferus* (Linnaeus, 1758) | ABU9496 | 5 | 1.45 | 3.16 | *Nabis ericetorum* | 11.96 |
| *Nabis flavomarginatus* Scholtz, 1847 | AAG8973 | 7 | 0.31 | 1.01 | *Nabis limbatus* | 11.82 |
| *Nabis limbatus* Dahlbom, 1851 | ABU7333 | 12 | 0.54 | 2.58 | *Nabis flavomarginatus* | 11.82 |
| *Nabis pseudoferus* Remane, 1949 | AAZ3346 | 11 | 0.08 | 0.31 | *Nabis brevis* | 0.15 |
| *Nabis rugosus* (Linnaeus, 1758) | AAZ3346 | 6 | 0.16 | 0.5 | *Nabis brevis* | 0 |
| Anthocoridae |  |  |  |  |  |  |
| *Anthocoris amplicollis* Horváth, 1893 | ABV9164 | 5 | 0.15 | 0.25 | *Anthocoris confusus* | 11.42 |
| *Anthocoris confusus* Reuter, 1884 | ABV9165 | 1 | - | - | *Anthocoris amplicollis* | 11.42 |
| *Anthocoris minki* Dohrn, 1860 | - | 1 | - | - | *Anthocoris confusus* | 14.64 |
| *Anthocoris nemoralis* (Fabricius, 1794) | AAY8896 | 4 | 0 | 0 | *Anthocoris confusus* | 12.23 |
| *Anthocoris nemorum* (Linnaeus, 1761) | AAY9414 | 9 | 0 | 0 | *Anthocoris confusus* | 12.32 |
| *Anthocoris sarothamni* Douglas & Scott, 1865 | ACB9322 | 2 | 0 | 0 | *Anthocoris amplicollis* | 11.83 |
| *Orius laticollis* (Reuter, 1884) | ABA3644 | 2 | 0 | 0 | *Orius niger* | 10.69 |
| *Orius majusculus* (Reuter, 1879) | ABA5781 | 4 | 1.15 | 2.31 | *Orius minutus* | 11.38 |
| *Orius minutus* (Linnaeus, 1758) | - | 16 | 0.03 | 0.25 | *Orius majusculus* | 11.38 |
| *Orius niger* (Wolff, 1811) | ABU8770, ABW5859 | 10 | 4.19 | 8.56 | *Orius laticollis* | 10.69 |
| *Temnostethus gracilis* Horváth, 1907 | ABU5857 | 7 | 0.64 | 1.02 | *Anthocoris confusus* | 12.5 |
| *Temnostethus pusillus* (Herrich-Schaeffer, 1835) | ABV4558 | 1 | - | - | *Anthocoris nemorum* | 14.92 |
| *Xylocoris cursitans* (Fallén, 1807) | ABA4229 | 4 | 0 | 0 | *Orius niger* | 16.73 |
| *Xylocoris galactinus* (Fieber, 1836) | AAY6750 | 1 | - | - | *Xylocoris cursitans* | 16.73 |
| Reduviidae |  |  |  |  |  |  |
| *Coranus subapterus* (De Geer, 1773) | ABC9008 | 2 | 1.52 | 1.52 | *Rhynocoris annulatus* | 15.75 |
| *Empicoris vagabundus* (Linnaeus, 1758) | AAY9215 | 2 | 0 | 0 | *Coranus subapterus* | 25.95 |
| *Reduvius personatus* (Linnaeus, 1758) | AAH2979 | 1 | - | - | *Coranus subapterus* | 23.01 |
| *Rhynocoris annulatus* (Linnaeus, 1758) | AAY9319 | 3 | 0.17 | 0.25 | *Coranus subapterus* | 15.75 |
| *Rhynocoris iracundus* (Poda, 1761) | ACB9169 | 3 | 0.1 | 0.15 | *Coranus subapterus* | 16.81 |
| Phymatidae |  |  |  |  |  |  |
| *Phymata crassipes* (Fabricius, 1775) | AAY9490 | 6 | 0 | 0.03 | - | - |
| Aradidae |  |  |  |  |  |  |
| *Aneurus avenius* (Dufour, 1833) | ABU9082, ABW2173 | 7 | 12.82 | 23.31 | *Aradus conspicuus* | 25.04 |
| *Aradus betulae* (Linnaeus, 1758) | ABV4728 | 4 | 0.68 | 1.45 | *Aradus cinnamomeus* | 20.3 |
| *Aradus cinnamomeus* Panzer, 1806 | ABA3240 | 4 | 0.19 | 0.5 | *Aradus betulae* | 20.3 |
| *Aradus conspicuus* Herrich-Schaeffer, 1835 | ABW4576 | 3 | 0.1 | 0.15 | *Aradus betulae* | 22.33 |
| *Aradus depressus* (Fabricius, 1794) | ABW4547 | 3 | 0.84 | 1.27 | *Aradus betulae* | 20.46 |
| *Mezira tremulae* (Germar, 1822) | ABU9379 | 3 | 0 | 0 | *Aneurus avenius* | 25.24 |
| Lygaeidae |  |  |  |  |  |  |
| *Arocatus longiceps* Stål, 1872 | AAY8974 | 12 | 0.61 | 1.24 | *Arocatus roeselii* | 0 |
| *Arocatus roeselii* (Schilling, 1829) | AAY8974 | 2 | 0 | 0 | *Arocatus longiceps* | 0 |
| *Belonochilus numenius* (Say, 1832) | ABV9619 | 1 | - | - | *Nithecus jacobaeae* | 14.74 |
| *Kleidocerys ericae* (Horváth, 1908) | AAY8761 | 1 | - | - | *Kleidocerys resedae* | 1.71 |
| *Kleidocerys resedae* (Panzer, 1797) | AAY8761 | 14 | 0.15 | 0.49 | *Kleidocerys ericae* | 1.71 |
| *Lygaeus equestris* (Linnaeus, 1758) | ACB9437 | 7 | 0.19 | 0.5 | *Lygaeus simulans* | 4.78 |
| *Lygaeus simulans* Deckert, 1985 | ACB9692 | 1 | 1 | 1 | *Lygaeus equestris* | 4.78 |
| *Nithecus jacobaeae* (Schilling, 1829) | ABV8626 | 5 | 0.18 | 0.31 | *Nysius helveticus* | 12.23 |
| *Nysius cymoides* (Spinola, 1837) | AAN8267 | 1 | - | - | *Nysius ericae* | 5.96 |
| *Nysius ericae* (Schilling, 1829) | ACE3937 | 3 | 0.1 | 0.15 | *Nysius cymoides* | 5.96 |
| *Nysius graminicola* (Kolenati, 1845) | ABV8137 | 2 | 0.46 | 0.46 | *Nysius thymi* | 8.39 |
| *Nysius helveticus* (Herrich-Schaeffer, 1850) | ACB9233 | 3 | 0 | 0 | *Nysius graminicola* | 11.22 |
| *Nysius senecionis* (Schilling, 1829) | ACB9324 | 3 | 0 | 0 | *Nysius graminicola* | 11.58 |
| *Nysius thymi* (Wolff, 1804) | AAZ3385 | 9 | 0.08 | 0.36 | *Nysius graminicola* | 8.39 |
| *Orsillus depressus* (Mulsant & Rey, 1852) | ACA9256 | 1 | - | - | *Nysius helveticus* | 17.42 |
| *Ortholomus punctipennis* (Herrich-Schaeffer, 1838) | ABU6902 | 3 | 0 | 0 | *Belonochilus numenius* | 15.25 |
| *Spilostethus pandurus* (Scopoli 1763) | AAV0102 | 2 | 0 | 0 | *Spilostethus saxatilis* | 9.37 |
| *Spilostethus saxatilis* (Scopoli, 1763) | AAV6179 | 7 | 0.07 | 0.15 | *Spilostethus pandurus* | 9.37 |
| *Tropidothorax leucopterus* (Goeze, 1778) | ACB8914 | 2 | 0.15 | 0.15 | *Arocatus longiceps* | 12.11 |
| Heterogastridae |  |  |  |  |  |  |
| *Platyplax salviae* (Schilling, 1829) | ACA9154 | 4 | 0.63 | 1.27 | - | - |
| Atheneidae |  |  |  |  |  |  |
| *Chilacis typhae* (Perris, 1857) | AAF3704 | 7 | 0 | 0 | - | - |
| Blissidae |  |  |  |  |  |  |
| *Dimorphopterus spinolae* (Signoret, 1857) | ABX0394 | 1 | - | - | *Ischnodemus sabuleti* | 20.73 |
| *Ischnodemus sabuleti* (Fallén, 1826) | ABY6046, AAY9271 | 11 | 0.75 | 2.55 | *Dimorphopterus spinolae* | 20.73 |
| Cymidae |  |  |  |  |  |  |
| *Cymus aurescens* Distant, 1883 | AAY9365 | 6 | 0 | 0 | *Cymus glandicolor* | 1.26 |
| *Cymus claviculus* (Fallén, 1807) | AAY8996 | 3 | 0 | 0 | *Cymus glandicolor* | 13.04 |
| *Cymus glandicolor* Hahn, 1832 | AAY9365 | 5 | 0.35 | 0.72 | *Cymus aurescens* | 1.26 |
| *Cymus melanocephalus* Fieber, 1861 | ABX0395 | 3 | 0 | 0 | *Cymus aurescens* | 11.84 |
| Geocoridae |  |  |  |  |  |  |
| *Geocoris ater* (Fabricius, 1787) | ABV9790 | 1 | - | - | *Geocoris grylloides* | 13.77 |
| *Geocoris dispar* (Waga, 1839) | ABU6682 | 1 | - | - | *Geocoris grylloides* | 9.91 |
| *Geocoris erythrocephalus* (Lepeletier & Serville, 1825) |  | 1 | - | - | *Geocoris grylloides* | 15.3 |
| *Geocoris grylloides* (Linnaeus, 1761) | ABV4861 | 4 | 0.48 | 0.73 | *Geocoris dispar* | 9.91 |
| *Henestaris halophilus* (Burmeister, 1835) | ABW9319 | 1 | - | - | *Henestaris laticeps* | 18.07 |
| *Henestaris laticeps* (Curtis, 1836) | ABV9781 | 1 | - | - | *Henestaris halophilus* | 18.07 |
| Oxycarenidae |  |  |  |  |  |  |
| *Macroplax preyssleri* (Fieber, 1837) | ACA9237 | 3 | 0.1 | 0.15 | *Oxycarenus pallens* | 15.21 |
| *Oxycarenus lavaterae* (Fabricius, 1787) | ABV8673 | 2 | 0 | 0 | *Oxycarenus pallens* | 15.87 |
| *Oxycarenus pallens* (Herrich-Schaeffer, 1850) | AAZ8331 | 2 | 0.31 | 0.31 | *Macroplax preyssleri* | 15.21 |
| *Tropidophlebia costalis* (Herrich-Schaeffer, 1850) | ACA7158 | 1 | - | - | *Macroplax preyssleri* | 16.78 |
| Rhyparochromidae |  |  |  |  |  |  |
| *Acompus rufipes* (Wolff, 1804) | AAY9427 | 4 | 0.31 | 0.63 | *Rhyparochromus pini* | 14.23 |
| *Aellopus atratus* (Goeze, 1778) | ACA7461 | 1 | - | - | *Graptopeltus lynceus* | 12.21 |
| *Beosus maritimus* (Scopoli, 1763) | ABW9272 | 3 | 0.1 | 0.15 | *Megalonotus dilatatus* | 15.03 |
| *Drymus brunneus* (R. F. Sahlberg, 1848) | AAY8994 | 3 | 0.1 | 0.15 | *Drymus ryeii* | 12.33 |
| *Drymus ryeii* Douglas & Scott, 1865 | ABY3202 | 1 | - | - | *Drymus sylvaticus* | 5.12 |
| *Drymus sylvaticus* (Fabricius, 1775) | AAZ3659 | 1 | - | - | *Drymus ryeii* | 5.12 |
| *Emblethis verbasci* (Fabricius, 1803) | ABU6645 | 3 | 0.2 | 0.31 | *Rhyparochromus pini* | 16.82 |
| *Eremocoris abietis* (Linnaeus, 1758) | ABU6590 | 1 | - | - | *Eremocoris plebejus* | 7.29 |
| *Eremocoris plebejus* (Fallén, 1807) | AAY9381 | 8 | 0.41 | 0.93 | *Eremocoris abietis* | 7.29 |
| *Eremocoris podagricus* (Fabricius, 1775) | AAY9382 | 1 | - | - | *Eremocoris plebejus* | 10.05 |
| *Gastrodes abietum* Bergroth, 1914 | AAY9298 | 10 | 0.3 | 1.01 | *Eremocoris plebejus* | 13.52 |
| *Gastrodes grossipes* (De Geer, 1773) | ABV0128 | 8 | 0.08 | 0.25 | *Gastrodes abietum* | 13.77 |
| *Graptopeltus lynceus* (Fabricius, 1775) | ACA7442 | 3 | 0.1 | 0.15 | *Aellopus atratus* | 12.21 |
| *Ischnocoris angustulus* (Boheman, 1852) | - | 2 | 0.25 | 0.25 | *Ischnocoris hemipterus* | 4.9 |
| *Ischnocoris hemipterus* (Schilling, 1829) | ABV5611 | 4 | 0.17 | 0.36 | *Ischnocoris angustulus* | 4.9 |
| *Macrodema microptera* (Curtis, 1836) | AAZ7857 | 7 | 0.04 | 0.15 | *Pionosomus varius* | 11.91 |
| *Megalonotus antennatus* (Schilling, 1829) | AAY9530 | 1 | - | - | *Megalonotus chiragra* | 8.36 |
| *Megalonotus chiragra* (Fabricius, 1794) | AAF4462 | 3 | 0.31 | 0.46 | *Megalonotus sabulicola* | 0.93 |
| *Megalonotus dilatatus* (Herrich-Schaeffer, 1840) | ACA7701 | 2 | 0 | 0 | *Megalonotus sabulicola* | 8.7 |
| *Megalonotus praetextatus* (Herrich-Schaeffer, 1835) | ACA7484 | 2 | 0 | 0 | *Megalonotus chiragra* | 12.4 |
| *Megalonotus sabulicola* (Thomson, 1870) | AAF4462 | 1 | - | - | *Megalonotus chiragra* | 0.93 |
| *Pachybrachius fracticollis* (Schilling, 1829) | AAY9493 | 3 | 0 | 0 | *Megalonotus antennatus* | 14.66 |
| *Panaorus adspersus* (Mulsant & Rey, 1852) | ACA7572 | 1 | - | - | *Megalonotus antennatus* | 14.68 |
| *Peritrechus geniculatus* (Hahn, 1832) | AAY9499 | 8 | 0.11 | 0.5 | *Peritrechus meridionalis* | 9.92 |
| *Peritrechus gracilicornis* Puton, 1877 | AAY9500 | 2 | 0.15 | 0.15 | *Peritrechus meridionalis* | 6.85 |
| *Peritrechus lundii* (Gmelin, 1790) | AAY9501 | 3 | 1.14 | 1.71 | *Rhyparochromus phoeniceus* | 12.67 |
| *Peritrechus meridionalis* Puton, 1877 | ABV8585 | 1 | - | - | *Peritrechus gracilicornis* | 6.85 |
| *Pionosomus varius* (Wolff, 1804) | ABW9656 | 1 | - | - | *Macrodema microptera* | 11.91 |
| *Plinthisus pusillus* (Scholtz, 1847) | ACA7688 | 2 | 0.15 | 0.15 | *Eremocoris abietis* | 17.93 |
| *Pterotmetus staphyliniformis* (Schilling, 1829) | AAY9334 | 3 | 0.1 | 0.15 | *Macrodema microptera* | 12.97 |
| *Raglius alboacuminatus* (Goeze, 1778) | ABW8820, ACA7459 | 3 | 1.47 | 2.2 | *Sphragisticus nebulosus* | 13.81 |
| *Rhyparochromus phoeniceus* (Rossi, 1794) | ACA7561 | 3 | 0.2 | 0.31 | *Rhyparochromus pini* | 10.05 |
| *Rhyparochromus pini* (Linnaeus, 1758) | AAY9318 | 11 | 0.24 | 0.5 | *Rhyparochromus phoeniceus* | 10.05 |
| *Rhyparochromus vulgaris* (Schilling, 1829) | AAY8938 | 9 | 0.21 | 0.62 | *Rhyparochromus pini* | 11.28 |
| *Scolopostethus affinis* (Schilling, 1829) | AAZ2038 | 1 | - | - | *Scolopostethus decoratus* | 4.29 |
| *Scolopostethus decoratus* (Hahn, 1833) | AAY8932 | 2 | 0.31 | 0.31 | *Scolopostethus affinis* | 4.29 |
| *Scolopostethus pictus* (Schilling, 1829) | AAY8931 | 4 | 0.17 | 0.34 | *Scolopostethus decoratus* | 12.8 |
| *Scolopostethus thomsoni* Reuter, 1875 | AAZ2037 | 8 | 0.04 | 0.16 | *Scolopostethus affinis* | 6.92 |
| *Sphragisticus nebulosus* (Fallén, 1807) | ABW7046 | 1 | - | - | *Xanthochilus quadratus* | 12.64 |
| *Stygnocoris cimbricus* (Gredler, 1870) | ACA7369 | 1 | - | - | *Stygnocoris sabulosus* | 13.97 |
| *Stygnocoris fuligineus* (Geoffroy, 1785) | ACA7292 | 3 | 0.31 | 0.31 | *Stygnocoris sabulosus* | 14.35 |
| *Stygnocoris rusticus* (Fallén, 1807) | AAE2297 | 6 | 0.26 | 0.77 | *Stygnocoris cimbricus* | 15.19 |
| *Stygnocoris sabulosus* (Schilling, 1829) | AAD5142 | 7 | 0.05 | 0.25 | *Stygnocoris cimbricus* | 13.97 |
| *Trapezonotus arenarius* (Linnaeus, 1758) | ABA2811 | 2 | 0.62 | 0.62 | *Trapezonotus dispar* | 0.15 |
| *Trapezonotus dispar* Stål, 1872 | ABA2811 | 5 | 0.06 | 0.15 | *Trapezonotus arenarius* | 0.15 |
| *Tropistethus holosericeus* (Scholtz, 1846) | ABV4591 | 1 | - | - | *Pachybrachius fracticollis* | 18.06 |
| *Xanthochilus quadratus* (Fabricius, 1798) | ABW9570 | 4 | 0.31 | 0.46 | *Sphragisticus nebulosus* | 12.64 |
| Piesmatidae |  |  |  |  |  |  |
| *Parapiesma quadratum* (Fieber, 1844) | ACA7368 | 1 | - | - | *Piesma maculatum* | 15.28 |
| *Piesma maculatum* (Laporte, 1833) | AAZ9928 | 10 | 0.06 | 0.31 | *Parapiesma quadratum* | 15.28 |
| Berytidae |  |  |  |  |  |  |
| *Berytinus clavipes* (Fabricius, 1775) | AAY9033 | 6 | 0.13 | 0.31 | *Berytinus minor* | 10.89 |
| *Berytinus crassipes* (Herrich-Schaeffer, 1835) | ABU9152 | 5 | 0.15 | 0.31 | *Berytinus montivagus* | 10.04 |
| *Berytinus minor* (Herrich-Schaeffer, 1835) | AAG5709 | 8 | 0.19 | 0.5 | *Berytinus clavipes* | 10.89 |
| *Berytinus montivagus* (Meyer-Dür, 1841) | ACA7025 | 2 | 0 | 0 | *Berytinus crassipes* | 10.04 |
| *Berytinus signoreti* (Fieber, 1859) | ACA7238 | 2 | 0 | 0 | *Berytinus crassipes* | 14.53 |
| *Gampsocoris punctipes* (Germar, 1822) | ABV9254 | 3 | 0.31 | 0.46 | *Neides tipularius* | 14.5 |
| *Metatropis rufescens* (Herrich-Schaeffer, 1835) | ABV5708 | 4 | 0.08 | 0.17 | *Neides tipularius* | 10.66 |
| *Neides tipularius* (Linnaeus, 1758) | ACA7003 | 6 | 0.13 | 0.31 | *Metatropis rufescens* | 10.66 |
| Pyrrhocoridae |  |  |  |  |  |  |
| *Pyrrhocoris apterus* (Linnaeus, 1758) | AAY8951 | 14 | 0 | 0.01 | *-* | - |
| Alydidae |  |  |  |  |  |  |
| *Alydus calcaratus* (Linnaeus, 1758) | AAZ6465 | 12 | 0 | 0 | *-* | - |
| Coreidae |  |  |  |  |  |  |
| *Arenocoris fallenii* (Schilling, 1829) | ACA6981 | 3 | 1.14 | 1.71 | *Ceraleptus gracilicornis* | 13.62 |
| *Bathysolen nubilus* (Fallén, 1807) | ACA7092 | 4 | 0.41 | 0.62 | *Coriomeris denticulatus* | 8.71 |
| *Ceraleptus gracilicornis* (Herrich-Schaeffer, 1835) | ABW9608 | 2 | 0.77 | 0.77 | *Ceraleptus lividus* | 9.28 |
| *Ceraleptus lividus* Stein, 1858 | ACA7307 | 1 | - | - | *Ceraleptus gracilicornis* | 9.28 |
| *Coreus marginatus* (Linnaeus, 1758) | AAY8971 | 8 | 0.4 | 0.62 | *Coriomeris denticulatus* | 16.52 |
| *Coriomeris denticulatus* (Scopoli, 1763) | ABU9164 | 9 | 1.28 | 2.34 | *Bathysolen nubilus* | 8.71 |
| *Enoplops scapha* (Fabricius, 1794) | ABW9378 | 5 | 0.31 | 0.31 | *Coriomeris denticulatus* | 15.6 |
| *Gonocerus acuteangulatus* (Goeze, 1778) | AAZ9908 | 6 | 0.14 | 0.31 | *Gonocerus juniperi* | 9.05 |
| *Gonocerus juniperi* Herrich-Schaeffer, 1839 | AAY9515 | 6 | 0.1 | 0.31 | *Gonocerus acuteangulatus* | 9.05 |
| *Leptoglossus occidentalis* Heidemann, 1910 | AAE3160 | 2 | 0 | 0 | *Coriomeris denticulatus* | 16.28 |
| *Spathocera dalmanii* (Schilling, 1829) | ABW9211 | 3 | 0.51 | 0.77 | *Enoplops scapha* | 16.18 |
| *Syromastus rhombeus* (Linnaeus, 1767) | ABX4334 | 4 | 0.08 | 0.15 | *Coriomeris denticulatus* | 15.39 |
| Rhopalidae |  |  |  |  |  |  |
| *Brachycarenus tigrinus* (Schilling, 1829) | AAD4531 | 1 | - | - | *Rhopalus parumpunctatus* | 13.62 |
| *Chorosoma schillingii* (Schilling, 1829) | ABV9616 | 1 | - | - | *Myrmus miriformis* | 10.6 |
| *Corizus hyoscyami* (Linnaeus, 1758) | AAY8972 | 8 | 0.11 | 0.31 | *Rhopalus parumpunctatus* | 11.85 |
| *Liorhyssus hyalinus* (Fabricius, 1794) | AAG8881 | 3 | 0.82 | 1.08 | *Rhopalus parumpunctatus* | 14.1 |
| *Myrmus miriformis* (Fallén, 1807) | AAZ3702 | 7 | 0.03 | 0.16 | *Chorosoma schillingii* | 10.6 |
| *Rhopalus conspersus* (Fieber, 1837) | ABV9473 | 1 | - | - | *Rhopalus subrufus* | 12.27 |
| *Rhopalus maculatus* (Fieber, 1837) | AAY9321 | 3 | 0 | 0 | *Myrmus miriformis* | 14.21 |
| *Rhopalus parumpunctatus* Schilling, 1829 | AAY8957 | 8 | 0.08 | 0.31 | *Corizus hyoscyami* | 11.85 |
| *Rhopalus subrufus* (Gmelin, 1790) | AAY9322 | 2 | 0.15 | 0.15 | *Rhopalus conspersus* | 12.27 |
| *Stictopleurus abutilon (*Rossi, 1790) | AAY9315, AAZ3130 | 6 | 1.89 | 5.11 | *Stictopleurus pictus* | 6.77 |
| *Stictopleurus crassicornis* (Linnaeus, 1758) | ABA4879 | 8 | 0.22 | 0.48 | *Stictopleurus abutilon* | 10.87 |
| *Stictopleurus pictus* (Fieber, 1861) | ABA4878 | 2 | 0 | 0 | *Stictopleurus abutilon* | 6.77 |
| *Stictopleurus punctatonervosus* (Goeze, 1778) | AAZ3560 | 5 | 0.12 | 0.31 | *Stictopleurus abutilon* | 11.56 |
| Stenocephalidae |  |  |  |  |  |  |
| *Dicranocephalus medius* (Mulsant & Rey, 1870) | AAY9391 | 1 | - | - | *-* | - |
| Plataspidae |  |  |  |  |  |  |
| *Coptosoma scutellatum* (Geoffroy, 1785) | AAC4357 | 2 | 0 | 0 | *-* | - |
| Cydnidae |  |  |  |  |  |  |
| *Legnotus limbosus* (Geoffroy, 1785) | AAZ0138 | 1 | - | - | *Tritomegas sexmaculatus* | 20.47 |
| *Microporus nigrita* (Fabricius, 1794) | ABW9452 | 1 | - | - | *Tritomegas bicolor* | 20.96 |
| *Tritomegas bicolor* (Linnaeus, 1758) | AAY8975 | 2 | 0.15 | 0.15 | *Tritomegas sexmaculatus* | 15.88 |
| *Tritomegas sexmaculatus* (Rambur, 1839) | ABW8798 | 2 | 0.62 | 0.62 | *Tritomegas bicolor* | 15.88 |
| Scutelleridae |  |  |  |  |  |  |
| *Eurygaster austriaca* (Schrank, 1776) | - | 1 | - | - | *Eurygaster maura* | 8.68 |
| *Eurygaster maura* (Linnaeus, 1758) | AAZ3231 | 5 | 0.09 | 0.31 | *Eurygaster testudinaria* | 0 |
| *Eurygaster testudinaria* (Geoffroy, 1785) | AAZ3231 | 5 | 0.17 | 0.5 | *Eurygaster maura* | 0 |
| *Odontotarsus purpureolineatus* (Rossi, 1790) | ABW9503 | 1 | - | - | *Eurygaster maura* | 16.16 |
| Pentatomidae |  |  |  |  |  |  |
| *Aelia acuminata* (Linnaeus, 1758) | AAY9083 | 10 | 0.08 | 0.52 | *Neottiglossa leporina* | 13.2 |
| *Aelia klugii* Hahn, 1833 | ABV4564 | 1 | - | - | *Neottiglossa leporina* | 12.34 |
| *Arma custos* (Fabricius, 1794) | ABV9588 | 1 | - | - | *Troilus luridus* | 10.91 |
| *Carpocoris fuscispinus* (Boheman, 1851) | ACD1181 | 4 | 0.63 | 1.27 | *Carpocoris purpureipennis* | 10.51 |
| *Carpocoris purpureipennis* (De Geer, 1773) | ABV3440 | 5 | 0.14 | 0.51 | *Carpocoris fuscispinus* | 10.51 |
| *Chlorochroa juniperina* (Linnaeus, 1758) | ABV5200 | 1 | - | - | *Chlorochroa pinicola* | 0.93 |
| *Chlorochroa pinicola* (Mulsant & Rey, 1852) | ABV5200 | 2 | 0 | 0 | *Chlorochroa juniperina* | 0.93 |
| *Dolycoris baccarum* (Linnaeus, 1758) | AAP3525 | 8 | 0.7 | 1.55 | *Neottiglossa leporina* | 15.9 |
| *Eurydema dominulus* (Scopoli, 1763) | AAF6431 | 1 | - | - | *Eurydema ornata* | 6.56 |
| *Eurydema oleracea* (Linnaeus, 1758) | ABY3233 | 6 | 0.33 | 0.76 | *Eurydema ornata* | 3.09 |
| *Eurydema ornata* (Linnaeus, 1758) | - | 2 | 0.5 | 0.5 | *Eurydema oleracea* | 3.09 |
| *Eysarcoris aeneus* (Scopoli, 1763) | ABW5893 | 5 | 0.06 | 0.15 | *Zicrona caerulea* | 15.08 |
| *Eysarcoris venustissimus* (Schrank, 1776) | ACJ5848 | 2 | 0 | 0 | *Piezodorus lituratus* | 14.63 |
| *Graphosoma lineatum* (Linnaeus, 1758) | AAY9133 | 9 | 0.26 | 0.79 | *Piezodorus lituratus* | 12.84 |
| *Holcostethus strictus* (Wolff, 1804) | AAY9503 | 4 | 0.46 | 0.76 | *Zicrona caerulea* | 14.87 |
| *Neottiglossa leporina* (Herrich-Schaeffer, 1830) | ABX0380 | 2 | 0.31 | 0.31 | *Piezodorus lituratus* | 12.11 |
| *Neottiglossa pusilla* (Gmelin, 1790) | AAY8954 | 2 | 0.46 | 0.46 | *Neottiglossa leporina* | 12.74 |
| *Palomena prasina* (Linnaeus, 1761) | AAG8727 | 8 | 0.48 | 0.76 | *Neottiglossa leporina* | 12.71 |
| *Pentatoma rufipes* (Linnaeus, 1758) | AAZ7767 | 8 | 0.19 | 0.89 | *Eurydema dominulus* | 11.97 |
| *Picromerus bidens* (Linnaeus, 1758) | AAV0223 | 2 | 0 | 0 | *Neottiglossa leporina* | 15.11 |
| *Piezodorus lituratus* (Fabricius, 1794) | AAY9491 | 6 | 0.65 | 0.93 | *Neottiglossa leporina* | 12.11 |
| *Podops inunctus* (Fabricius, 1775) | ABV5501 | 1 | - | - | *Piezodorus lituratus* | 13.91 |
| *Rhacognathus punctatus* (Linnaeus, 1758) | ABU5656 | 1 | - | - | *Eurydema dominulus* | 11.29 |
| *Rhaphigaster nebulosa* (Poda, 1761) | AAY8964 | 8 | 0.03 | 0.15 | *Arma custos* | 11.82 |
| *Sciocoris cursitans* (Fabricius, 1794) | AAZ7101 | 5 | 0.49 | 1.23 | *Neottiglossa leporina* | 15.63 |
| *Sciocoris homalonotus* Fieber, 1851 | - | 1 | - | - | *Sciocoris umbrinus* | 3.09 |
| *Sciocoris umbrinus* (Wolff, 1804) | - | 1 | - | - | *Sciocoris homalonotus* | 3.09 |
| *Troilus luridus* (Fabricius, 1775) | ABX8078, AAY9349 | 2 | 2.66 | 2.66 | *Arma custos* | 10.91 |
| *Zicrona caerulea* (Linnaeus, 1758) | AAZ6776 | 1 | - | - | *Arma custos* | 11.45 |
| Acanthosomatidae |  |  |  |  |  |  |
| *Acanthosoma haemorrhoidale* (Linnaeus, 1758) | AAJ3747 | 5 | 0 | 0 | *Elasmostethus minor* | 14.57 |
| *Cyphostethus tristriatus* (Fabrcius, 1787) | - | 1 | - | - | *Elasmostethus minor* | 17.52 |
| *Elasmostethus interstinctus* (Linnaeus, 1758) | ABZ2225 | 3 | 0 | 0 | *Elasmostethus minor* | 0 |
| *Elasmostethus minor* Horváth, 1899 | ABZ2225 | 1 | - | - | *Elasmostethus interstinctus* | 0 |
| *Elasmucha ferrugata* (Fabricius, 1787) | ABA7122 | 4 | 0.25 | 0.51 | *Elasmucha grisea* | 14.85 |
| *Elasmucha grisea* (Linnaeus, 1758) | AAY9405 | 3 | 0.23 | 0.35 | *Elasmucha ferrugata* | 14.85 |
