# Supplementary material for: Building-Up of a DNA Barcode Library for True Bugs (Insecta: Hemiptera: Heteroptera) of Germany Reveals Taxonomic Uncertainties and Surprises
Source: PLoS One. 2014 Sep 9;9(9):e106940. doi: 10.1371/journal.pone.0106940 (PMC4159288; doi:10.1371/journal.pone.0106940)
Supplement: Appendix S7 — The distribution of sequence divergence within species and within genera based on the given classification. All tables and histograms were calculated using the BOLD V3.0 working bench. (DOCX) [file pone.0106940.s007.docx]

**Intra- and interspecific distance calculations**

The distribution of sequence divergence within species and within genera based on the given classification of analyzed specimens is summarized below and visualized as histograms (A: within species, B: within genus).

|  | **n** | **taxa** | **comparisons** | **Min. distance (%)** | **Mean distance (%)** | **Max. distance (%)** | **SE distance (%)** |
| --- | --- | --- | --- | --- | --- | --- | --- |
| **Within species** | 1614 | 329 | 4493 | 0 | 0.45 | 23.31 | 0 |
| **Within genus** | 1253 | 97 | 8979 | 0 | 13.59 | 27.67 | 0 |

A:


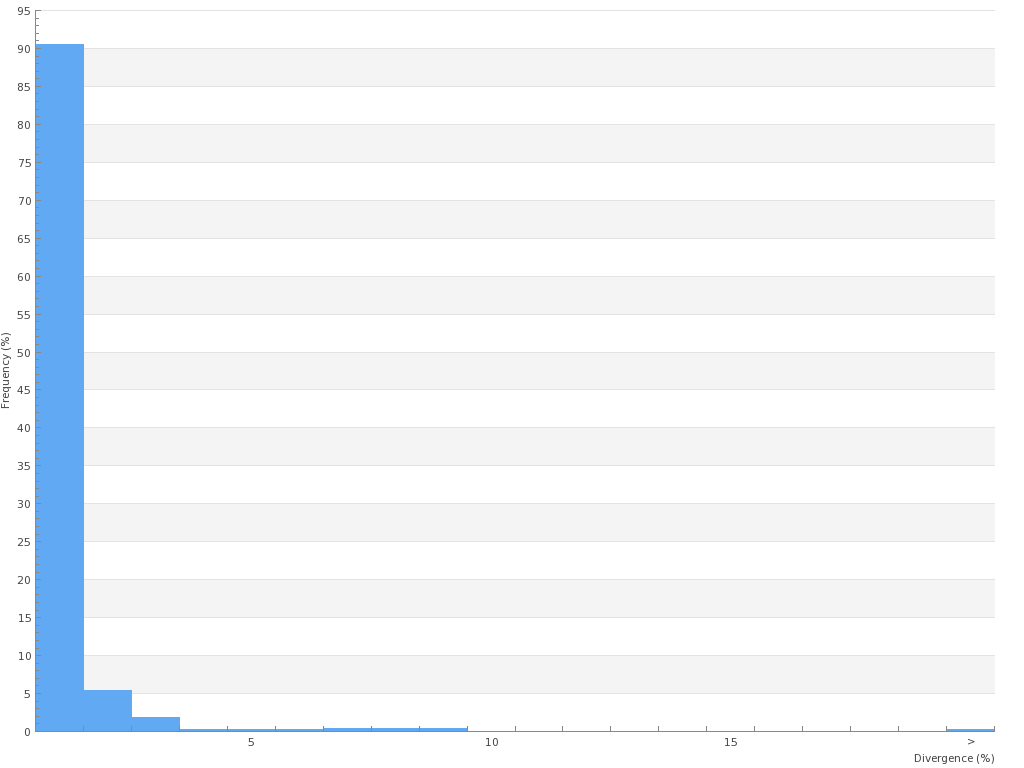


B:


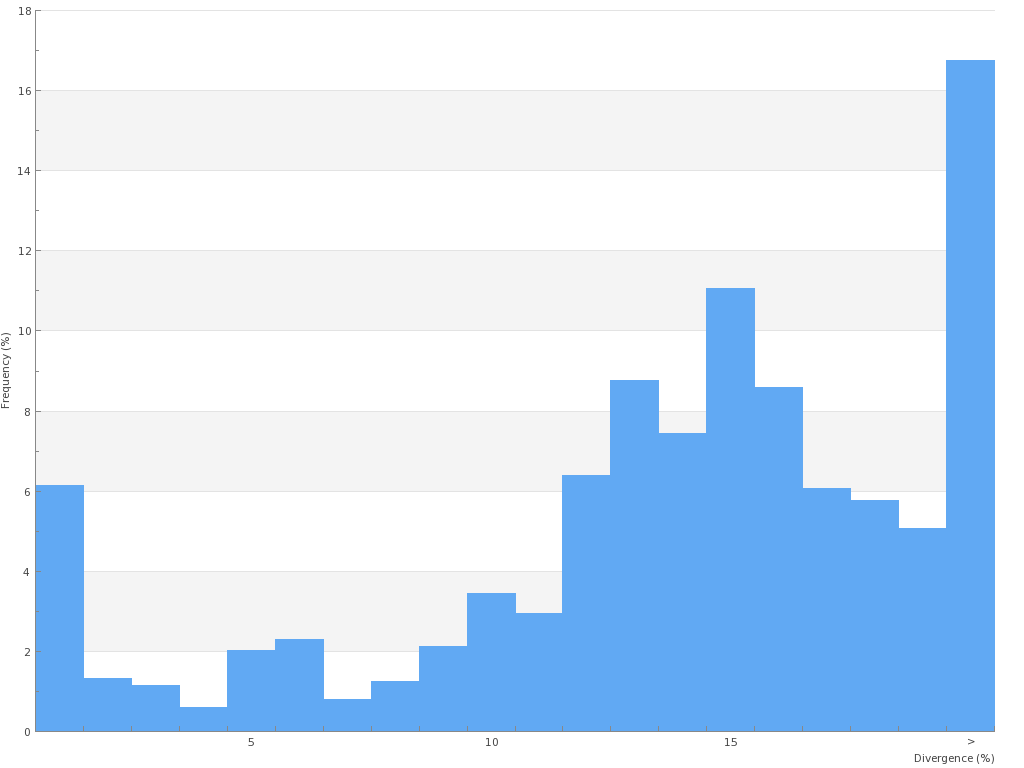


The within-species distribution is normalized to reduce bias in sampling at the species level. The table below summarizes this distribution, while the histogram plots the distribution of normalized divergence for species (intraspecific, pink) against the genus divergences (interspecific, green).

Species count: 329

Mean within-species distance (%): 0.41

SE of mean within-species distance (%): 0

Minimum between-species distance (%): 0


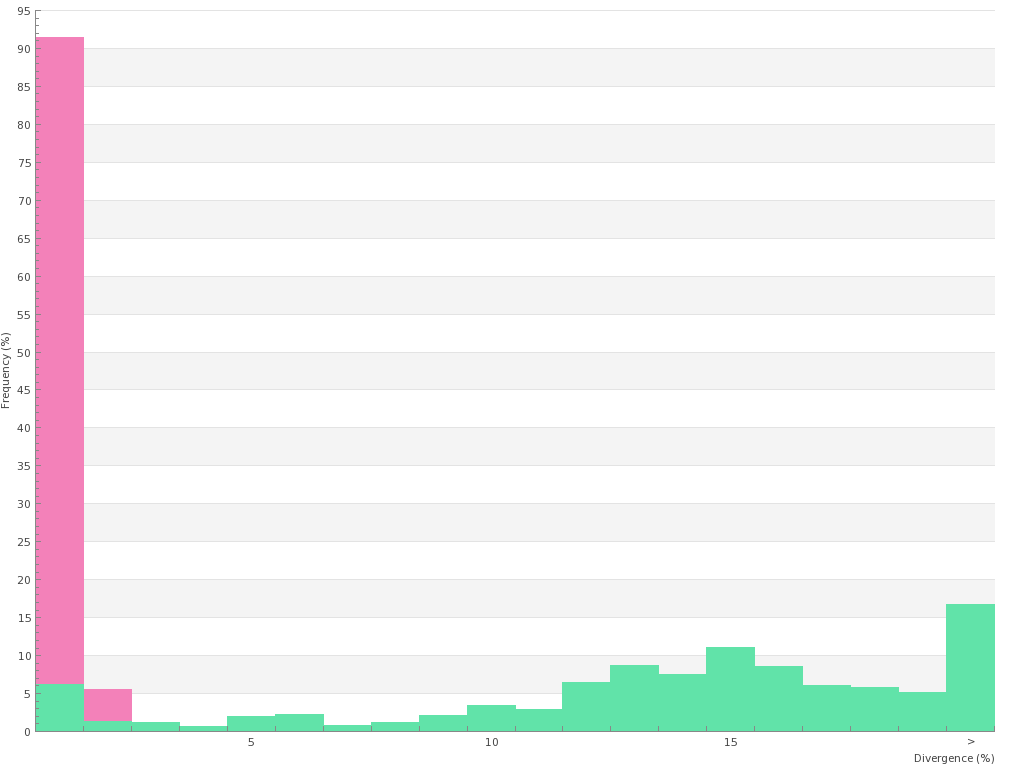


All tables and histograms were calculated using the BOLD V3.0 working bench.
