# Supplementary material for: Building-Up of a DNA Barcode Library for True Bugs (Insecta: Hemiptera: Heteroptera) of Germany Reveals Taxonomic Uncertainties and Surprises
Source: PLoS One. 2014 Sep 9;9(9):e106940. doi: 10.1371/journal.pone.0106940 (PMC4159288; doi:10.1371/journal.pone.0106940)
Supplement: Appendix S8 — Matrix plots of the ABGD results The image plots show species names on the y- and the intraspecific divergence prior on the x-axis. For each plot, the relative gap width X is constant (minimum and maximum X values are shown). The image plots were produced automatically by discriminating different cases. Possible cases are i) all sequences of one species are found in one single group (red), or ii) sequences that belong to one species are found in at least two groups (blue). (PDF) [file pone.0106940.s008.pdf]

Anthocoridae X=0.1

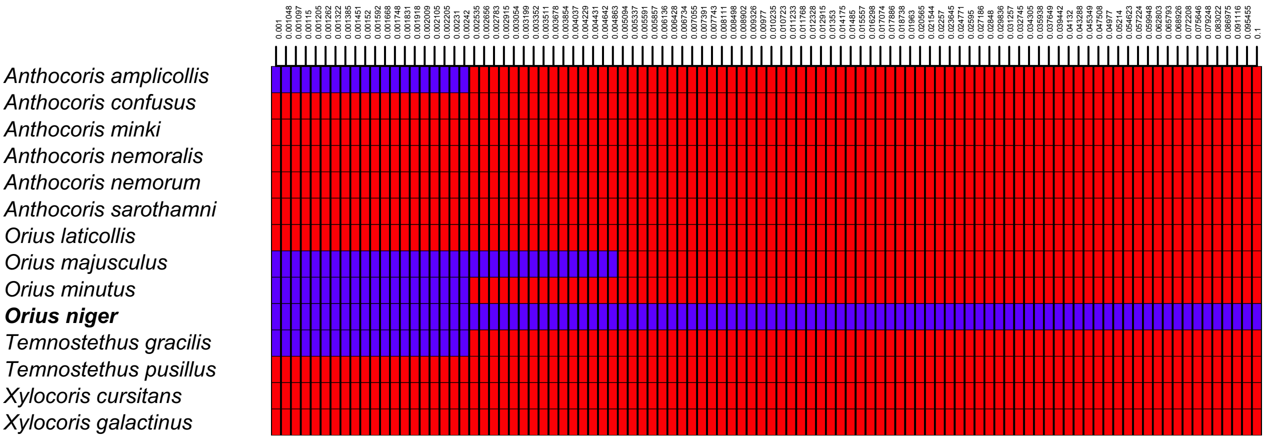

Anthocoridae X=1

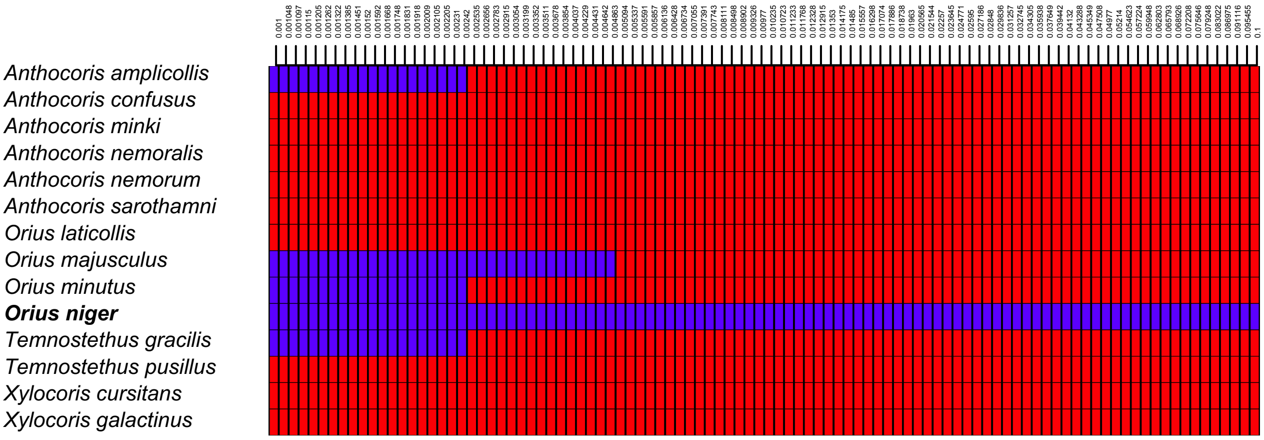

Aradidae X=0.1

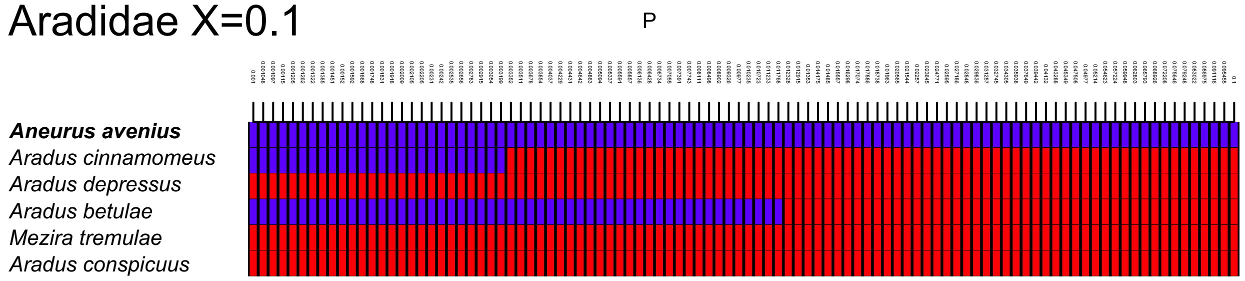

Aradidae X=1.1

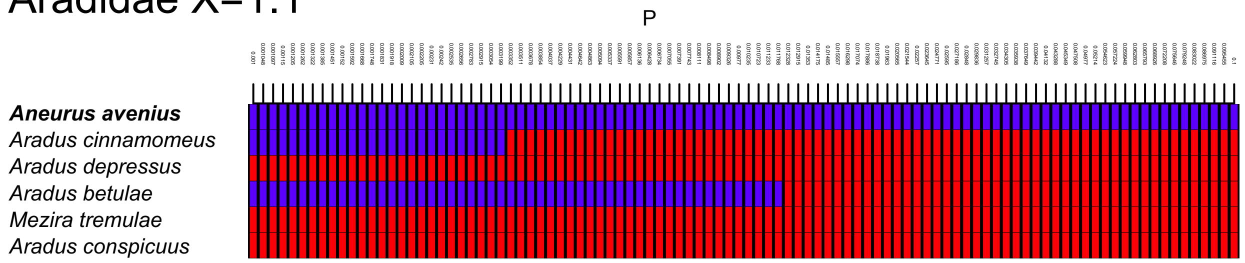

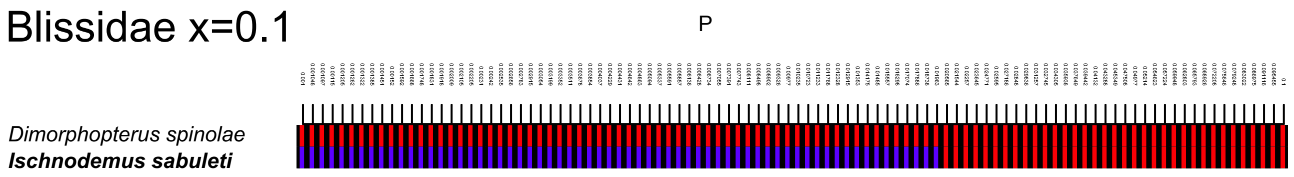
Blissidae x=0.1
P
Dimorphopterus spinolae
Ischnodemus sabuleti

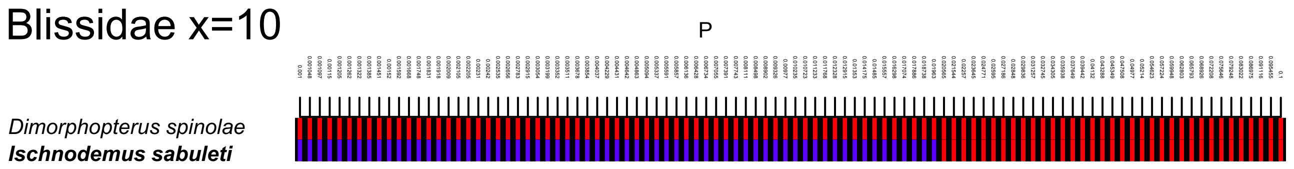
Blissidae x=10
P
Dimorphopterus spinolae
Ischnodemus sabuleti

Corixidae X=0.1

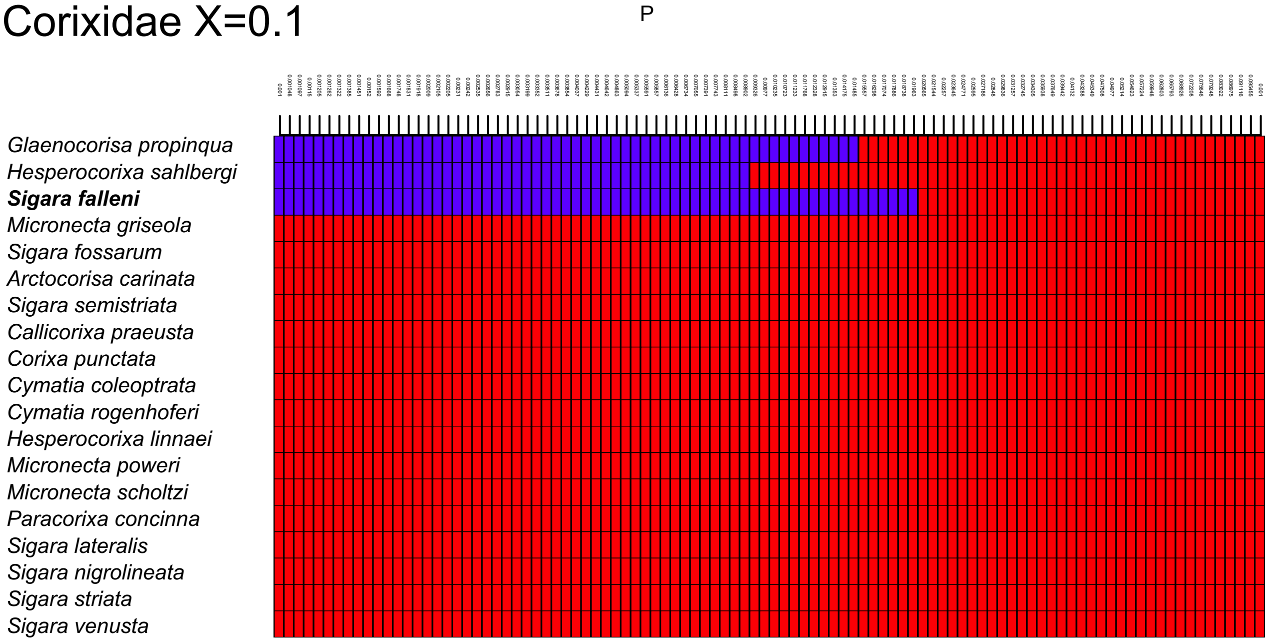

Corixidae X=1.1

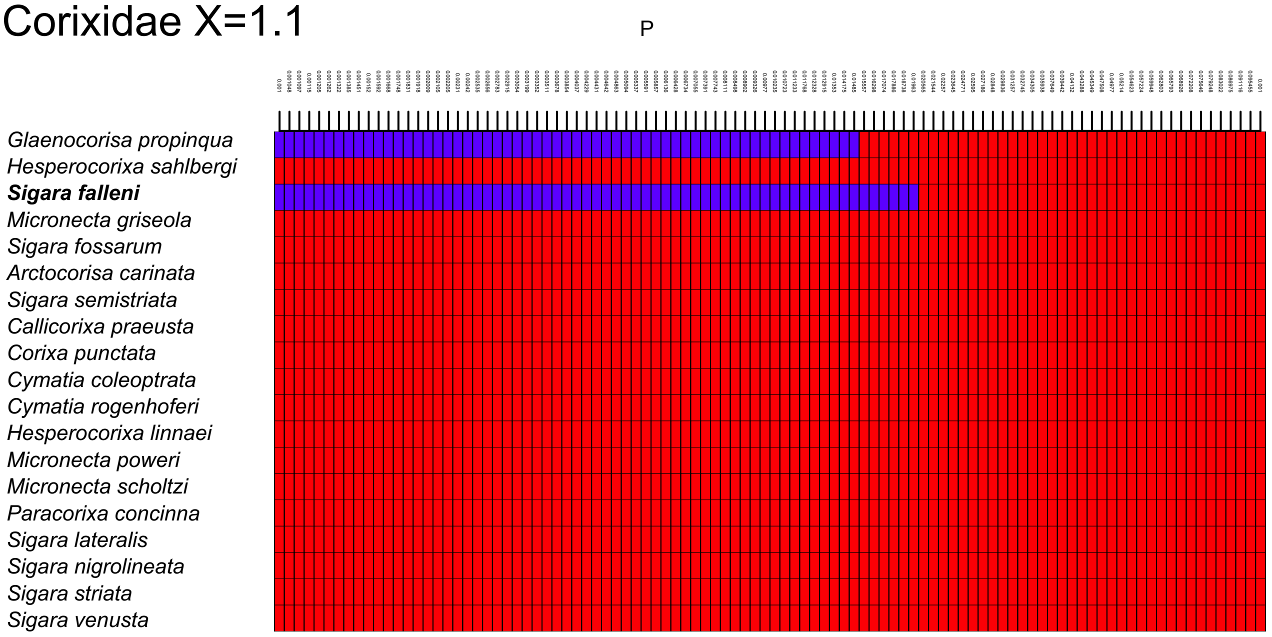

Miridae X=0.1

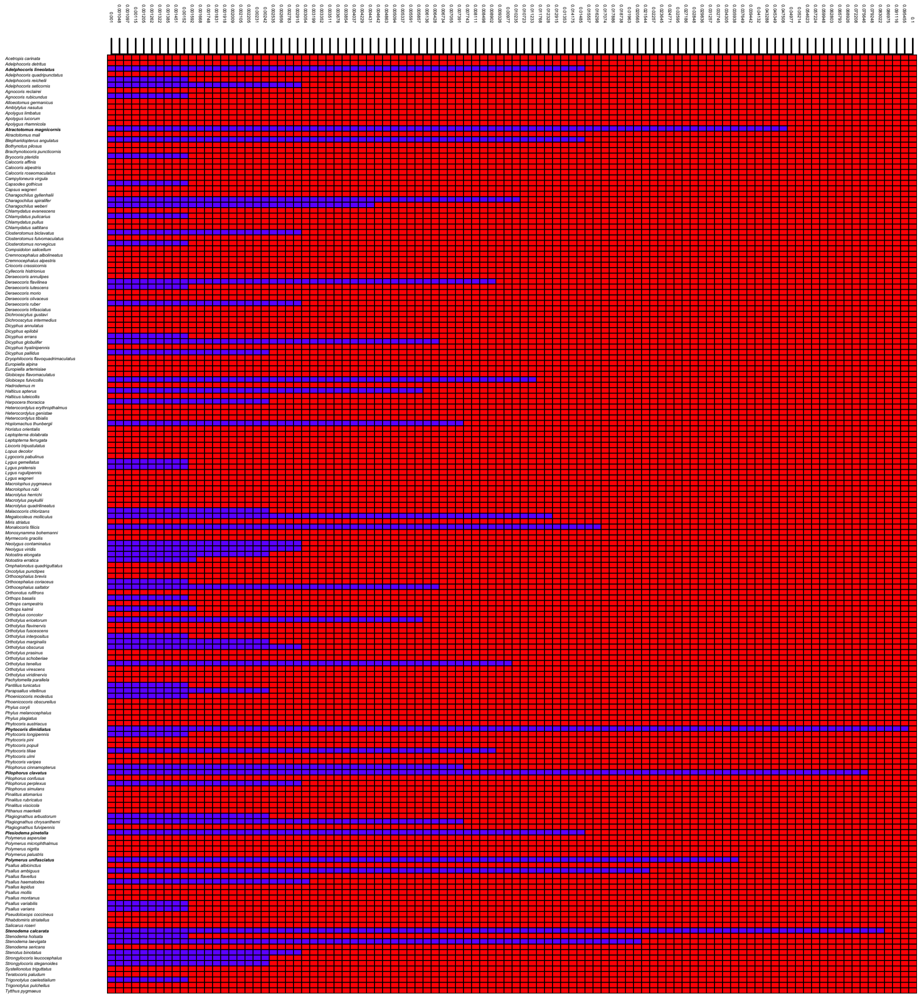

Miridae X=10

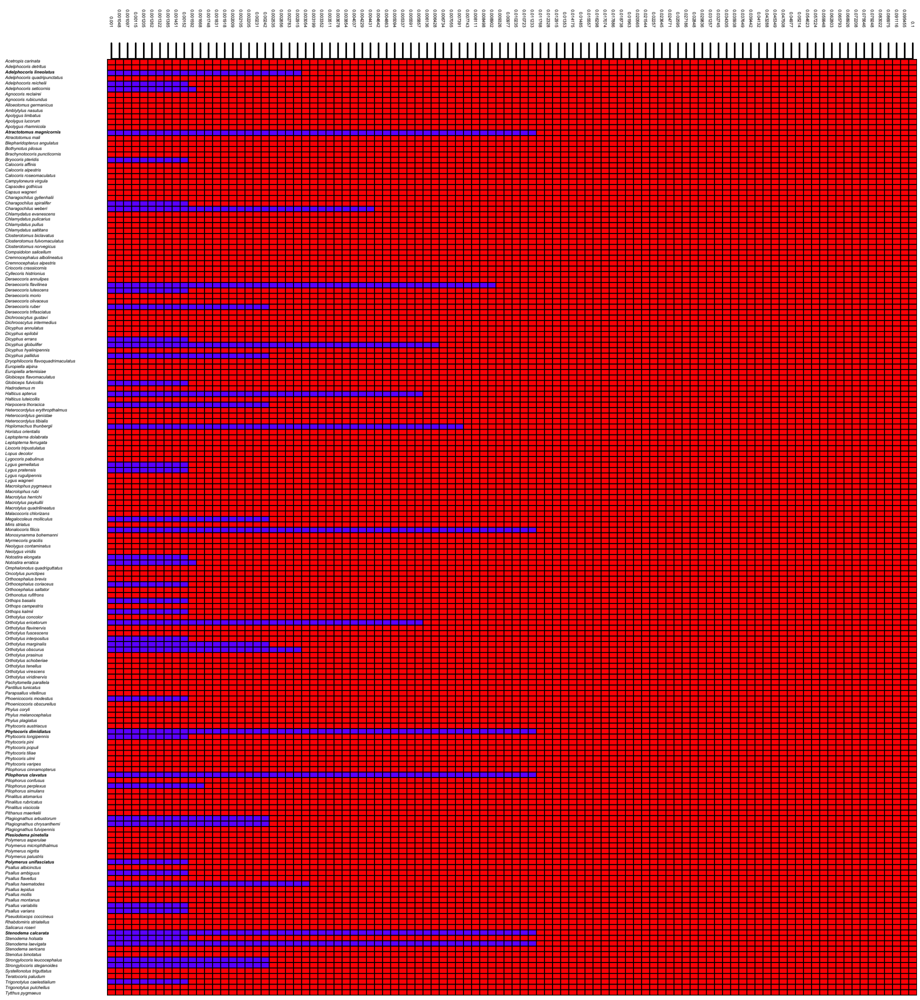

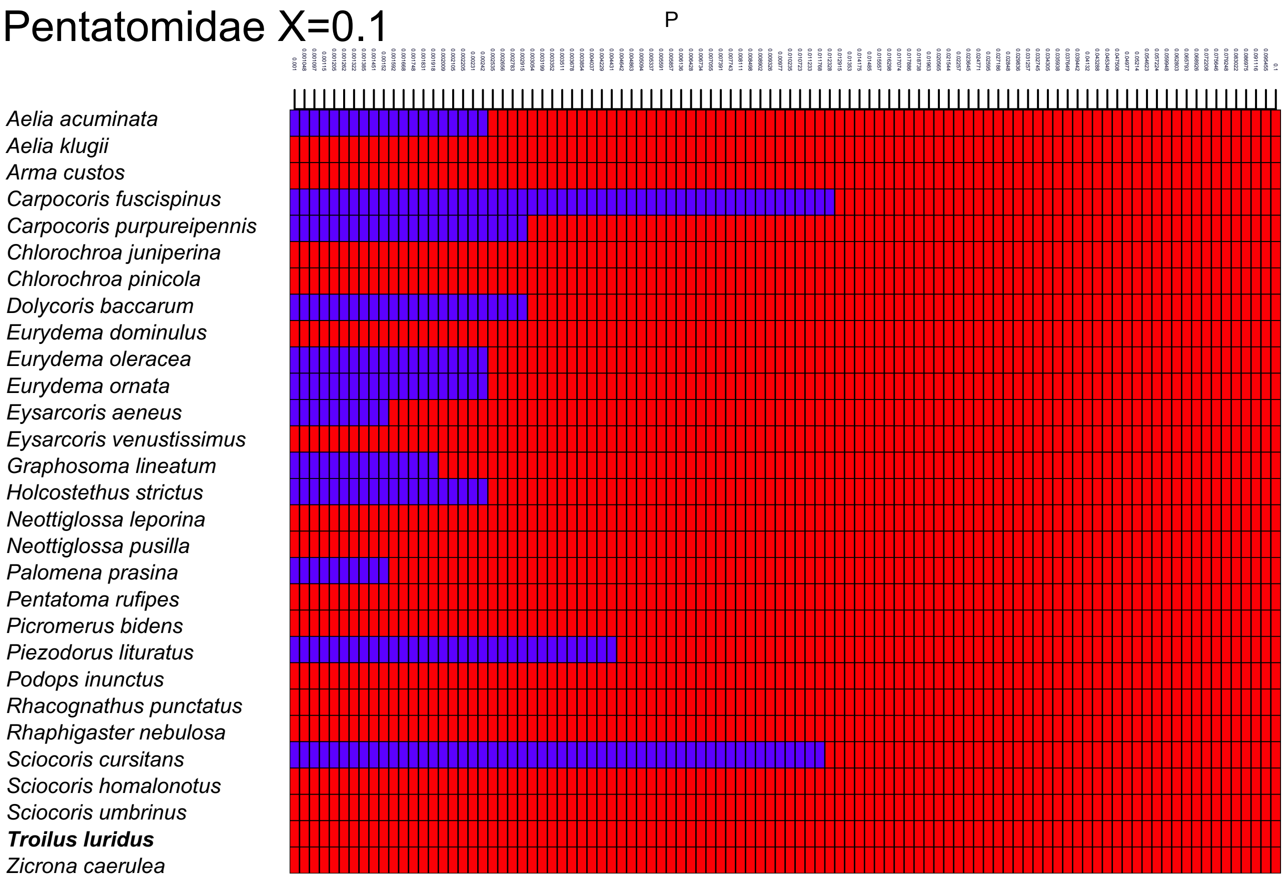
Pentatomidae X=0.1
P
Aelia acuminata
Aelia klugii
Arma custos
Carpocoris fuscispinus
Carpocoris purpureipennis
Chlorochroa juniperina
Chlorochroa pinicola
Dolycoris baccarum
Eurydema dominulus
Eurydema oleracea
Eurydema ornata
Eysarcoris aeneus
Eysarcoris venustissimus
Graphosoma lineatum
Holcostethus strictus
Neottiglossa leporina
Neottiglossa pusilla
Palomena prasina
Pentatoma rufipes
Picromerus bidens
Piezodorus lituratus
Podops inunctus
Rhacognathus punctatus
Rhaphigaster nebulosa
Sciocoris cursitans
Sciocoris homalonotus
Sciocoris umbrinus
Troilus luridus
Zicrona caerulea

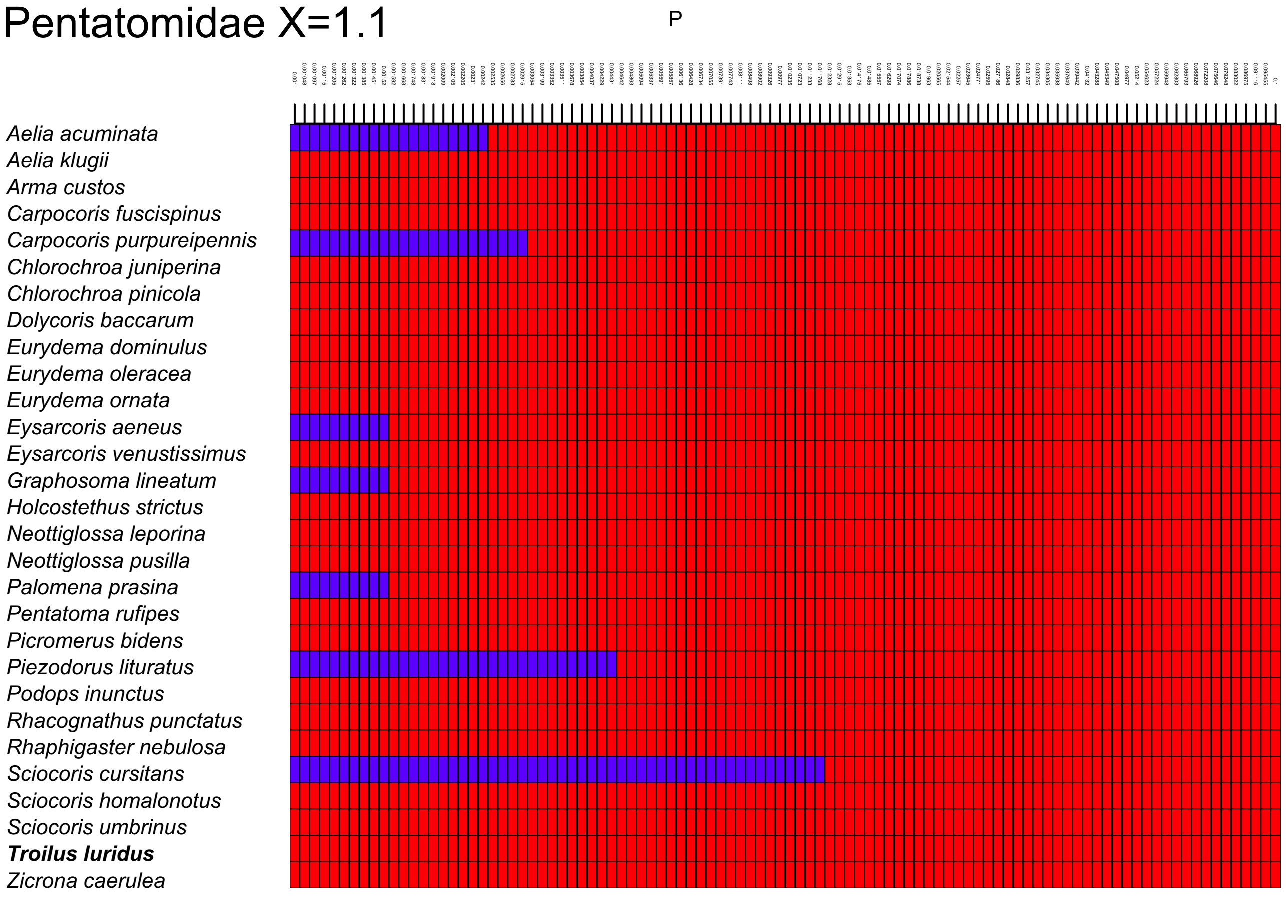
Pentatomidae X=1.1
P
Aelia acuminata
Aelia klugii
Arma custos
Carpocoris fuscispinus
Carpocoris purpureipennis
Chlorochroa juniperina
Chlorochroa pinicola
Dolycoris baccarum
Eurydema dominulus
Eurydema oleracea
Eurydema ornata
Eysarcoris aeneus
Eysarcoris venustissimus
Graphosoma lineatum
Holcostethus strictus
Neottiglossa leporina
Neottiglossa pusilla
Palomena prasina
Pentatoma rufipes
Picromerus bidens
Piezodorus lituratus
Podops inunctus
Rhacognathus punctatus
Rhaphigaster nebulosa
Sciocoris cursitans
Sciocoris homalonotus
Sciocoris umbrinus
Troilus luridus
Zicrona caerulea

Rhopalidae X=0.1

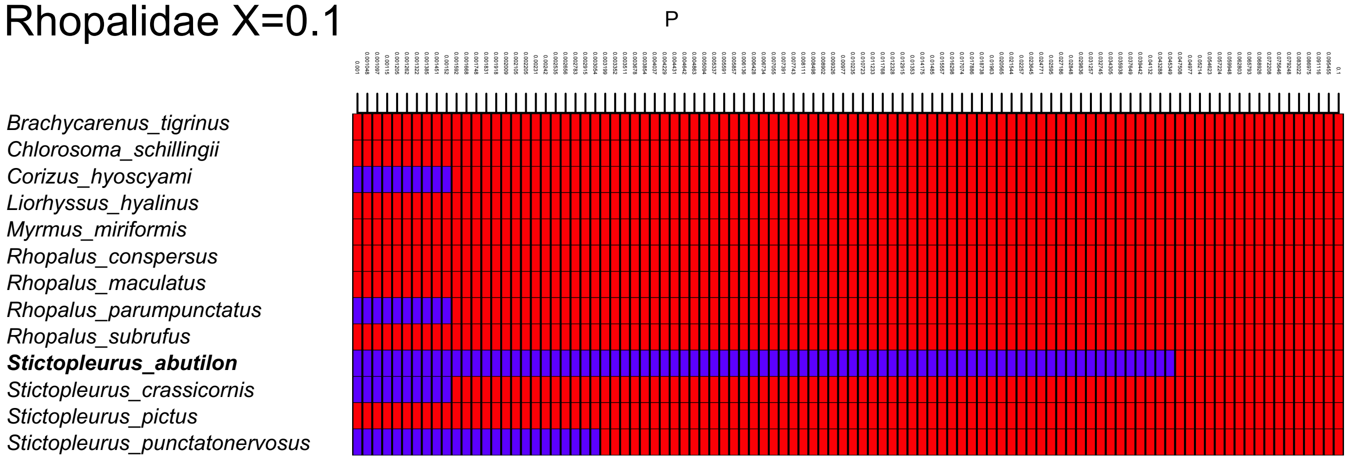

Rhopalidae X=1.1

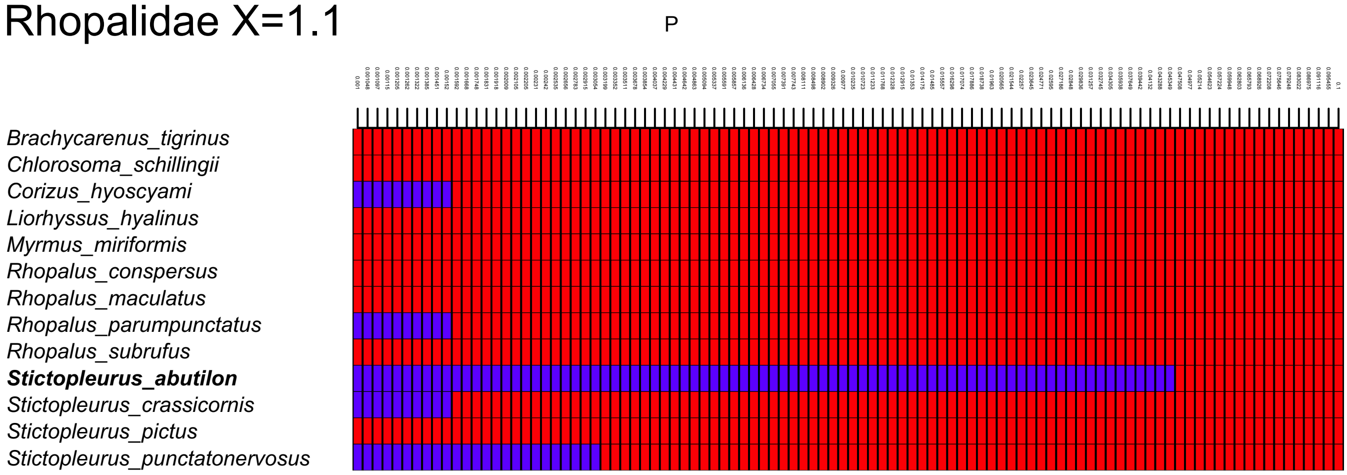

Rhyparochromidae X=0.1

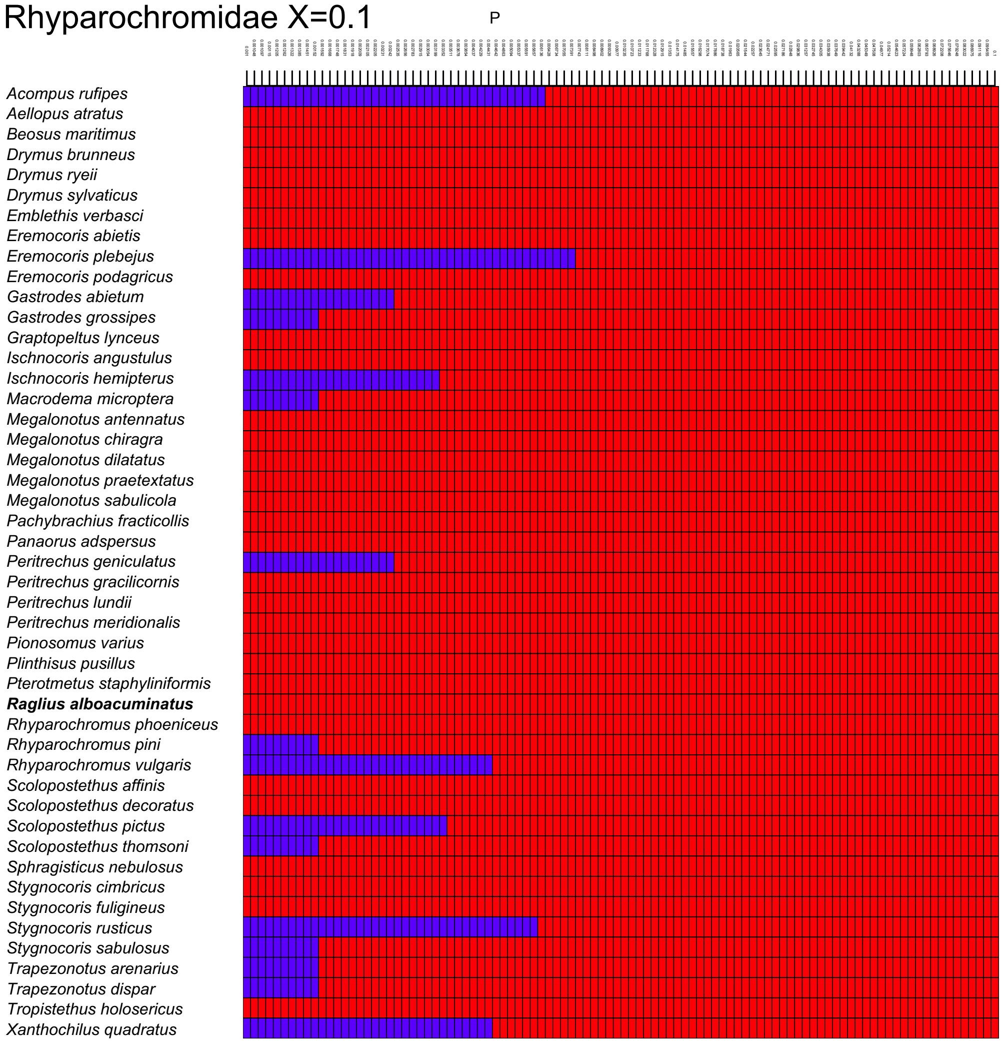

Rhyparochromidae X=1.1

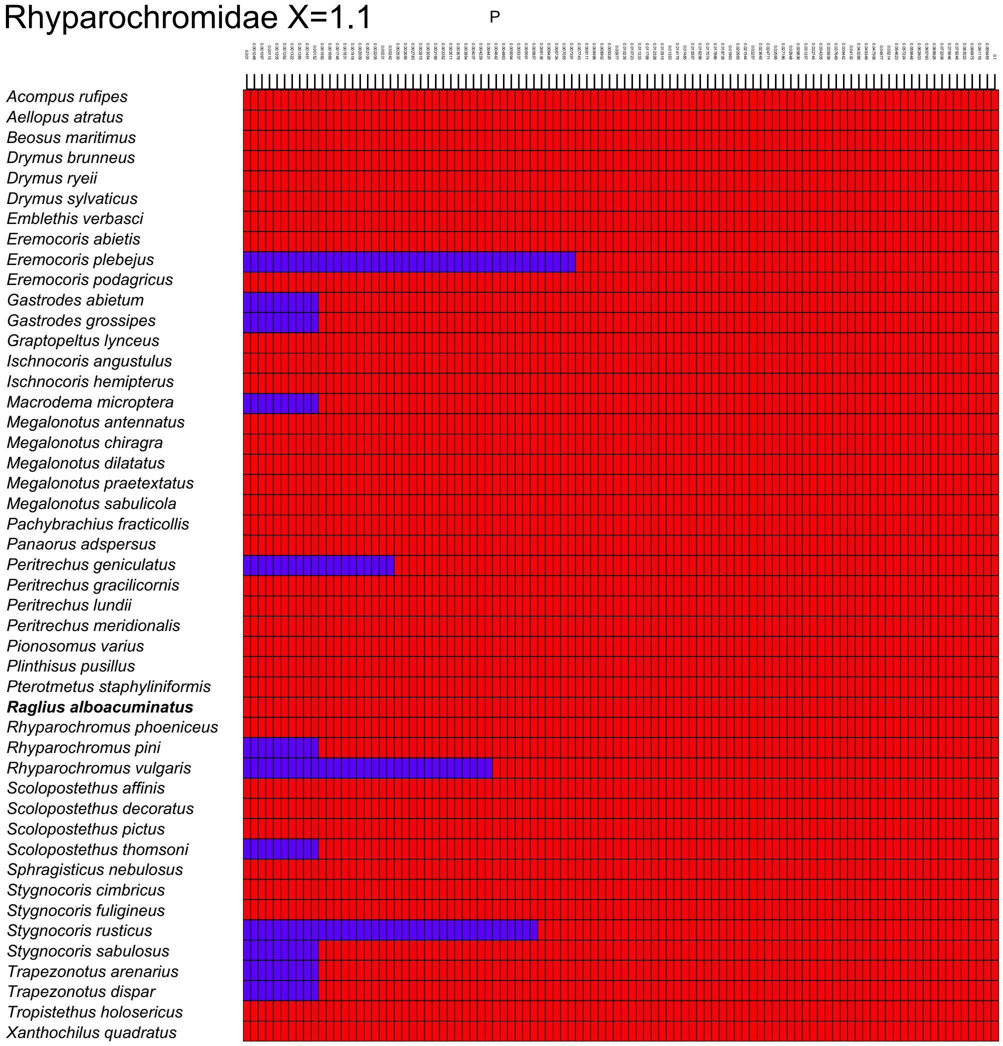

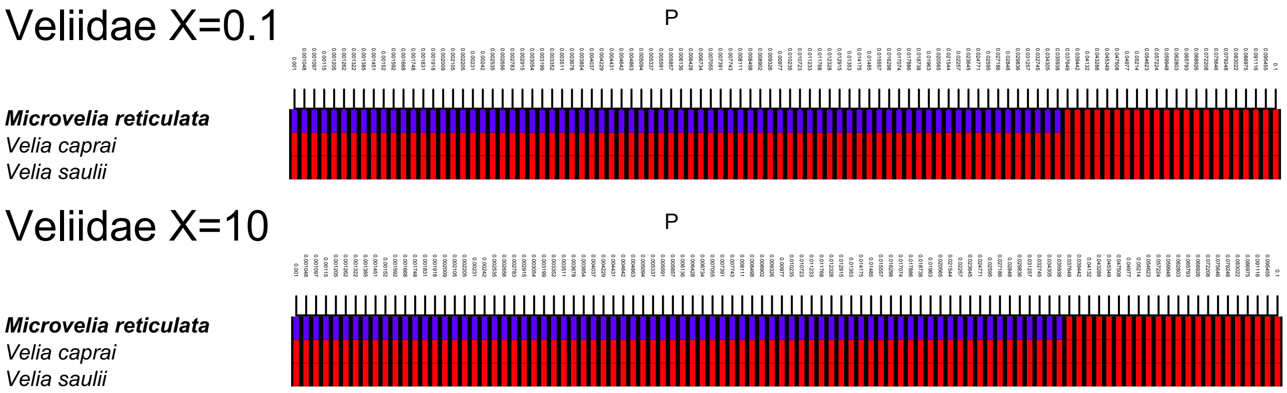
Veliidae X=0.1
P
Microvelia reticulata
Velia caprai
Velia saulii
Veliidae X=10
P
Microvelia reticulata
Velia caprai
Velia saulii
