# Supplementary figures and images for: Building-Up of a DNA Barcode Library for True Bugs (Insecta: Hemiptera: Heteroptera) of Germany Reveals Taxonomic Uncertainties and Surprises
Source: PLoS One. 2014 Sep 9;9(9):e106940. doi: 10.1371/journal.pone.0106940 (PMC4159288; doi:10.1371/journal.pone.0106940)

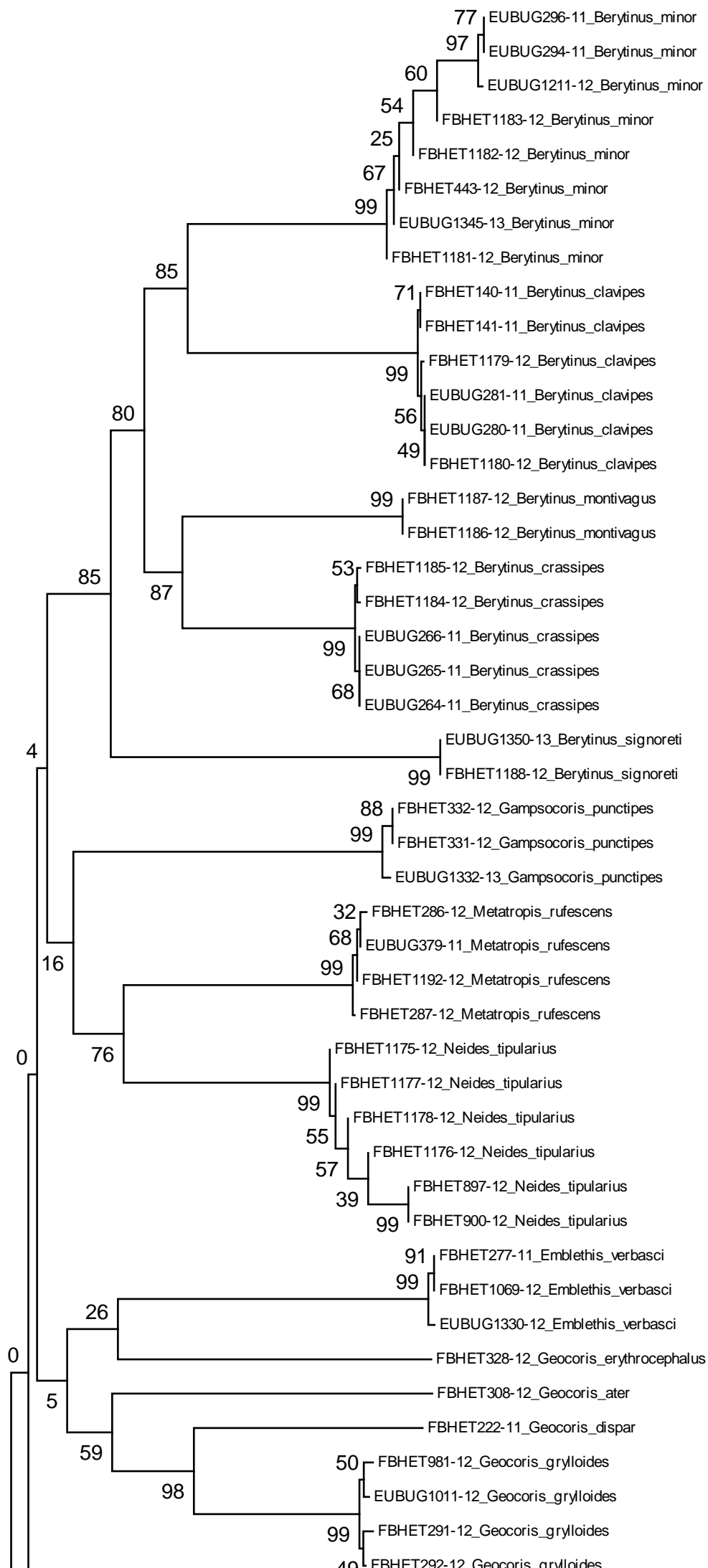

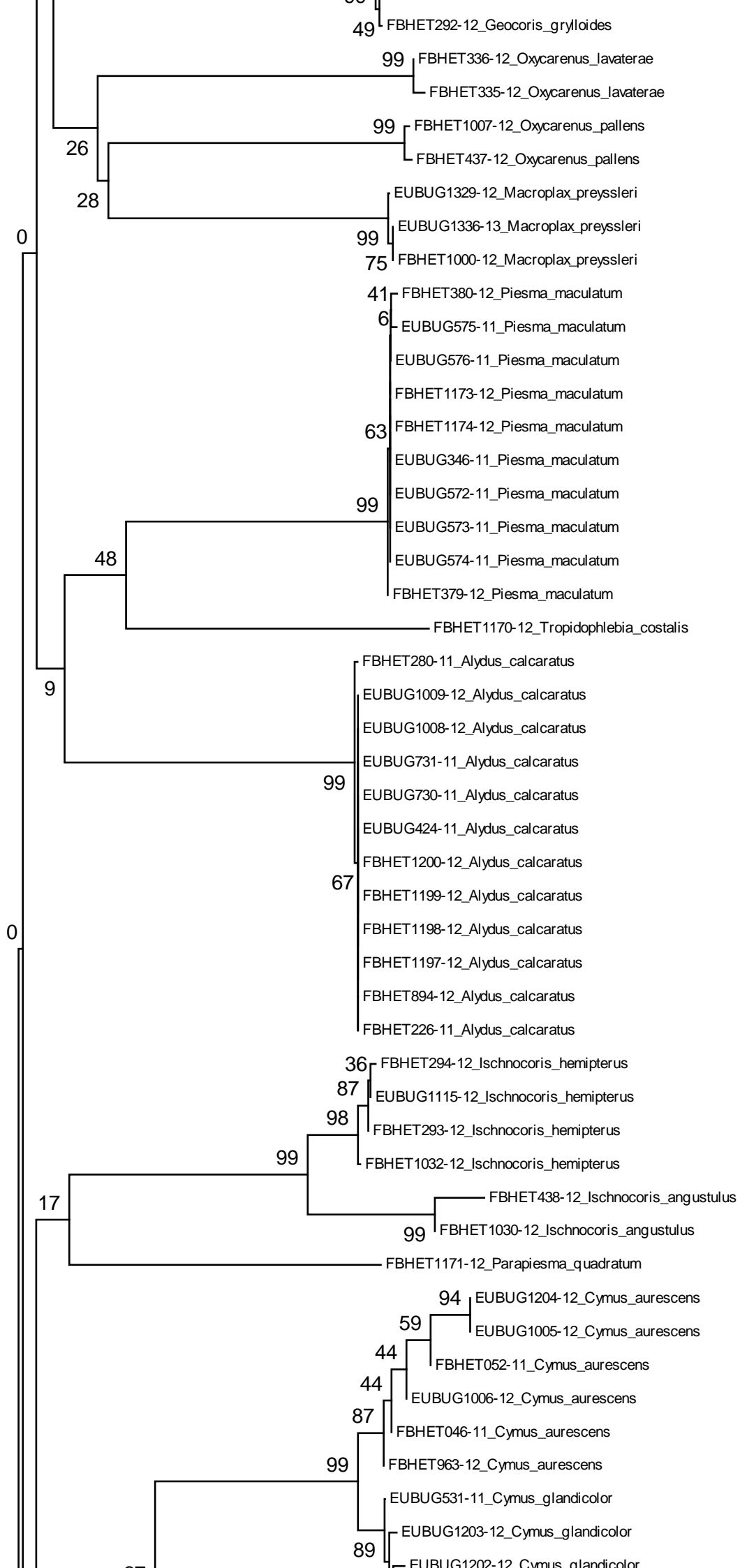

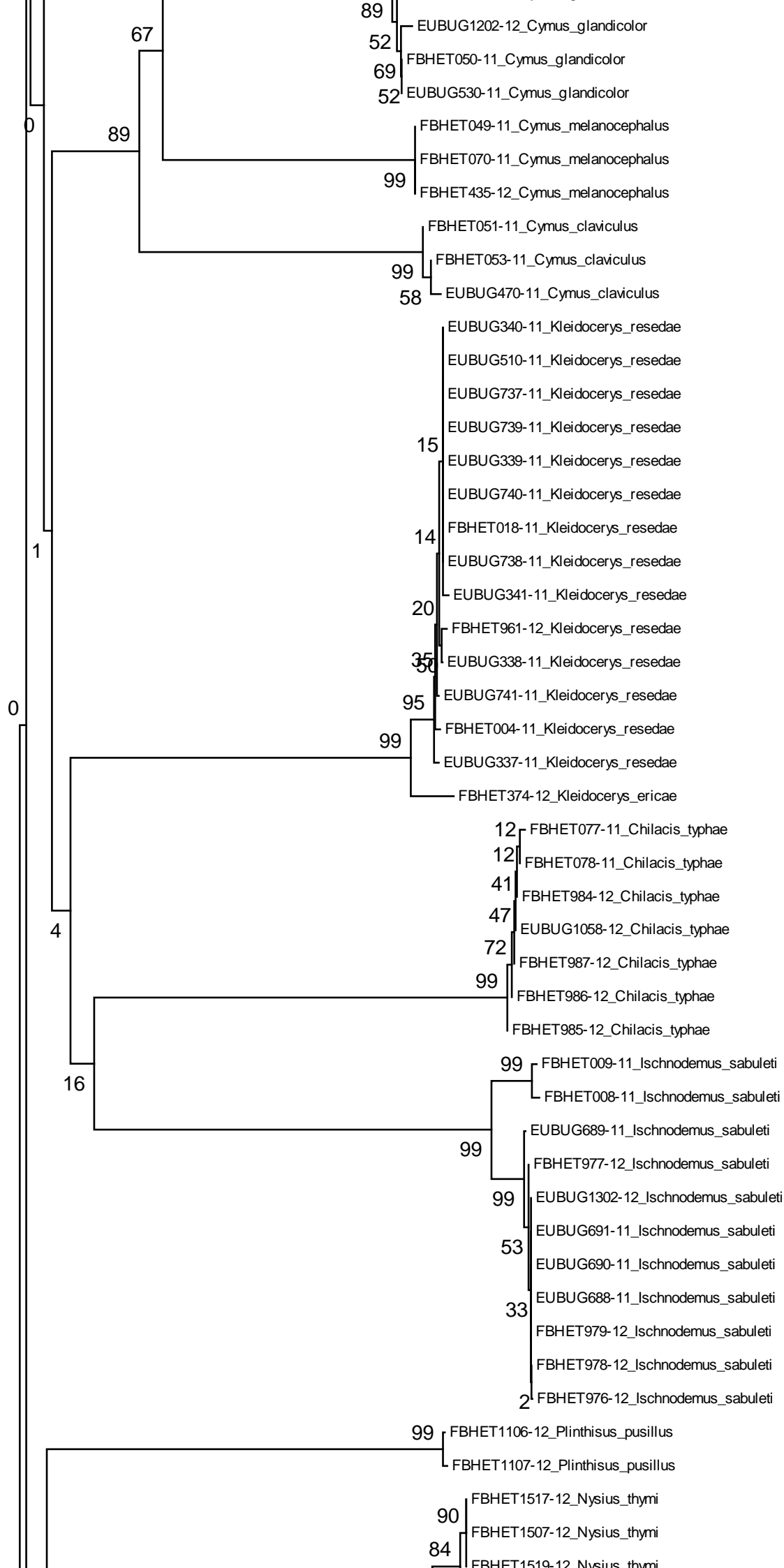

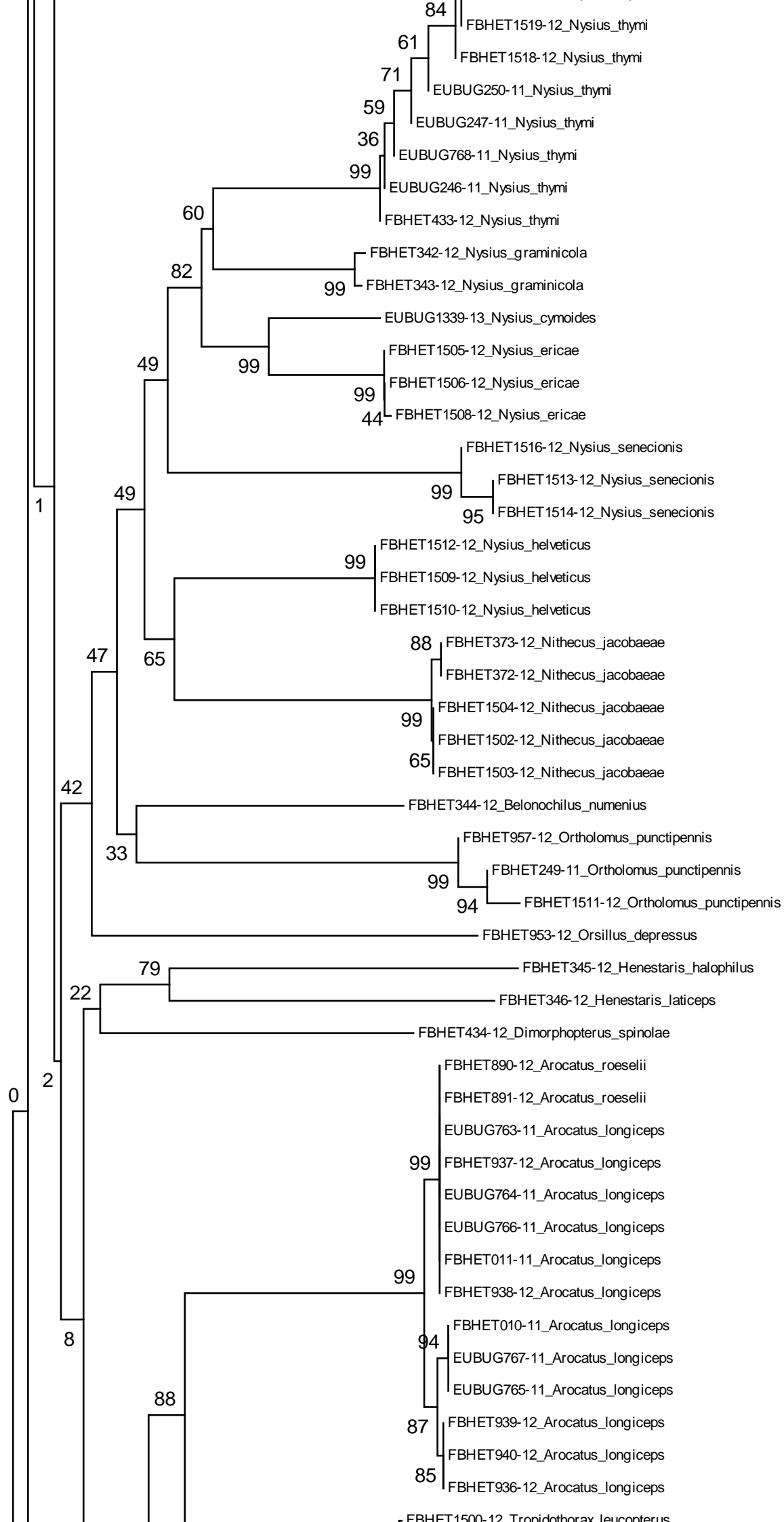

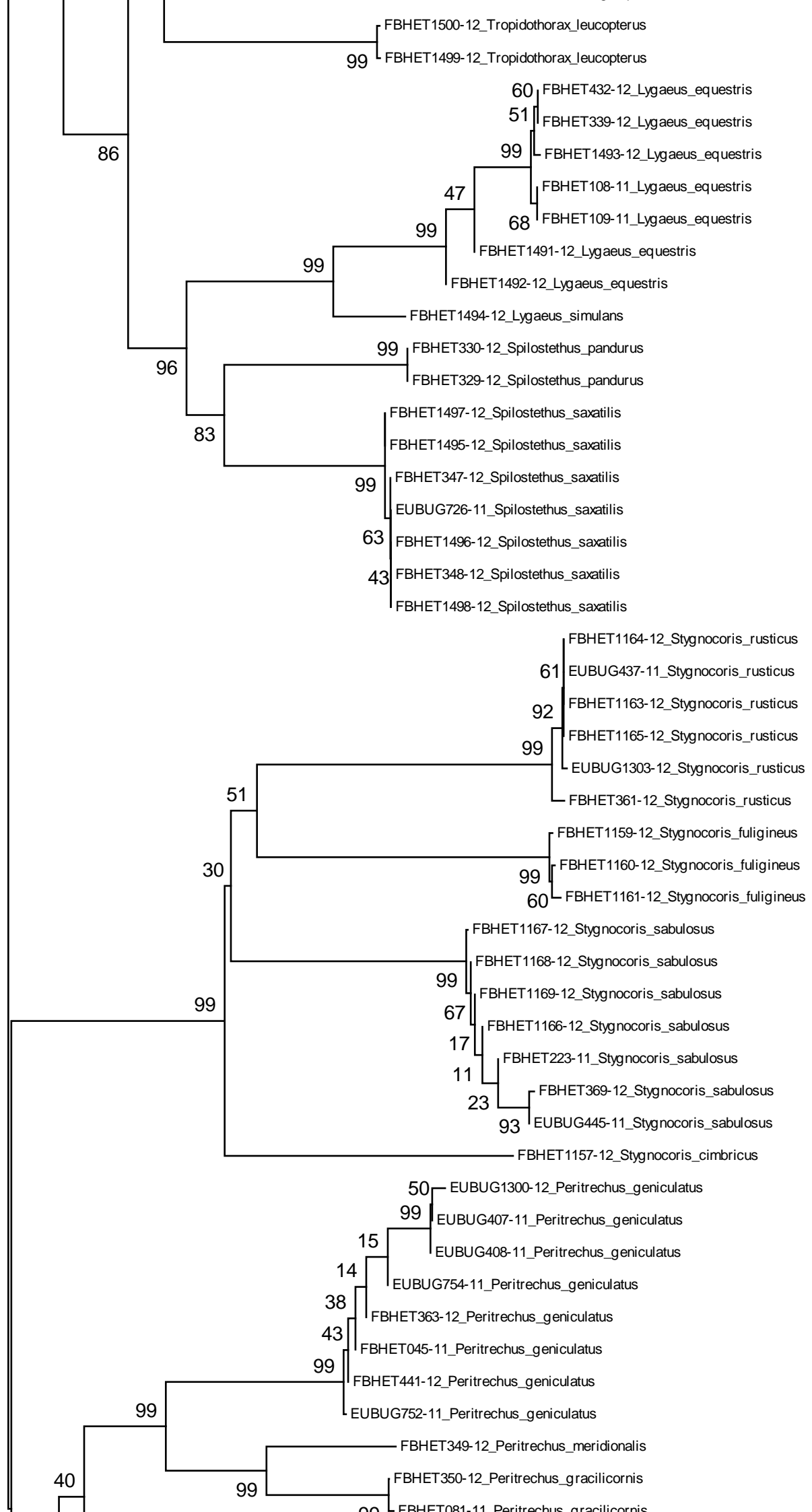

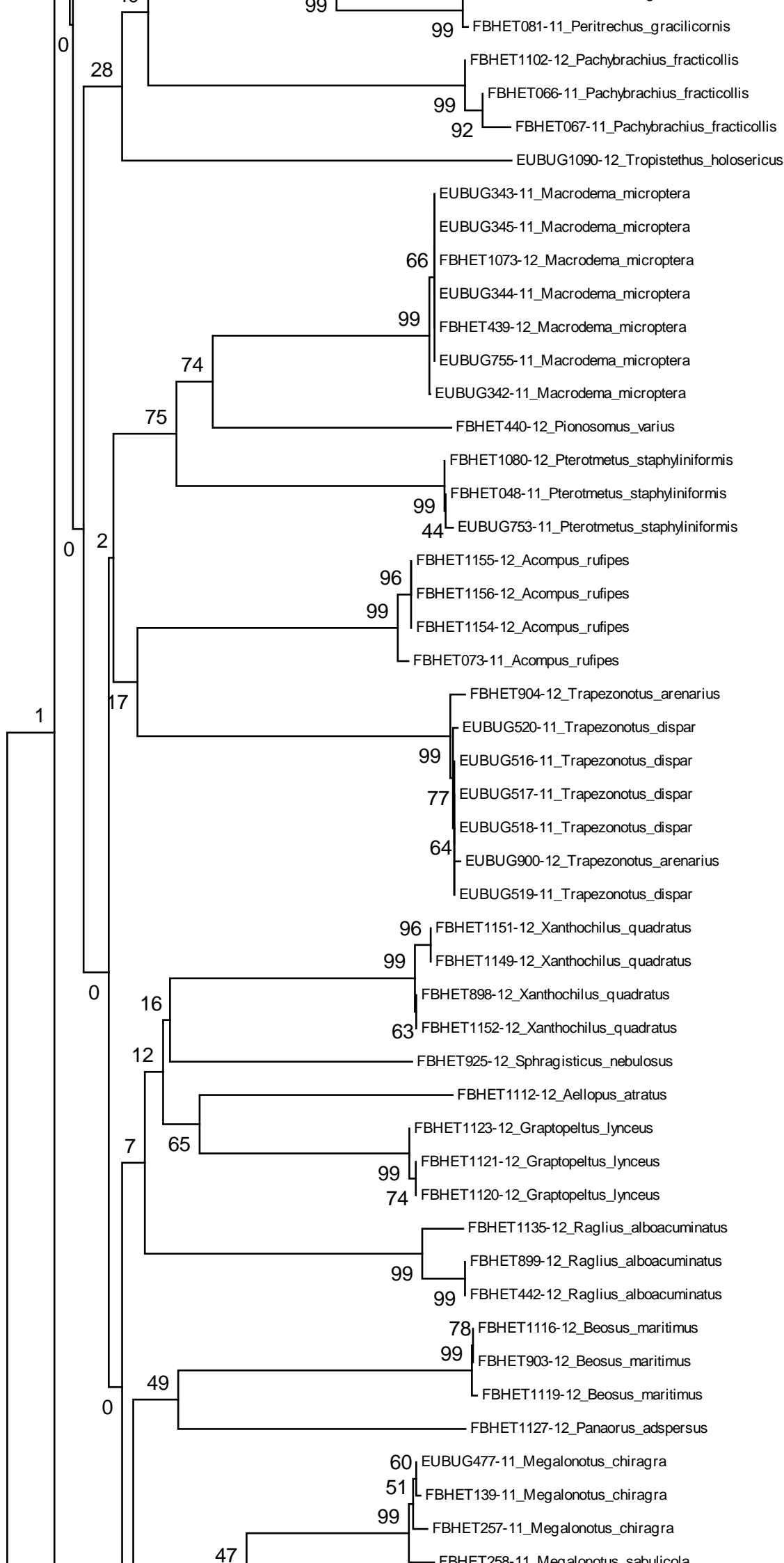

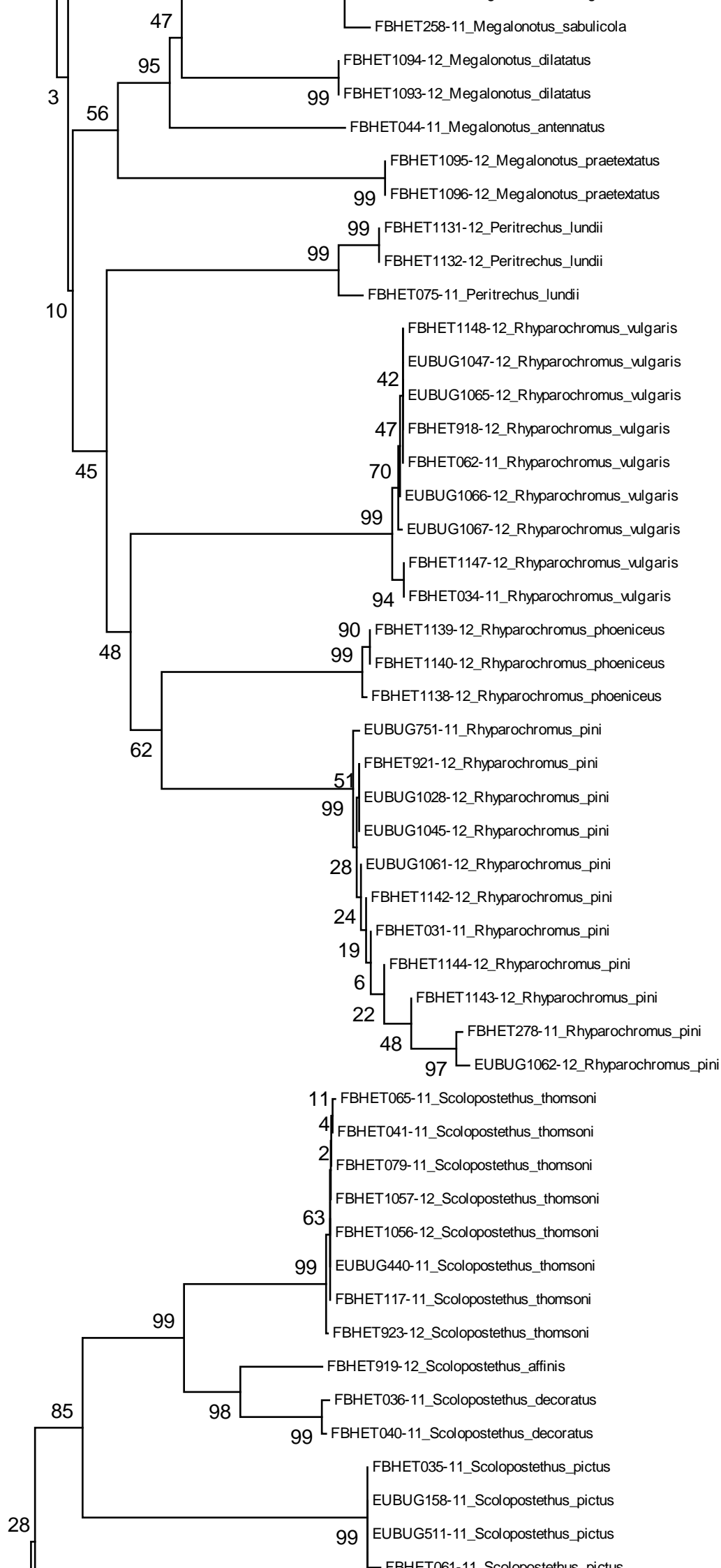

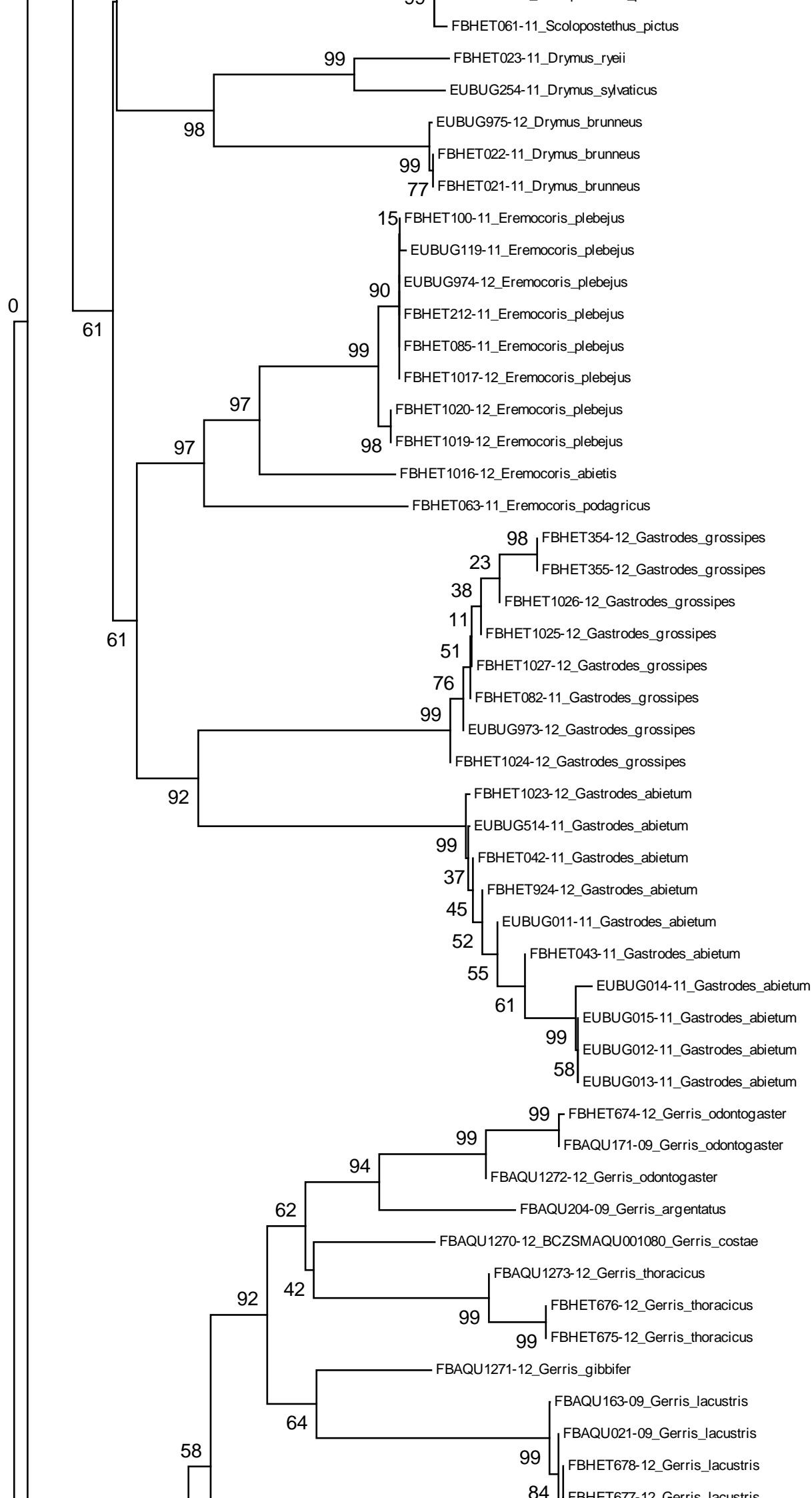

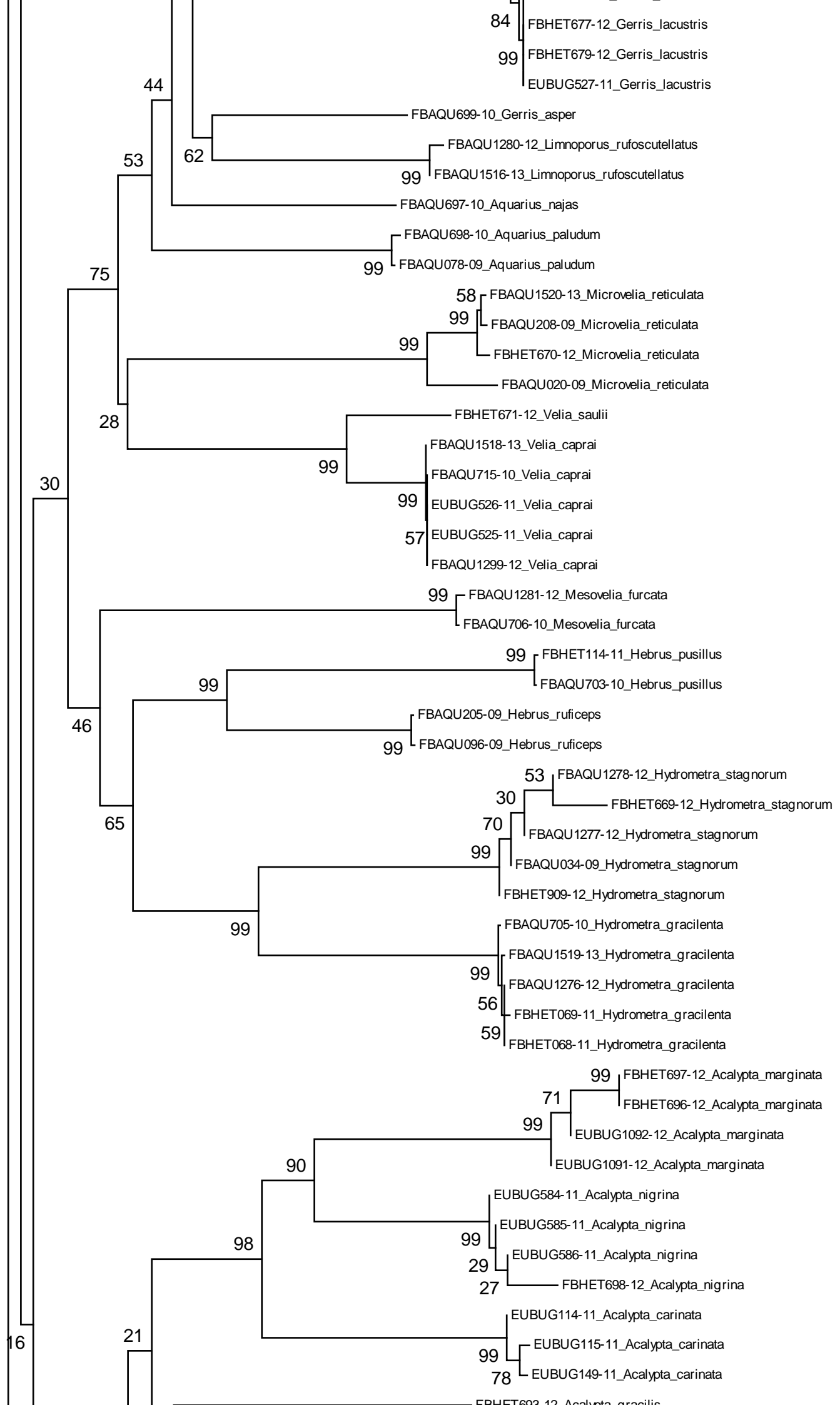

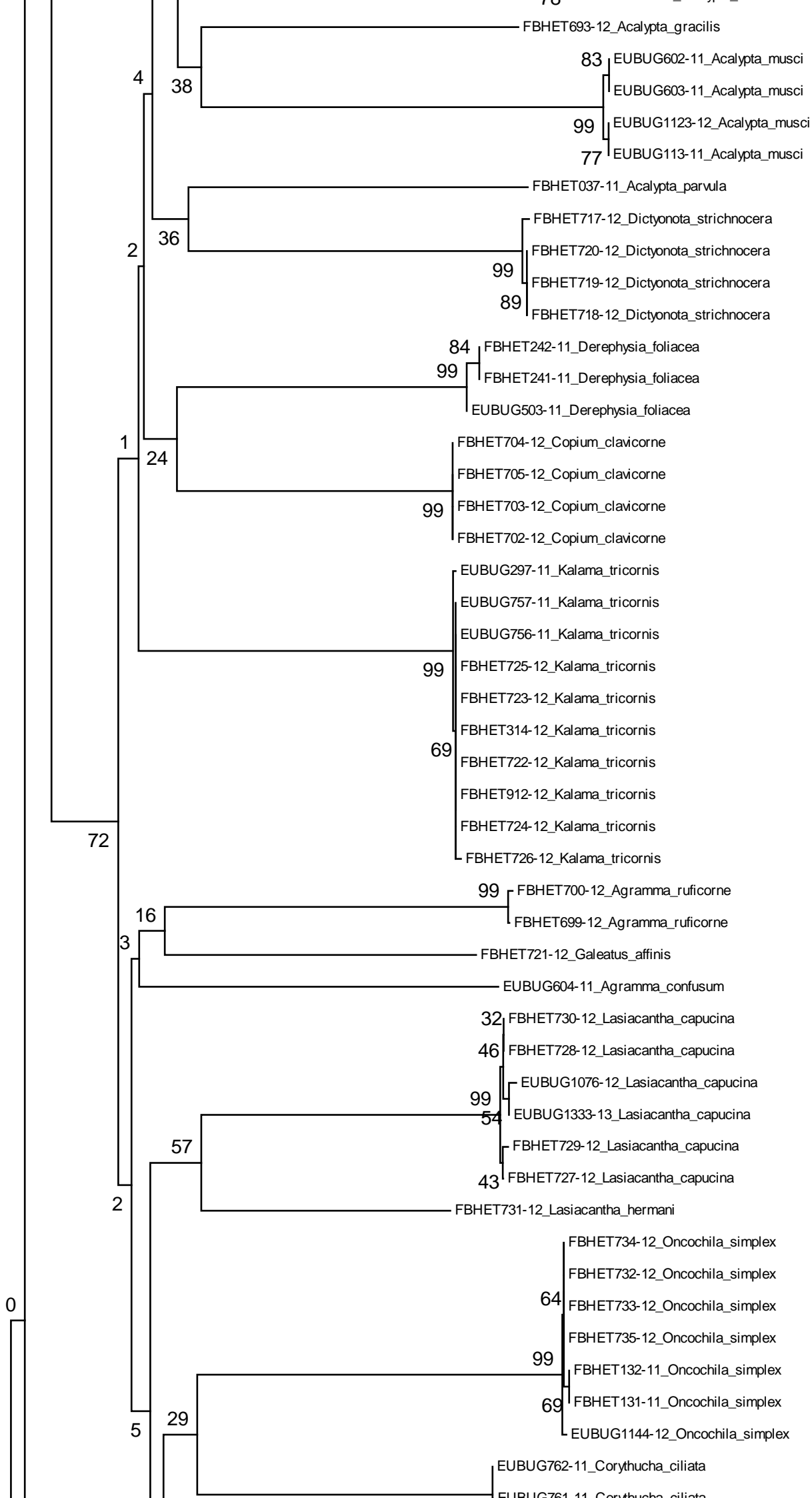

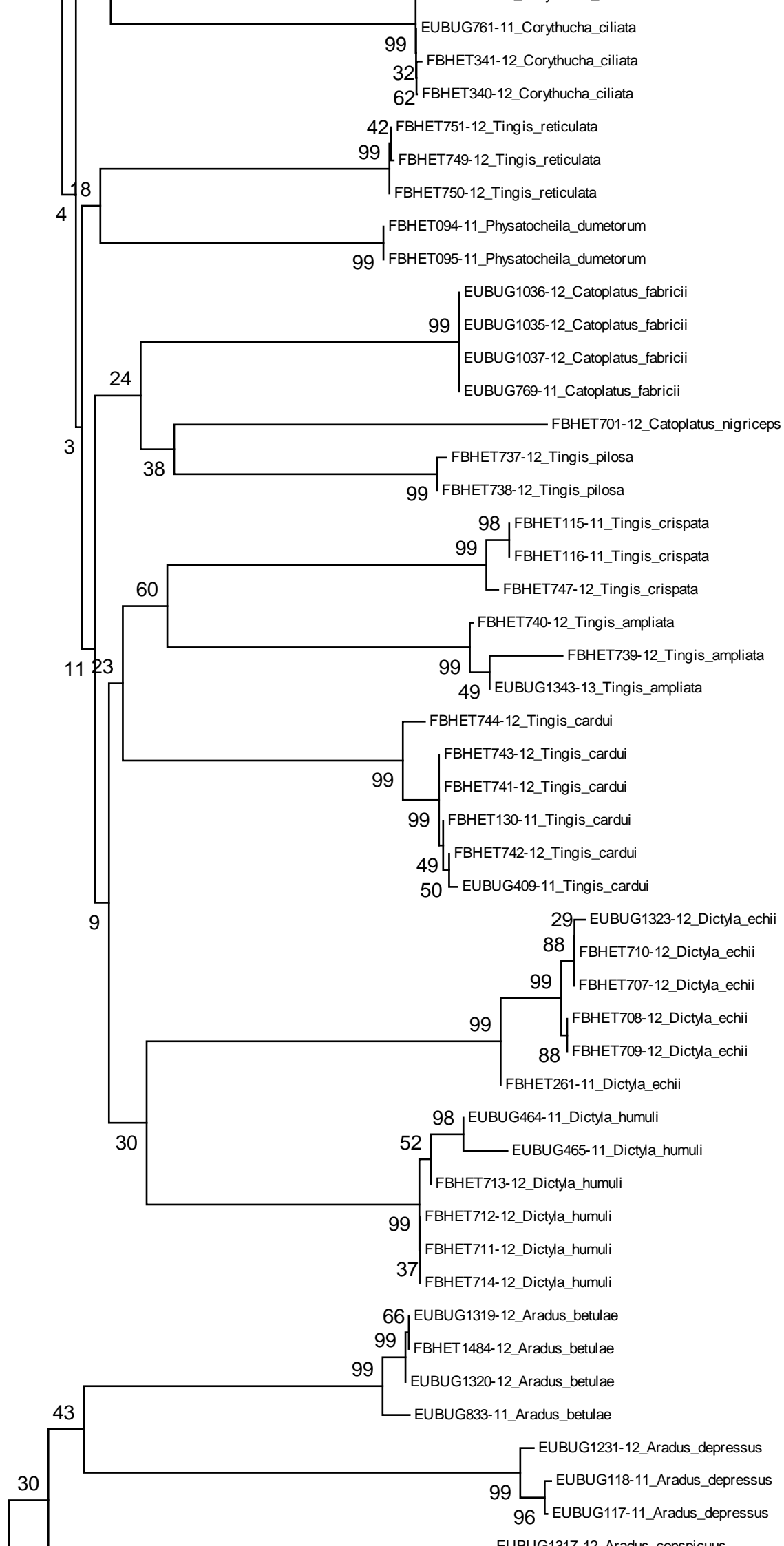

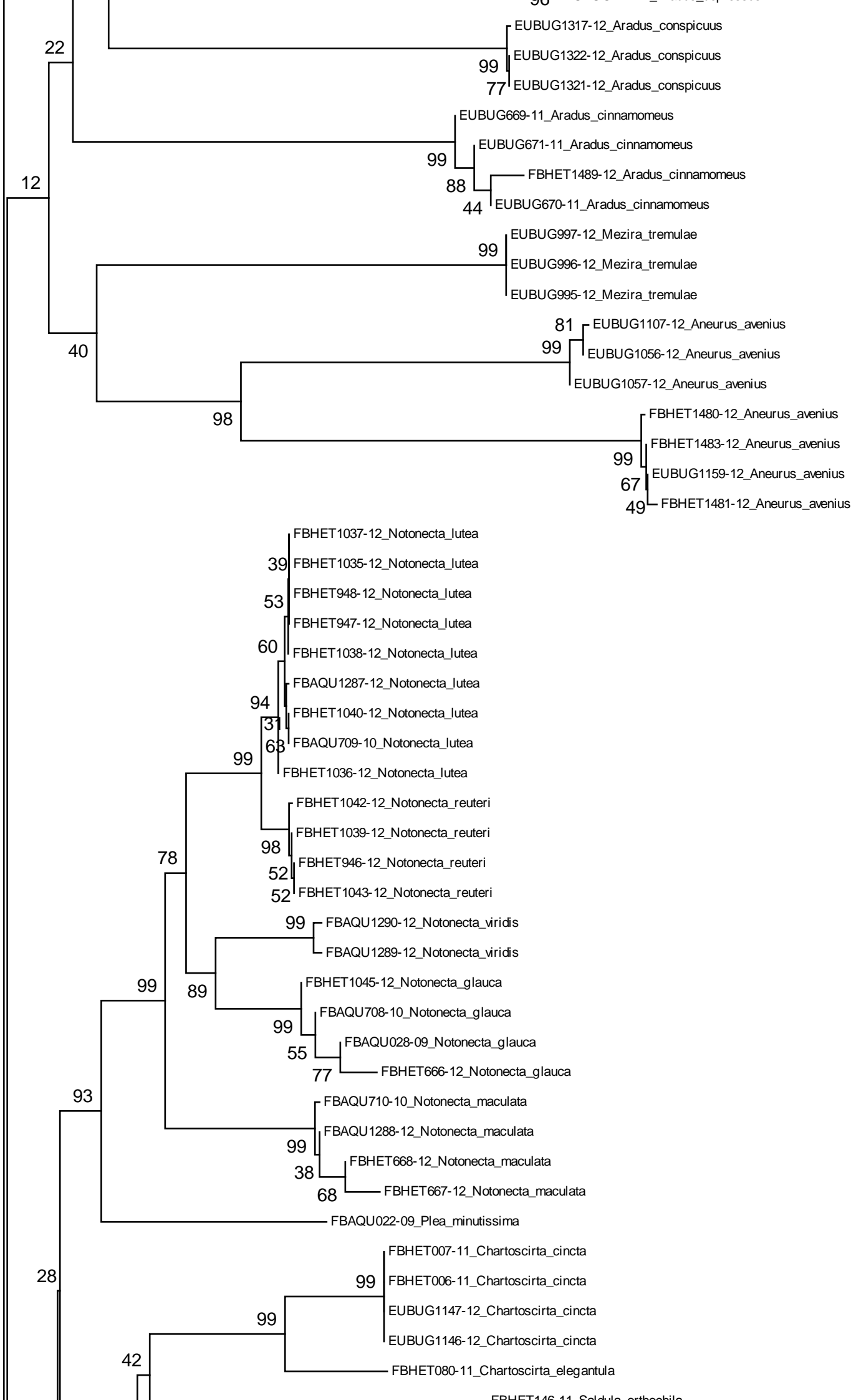

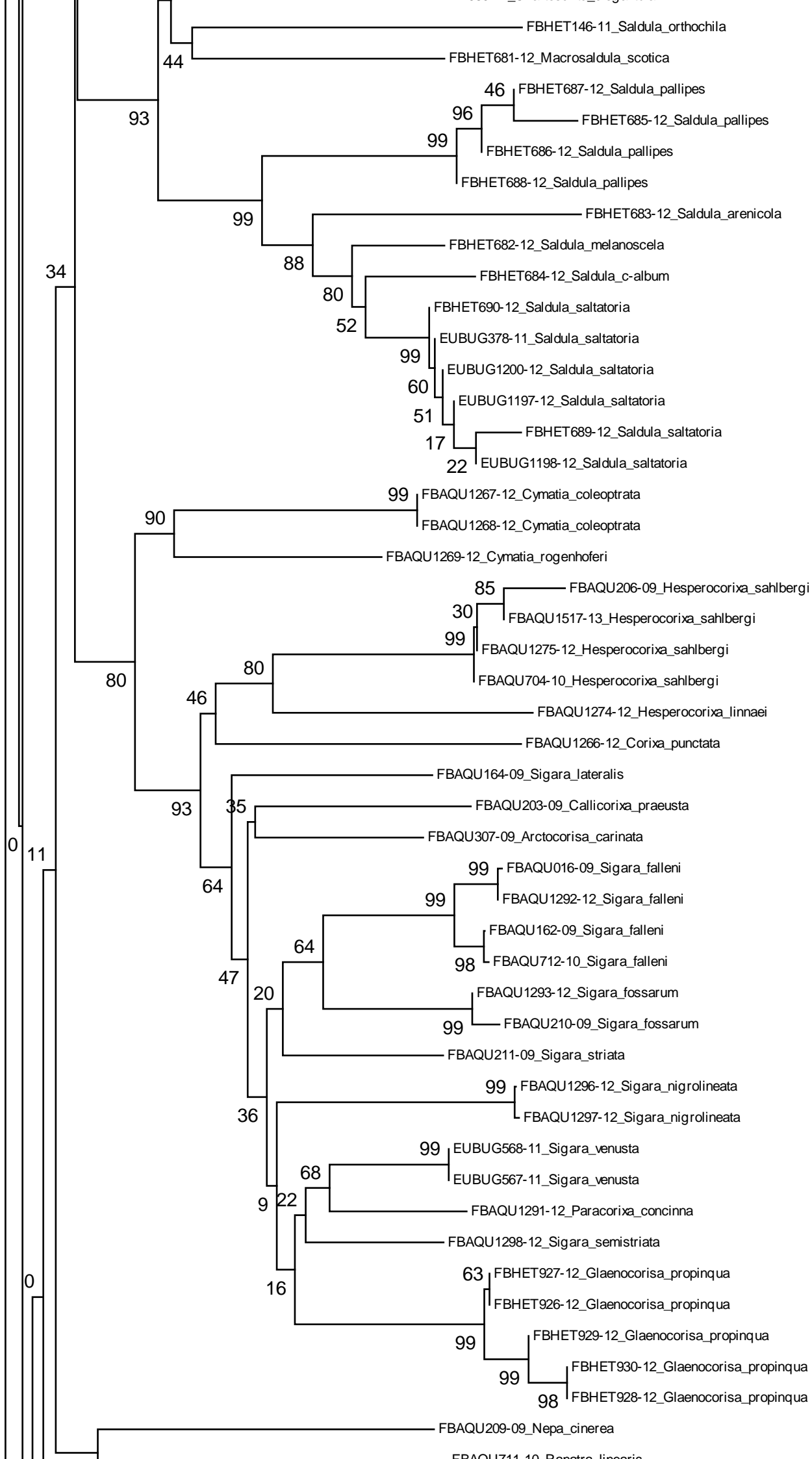

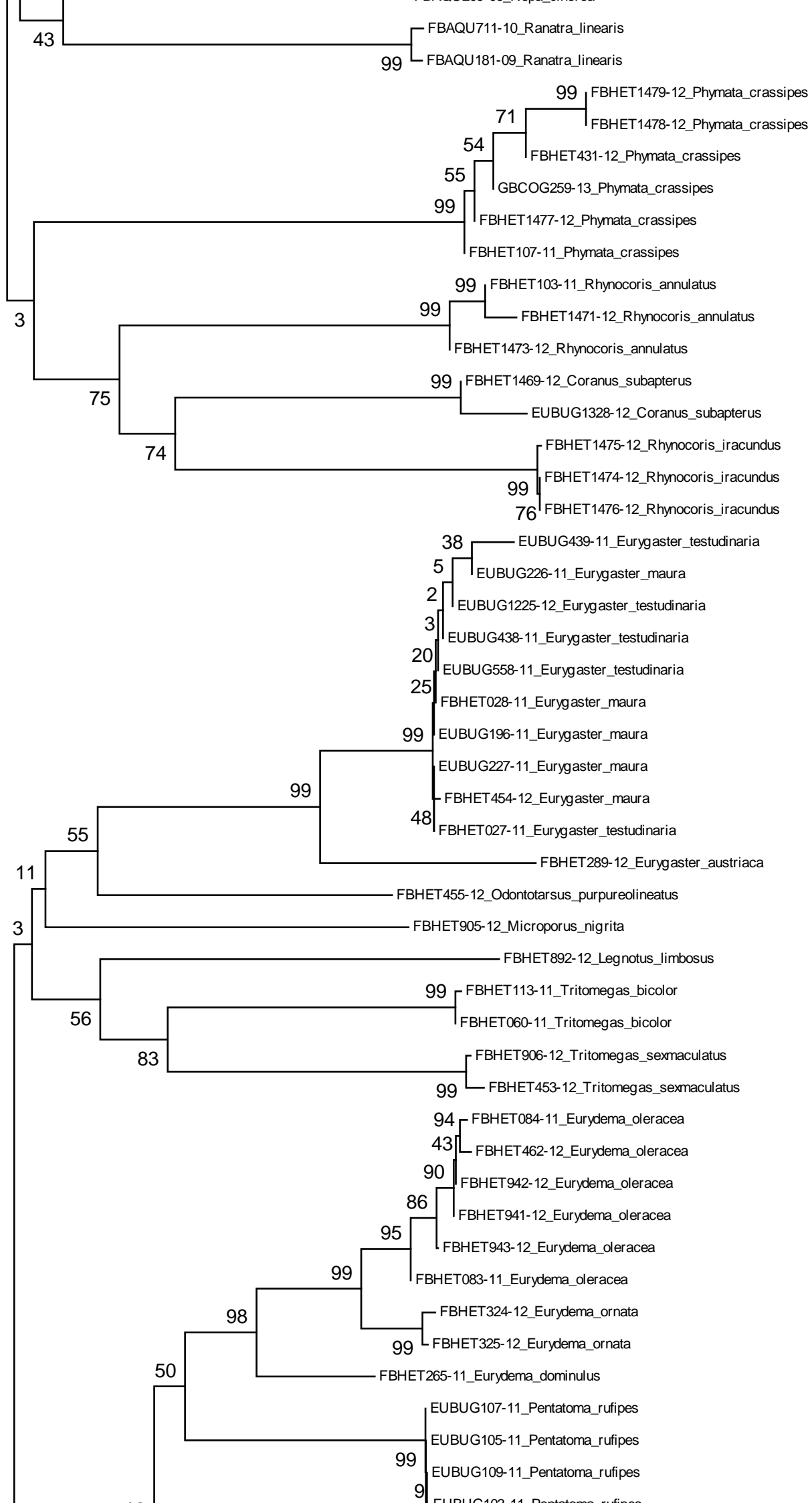

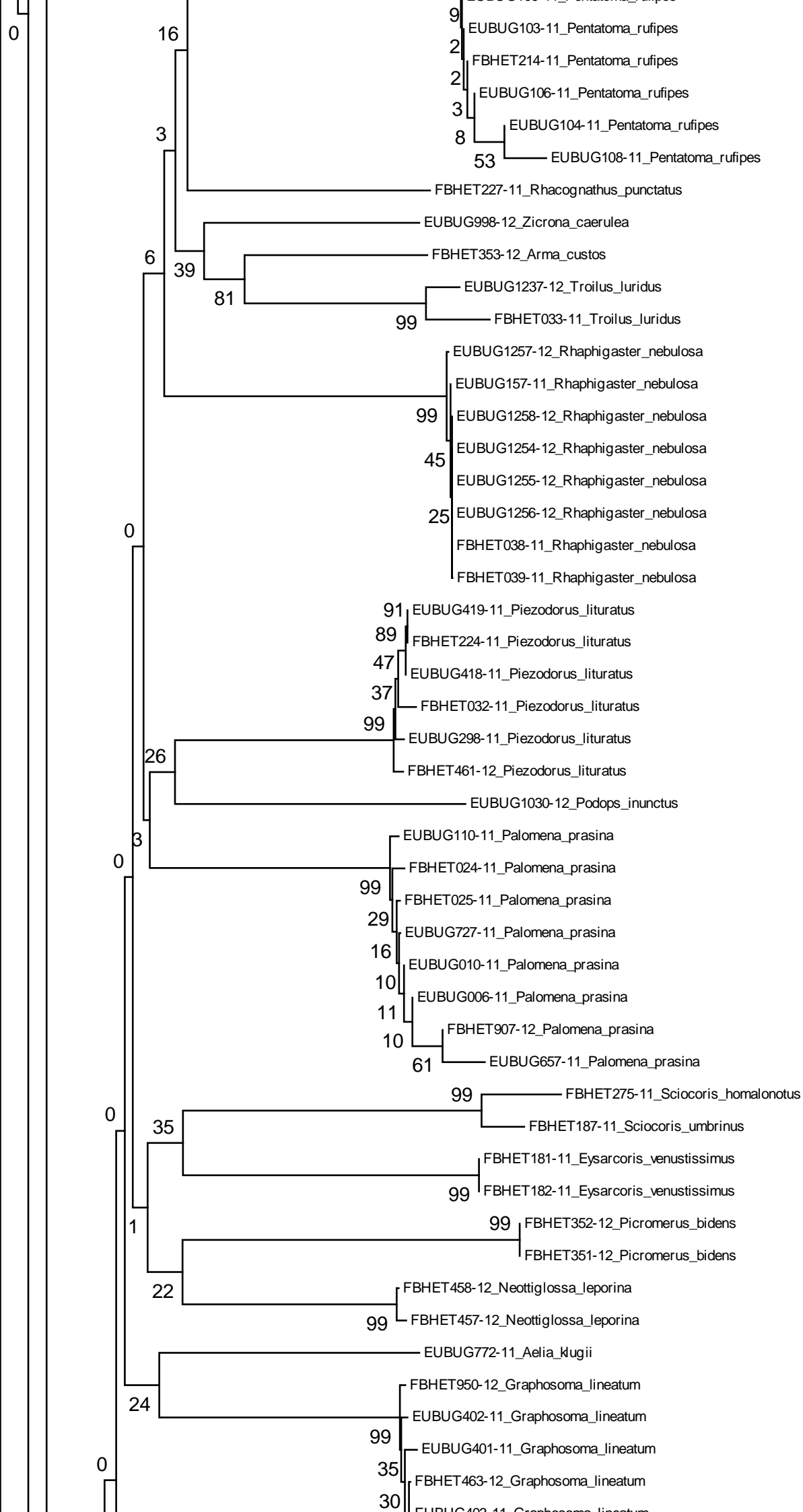

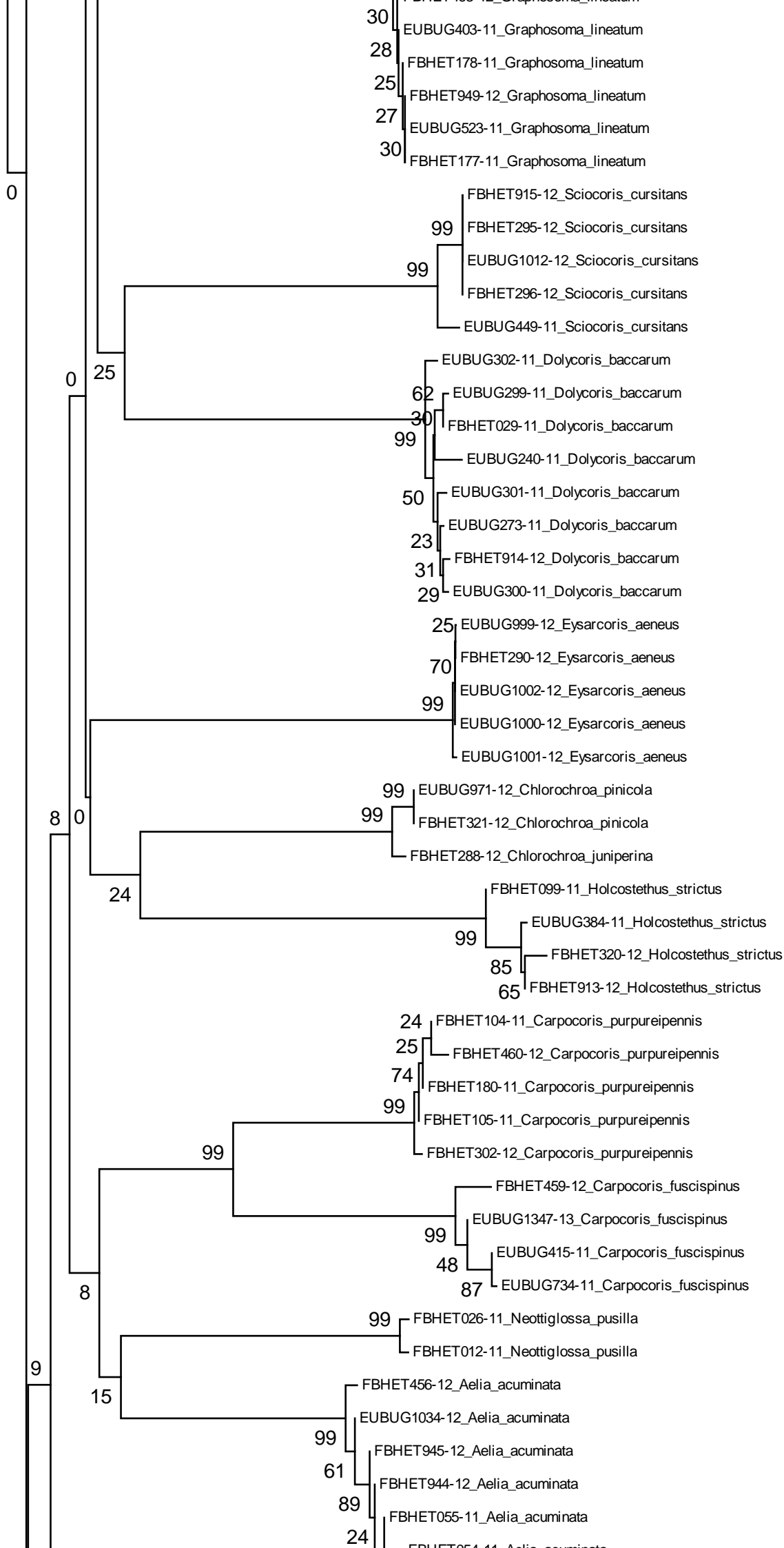

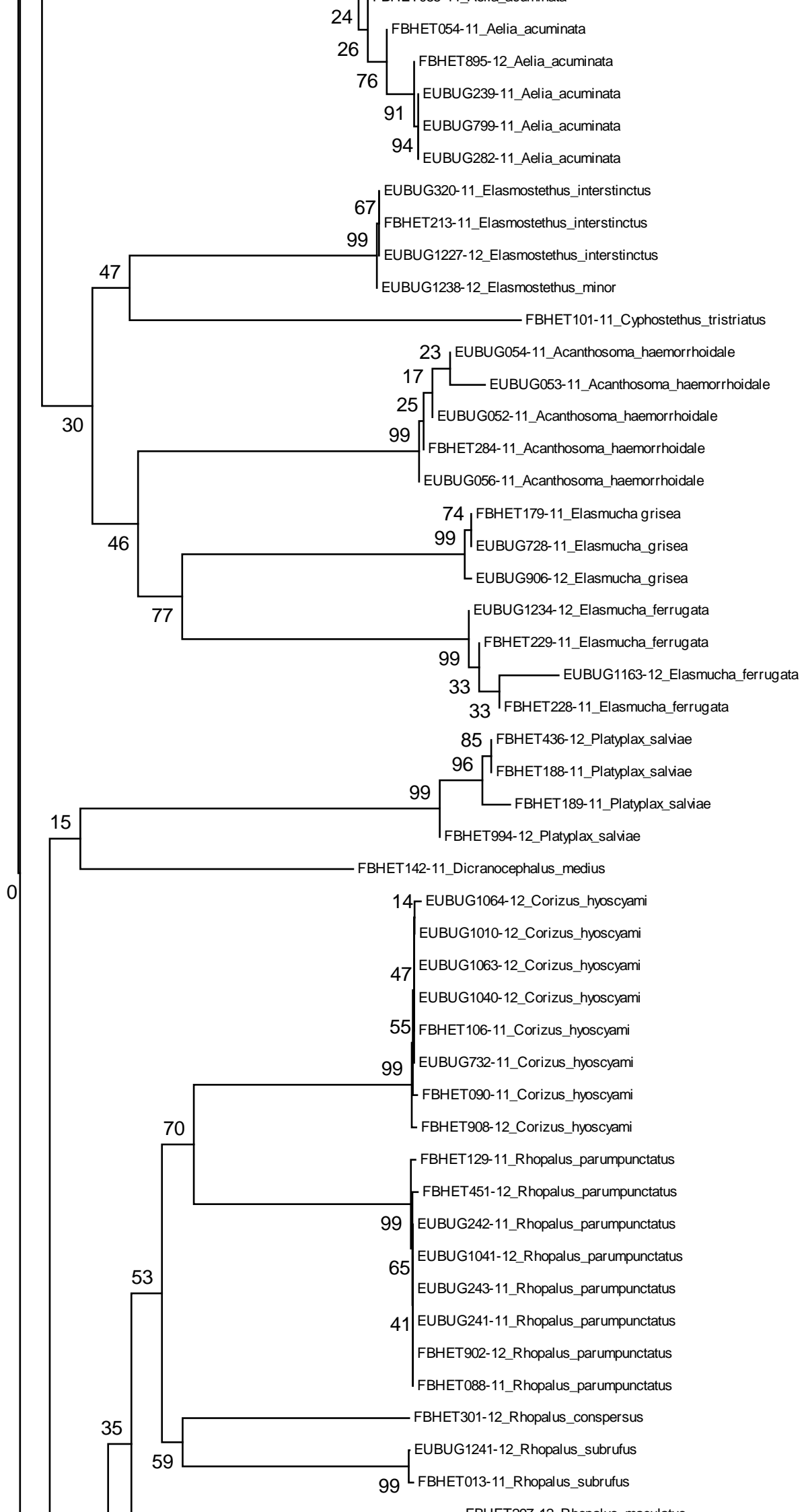

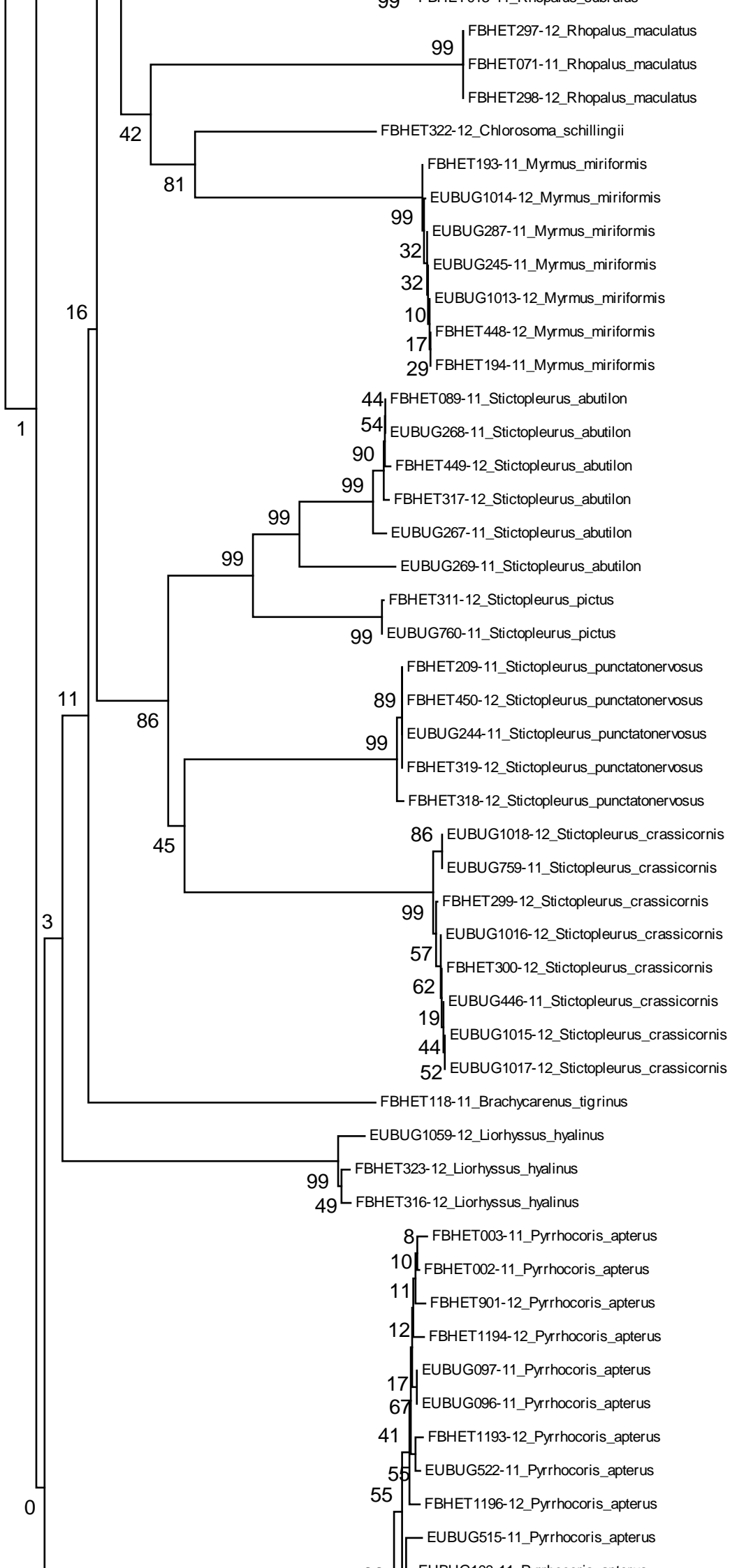

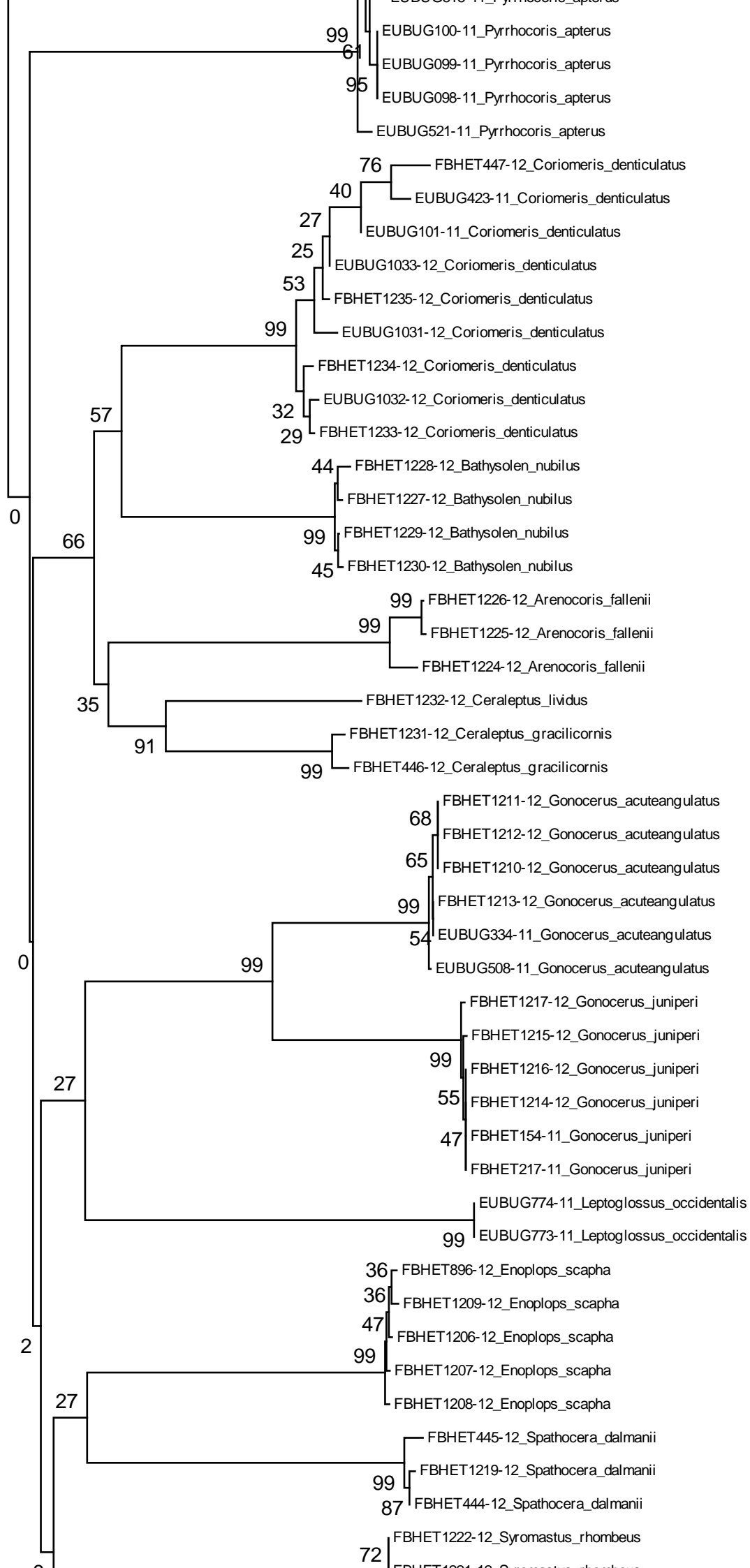

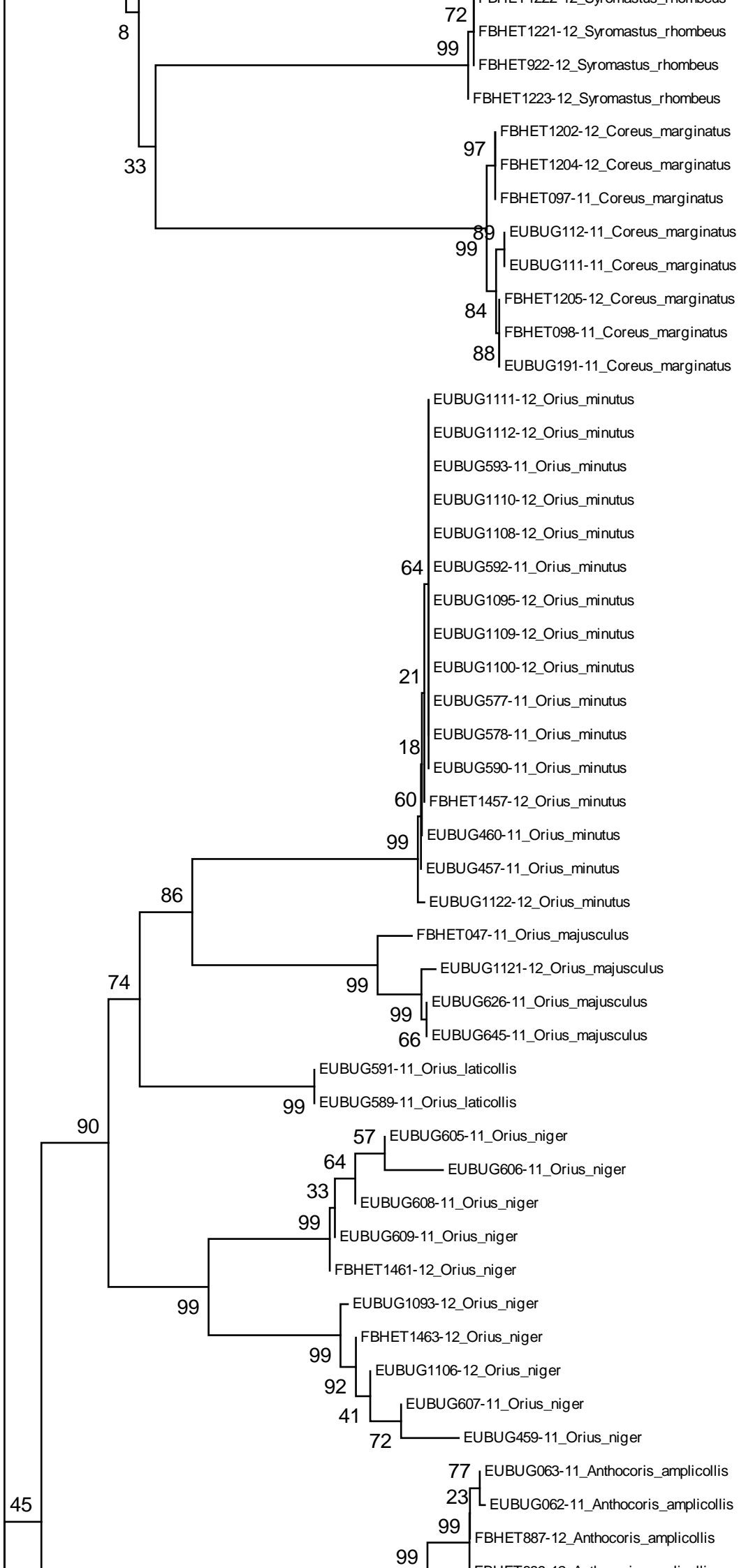

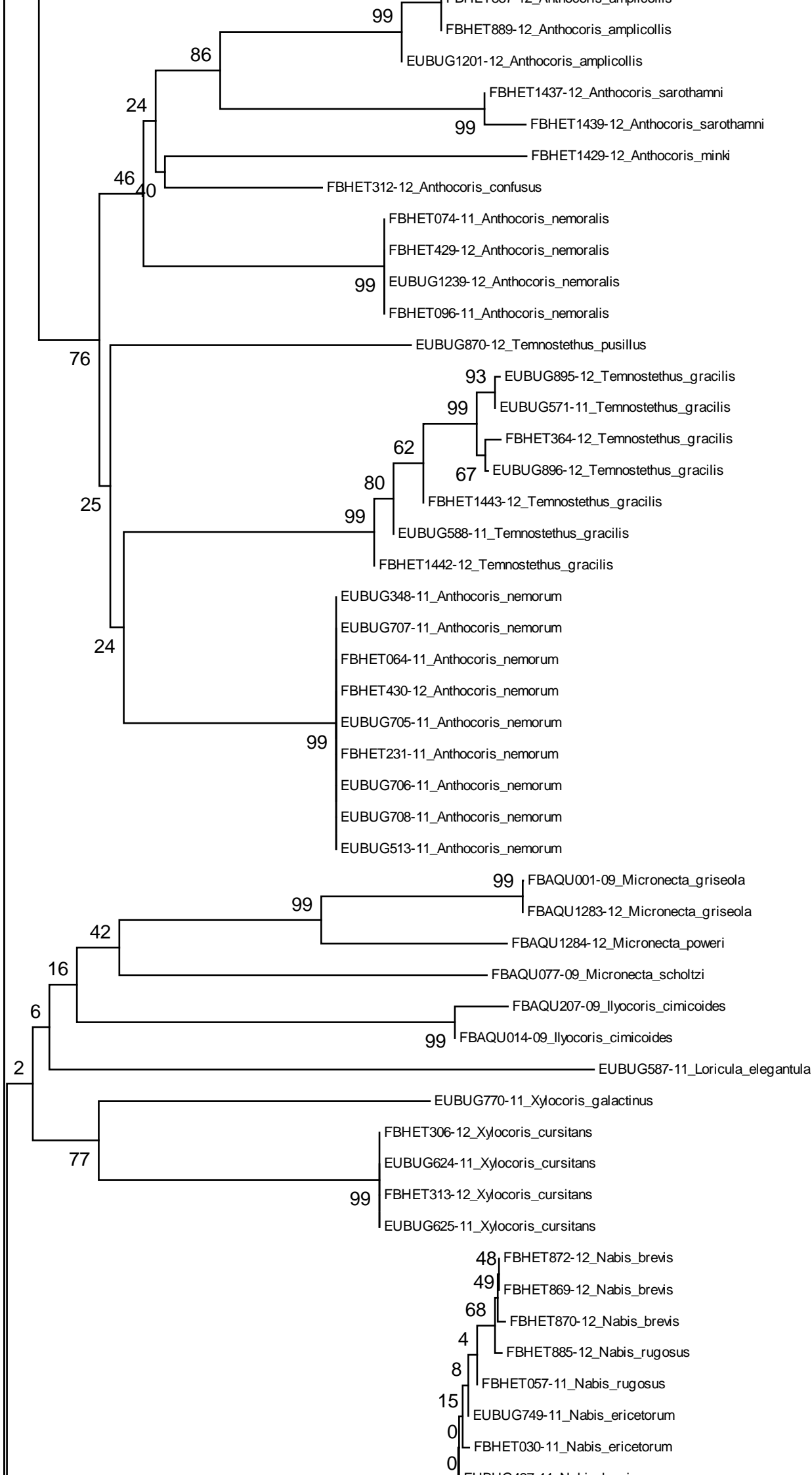

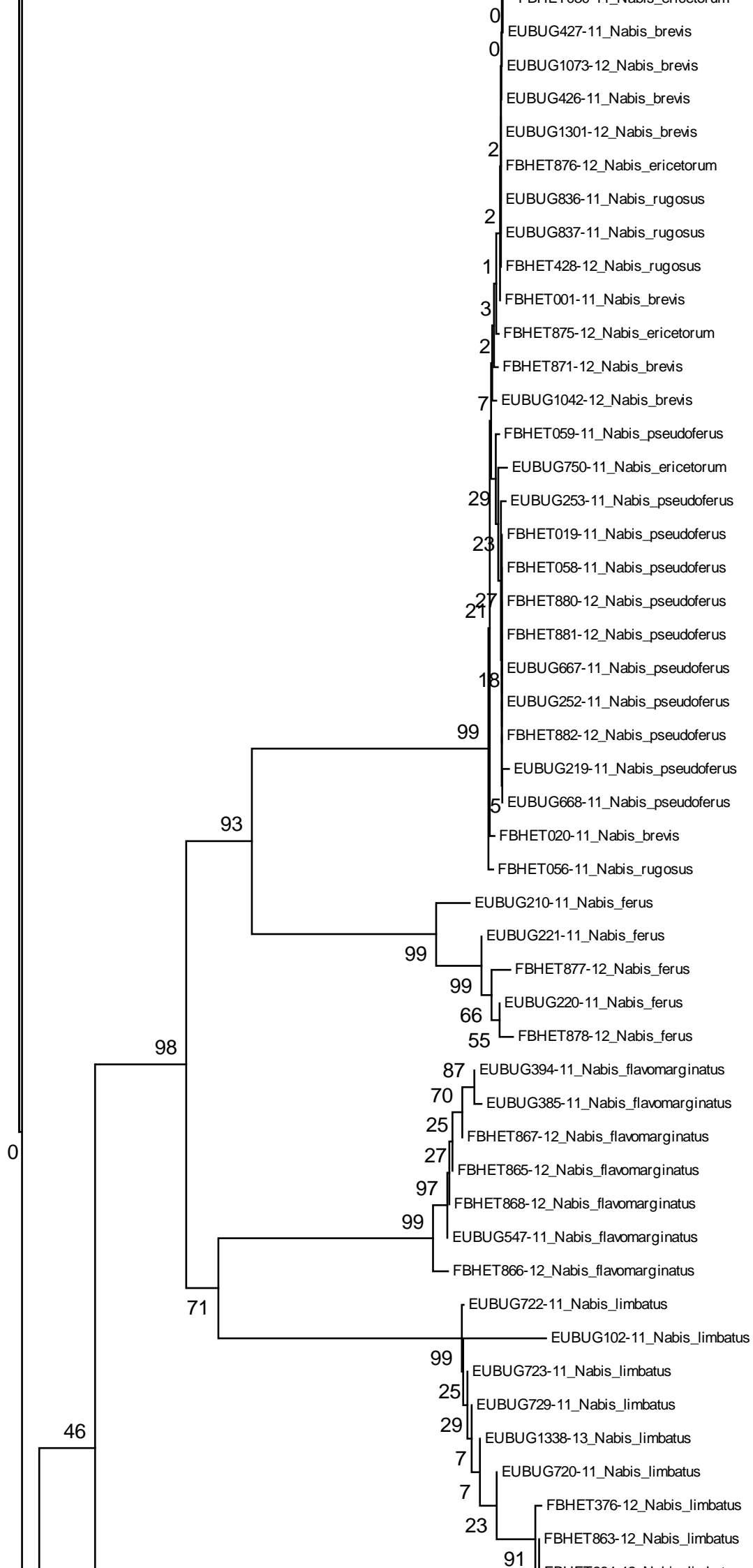

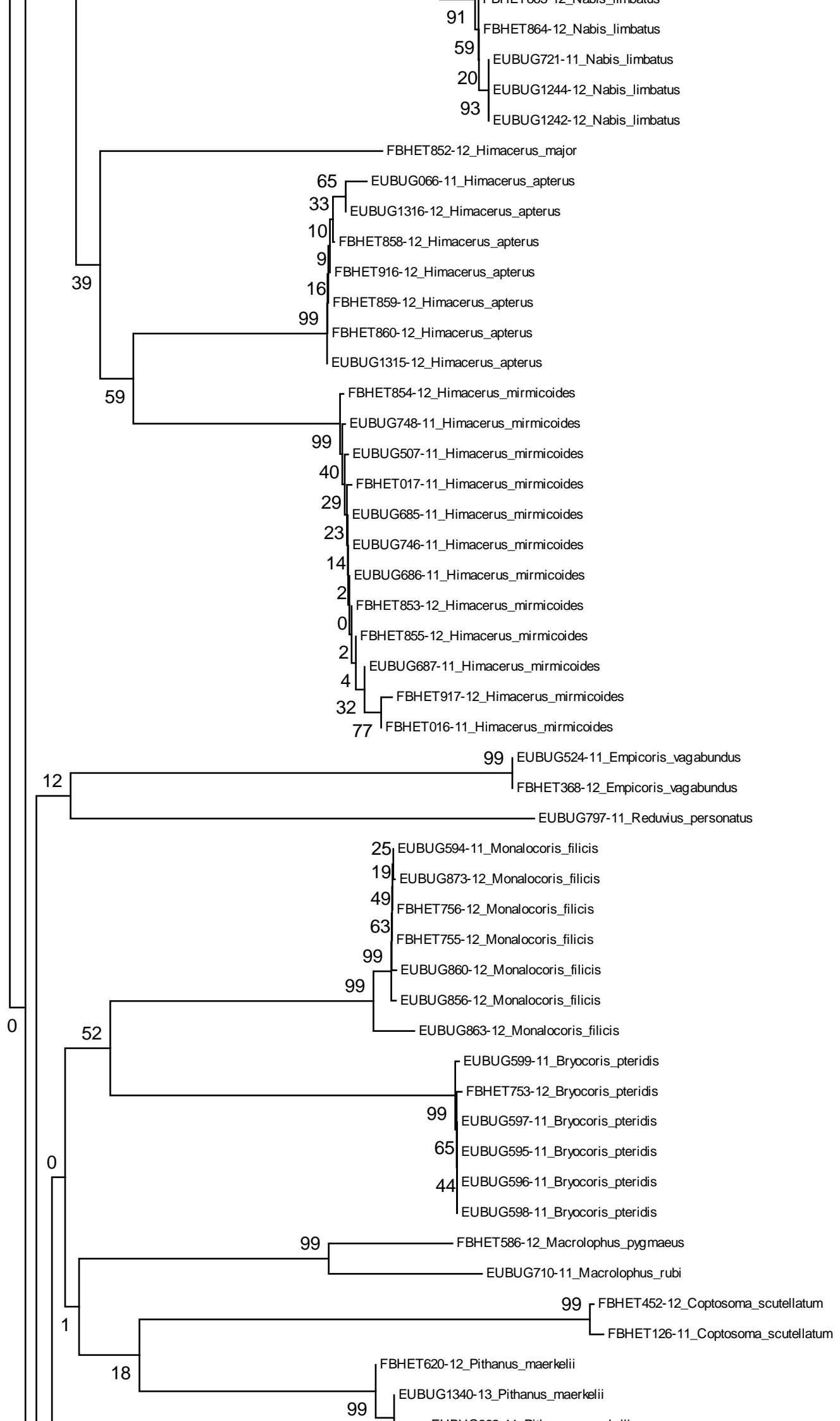

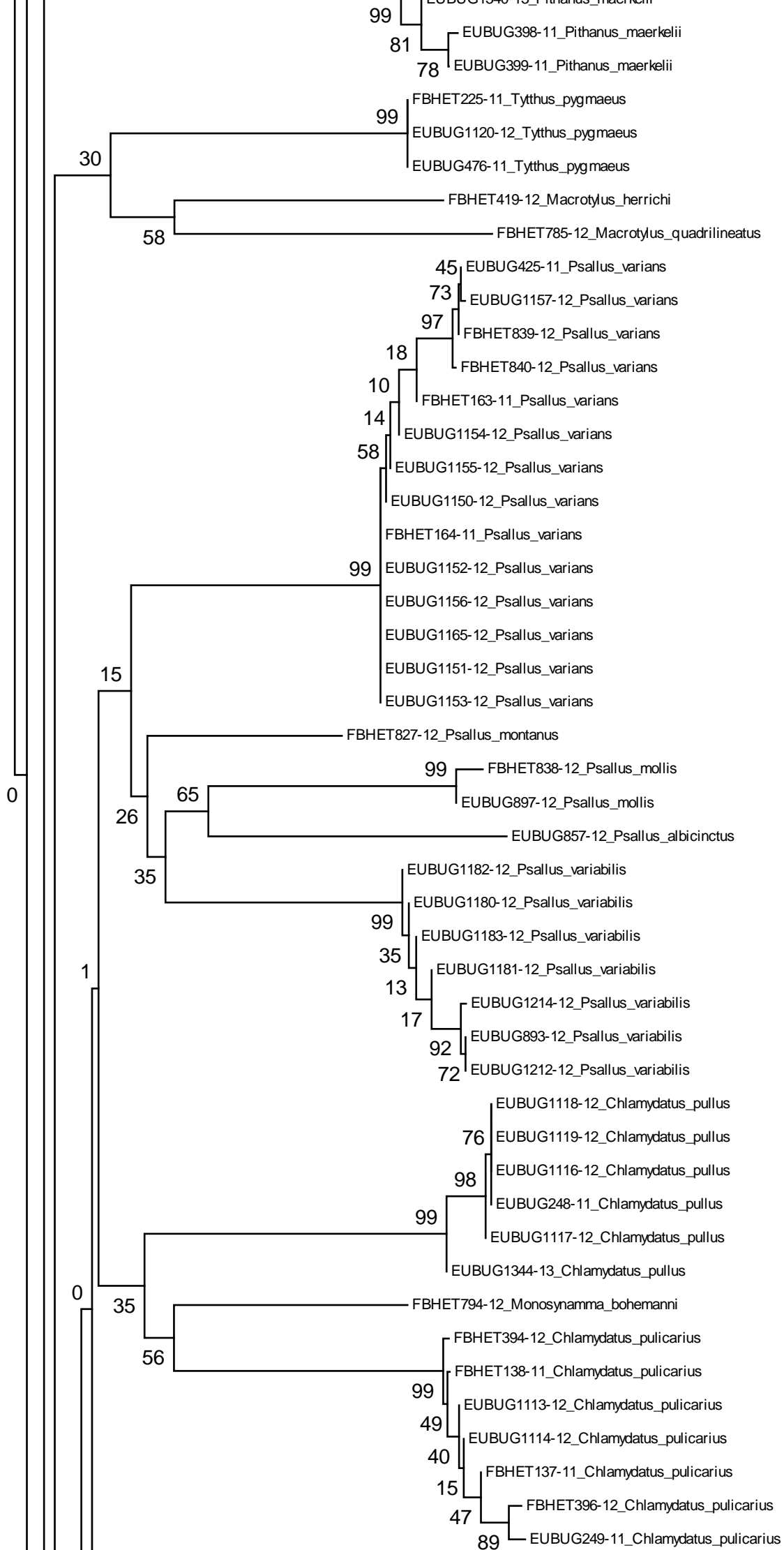

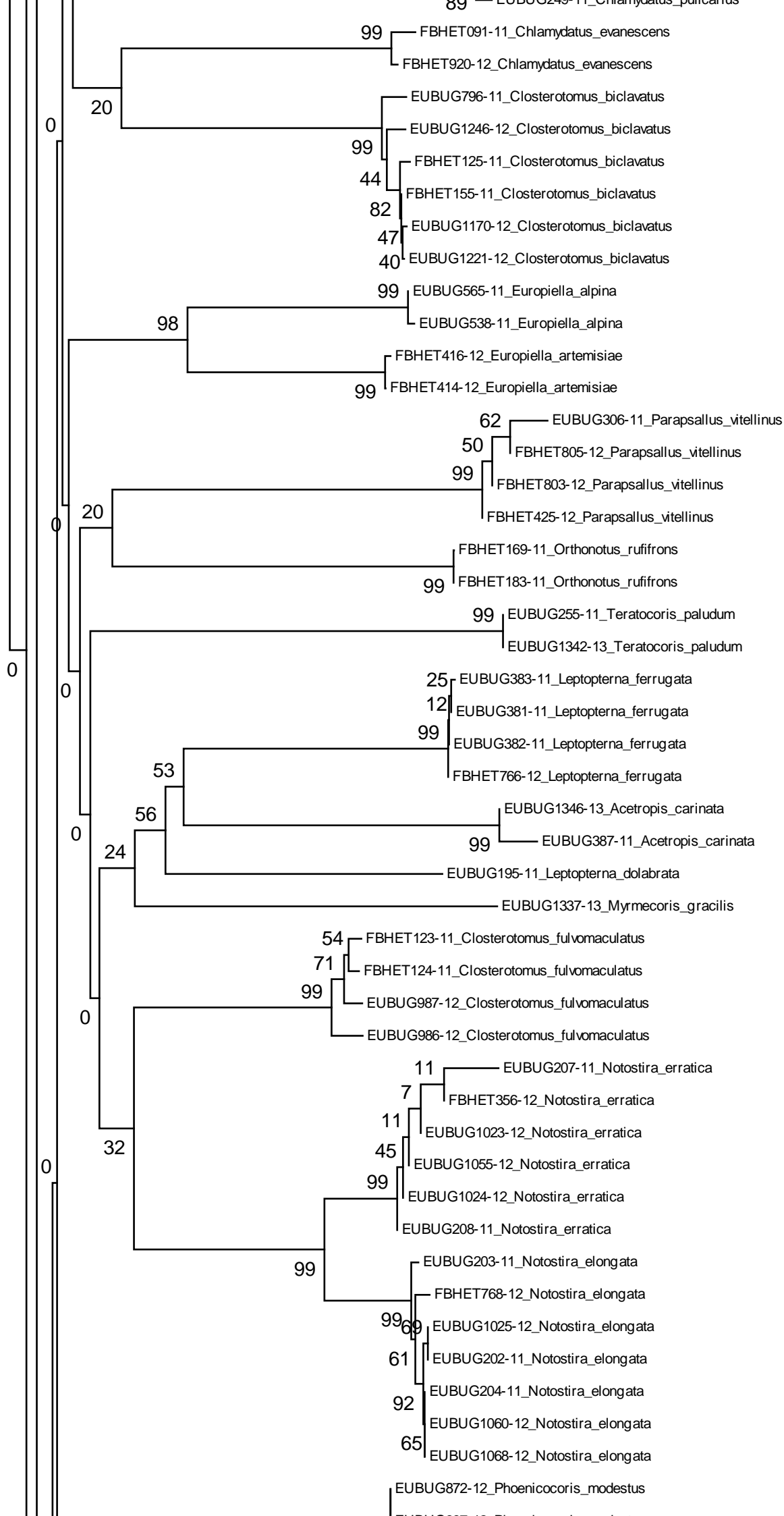

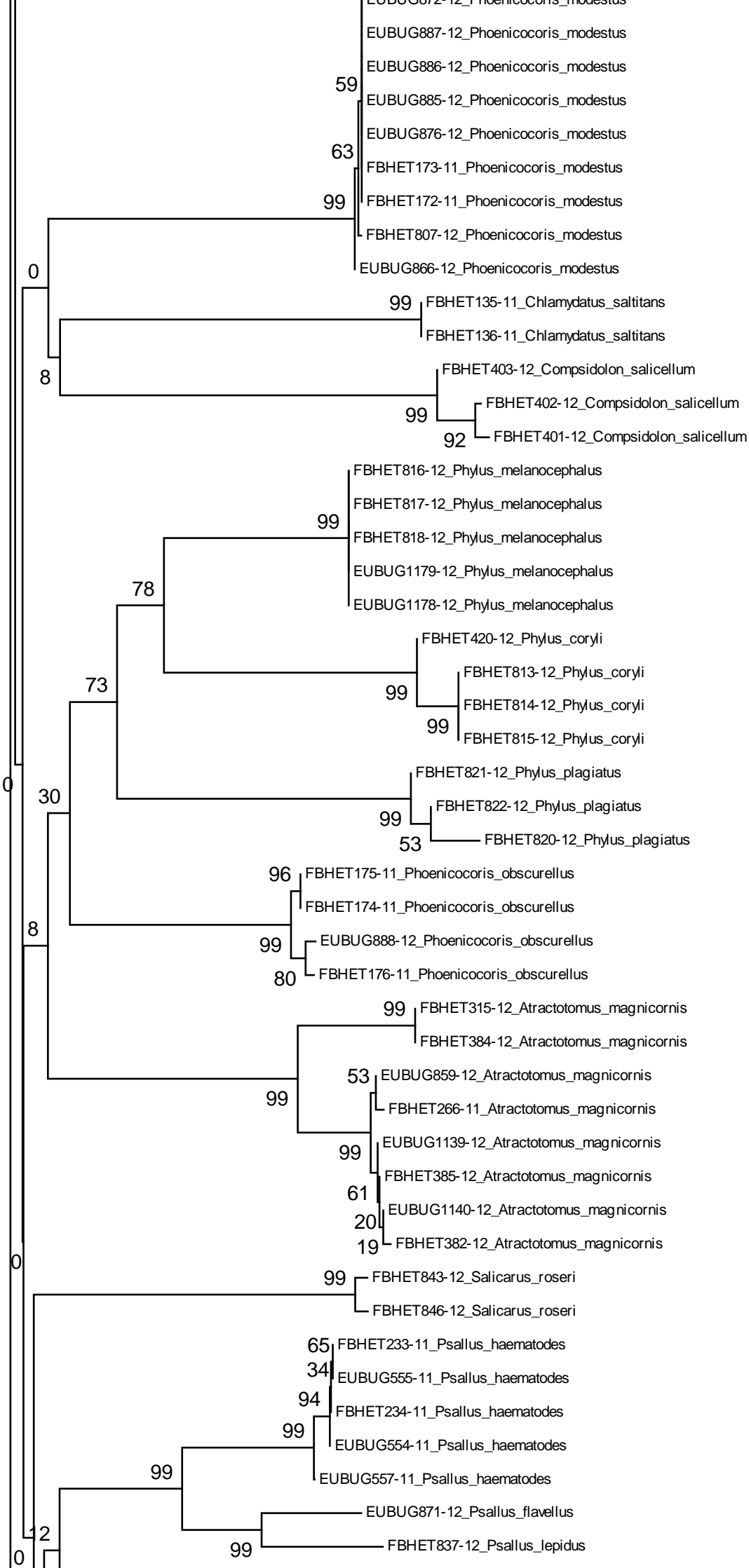

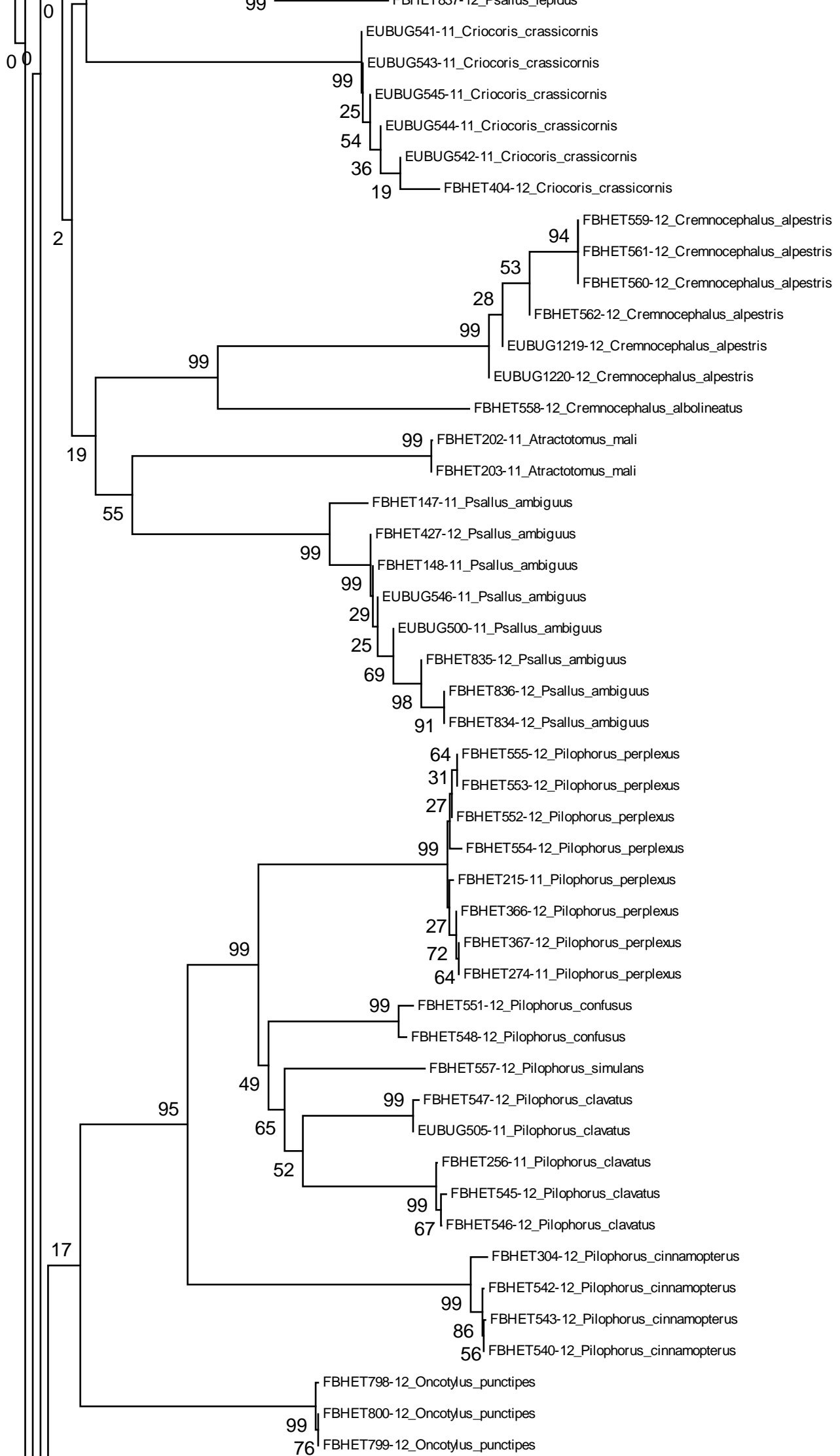



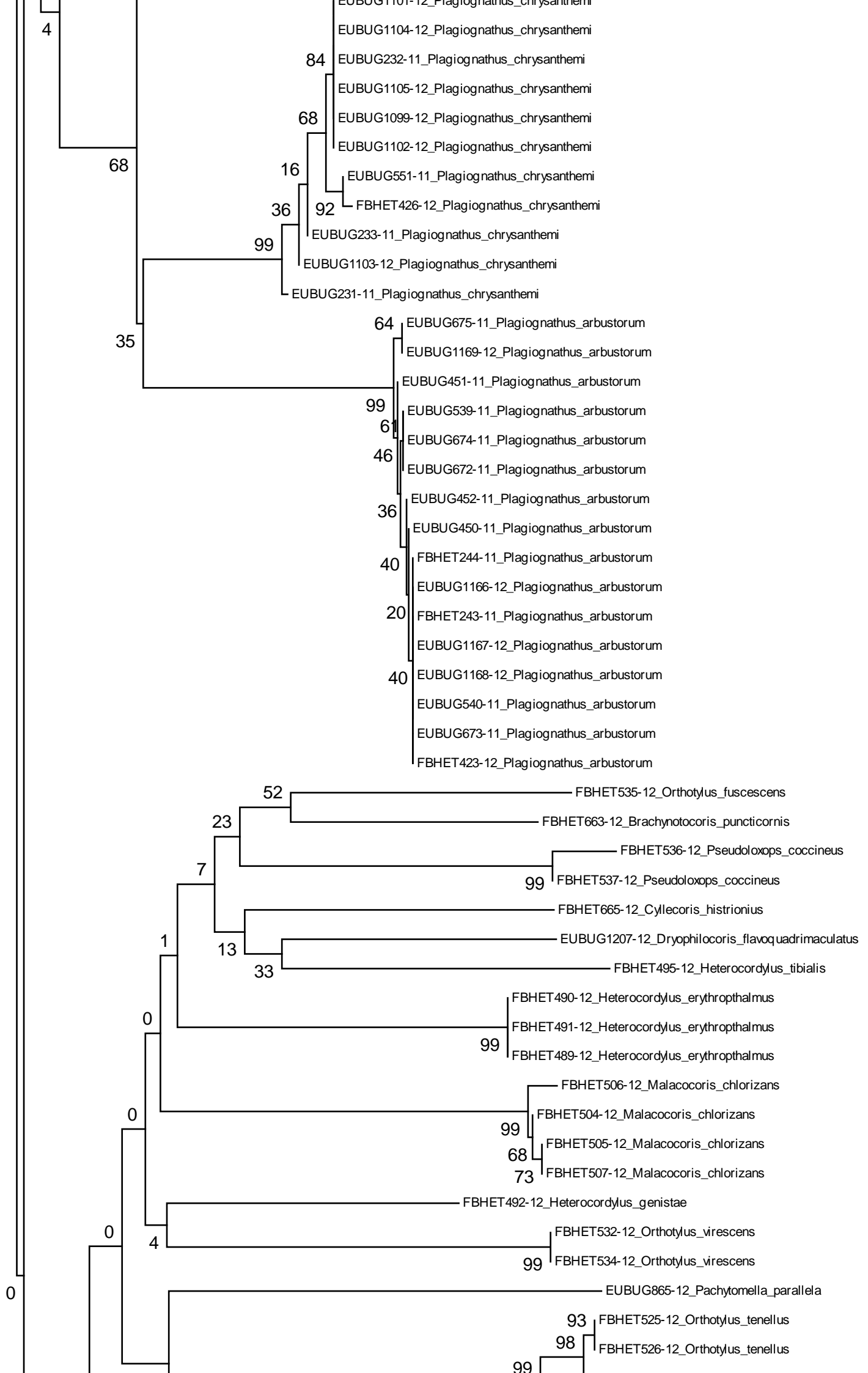

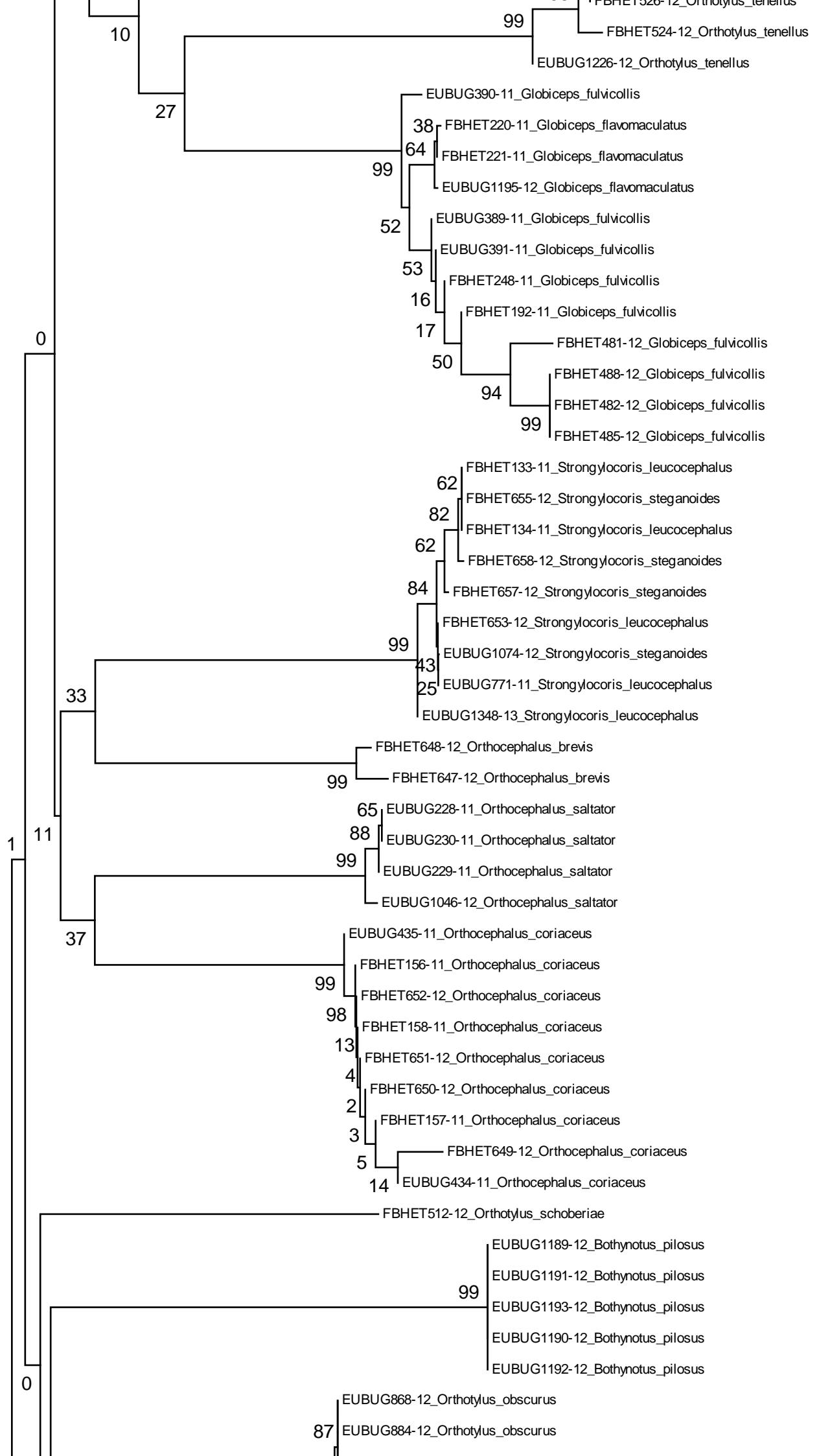

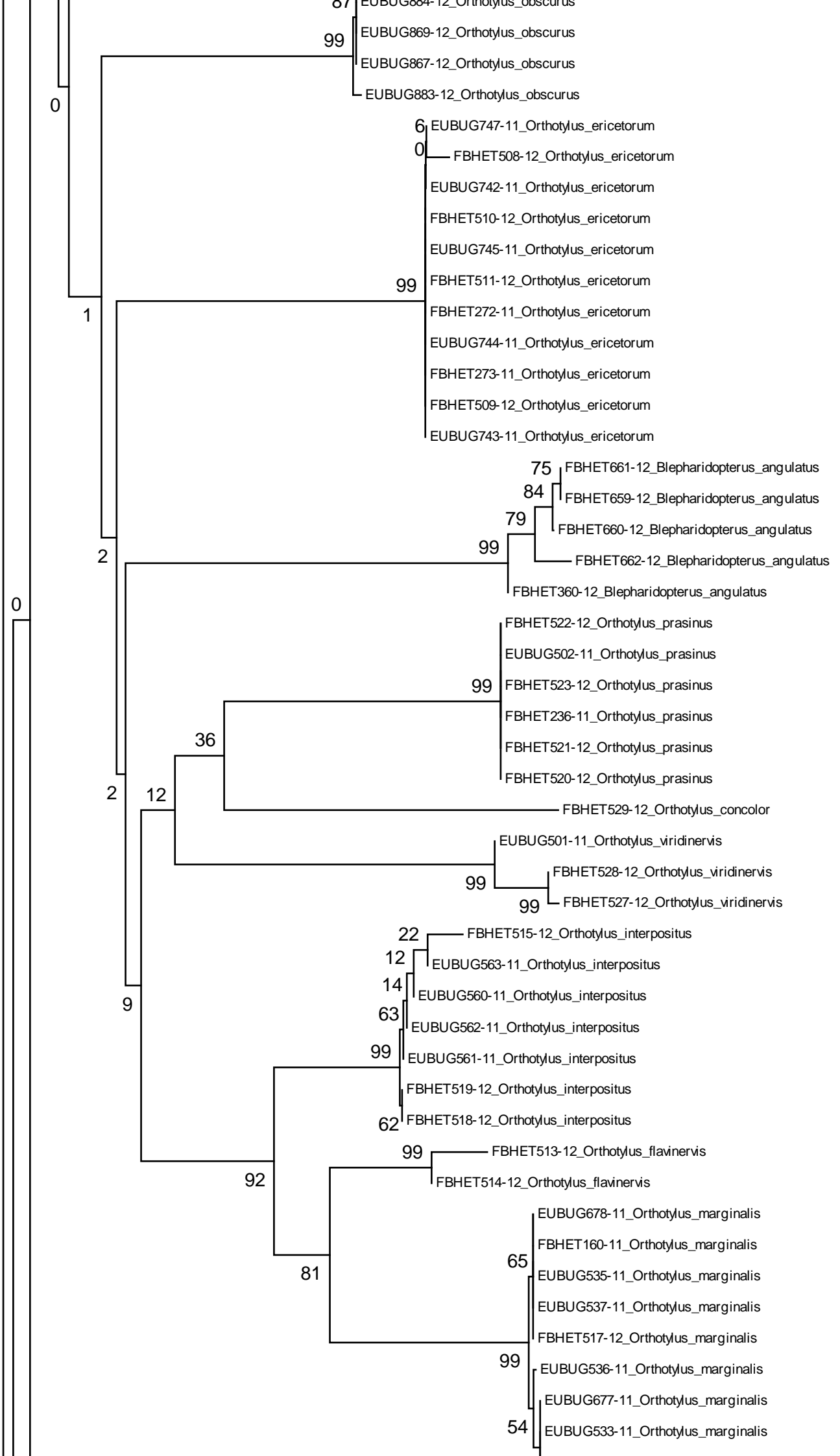

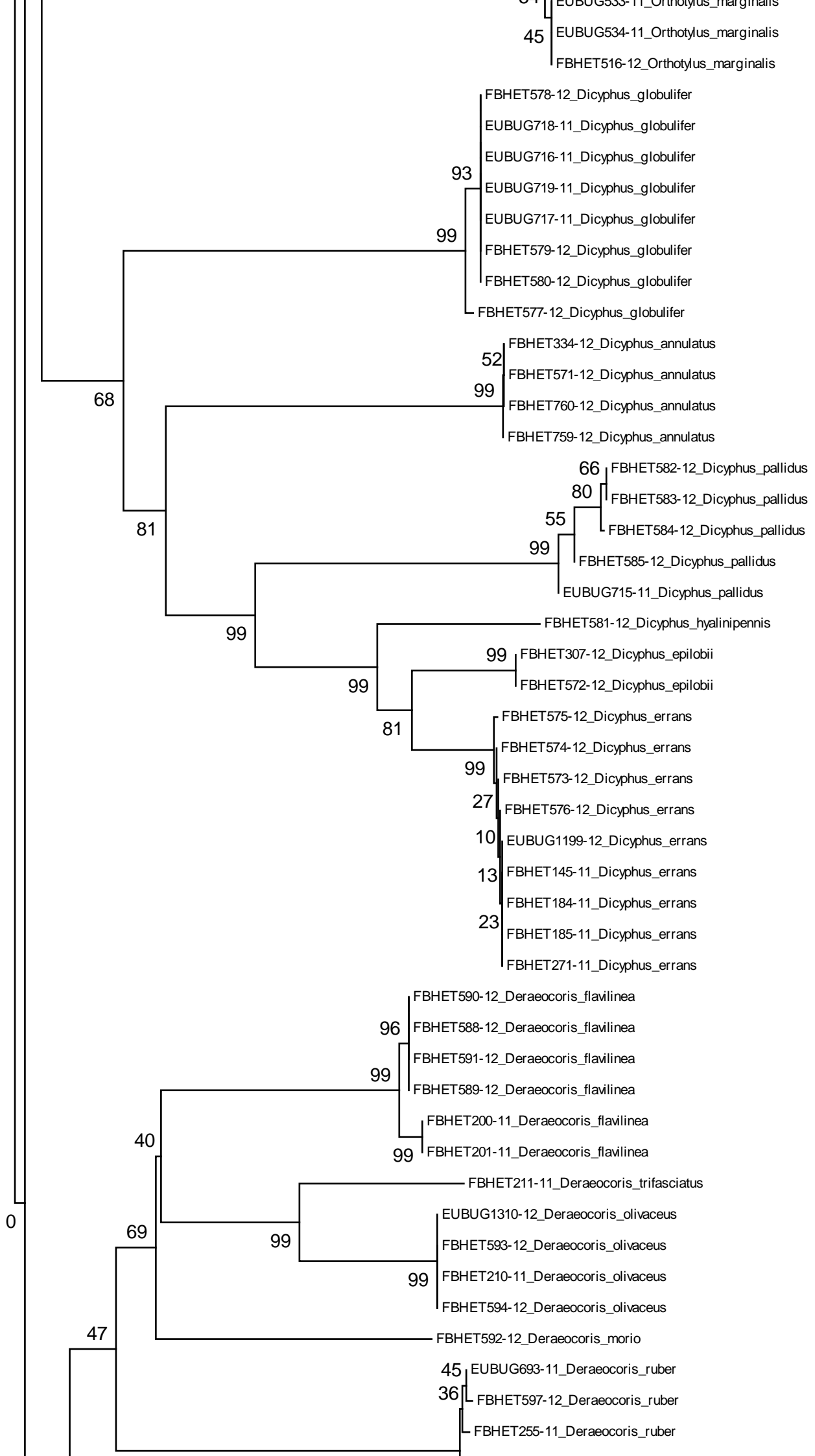

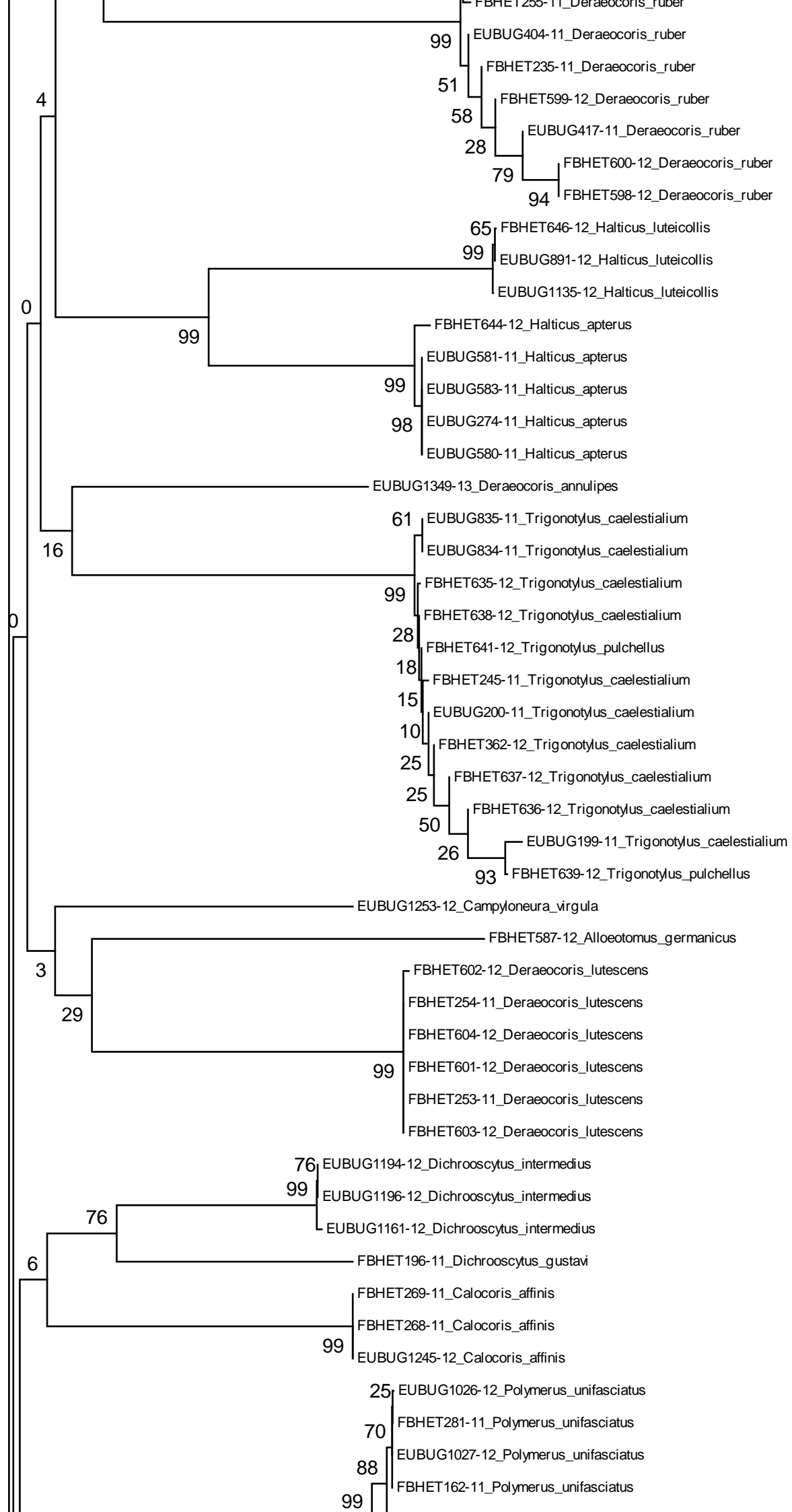

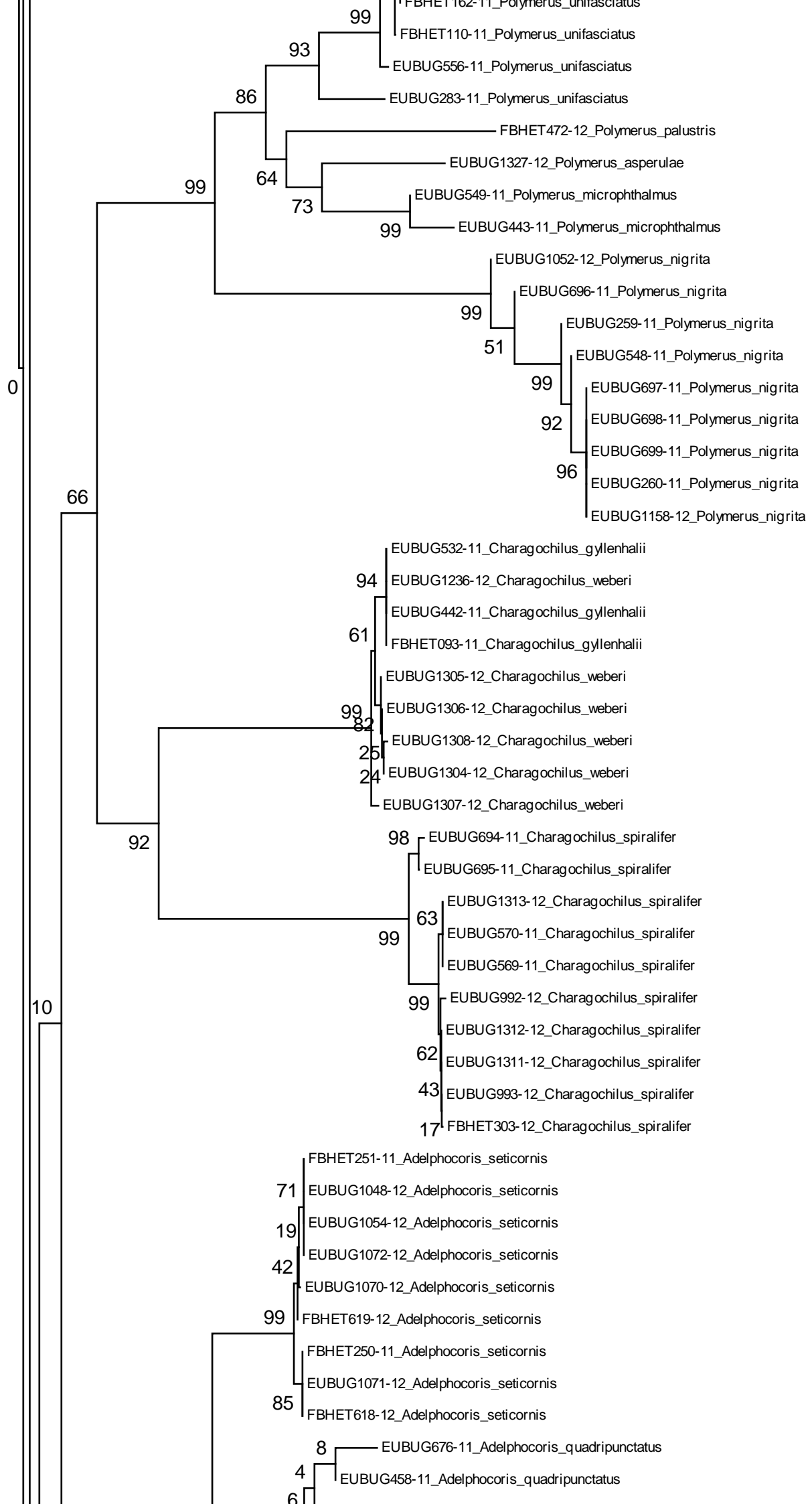

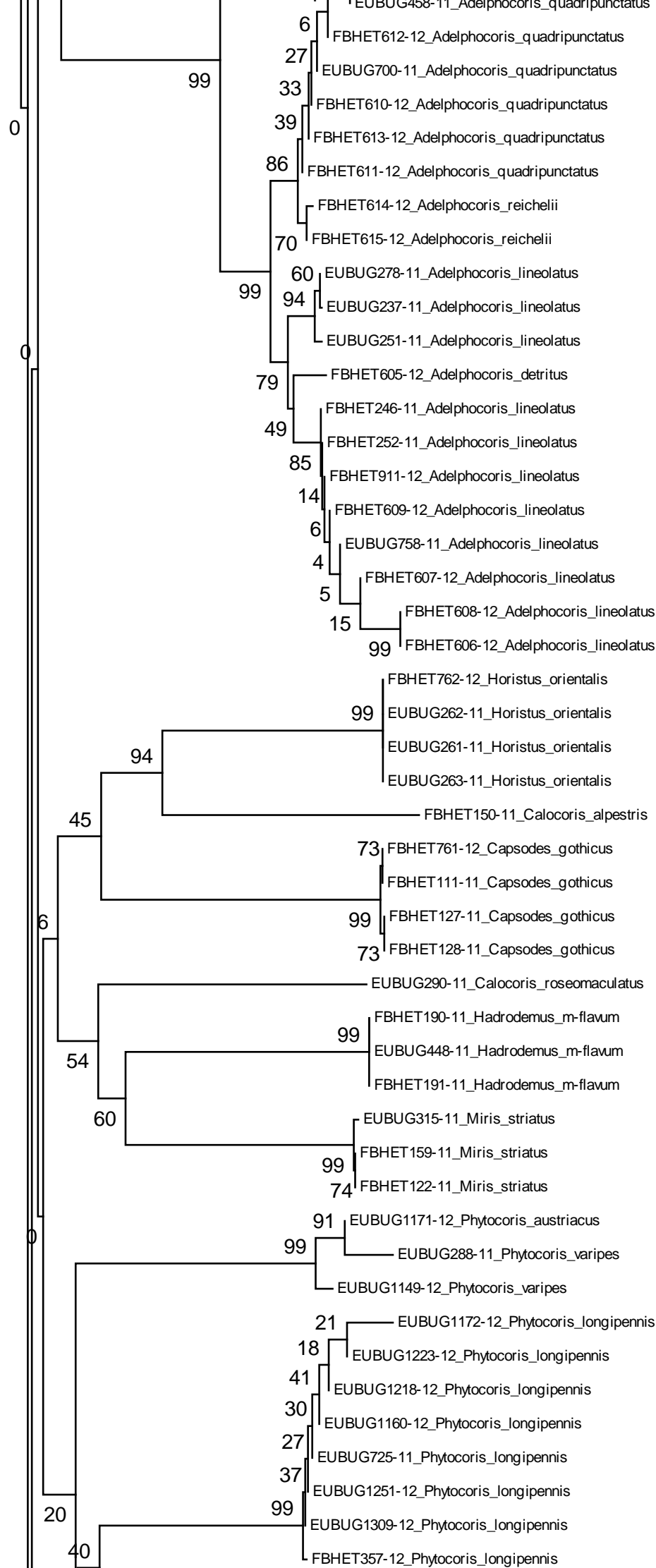

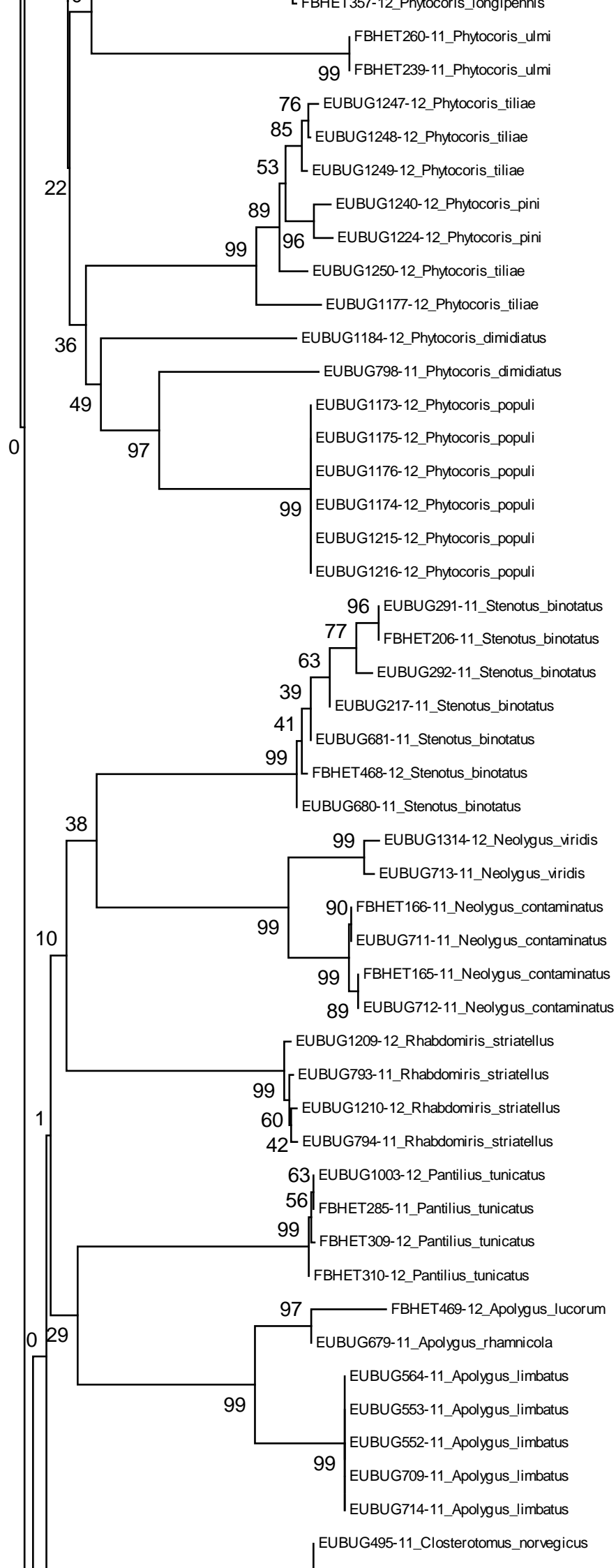

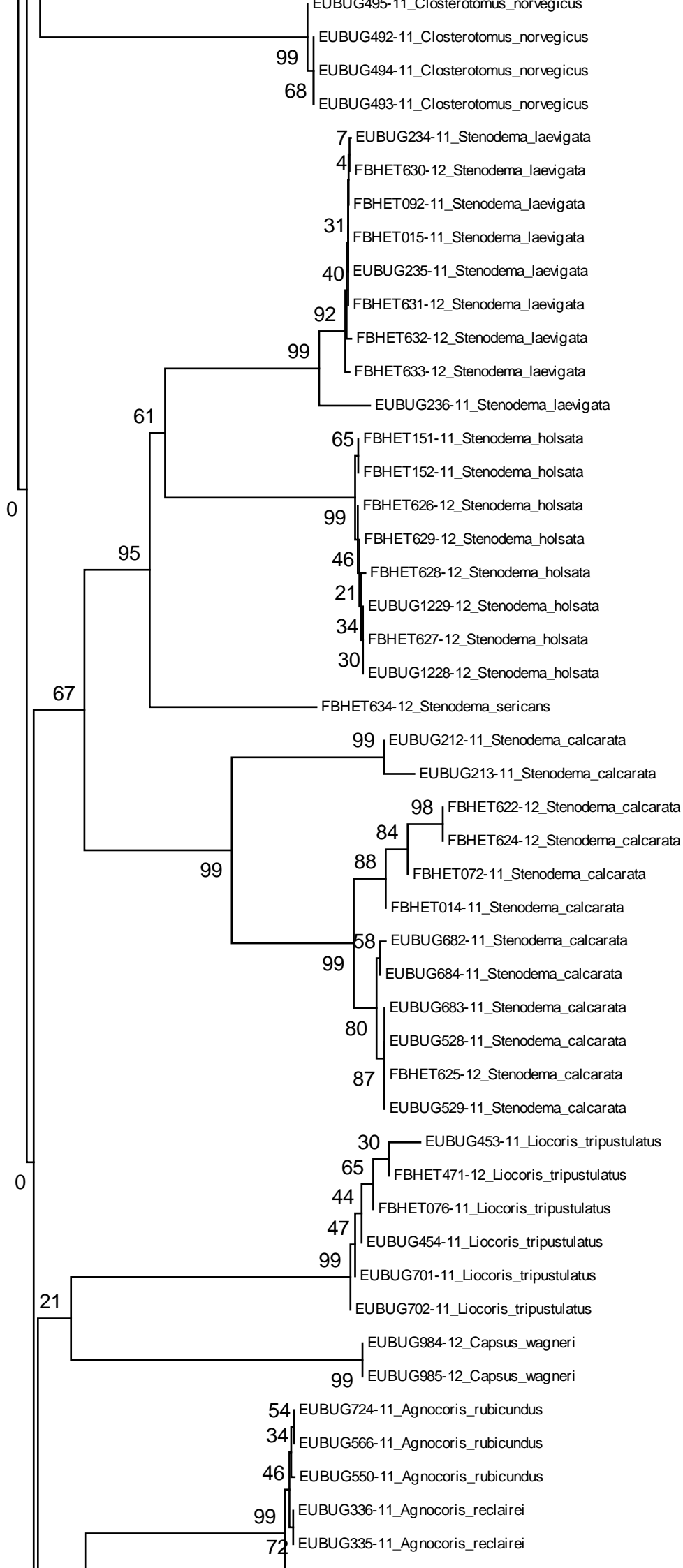

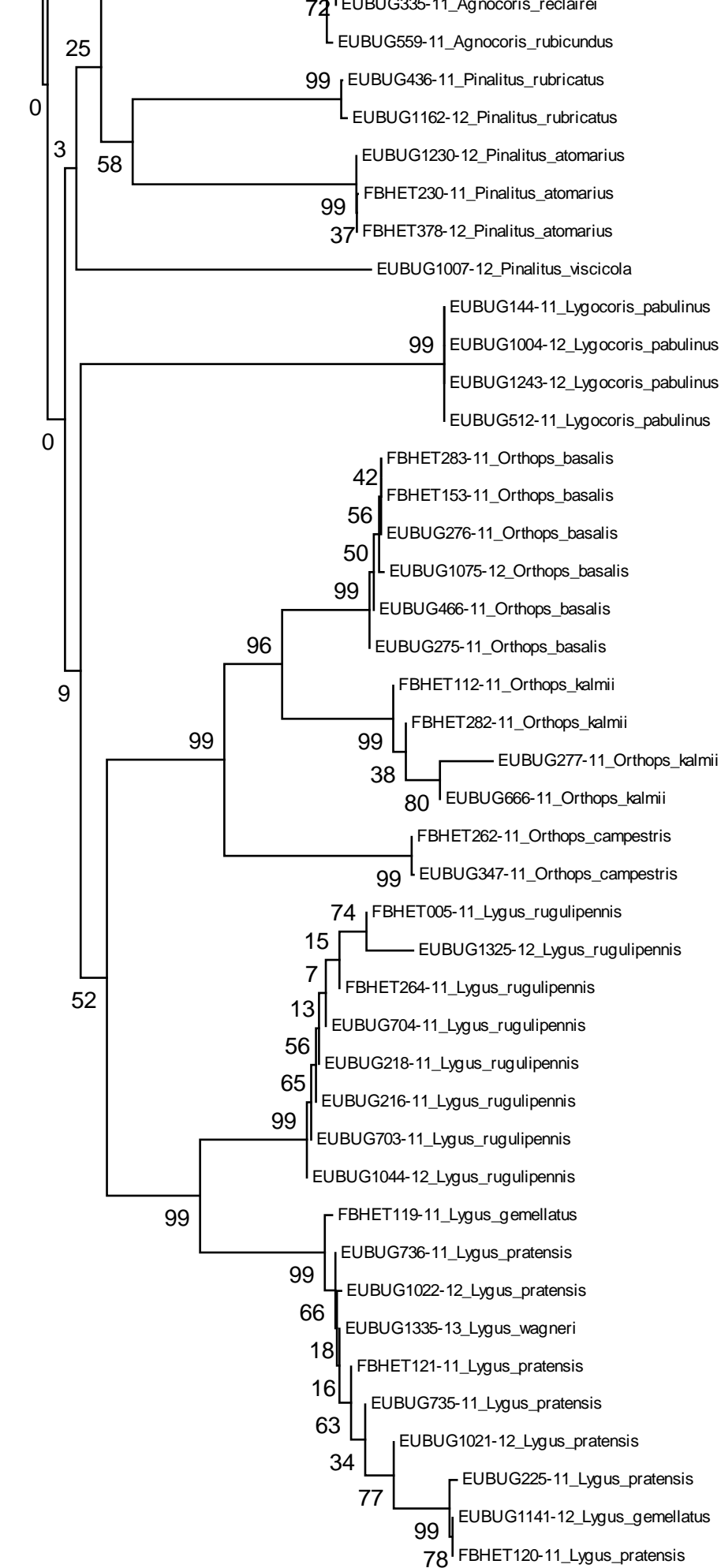

0.02

Supplement: Appendix S9 — Complete Neighbour joining topology of all analyzed specimens of the Heteroptera based on Kimura 2-parameter distances. Branches with specimen ID-number from BOLD and species name. Numbers next to internal branches are bootstrap values (1000 replicates, in %). (PDF) [file pone.0106940.s009.pdf]
