# Supplementary material for: Building-Up of a DNA Barcode Library for True Bugs (Insecta: Hemiptera: Heteroptera) of Germany Reveals Taxonomic Uncertainties and Surprises
Source: PLoS One. 2014 Sep 9;9(9):e106940. doi: 10.1371/journal.pone.0106940 (PMC4159288; doi:10.1371/journal.pone.0106940)
Supplement: Appendix S10 — Table of all non-monophyletic species/lineages of the analyzed data set. (DOCX) [file pone.0106940.s010.docx]

Non-monophyletic species of the analyzed Heteroptera:

| Species | Familiy |
| --- | --- |
| *Adelphocoris lineolatus* | Miridae |
| *Agnocoris rubicundus* | Miridae |
| *Agnocoris reclairei* | Miridae |
| *Arocatus roeselii* | Lygaeidae |
| *Arocatus longiceps* | Lygaeidae |
| *Charagochilus gyllenhalii* | Miridae |
| *Charagochilus weberi* | Miridae |
| *Elasmostethus interstinctus* | Acanthosomatidae |
| *Eurygaster maura* | Scutelleridae |
| *Eurygaster testudinaria* | Scutelleridae |
| *Globiceps fulvicollis* | Miridae |
| *Lygus gemellatus* | Miridae |
| *Lygus pratensis* | Miridae |
| *Megalonotus chiragra* | Rhyparochromidae |
| *Nabis brevis* | Nabidae |
| *Nabis ericetorum* | Nabidae |
| *Nabis pseudoferus* | Nabidae |
| *Nabis rugosus* | Nabidae |
| *Phytocoris varipes* | Miridae |
| *Phytocoris tiliae* | Miridae |
| *Pilophorus clavatus* | Miridae |
| *Strongylocoris leucocephalus* | Miridae |
| *Strongylocoris steganoides* | Miridae |
| *Trapezonotus dispar* | Rhyparochromidae |
| *Trigonotylus pulchellus* | Miridae |
| *Trigonotylus caelestialium* | Miridae |
