# Supplementary material for: Building-Up of a DNA Barcode Library for True Bugs (Insecta: Hemiptera: Heteroptera) of Germany Reveals Taxonomic Uncertainties and Surprises
Source: PLoS One. 2014 Sep 9;9(9):e106940. doi: 10.1371/journal.pone.0106940 (PMC4159288; doi:10.1371/journal.pone.0106940)
Supplement: Supplementary file 9 [file pone.0106940.s004.docx]

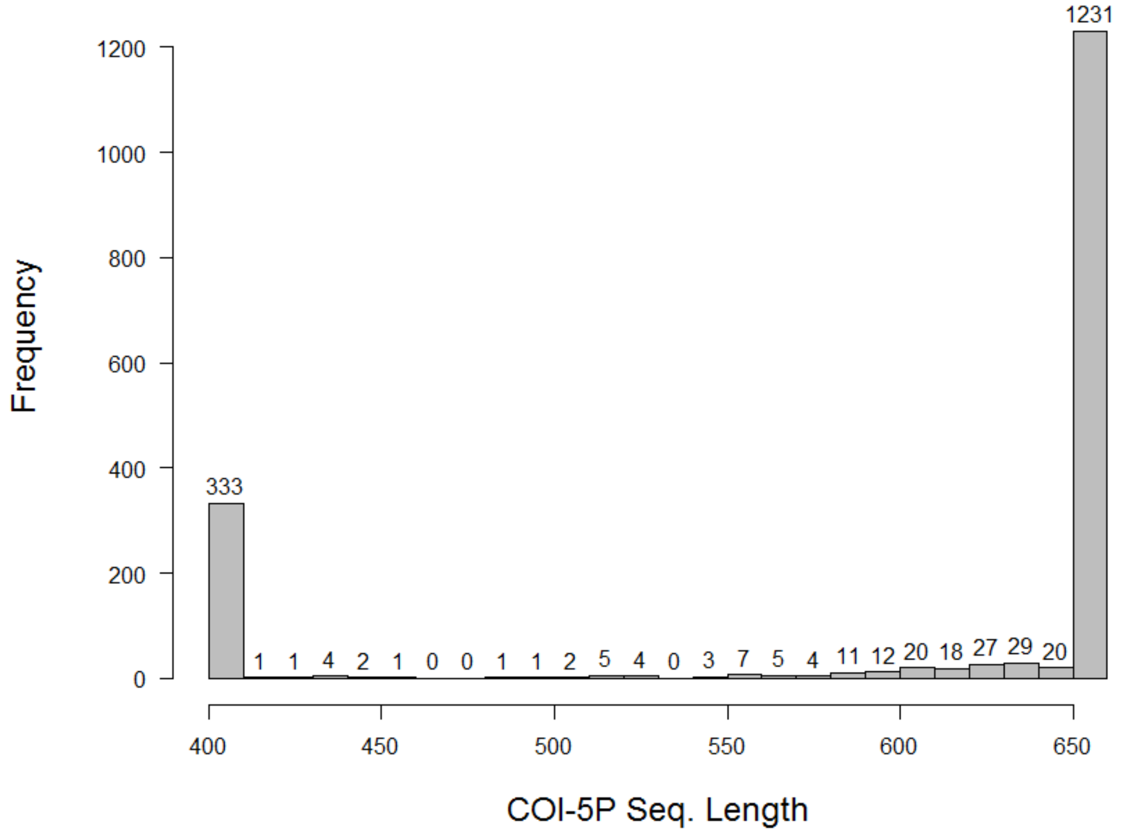

Frequency
COI-5P Seq. Length
1200
1000
800
600
400
200
0
400
450
500
550
600
650
333
1
1
4
2
1
0
0
1
1
2
5
4
0
3
7
5
4
11
12
20
18
27
29
20
1231
