# Supplementary material for: Building-Up of a DNA Barcode Library for True Bugs (Insecta: Hemiptera: Heteroptera) of Germany Reveals Taxonomic Uncertainties and Surprises
Source: PLoS One. 2014 Sep 9;9(9):e106940. doi: 10.1371/journal.pone.0106940 (PMC4159288; doi:10.1371/journal.pone.0106940)
Supplement: Supplementary file 10 [file pone.0106940.s005.docx]

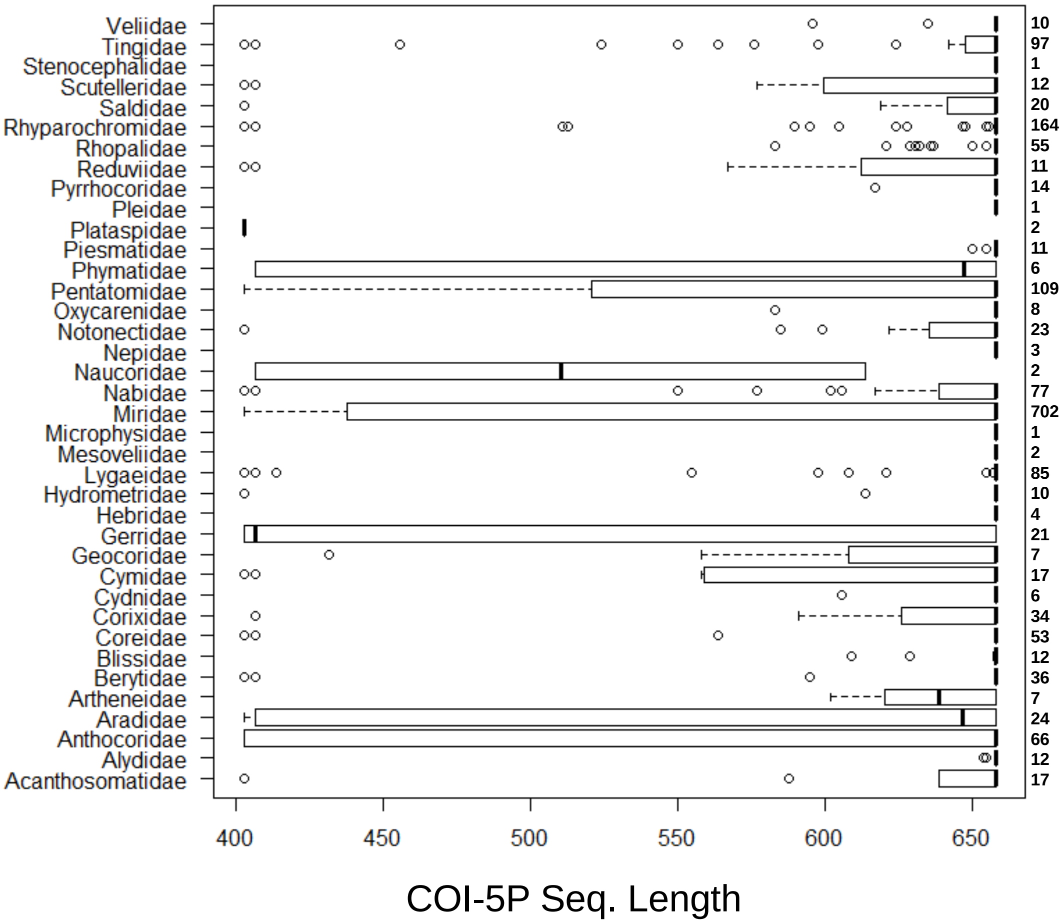
Veliidae
Tingidae
Stenocephalidae
Scutelleridae
Saldidae
Rhyparochromidae
Rhopalidae
Reduviidae
Pyrrhocoridae
Pleidae
Plataspidae
Piesmatidae
Phymatidae
Pentatomidae
Oxycarenidae
Notonectidae
Nepidae
Naucoridae
Nabidae
Miridae
Microphysidae
Mesoveliidae
Lygaeidae
Hydrometridae
Hebridae
Gerridae
Geocoridae
Cymidae
Cydnidae
Corixidae
Coreidae
Blissidae
Berytidae
Artheneidae
Aradidae
Anthocoridae
Alydidae
Acanthosomatidae
10
97
1
12
20
164
55
11
14
1
2
11
6
109
8
23
3
2
77
702
1
2
85
10
4
21
7
17
6
34
53
12
36
7
24
66
12
17
400
450
500
550
600
650
COI-5P Seq. Length
